# When local governments’ stay-at-home orders meet the white house’s “opening up america again”

**DOI:** 10.1371/journal.pone.0298115

**Published:** 2024-03-20

**Authors:** Reza Mousavi, Bin Gu

**Affiliations:** 1 McIntire School of Commerce, University of Virginia, Charlottesville, Virginia, United States of America; 2 Questrom School of Business, Boston University, Boston, Massachusetts, United States of America; Central University of Finance and Economics, CHINA

## Abstract

In the current polarized political climate, citizens frequently face conflicting directives from their local and federal government officials. For instance, on April 16^th^, 2020, The White House launched the “Opening up America Again” (OuAA) campaign while many U.S. counties had stay-at-home orders. We created a panel data set of U.S. counties to study the impact of U.S. counties’ stay-at-home orders on community mobility before and after The White House’s campaign to reopen the country. Our results suggest that before the OuAA campaign, stay-at-home orders substantially decreased the time spent in retail and recreation businesses. However, after the launch of the OuAA campaign, the time spent at retail and recreational businesses in a typical conservative county increased significantly more than in liberal counties (23% increase in a typical conservative county vs. 9% increase in a typical liberal county). We also found that in conservative counties with stay-at-home orders, time spent at retail and recreational businesses increased less than in those without stay-at-home orders. These findings illuminate that when federal and local government policies are at odds, residents decide which policies to adhere to based on the alignment between their political ideology and the government body. Our findings highlight the substantial importance of each government body in forming citizens’ behaviors, offering practical implications for policy makers during natural disasters.

## Introduction

The COVID-19 pandemic has been raging in the world since December 2019. The immense impact of the COVID-19 pandemic on the lives of billions of people has forced authorities to devise response strategies to contain the damage. The authorities have devised various response strategies, from enforcing stay-at-home orders to restricting public and private gatherings to wearing masks and social distancing. These restrictive measures are effective only if they are adhered to by citizens. Given the diverse set of opinions citizens hold, their level of adherence to the restrictions could be different. Initial research studies provide evidence of these differences based on residents’ political beliefs. For instance, Painter and Qiu [1] found that residents’ political views affect their compliance with social distancing orders imposed in response to the spread of COVID-19. Their findings reveal that counties that voted for President Trump in the 2016 presidential election are more likely to disobey the social distancing orders. Another study by Grossman et al. [2] also revealed that the Democratic counties were likelier to obey stay-at-home orders enforced by the state governors. They also found that Democratic counties with Republican governors are more likely to stay at home when compared to other counties. Overall, the current research signals the impact of stay-at-home orders on residents’ stay-at-home behaviors. Although both studies offer similar findings, they use SafeGraph’s shelter-in-place data. This data set uses a sample of users’ cell phone locations to determine the time stayed at home. However, we believe that another data set published by Google would be more informative as it includes not only data about the amount of time people spent at home, but also data about the amount of time people spent in retail & recreation locations (such as bars, restaurants, and gyms), grocery stores, parks, places of work, and transit locations. We believe such data would be more informative in measuring the impact of stay-at-home orders.

Another important distinction between our research and prior studies is that we include the federal government’s intervention during and after the local governments’ stay-at-home orders. One unique difference between our study and the previous studies is that we factor in the potential effects of the federal government’s intervention as we study the effect of local government interventions. This setting would allow us to identify the potential misalignment between the two interventions and measure its impact on community compliance during natural disasters such as the COVID-19 pandemic. In particular, while many local governments still had stay-at-home orders in place, the federal government intervened: “[w]e are starting our life AGAIN!,” [3] said the U.S. president during his Coronavirus Task Force press briefing on April 16^th^. A day earlier, during another press briefing, the president claimed that the U.S. has “passed the pick on new cases.”[4] Yet a couple of days before that, on April 13^th^, he claimed “total authority” [3] over governors. The three Coronavirus Task Force press briefings on April 13^th^, 15^th^, and 16^th^ mark a significant shift in the U.S. president’s response policy regarding the pandemic. What followed this new policy was a set of guidelines to open up America. The White House launched the “Opening up America Again” (OuAA) website on April 16^th^. Along with these guidelines, President Trump used his powerful Twitter account to encourage protestors (mainly composed of his supporters) to “liberate” Michigan and Minnesota, two states with Democratic governors who imposed strict social distancing restrictions [5].

We are of the conviction that this particular perspective is not only critical and important but also represents a largely uncharted territory in academic research. While existing literature includes examinations of the collaborative dynamics between federal and local governments in the context of disaster management [5,6], our review indicates a significant gap in studies specifically addressing the concurrent impacts of conflicting federal and local policies on the affected communities. To our best knowledge, this aspect of policy analysis—examining the simultaneous and potentially opposing influences of different government levels on public behavior and outcomes—remains largely unexplored. This oversight in existing research underscores the novelty and relevance of our study, as it seeks to fill this crucial gap by providing insights into the complex interplay between contradictory policies from different government tiers during public health crises such as the COVID-19 pandemic.

Furthermore, our study delves into the intricate ways in which the political orientation of the population further enriches and complicates this relationship. We demonstrate that the political leanings of constituents add a layer of complexity and nuance to the interaction between federal and local policies. Specifically, our analysis reveals that the effectiveness and public reception of these policies are significantly influenced by the prevailing political attitudes within the community. This factor is crucial in understanding the varying degrees of compliance and resistance to health directives, reflecting a broader socio-political landscape where personal beliefs and affiliations shape responses to government interventions. In essence, our research not only highlights the unique challenges posed by conflicting government policies but also brings to light the pivotal role of political orientation in shaping the public’s reaction to such policies during unprecedented times like a pandemic.

In this study, we use weekly panel data about U.S. counties’ community mobility, unemployment rate, political orientation, and COVID-19 cases and deaths, along with stay-at-home and shelter-in-place restrictions to understand the impact of stay-at-home orders on community mobility and to what extent this impact is moderated by the political orientation of the county and by the OuAA campaign. Our findings reveal that:

1. Stay-at-home and shelter-in-place restrictions imposed by counties and states decreased time spent at retail & recreation places such as bars, restaurants, gyms, and movie theatres and increased time spent at residential places.
2. Liberal counties spent more time at home and less time at retail stores than conservative counties during the stay-at-home orders.
3. Liberal counties spent more time at home and less time at retail stores compared to conservative counties after the OuAA campaign launched by the White House.
4. Conservative counties with a stay-at-home order spent more time at home and less at retail & recreation places even after the OuAA campaign launch compared to conservative counties without stay-at-home orders.

Our results are based on a quasi-experimental setting. We have controlled the number of cases and deaths per 100k population, county-fixed effects, time-fixed effects, and the interaction of state and time-fixed effects. We also examined the robustness of our findings by running the models using a matched sample of counties. We used county-level data about residents’ education, population, deaths, births, domestic and international migration, percent below the federal poverty line, unemployment rate, and median household income. In this summary, we report our study design, data analysis, and preliminary findings.

## Materials (data)

We created a panel data set of U.S. counties by collecting data about community mobility scores, COVID-19 new cases and deaths (adjusted by population), the ideological orientation of counties, and state’s COVID-19 response data (stay-at-home, shelter-in-place, and other types of restrictions and their timelines) for the period between the first week of March 2020 and the first week of June 2020. From 3,141 U.S. counties, we removed any county that did not have a value for the variables in our models. For instance, many counties did not have a value for the community mobility indices (Retail and Residential described in Table 1). After removing the counties with substantial missing values, we ended up with panel data of 1,211 (1,563 in the robustness check) counties observed during 14 weeks. Below, we describe the sources of data and our data collection approach.

**Table 1:** Descriptive statistics of the variables.

Variable	Description	Count	Mean	Std.	Min	Max
*Dependent Variables*:						
Retail	Mobility trends for places like restaurants, cafes, shopping centers, theme parks, museums, libraries, and movie theaters.	16,443	-19.764	19.883	-86.571	100.33
Residential	Mobility trends for places of residence.	16,443	11.498	6.455	-3.400	31.428
*Independent Variables*:						
stay	Whether the county enforced a stay-at-home order during the focal week.	Not Enforced: 7,040 Enforced: 9,403				
post_reopen	Equals 1 on or after week of April 13^th^ and zero otherwise.	Before OuAA: 7,105 After OuAA: 9,338				
conservative	Ideological orientation of each county. This variable measures to what extent a county is conservative	16,443	0.650	0.149	0	1
*Control Variables*:						
cases	Number of daily new cases per 100k residents averaged per week. In some rare cases, the number of daily cases and deaths are negative. This is because of the adjustments made to the counts made for the previous days.	16,443	4.043	9.448	-14.670	389.368
deaths	Number of daily new deaths per 100k residents averaged per week	16,443	0.193	0.505	-1.030	8.993
unemployment	Weekly unemployment rate in the county	16,443	10.159	5.011	1.800	34.300
gathering50	Whether gatherings of 50 or more were banned in the county during the focal week.	Not Enforced: 3,500 Enforced: 12,943				
gathering500	Whether gatherings of 500 or more were banned in the county during the focal week.	Not Enforced: 2,710 Enforced: 13,733				
dine_in	Whether restaurant dine-ins were prohibited in the county during the focal week.	Not Enforced: 5,374 Enforced: 11,069				
gym	Whether entertainment businesses and gyms were closed in the county during the focal week.	Not Enforced: 5,298 Enforced: 11,145				

### Community mobility data

This data set was obtained from Google [6]. Google’s community mobility dataset “shows how visits to places, such as grocery stores and parks, are changing in each geographic region.”[6] The documentations show how visits and length of stay at different places change compared to a baseline. The baseline is the median value, for the corresponding day of the week, during the 5 weeks from January 3^rd^, 2020, to February 6th, 2020. Google indicates that the data is included in the calculations based on user settings, connectivity, and whether there is any privacy concern (due to the small sample size in some areas). If there are any concerns regarding the privacy of Google’s users, the data fields will be left empty. Due to these omitted values, we did not include counties with missing values.

Google’s community mobility data set includes six categories: grocery & pharmacy, parks, transit stations, retail & recreation, residential, and workplaces. We used retail & recreation, and residential categories from these six categories. The reason why we limited our study to these two categories is that these two categories portray a more accurate picture of community mobility trends during the pandemic. The retail & recreation category includes mobility trends for places like restaurants, cafes, shopping centers, theme parks, museums, libraries, and movie theatres. These businesses are non-essential businesses that could be avoided during the pandemic. If stay-at-home orders are effective, we expect a decrease in retail & recreation trend. The residential category refers to the mobility trends of places of residence. If stay-at-home orders are effective, we expect an upward trend in the residential category. Other categories, such as grocery & pharmacy stores, workplaces, and transit stations, are either essential or determined by the employers rather than the residents themselves. Therefore, trends in grocery & pharmacy stores, workplaces, and transit stations may not provide a reasonable pattern about residents’ will to adhere to stay-at-home orders and other restrictions. We also excluded park mobility trends because visiting such places would be possible with minimal risk of infection (people could stay six feet apart in the open areas). In summary, we believe that the amount of time people spent at retail and recreational places as well as the amount of time they spent in residential places reflects their voluntary compliance with the stay-at-home orders. As we discussed above, the other indices in Google community mobility report either do not reflect voluntary compliance (such as workplace) or do not reflect compliance (such as the amount of time people spent at parks). Hence, we created two dependent variables, *Retail* and *Residential*, based on Google’s mobility trends for retail & recreation and residential places.

### COVID-19 data

This data set was obtained from NY Times’ GitHub page [7]. This dataset includes the number of new cases and the number of new deaths per day per county per 100k residents. We aggregated this data by taking the means of daily new cases and daily new deaths each week.

### Ideological orientation data

This data set was obtained from American Ideology Project [8]. We used the 2022 release of “County-Level Preference Estimates.” From this data file, we used the variable mrp_mean, which is the estimate of the mean ideology of the county. This measure ranges from a negative number to a positive number. The smallest value in mrp_mean represents the most liberal county, while the largest value in mrp_mean is associated with the most conservative county. Therefore, we can interpret mrp_mean as a metric for gauging the level of the conservativeness of a county. Hence, in our study, we call this variable *conservative in* the 2022 release of the data, *conservative* ranges from-1.098 (the least conservative county) to 0.842 (the most conservative county). We used Min-Max transformation to transform the scale to range from zero (most liberal) to 1 (most conservative). The methodology for estimating ideological orientation scores is described in [9].

### County-level restrictions data

This data set was obtained from a GitHub repository [10]. The data summaries and the methods used for assembling the data sets are detailed in [11]. There are multiple data files in the repository, from which we used the “interventions.csv” data file. This data file contains the dates that counties (or states governing them) enforced policies (such as stay-at-home orders) to mitigate the spread of COVID-19 by restricting community mobility or gatherings. In addition to the dates of policy enforcement initiations, this data set includes the dates the policies were rolled back. From this data set, we used five types of restrictions:

1. Type 1: Stay-at-home orders
2. Type 2: Prohibiting gatherings of 50 or more people
3. Type 3: Prohibiting gatherings of 500 or more people
4. Type 4: Prohibiting dine-in restaurants and bars
5. Type 5: Closing entertainment businesses and gyms

Type 1 is the main variable of interest in our study. We use Type 2 through Type 5 as control variables in our models.

### County-level socio-economic data

This data set was obtained from The U.S. Department of Agriculture (USDA) [12] and included information about the socio-economic indicators at the county level. In particular, this data set provides information about education level and population estimates, including national and international migration, poverty, and unemployment.

### Variables

**Fig 1:**
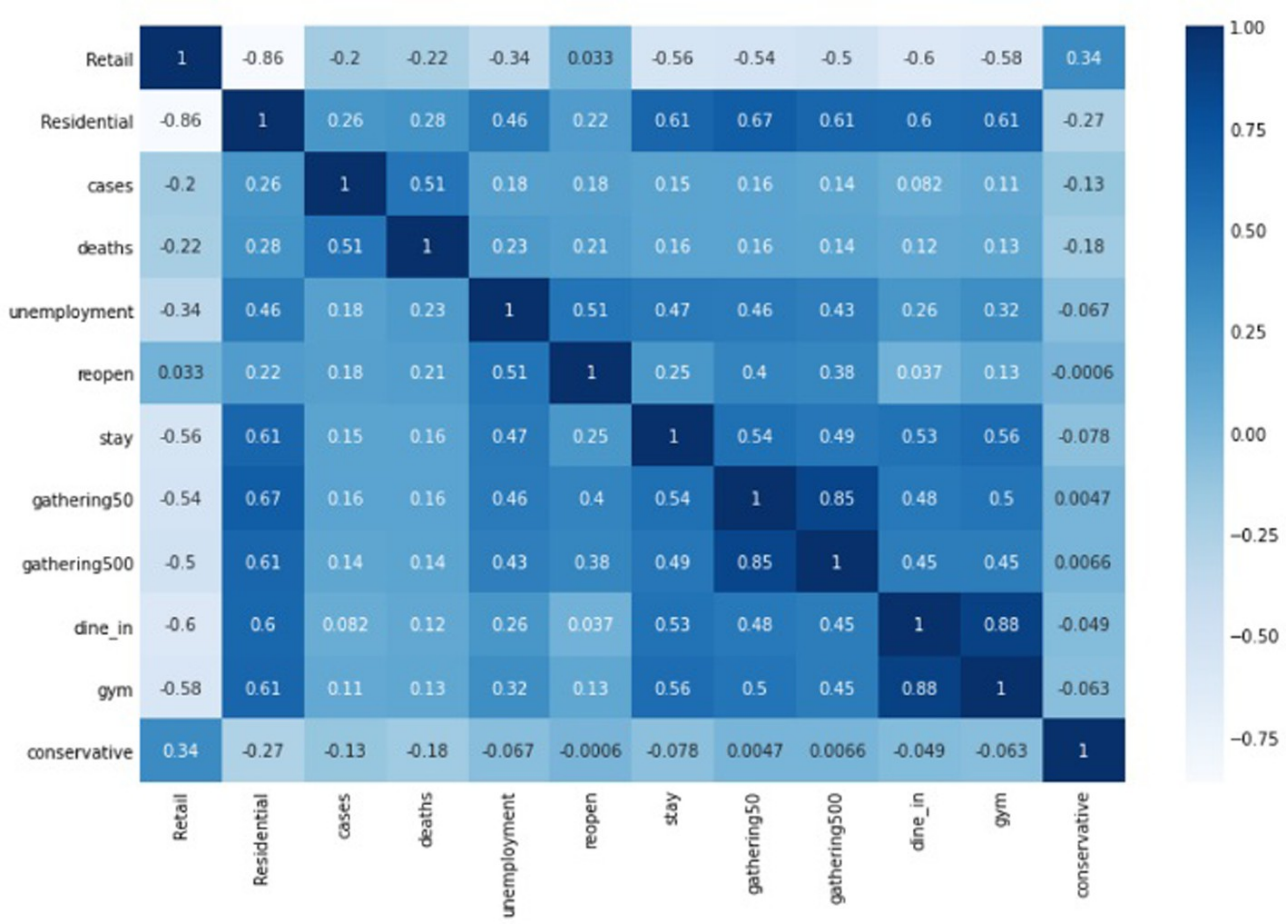
Correlation matrix (correlation coefficients).

Table 1 reports the variables’ list, descriptions, and summary statistics. Dependent variables are floored (at 0.005) and capped (at 0.995) to treat the outliers. Fig 1 reports the correlation coefficients. Our data set includes data about 1,211 counties over 14 weeks (from the 10^th^ week of 2020 through the 23^rd^ week of 2020). This period covers the first peak in the number of cases in the U.S. and includes the time before and after the rollout of the OuAA campaign by The White House. Furthermore, many counties and states enforced stay-at-home orders during this period. Some of those restrictions were lifted again in the same time frame of our study.

We excluded observations with any missing value from the data set. This resulted in 16,443 observations with complete data. The community mobility indices (*Retail* and *Residential*) were obtained from Google’s Community Mobility Report. According to Google, these data are based on “data from users who have opted-in to Location History for their Google Account.” [6] *Retail* and *Residential* indices reflect the change in users’ locations based on a baseline. Per Google’s documentation, the baseline is the median value, for the corresponding day of the week, during the five weeks from January 3^rd^ through February 6^th^ of 2020. A negative value for *Retail* means that users spent less time in retail stores compared to the baseline timeframe. A positive score for *Residential* implies that the users spent more time at a residential location (i.e., home) compared to the baseline timeframe.

**Fig 2:**
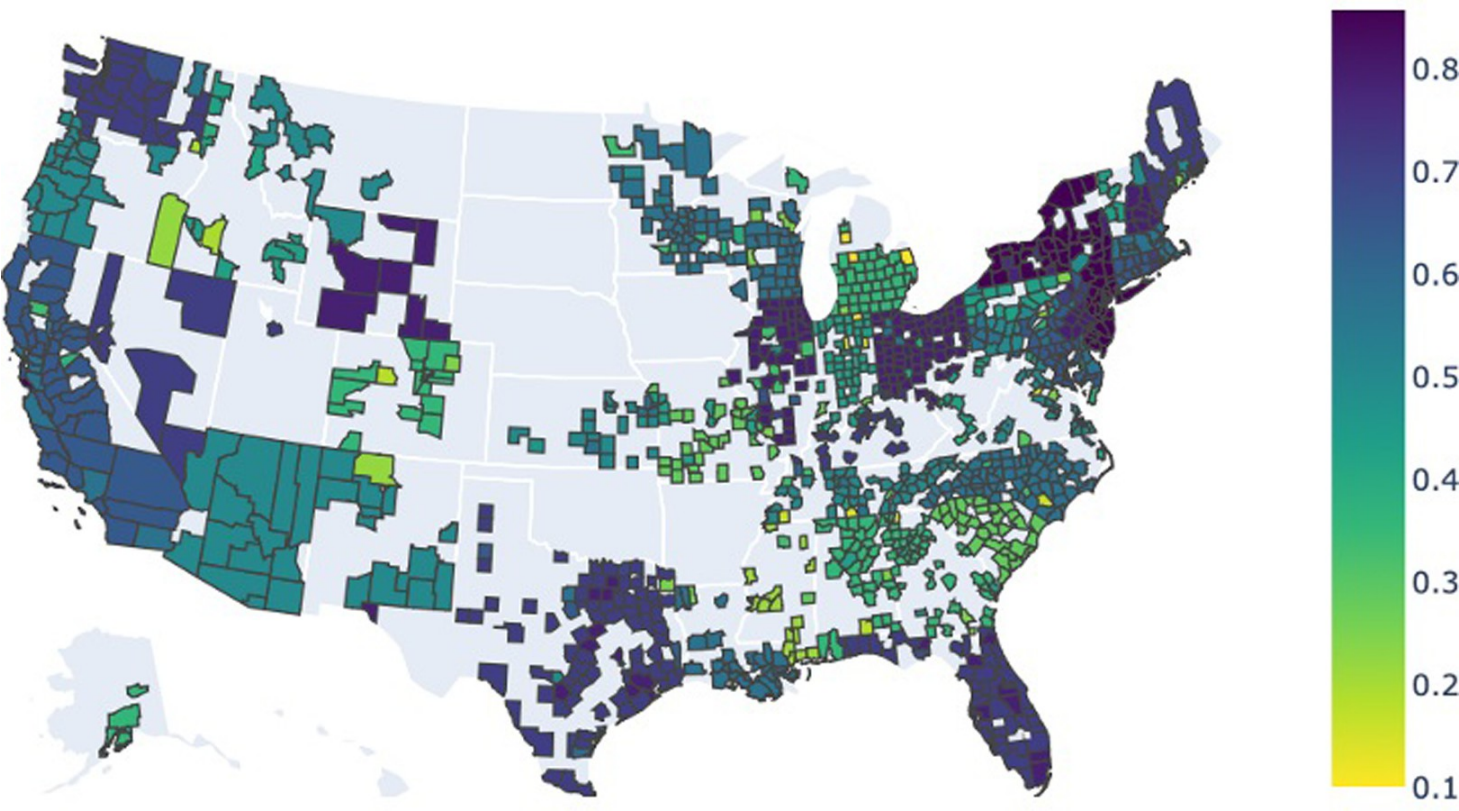
Average county-level stay-at-home restrictions color-coded based on longevity of orders (counties with darker colors enforced stay-at-home orders for a more extended period) © plotly (https://github.com/plotly/datasets/blob/master/LICENSE) MIT License.

Fig 2 visualizes the longevity of stay-at-home orders during our study period. Lighter colors mean that the stay-at-home orders were in place for a short period (or not enforced at all), while darker colors represent longer stay-at-home orders. This plot only includes the counties we used in our analysis.

**Fig 3:**
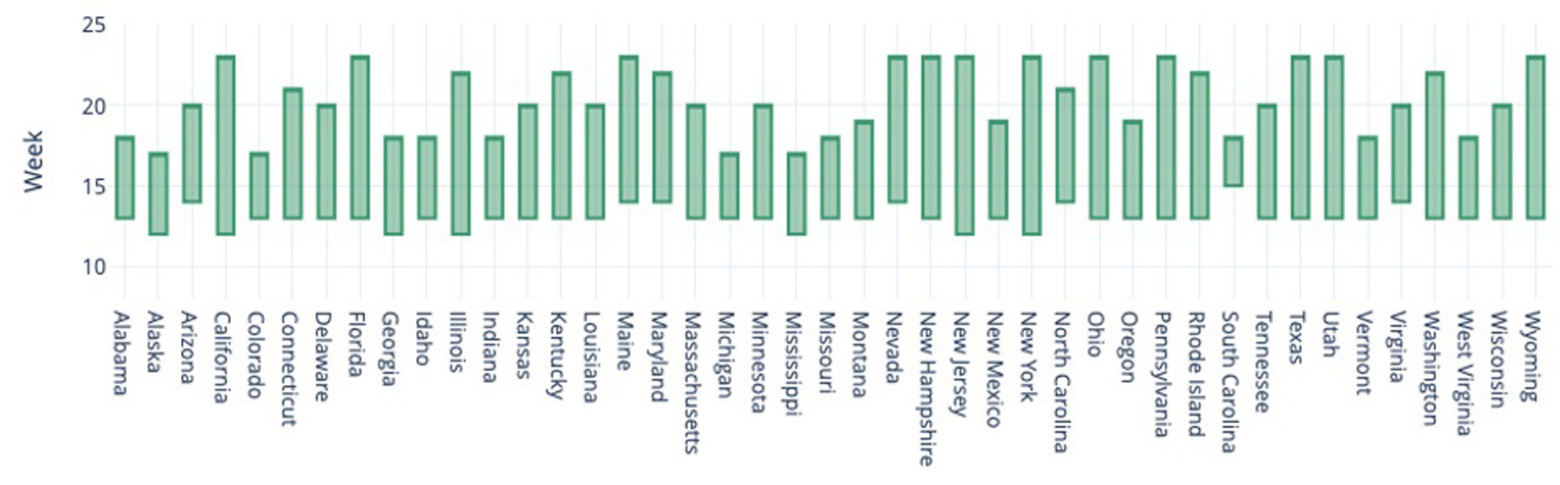
Duration of stay-at-home restrictions in the U.S. States.

Fig 3 compares the length of stay-at-home restrictions in U.S. states. The bars represent the week at least one county within the state enforced a stay-at-home order. New York, California, and New Jersey are among the states with longer stay-at-home orders, at least in one of their counties.

**Fig 4:**
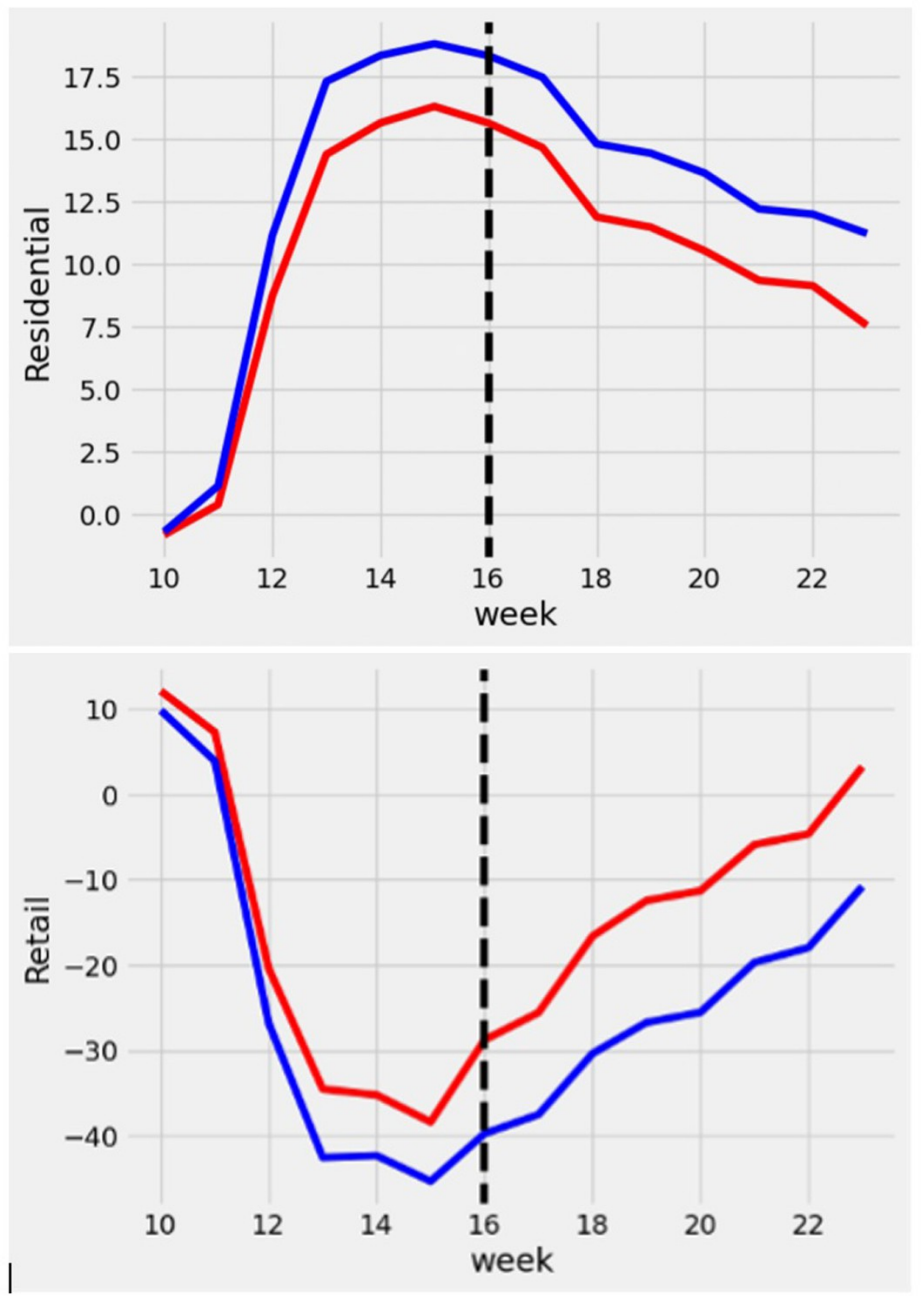
Weekly changes in Retail and Residential indices by Ideological Groups, (Top) Retail Index, (Bottom) Residential Index.

The two plots in Fig 4 visualize the average weekly trend in *Retail* (Top) and *Residential* (Bottom) indices over the period of our study. We separated the counties based on their ideological orientation (i.e., *conservative*). The counties with an above-median score for *conservative* are labeled as Conservative Counties, and counties with a score below the median for *conservative* are labeled as Liberal Counties. According to the plots, Conservative Counties spent more time at retail locations and less time at residential places. Also, we can observe that the retail activity in both Conservative Counties and Liberal Counties started to increase on week 16. We can also observe that both Conservative Counties and Liberal Counties spent less time at residential locations after the OuAA campaign.

Before introducing our econometric model, we present model-free comparisons of *Retail* and *Residential* indices in U.S. counties based on stay-at-home orders, counties’ ideological categories (Conservative County or Liberal County), and the OuAA campaign. According to Table 2, in Liberal counties (Conservative County = 0) that did not have stay-at-home orders in place (stay-at-home order = 0), the value of *Retail* changed from -5.666 to -15.958 (decrease in retail & recreation activity), and the value for *Residential* increased from 4.348 to 11. 869 after the launch of OuAA campaign. For Conservative counties without stay-at-home orders, the value for *Retail* changed from 4.525 to -3.102, and the value for *Residential* changed from 4.525 to 9.072. In Liberal counties with stay-at-home orders in place, the value of *Retail* changed from -43.170 to -30.077 (increase in retail & recreation activity), and the value for *Residential* decreased from 18.084 to 15.235 after the launch of the OuAA campaign. For Conservative counties without stay-at-home orders, the value for *Retail* changed from -37.312 to -17.382, and the value for *Residential* changed from 15.928 to 12.343 after the launch of the OuAA campaign.

**Table 2:** Model-free comparison of U.S. Counties.

Stay-at-home Order	Conservative County	After OuAA Campaign	Retail	Residential
0	0	0	-5.666	4.348
0	0	1	-15.958	11.869
0	1	0	4.525	4.525
0	1	1	-3.102	9.072
1	0	0	-43.170	18.084
1	0	1	-30.077	15.235
1	1	0	-37.312	15.928
1	1	1	-17.382	12.343

## Methods

We use the Difference-in-Difference (DiD) study design to understand the impact of stay-at-home orders on community mobility. The enforcement of stay-at-home orders by counties over time creates a natural experiment setting that allows the comparison of the difference in community mobility before and after enforcing the stay-at-home orders across the counties. We further address the endogeneity of stay-at-home order decisions using a matched sample of counties (a match between counties that enforced an order and counties that never did). To assess the effect of stay-at-home orders on community mobility indices, we employ the following model:

$$\begin{array}{c} y_{ist}=\alpha +\beta_{0}\;stay_{ist}\\ +\beta_{1}\;stay_{ist}\times conservative_{is}+\beta_{2}\;post\_reopen_{t}\times conservative_{is}+\beta_{3}\;stay_{ist}\times post\_reopen_{t}\\ +\beta_{4}\;stay_{ist}\times post\_reopen_{t}\times conservative_{is}\\ +\beta_{5}\;cases_{ist}+\beta_{6}\;deaths_{ist}+\beta_{7}\;unemployment_{ist}+\beta_{8}\;gathering50_{ist}+\beta_{9}\;gathering500_{ist}\\ +\beta_{10}\;dine\_in_{ist}+\beta_{11}\;gym_{ist}\\ +\delta_{t}+\zeta_{is}+\delta_{t}\times \xi_{s}+\epsilon_{ist}, \end{array}$$

where *i* represents the county, *s* represents the state, and *t* represents the week. *y*_*ist*_ is the community mobility index (i.e., *Retail* or *Residential*). We are interested in *β*_0_ through *β*_4_. *β*_0_ is the DiD coefficient, and *β*_1_ through *β*_4_ capture the interaction effects between *stay* and *conservative*, *post_reopen* and *conservative*, and *stay*, *post_reopen*, and *conservative*, respectively. *β*_5_ through *β*_11_ capture the effects of the control variables. *δ*_*t*_ captures time-fixed effects, ζ_*is*_ captures county-fixed effects, and the interaction between time- and state-fixed effects.

## Results

Table 3 reports the results of our DiD analysis. For stay-at-home orders to be effective, we expect a drop in *Retail* and a jump in *Residential*. In models 1 through 3, the coefficient for *stay* is negative and significant. That is, retail & recreation activities decreased in counties with a stay-at-home order after controlling for the number of cases and deaths per 100k, unemployment rate, other types of restrictions, county-fixed effects, time-fixed effects, and the interaction of time- and state-fixed effects. In models 4 through 6, the coefficient for *stay* is positive and significant. This indicates that stay-at-home orders were effective in keeping residents at home. In model 2, the interaction between *stay* and *conservative* is positive and significant. This means that more conservative counties had more retail & recreation activities than liberal counties. In model 5, the coefficient for this interaction is negative and significant. This indicates that the conservative counties spent less time at home during the stay-at-home enforcement. In model 3, the interaction between *post_reopen* and *conservative* is positive and significant, which indicates that the conservative counties had more retail & recreation activities than liberal counties after the OuAA campaign launch. This coefficient is negative and significant in model 6, suggesting that more conservative counties stayed less at home after the OuAA campaign launch than less conservative counties. In model 3, the interaction between *stay*, *post_reopen*, and *conservative* is negative and significant. This means that conservative counties with a stay-at-home order enforced had fewer retail & recreation activities after the OuAA launch than conservative counties that did not. The three-way interaction coefficient in model 6 also suggests that the conservative counties with stay-at-home orders in place spent more time at home after the launch of the OuAA campaign compared to conservative counties that did not.

**Table 3:** The impact of “stay-at-home” orders and “Reopen America” on community mobility indices in U.S. Counties.

	Retail			Residential		
	Model 1	Model 2	Model 3	Model 4	Model 5	Model 6
stay	-3.108*** [0.443]	-8.070*** [0.682]	-7.695*** [0.770]	1.231*** [0.099]	3.237*** [0.194]	4.930*** [0.253]
stay × conservative		7.655*** [0.793]	8.288*** [0.907]		-3.095*** [0.234]	-5.353*** [0.314]
post_reopen × conservative			30.341*** [1.093]			-6.589*** [0.324]
stay × post_reopen			6.179*** [1.103]			-4.275*** [0.312]
stay × post_reopen × conservative			-13.613*** [1.297 ]			6.154*** [0.365]
cases	-0.046*** [0.012]	-0.044*** [0.011]	-0.045*** [0.011]	0.017*** [0.003]	0.017*** [0.003]	0.018*** [0.003]
deaths	-0.527*** [0.161]	-0.438*** [0.159]	-0.264* [0.153]	0.230*** [0.029]	0.194*** [0.031]	0.174*** [0.030]
unemployment	-0.699*** [0.045]	-0.691*** [0.045]	-0.510*** [0.044]	0.145*** [0.009]	0.142*** [0.009]	0.112*** [0.009]
other county restrictions	✓	✓	✓	✓	✓	✓
time fixed effects	✓	✓	✓	✓	✓	✓
county fixed effects	✓	✓	✓	✓	✓	✓
time fixed effects × state fixed effects	✓	✓	✓	✓	✓	✓
Observations	16,443	16,443	16,443	16,443	16,443	16,443
*R* ^2^	0.949	0.950	0.955	0.970	0.970	0.972
F-statistic	155.4***	156.8***	173.6***	264.8***	271.3***	285.4***

To better interpret the results, we can consider a typical conservative county (defined as a county with a score of one standard deviation above the median for variable conservative) and a typical liberal county (defined as a county with a score of one standard deviation below the median for variable conservative) that enforced stay-at-home orders before OuAA’s launch. For these two counties, we can define four phases over time:

- Phase 1: No stay-at-home order and before OuAA
- Phase 2: Stay-at-home order enforced and before OuAA
- Phase 3: Stay-at-home order enforced and after OuAA
- Phase 4: Stay-at-home order expired after OuAA.

**Fig 5:**
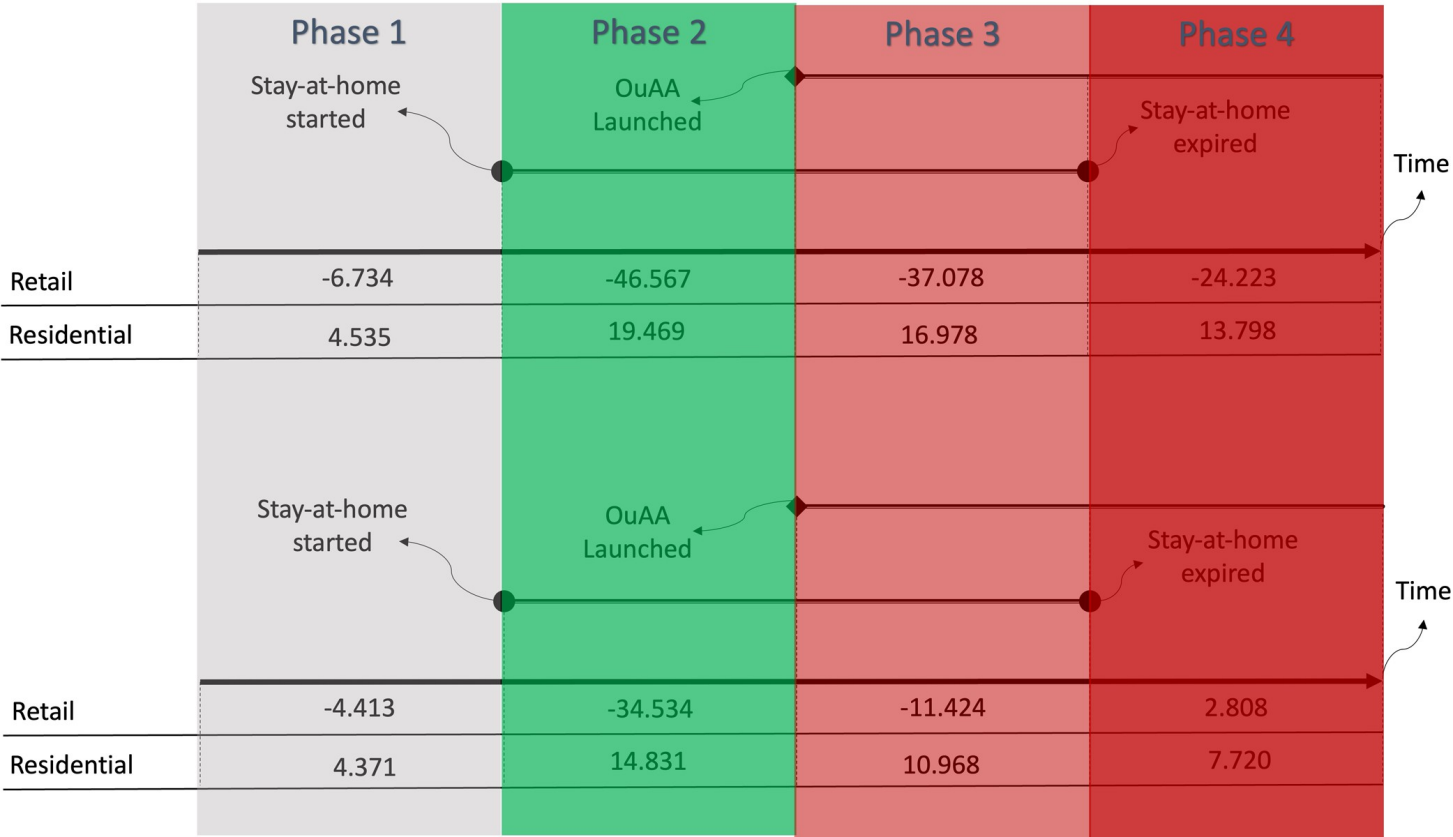
Predicted retail and residential scores for a typical conservative county (Top) and a typical liberal county (Bottom).

We used our model for each of these four phases to obtain the predicted value for Retail and Residential for each of those counties. Fig 5 shows how these values change over each phase for a typical liberal county (Top) and a typical conservative county (Bottom).

For instance, for a liberal county in Fig 5(Top), the predicted value for Retail was -6.734. Even before the county enforced the stay-at-home order, the retail and recreational activities were 6.734 below the baseline (the 5 weeks from January 3^rd^, 2020, to February 6th, 2020). When the county enforced the stay-at-home order (moved to phase 2), the predicted value for Retail decreased further to -46.567. That is, the retail and recreational activities dropped almost 40 points. When OuAA was launched, the predicted value for Retail increased to -37.078 (9.489 points increase). Finally, when the county entered phase 4 (stay-at-home order expired), the value of Retail further increased to -24.223 (12.855 points increase).

For a typical conservative county, retail & recreational activities decreased from -4.413 to -34.534 once the county entered phase 2 (stay-at-home order enforced before OuAA). Once OuAA was launched, the county moved to phase 3, and retail & recreational activities increased to -11.424. When the county entered phase 4 (stay-at-home order lifted after the launch of OuAA), retail activities increased to 2.808.

Overall, Fig 5 supports that stay-at-home orders were, to some extent, effective in decreasing retail and recreational activities in both conservative and liberal counties. However, OuAA was more impactful in opening conservative counties than in liberal counties. In Fig 5, the conservative county’s Retail score increased from -34.534 to -11.424 (23.110 points increase). However, the liberal county’s Retail score increased from -46.567 to -37.078 (9.489 points increase). The difference in the increase in Retail is larger in the conservative county than in the liberal county (23.110 points vs. 9.489). A similar pattern of the impact of OuAA can be observed when we consider the Residential score. That is, the Residential score decreased more for the conservative county than it did for the liberal county after the launch of OuAA.

### Robustness checks

Given the autoregressive nature of our dependent variables (*Retail* and *Residential*), we used the Bayesian information criterion (BIC) to determine the correct lag structure. We started by running our models (Model 3 and Model 6 in Table 3) only with one lag and measuring the BIC. We iteratively added more lags and tracked BIC. We noticed that in both models, the BIC continued to decrease until 8 lags were used in the model. From 7 lags to 8 lags, we noticed an increase in the BIC. Therefore, we decided to use 7 lags. The sign and significance of the coefficients for the interaction terms we hypothesized about in Model 3 and Model 6 remained unchanged:

- In Model 3, the coefficient for the interaction between *conservative* and *post_reopen* was positive and significant (13.919, p-value<0.001), the coefficient for the interaction between *conservative* and *stay* was positive and significant (1.706, p-value<0.05), and the coefficient for the interaction among *conservative* and *stay* and *post_reopen* was negative and significant (-3.451, p-value<0.01).
- In Model 6, the coefficient for the interaction between *conservative* and *post_reopen* was negative and significant (-3.460, p-value<0.001), the coefficient for the interaction between *conservative* and *stay* was negative and significant (-2.273, p-value<0.001), and the coefficient for the interaction among *conservative* and *stay* and *post_reopen* was positive and significant (3.287, p-value<0.001).

Furthermore, given that the decision to enforce stay-at-home orders could be endogenous, we used propensity score matching to find the best match for each county with no stay-at-home enforcement and a similar county that enforced state-at-home orders. We used county-level data about residents’ education, population, deaths, births, domestic and international migration, percent below the federal poverty line, unemployment rate, and median household income to find similar counties (matches for counties that did not have stay-at-home orders). To further strengthen the robustness of our results, instead of the 2016 release of the “County-Level Preference Estimates,” we used the 2022 release in this analysis. This resulted in 660 counties (330 counties without stay-at-home orders and 330 similar counties with stay-at-home orders). We repeated our main DiD analysis using this matched sample instead of the entire data. The results of this analysis are reported in Table 4. According to the results reported in this table, our findings are robust.

**Table 4:** Results based on matched samples.

	Retail	Residential
*Variables*	Model 7	Model 6
Stay	-11.424*** [2.058]	4.684*** [0.559]
stay × conservative	13.580*** [2.152]	-4.845*** [0.585]
post_reopen × conservative	13.908*** [1.496]	-2.116*** [0.407]
stay × post_reopen	2.190 [2.884]	-2.337** [0.784]
stay × post_reopen × conservative	-5.925* [2.662]	1.744* [0.723]
cases	-0.068*** [0.008]	0.020*** [0.002]
deaths	-0.541* [0.215]	0.255*** [0.058]
unemployment	-0.294*** [0.054]	0.158*** [0.015]
other county restrictions	✓	✓
time fixed effects	✓	✓
county fixed effects	✓	✓
time fixed effects × state fixed effects	✓	✓
Observations	6,292	6,292
*R* ^2^	0.936	0.955
F-statistic	138.989***	198.916***

## Discussion, conclusion, and limitations

The immense impact of the COVID-19 pandemic on the lives of billions of people has forced authorities to devise response strategies to contain the damage. We created a panel data set of U.S. counties by collecting data about weekly community mobility scores, weekly COVID-19 new cases, and deaths, the ideological orientation of counties, and state’s COVID-19 response data (stay-at-home and shelter-in-place restrictions timelines) for the period between the first week of March 2020 and the first week of June 2020.

Over time, the enforcement of stay-at-home orders by counties created a natural experiment setting that allows the comparison of differences in community mobility before and after enforcing the stay-at-home orders across the counties. We used the Difference-in-Difference (DiD) study design to understand the impact of stay-at-home orders on community mobility. We further address the endogeneity of stay-at-home order decisions using a matched sample of counties (a match between counties that enforced an order and counties that never did).

Our results indicate that stay-at-home orders effectively decreased commutes to retail stores and increased time spent at home. We also find that conservative counties were more likely to ignore the stay-at-home orders. This finding is aligned with similar studies about partisan behavior in obeying coronavirus restrictions [13]. We further find that the “Opening up America Again” (OuAA) campaign launched by The White House increased retail & recreation activities and decreased time spent at home. We also find that in conservative counties that enforced stay-at-home, the OuAA campaign was less effective compared to conservative counties without stay-at-home orders. These results suggest promising news for local authorities. That is, even when the federal government is reopening the country, the local authorities that enforced stay-at-home restrictions were to some extent effective in decreasing the commute to retail stores and recreational facilities such as gyms and increasing time spent at home. Our findings extend the findings of previous research [2,14].

Our results have profound implications for local and federal government bodies as we show that a misalignment between their policies could result in less effective implementation. Although we used the policies concerning the COVID-19 pandemic, we believe our findings could be generalized to other important contexts, such as views on education, abortion, and gun control. In particular, our findings suggest that the federal government should pay close attention to and not discount the impacts of local government policies that may not be fully aligned with federal policies. Given the current and ever-increasing political divide between local and federal bodies of government, our study calls for more studies on this subject to shed more light on the underlying mechanisms and to offer best practices that would promote the highest utility for citizens.

Finally, it is worth noting that although using publicly available data to conduct this study can be considered one of its strengths, such data sets do not allow us to control for some of the confounding factors which we could have controlled for if we had the opportunity to conduct a controlled experiment. In addition, we rely on Google’s community mobility report, which may have varying degrees of accuracy in different counties (e.g., more accurate for more populated counties and less accurate for less populated counties). Furthermore, we acknowledge that our results are based on aggregated observations and may not fully reflect individual-level behaviors. Given these limitations in our study, we encourage future researchers to supplement our study by conducting analysis in different contexts and using different empirical strategies, such as controlled experiments.

## Supporting information

S1 Data Jupyter notebook (HTML) with results (for replication). (HTML)

S2 Data Data file for replication 1 (aip_counties_ideology_v2022a.csv). (CSV)

S3 Data Data file for replication 2 (res_covid_county_panel_data.csv). (CSV)
