# Supplementary material for: When local governments’ stay-at-home orders meet the white house’s “opening up america again”
Source: PLoS One. 2024 Mar 20;19(3):e0298115. doi: 10.1371/journal.pone.0298115 (PMC10954150; doi:10.1371/journal.pone.0298115)
Supplement: S1 Data — (HTML) [file pone.0298115.s001.html]

covid\_reopen\_plosone


## Impact of States' Stay at-home Orders on Twitter Conversations¶

Reza Mousavi (McIntire School of Commerce, University of Virginia, reza@virginia.edu)

Bin Gu (Questrom School of Business, Boston University, bgu@bu.edu)

In [8]:

```
import pandas as pd
import numpy as np
```

In [9]:

```
dd = pd.read_csv("res_covid_county_panel_data.csv")
dd['fips_code'] = dd.fips_code.astype(str).str.zfill(5)
mrp22 = pd.read_csv("aip_counties_ideology_v2022a.tab", sep = "\t")
mrp22 = mrp22.query('presidential_year==2020')
mrp22['fips_code'] = mrp22.county_fips.astype(str).str.zfill(5)
df = pd.merge(dd,mrp22.drop(columns = 'state'), on = ["fips_code"], how = "left")
```

In [10]:

```
len(dd.fips_code.unique())
```

Out[10]:

```
1211
```

In [11]:

```
df.week.max()
```

Out[11]:

```
23
```

In [12]:

```
df.columns
```

Out[12]:

```
Index(['state', 'fips_code', 'week', 'new_cases_per_100_000',
       'new_deaths_per_100_000', 'unemp_rate', 'retail', 'residential',
       'reopen', 'b_rest1', 'b_rest2', 'b_rest3', 'b_rest4', 'b_rest5',
       'b_rest_sum', 'county_fips_x', 'mrp_ideology_mean', 'Knowledge',
       'Patent', 'University', 'Business', 'mrp_cat', 'sum_stay', 'no_stay',
       'sum_stay_reopen', 'ideo_scale', 'screen_name', 'user_id',
       'favorite_count', 'retweet_count', 'quote_count', 'reply_count',
       'followers_count', 'friends_count', 'listed_count', 'statuses_count',
       'favourites_count', 'month', 'covid', 'weighted_res', 'norm_res',
       'sent', 'subj', 'anger', 'joy', 'fear', 'anticip', 'trust', 'surprise',
       'positive', 'negative', 'sadness', 'disgust', 'ncovid_count',
       'covid_count', 'Areaname', 'county_handle', 'STCOU', 'tweeted',
       'county_fips_y', 'mrp_ideology_se', 'mrp_ideology',
       'irt_ideology_unweighted', 'self_ideology', 'self_ideology_se',
       'irt_ideology_unweighted_sd', 'irt_ideology_unweighted_se',
       'sample_size', 'demshare_pres', 'presidential_year', 'survey_period',
       'county_name', 'population_2020'],
      dtype='object')
```

In [13]:

```
df.mrp_ideology_mean.max()
```

Out[13]:

```
0.842330993
```

In [14]:

```
df['conservative'] = (df.mrp_ideology_mean - df.mrp_ideology_mean.min())/(df.mrp_ideology_mean.max() - df.mrp_ideology_mean.min())
```

In [15]:

```
df[['new_cases_per_100_000',
       'new_deaths_per_100_000', 'unemp_rate', 'retail', 'residential',
       'reopen', 'b_rest1','mrp_ideology_mean','conservative']].describe()
```

Out[15]:

|  | new\_cases\_per\_100\_000 | new\_deaths\_per\_100\_000 | unemp\_rate | retail | residential | reopen | b\_rest1 | mrp\_ideology\_mean | conservative |
| --- | --- | --- | --- | --- | --- | --- | --- | --- | --- |
| count | 16443.000000 | 16443.000000 | 16443.000000 | 16443.000000 | 16443.000000 | 16443.000000 | 16443.000000 | 16443.000000 | 16443.000000 |
| mean | 4.043650 | 0.192503 | 10.158796 | -19.764945 | 11.498361 | 0.567901 | 0.571854 | 0.163060 | 0.649927 |
| std | 9.447560 | 0.504541 | 5.010671 | 19.883501 | 6.455727 | 0.495383 | 0.494825 | 0.290058 | 0.149486 |
| min | -14.670000 | -1.030000 | 1.800000 | -86.571429 | -3.400000 | 0.000000 | 0.000000 | -1.098037 | 0.000000 |
| 25% | 0.244286 | 0.000000 | 5.900000 | -35.285714 | 8.000000 | 0.000000 | 0.000000 | -0.012387 | 0.559507 |
| 50% | 1.445714 | 0.000000 | 10.100000 | -21.571429 | 12.000000 | 1.000000 | 1.000000 | 0.194820 | 0.666295 |
| 75% | 4.338571 | 0.171429 | 13.300000 | -3.619048 | 15.857143 | 1.000000 | 1.000000 | 0.363761 | 0.753361 |
| max | 389.368571 | 8.992857 | 34.300000 | 100.333333 | 31.428571 | 1.000000 | 1.000000 | 0.842331 | 1.000000 |

In [16]:

```
df.b_rest1.value_counts()
```

Out[16]:

```
1    9403
0    7040
Name: b_rest1, dtype: int64
```

In [17]:

```
df.b_rest2.value_counts()
```

Out[17]:

```
1    12943
0     3500
Name: b_rest2, dtype: int64
```

In [18]:

```
df.b_rest3.value_counts()
```

Out[18]:

```
1    13733
0     2710
Name: b_rest3, dtype: int64
```

In [19]:

```
df.b_rest4.value_counts()
```

Out[19]:

```
1    11069
0     5374
Name: b_rest4, dtype: int64
```

In [20]:

```
df.b_rest5.value_counts()
```

Out[20]:

```
1    11145
0     5298
Name: b_rest5, dtype: int64
```

In [21]:

```
df.reopen.value_counts()
```

Out[21]:

```
1    9338
0    7105
Name: reopen, dtype: int64
```

In [22]:

```
df = df.rename(columns={'new_cases_per_100_000': 'cases',
                        'new_deaths_per_100_000': 'deaths',
                        'unemp_rate':'unemployment',
                       'b_rest1':'stay','b_rest2':'gathering50',
                       'b_rest3':'gathering500','b_rest4':'dine_in',
                       'b_rest5':'gym','retail':'Retail','residential':'Residential'})
```

In [23]:

```
df.columns
```

Out[23]:

```
Index(['state', 'fips_code', 'week', 'cases', 'deaths', 'unemployment',
       'Retail', 'Residential', 'reopen', 'stay', 'gathering50',
       'gathering500', 'dine_in', 'gym', 'b_rest_sum', 'county_fips_x',
       'mrp_ideology_mean', 'Knowledge', 'Patent', 'University', 'Business',
       'mrp_cat', 'sum_stay', 'no_stay', 'sum_stay_reopen', 'ideo_scale',
       'screen_name', 'user_id', 'favorite_count', 'retweet_count',
       'quote_count', 'reply_count', 'followers_count', 'friends_count',
       'listed_count', 'statuses_count', 'favourites_count', 'month', 'covid',
       'weighted_res', 'norm_res', 'sent', 'subj', 'anger', 'joy', 'fear',
       'anticip', 'trust', 'surprise', 'positive', 'negative', 'sadness',
       'disgust', 'ncovid_count', 'covid_count', 'Areaname', 'county_handle',
       'STCOU', 'tweeted', 'county_fips_y', 'mrp_ideology_se', 'mrp_ideology',
       'irt_ideology_unweighted', 'self_ideology', 'self_ideology_se',
       'irt_ideology_unweighted_sd', 'irt_ideology_unweighted_se',
       'sample_size', 'demshare_pres', 'presidential_year', 'survey_period',
       'county_name', 'population_2020', 'conservative'],
      dtype='object')
```

In [24]:

```
temp = df[['Retail', 'Residential','cases', 'deaths', 'unemployment',
        'reopen', 'stay', 'gathering50',
       'gathering500', 'dine_in', 'gym','conservative']]
```

In [25]:

```
import matplotlib.pyplot as plt
import numpy as np


plt.style.use('fivethirtyeight')

# import pandas.rpy.common as com
import seaborn as sns
%matplotlib inline


# calculate the correlation matrix
corr = temp.corr()

# plot the heatmap
a4_dims = (11.7, 8.27)
fig, ax = plt.subplots(figsize=a4_dims)
sns.heatmap(corr,cmap="Blues", annot=True,
        xticklabels=corr.columns,
        yticklabels=corr.columns)
```

Out[25]:

```
<AxesSubplot:>
```

In [26]:

```
gdf = df.groupby(['fips_code'],as_index = False).agg('mean')
```

In [27]:

```
gdf.columns
```

Out[27]:

```
Index(['fips_code', 'week', 'cases', 'deaths', 'unemployment', 'Retail',
       'Residential', 'reopen', 'stay', 'gathering50', 'gathering500',
       'dine_in', 'gym', 'b_rest_sum', 'county_fips_x', 'mrp_ideology_mean',
       'Knowledge', 'Patent', 'University', 'Business', 'mrp_cat', 'sum_stay',
       'no_stay', 'sum_stay_reopen', 'ideo_scale', 'user_id', 'favorite_count',
       'retweet_count', 'quote_count', 'reply_count', 'followers_count',
       'friends_count', 'listed_count', 'statuses_count', 'favourites_count',
       'month', 'covid', 'weighted_res', 'norm_res', 'sent', 'subj', 'anger',
       'joy', 'fear', 'anticip', 'trust', 'surprise', 'positive', 'negative',
       'sadness', 'disgust', 'ncovid_count', 'covid_count', 'STCOU', 'tweeted',
       'county_fips_y', 'mrp_ideology_se', 'mrp_ideology',
       'irt_ideology_unweighted', 'self_ideology', 'self_ideology_se',
       'irt_ideology_unweighted_sd', 'irt_ideology_unweighted_se',
       'sample_size', 'demshare_pres', 'presidential_year', 'population_2020',
       'conservative'],
      dtype='object')
```

In [28]:

```
from urllib.request import urlopen
import json
with urlopen('https://raw.githubusercontent.com/plotly/datasets/master/geojson-counties-fips.json') as response:
    counties = json.load(response)

import plotly.express as px

fig = px.choropleth(gdf, geojson=counties, locations='fips_code', 
                    color='stay',
                           color_continuous_scale="Viridis_r",
#                            range_color=(0, 12),
                           scope="usa",
                           labels={'plot':'plot'}
                          )
fig.update_layout(margin={"r":0,"t":0,"l":0,"b":0})
fig.show()
```

In [21]:

```
df.week.max()
```

Out[21]:

```
23
```

In [29]:

```
df['Conservative'] = np.where(df.conservative<=df.conservative.median(),0,1)
```

In [30]:

```
gdf = df.groupby(["Conservative","week"], as_index = False).agg('mean')
gdf.head()
```

Out[30]:

|  | Conservative | week | cases | deaths | unemployment | Retail | Residential | reopen | stay | gathering50 | ... | irt\_ideology\_unweighted | self\_ideology | self\_ideology\_se | irt\_ideology\_unweighted\_sd | irt\_ideology\_unweighted\_se | sample\_size | demshare\_pres | presidential\_year | population\_2020 | conservative |
| --- | --- | --- | --- | --- | --- | --- | --- | --- | --- | --- | --- | --- | --- | --- | --- | --- | --- | --- | --- | --- | --- |
| 0 | 0 | 10 | 0.014300 | 0.000300 | 4.579242 | 9.809555 | -0.675343 | 0.0 | 0.000000 | 0.000000 | ... | -0.020163 | 3.009192 | 0.072750 | 0.975218 | 0.062360 | 714.029654 | 0.512877 | 2020.0 | 358935.813839 | 0.534035 |
| 1 | 0 | 11 | 0.086072 | 0.001252 | 4.579242 | 3.878324 | 1.156864 | 0.0 | 0.000000 | 0.039539 | ... | -0.020163 | 3.009192 | 0.072750 | 0.975218 | 0.062360 | 714.029654 | 0.512877 | 2020.0 | 358935.813839 | 0.534035 |
| 2 | 0 | 12 | 0.779755 | 0.010640 | 4.579242 | -26.827253 | 11.154570 | 0.0 | 0.220758 | 0.583196 | ... | -0.020163 | 3.009192 | 0.072750 | 0.975218 | 0.062360 | 714.029654 | 0.512877 | 2020.0 | 358935.813839 | 0.534035 |
| 3 | 0 | 13 | 3.001016 | 0.054578 | 4.596791 | -42.537645 | 17.319293 | 0.0 | 0.704392 | 0.996622 | ... | -0.024526 | 3.004187 | 0.071262 | 0.975054 | 0.061045 | 730.222973 | 0.515963 | 2020.0 | 367077.594595 | 0.532805 |
| 4 | 0 | 14 | 6.212334 | 0.190121 | 11.616728 | -42.329139 | 18.358586 | 0.0 | 0.979275 | 1.000000 | ... | -0.027926 | 2.998708 | 0.069537 | 0.976139 | 0.059787 | 745.081174 | 0.517525 | 2020.0 | 374473.179620 | 0.532079 |

5 rows × 68 columns

In [24]:

```
fig, ax = plt.subplots()
sns.lineplot(data=gdf.query("Conservative==1"), x="week", y="Retail", color = "red")
sns.lineplot(data=gdf.query("Conservative==0"), x="week", y="Retail", color = "blue")

ax.axvline(x = 16,    # Line on x = 2
           ymin = -400, # Bottom of the plot
           ymax = 10,
           color = "black", linestyle = "dashed") # Top of the plot
```

Out[24]:

```
<matplotlib.lines.Line2D at 0x17d50bd30>
```

In [25]:

```
fig, ax = plt.subplots()
sns.lineplot(data=gdf.query("Conservative==1"), x="week", y="Residential", color = "red")
sns.lineplot(data=gdf.query("Conservative==0"), x="week", y="Residential", color = "blue")

ax.axvline(x = 16,    # Line on x = 2
           ymin = -400, # Bottom of the plot
           ymax = 10,
           color = "black", linestyle = "dashed") # Top of the plot
```

Out[25]:

```
<matplotlib.lines.Line2D at 0x285fdb4f0>
```

In [26]:

```
df.columns
```

Out[26]:

```
Index(['state', 'fips_code', 'week', 'cases', 'deaths', 'unemployment',
       'Retail', 'Residential', 'reopen', 'stay', 'gathering50',
       'gathering500', 'dine_in', 'gym', 'b_rest_sum', 'county_fips_x',
       'mrp_ideology_mean', 'Knowledge', 'Patent', 'University', 'Business',
       'mrp_cat', 'sum_stay', 'no_stay', 'sum_stay_reopen', 'ideo_scale',
       'screen_name', 'user_id', 'favorite_count', 'retweet_count',
       'quote_count', 'reply_count', 'followers_count', 'friends_count',
       'listed_count', 'statuses_count', 'favourites_count', 'month', 'covid',
       'weighted_res', 'norm_res', 'sent', 'subj', 'anger', 'joy', 'fear',
       'anticip', 'trust', 'surprise', 'positive', 'negative', 'sadness',
       'disgust', 'ncovid_count', 'covid_count', 'Areaname', 'county_handle',
       'STCOU', 'tweeted', 'county_fips_y', 'mrp_ideology_se', 'mrp_ideology',
       'irt_ideology_unweighted', 'self_ideology', 'self_ideology_se',
       'irt_ideology_unweighted_sd', 'irt_ideology_unweighted_se',
       'sample_size', 'demshare_pres', 'presidential_year', 'survey_period',
       'county_name', 'population_2020', 'conservative', 'Conservative'],
      dtype='object')
```

In [27]:

```
df.groupby(['stay','Conservative','reopen'],as_index = False).agg('mean')[['stay','Conservative',
                                                                           'reopen','Retail',
                                                                           'Residential']]
```

Out[27]:

|  | stay | Conservative | reopen | Retail | Residential |
| --- | --- | --- | --- | --- | --- |
| 0 | 0 | 0 | 0 | -5.666733 | 4.348214 |
| 1 | 0 | 0 | 1 | -15.958111 | 11.869595 |
| 2 | 0 | 1 | 0 | -5.309894 | 4.525670 |
| 3 | 0 | 1 | 1 | -3.102908 | 9.072051 |
| 4 | 1 | 0 | 0 | -43.170042 | 18.084578 |
| 5 | 1 | 0 | 1 | -30.077958 | 15.235856 |
| 6 | 1 | 1 | 0 | -37.312839 | 15.928571 |
| 7 | 1 | 1 | 1 | -17.382869 | 12.343112 |

In [28]:

```
import statsmodels.formula.api as smf

reg = smf.ols('Retail ~ cases + deaths + unemployment + C(week) +\
C(week) * C(state) + C(fips_code) + b_rest_sum + stay',
              data = df).fit()

# print(reg.summary())
pd.DataFrame({'coef' : reg.params[-7:] , 'std err': reg.HC0_se[-7:] , 'tval' : reg.tvalues[-7:] , 'pval' : reg.pvalues[-7:]})
```

Out[28]:

|  | coef | std err | tval | pval |
| --- | --- | --- | --- | --- |
| C(week)[T.22]:C(state)[T.Wyoming] | 8.498948 | 2.894875 | 3.160205 | 1.579788e-03 |
| C(week)[T.23]:C(state)[T.Wyoming] | 8.182918 | 3.309282 | 3.043121 | 2.345522e-03 |
| cases | -0.046518 | 0.011591 | -8.383157 | 5.616990e-17 |
| deaths | -0.527976 | 0.161891 | -4.564637 | 5.044247e-06 |
| unemployment | -0.699406 | 0.045258 | -22.535236 | 1.400448e-110 |
| b\_rest\_sum | -7.895537 | 1.938421 | -5.565876 | 2.654104e-08 |
| stay | -3.108402 | 0.443649 | -6.431774 | 1.300300e-10 |

In [29]:

```
df.shape
```

Out[29]:

```
(16443, 75)
```

In [30]:

```
print(reg.summary())
```

```
                            OLS Regression Results                            
==============================================================================
Dep. Variable:                 Retail   R-squared:                       0.949
Model:                            OLS   Adj. R-squared:                  0.943
Method:                 Least Squares   F-statistic:                     155.4
Date:                Wed, 12 Apr 2023   Prob (F-statistic):               0.00
Time:                        17:36:22   Log-Likelihood:                -47948.
No. Observations:               16443   AIC:                         9.945e+04
Df Residuals:                   14668   BIC:                         1.131e+05
Df Model:                        1774                                         
Covariance Type:            nonrobust                                         
============================================================================================================
                                               coef    std err          t      P>|t|      [0.025      0.975]
------------------------------------------------------------------------------------------------------------
Intercept                                   15.2107      1.501     10.133      0.000      12.268      18.153
C(week)[T.11]                                4.4983      1.847      2.435      0.015       0.878       8.119
C(week)[T.12]                              -22.1281      1.847    -11.980      0.000     -25.748     -18.508
C(week)[T.13]                              -33.3790      1.847    -18.074      0.000     -36.999     -29.759
C(week)[T.14]                              -27.9285      1.910    -14.622      0.000     -31.672     -24.185
C(week)[T.15]                              -31.5364      1.935    -16.295      0.000     -35.330     -27.743
C(week)[T.16]                              -18.6965      1.935     -9.661      0.000     -22.490     -14.903
C(week)[T.17]                              -15.4330      1.929     -8.001      0.000     -19.214     -11.652
C(week)[T.18]                               -8.0716      1.914     -4.217      0.000     -11.823      -4.320
C(week)[T.19]                               -8.2612      1.854     -4.455      0.000     -11.896      -4.627
C(week)[T.20]                               -4.6164      1.854     -2.490      0.013      -8.251      -0.982
C(week)[T.21]                               -1.4416      1.854     -0.777      0.437      -5.076       2.193
C(week)[T.22]                               -4.4584      1.855     -2.404      0.016      -8.094      -0.823
C(week)[T.23]                                4.7235      1.851      2.552      0.011       1.096       8.351
C(state)[T.Alaska]                          -0.9194      2.243     -0.410      0.682      -5.316       3.477
C(state)[T.Arizona]                          1.9964      1.860      1.073      0.283      -1.650       5.643
C(state)[T.California]                      -5.2369      1.626     -3.220      0.001      -8.425      -2.049
C(state)[T.Colorado]                        -2.4317      1.774     -1.371      0.170      -5.909       1.045
C(state)[T.Connecticut]                     -4.6355      1.937     -2.393      0.017      -8.432      -0.839
C(state)[T.Delaware]                        -1.4953      2.338     -0.639      0.523      -6.079       3.088
C(state)[T.Florida]                         -4.2618      1.614     -2.640      0.008      -7.426      -1.098
C(state)[T.Georgia]                         -2.8234      1.608     -1.756      0.079      -5.975       0.328
C(state)[T.Idaho]                           -2.3831      1.873     -1.273      0.203      -6.054       1.288
C(state)[T.Illinois]                         1.7239      1.613      1.069      0.285      -1.437       4.885
C(state)[T.Indiana]                          1.0993      1.612      0.682      0.495      -2.061       4.260
C(state)[T.Kansas]                           1.2394      1.726      0.718      0.473      -2.144       4.622
C(state)[T.Kentucky]                         0.9528      1.685      0.566      0.572      -2.350       4.255
C(state)[T.Louisiana]                        2.0144      1.695      1.188      0.235      -1.309       5.337
C(state)[T.Maine]                           -1.1500      1.851     -0.621      0.535      -4.779       2.479
C(state)[T.Maryland]                        -4.8177      1.749     -2.755      0.006      -8.246      -1.390
C(state)[T.Massachusetts]                   -5.7604      1.873     -3.076      0.002      -9.431      -2.089
C(state)[T.Michigan]                         1.2251      1.630      0.752      0.452      -1.970       4.420
C(state)[T.Minnesota]                       -1.4795      1.653     -0.895      0.371      -4.721       1.761
C(state)[T.Mississippi]                      2.4041      1.785      1.347      0.178      -1.095       5.903
C(state)[T.Missouri]                         5.1700      1.654      3.126      0.002       1.929       8.412
C(state)[T.Montana]                         -2.0517      1.998     -1.027      0.304      -5.968       1.864
C(state)[T.Nevada]                          -4.2523      2.046     -2.078      0.038      -8.264      -0.241
C(state)[T.New Hampshire]                   -5.1809      1.926     -2.690      0.007      -8.956      -1.406
C(state)[T.New Jersey]                      -4.7850      1.739     -2.751      0.006      -8.194      -1.376
C(state)[T.New Mexico]                       0.5917      1.854      0.319      0.750      -3.043       4.226
C(state)[T.New York]                        -0.6278      1.609     -0.390      0.696      -3.781       2.525
C(state)[T.North Carolina]                  -1.4894      1.583     -0.941      0.347      -4.593       1.614
C(state)[T.Ohio]                             1.7731      1.582      1.121      0.262      -1.327       4.874
C(state)[T.Oregon]                          -2.4515      1.749     -1.401      0.161      -5.880       0.977
C(state)[T.Pennsylvania]                     0.4940      1.607      0.307      0.758      -2.655       3.643
C(state)[T.Rhode Island]                    -2.9552      2.043     -1.447      0.148      -6.960       1.049
C(state)[T.South Carolina]                  -3.5015      1.686     -2.077      0.038      -6.807      -0.196
C(state)[T.Tennessee]                        0.8435      1.617      0.522      0.602      -2.326       4.013
C(state)[T.Texas]                           -0.6131      1.564     -0.392      0.695      -3.679       2.453
C(state)[T.Utah]                            -1.8065      2.482     -0.728      0.467      -6.671       3.058
C(state)[T.Vermont]                         -4.4254      2.161     -2.047      0.041      -8.662      -0.189
C(state)[T.Virginia]                        -4.2083      1.671     -2.519      0.012      -7.483      -0.934
C(state)[T.Washington]                      -2.8626      1.687     -1.697      0.090      -6.170       0.445
C(state)[T.West Virginia]                   -0.4312      1.875     -0.230      0.818      -4.106       3.243
C(state)[T.Wisconsin]                       -2.0394      1.625     -1.255      0.209      -5.224       1.145
C(state)[T.Wyoming]                         -0.6630      1.998     -0.332      0.740      -4.580       3.254
C(fips_code)[T.01003]                       -4.0354      1.789     -2.255      0.024      -7.542      -0.528
C(fips_code)[T.01009]                        0.4964      1.789      0.278      0.781      -3.010       4.003
C(fips_code)[T.01015]                       -0.2190      1.792     -0.122      0.903      -3.732       3.294
C(fips_code)[T.01017]                        7.4901      1.867      4.013      0.000       3.831      11.149
C(fips_code)[T.01021]                        1.1160      1.788      0.624      0.533      -2.389       4.622
C(fips_code)[T.01031]                        4.1592      1.789      2.325      0.020       0.653       7.665
C(fips_code)[T.01033]                        1.3777      1.791      0.769      0.442      -2.132       4.888
C(fips_code)[T.01039]                        4.3107      1.910      2.257      0.024       0.567       8.055
C(fips_code)[T.01043]                       -2.8446      1.788     -1.591      0.112      -6.350       0.661
C(fips_code)[T.01045]                       -5.6251      1.789     -3.145      0.002      -9.131      -2.119
C(fips_code)[T.01049]                       -1.0320      1.788     -0.577      0.564      -4.537       2.473
C(fips_code)[T.01051]                       -0.8345      1.788     -0.467      0.641      -4.340       2.671
C(fips_code)[T.01055]                       -2.2417      1.793     -1.250      0.211      -5.757       1.273
C(fips_code)[T.01069]                       -6.7887      1.788     -3.796      0.000     -10.294      -3.283
C(fips_code)[T.01071]                        0.0377      1.789      0.021      0.983      -3.468       3.544
C(fips_code)[T.01073]                      -14.7318      1.789     -8.235      0.000     -18.238     -11.225
C(fips_code)[T.01077]                       -2.8221      1.789     -1.577      0.115      -6.329       0.685
C(fips_code)[T.01081]                      -14.3127      1.788     -8.003      0.000     -17.818     -10.807
C(fips_code)[T.01083]                       -0.5207      1.788     -0.291      0.771      -4.026       2.985
C(fips_code)[T.01089]                      -14.9048      1.788     -8.334      0.000     -18.410     -11.399
C(fips_code)[T.01095]                        0.4420      1.789      0.247      0.805      -3.064       3.948
C(fips_code)[T.01097]                       -1.1777      1.791     -0.658      0.511      -4.688       2.333
C(fips_code)[T.01101]                       -8.7041      1.790     -4.861      0.000     -12.214      -5.195
C(fips_code)[T.01103]                       -4.0596      1.788     -2.270      0.023      -7.565      -0.554
C(fips_code)[T.01113]                        2.1996      1.788      1.230      0.219      -1.306       5.705
C(fips_code)[T.01115]                        2.0714      1.788      1.158      0.247      -1.434       5.577
C(fips_code)[T.01117]                      -11.3796      1.789     -6.361      0.000     -14.886      -7.873
C(fips_code)[T.01121]                        6.3768      1.793      3.557      0.000       2.863       9.891
C(fips_code)[T.01123]                        5.1444      1.798      2.861      0.004       1.619       8.669
C(fips_code)[T.01125]                       -7.6302      1.790     -4.262      0.000     -11.139      -4.121
C(fips_code)[T.01127]                        1.8749      1.789      1.048      0.295      -1.631       5.381
C(fips_code)[T.02020]                       -9.5349      1.230     -7.750      0.000     -11.947      -7.123
C(fips_code)[T.02090]                       -5.1630      1.231     -4.195      0.000      -7.575      -2.751
C(fips_code)[T.02122]                        6.1675      1.233      5.003      0.000       3.751       8.584
C(fips_code)[T.02170]                        7.6111      1.231      6.182      0.000       5.198      10.024
C(fips_code)[T.04001]                       -4.6215      1.231     -3.753      0.000      -7.035      -2.208
C(fips_code)[T.04003]                       -4.6411      1.225     -3.787      0.000      -7.043      -2.239
C(fips_code)[T.04005]                       -3.1138      1.225     -2.541      0.011      -5.516      -0.712
C(fips_code)[T.04007]                       21.1924      1.225     17.300      0.000      18.791      23.593
C(fips_code)[T.04009]                        6.8028      1.229      5.535      0.000       4.394       9.212
C(fips_code)[T.04013]                       -8.2065      1.225     -6.700      0.000     -10.607      -5.806
C(fips_code)[T.04015]                        6.2554      1.227      5.099      0.000       3.851       8.660
C(fips_code)[T.04017]                       18.1804      1.226     14.827      0.000      15.777      20.584
C(fips_code)[T.04019]                       -9.1262      1.224     -7.454      0.000     -11.526      -6.726
C(fips_code)[T.04021]                        2.6011      1.225      2.124      0.034       0.201       5.002
C(fips_code)[T.04023]                      -16.4578      1.226    -13.429      0.000     -18.860     -14.056
C(fips_code)[T.04025]                       -0.8694      1.224     -0.710      0.478      -3.269       1.530
C(fips_code)[T.04027]                       -5.9993      1.256     -4.778      0.000      -8.460      -3.538
C(fips_code)[T.06001]                      -15.7968      1.254    -12.595      0.000     -18.255     -13.338
C(fips_code)[T.06007]                        4.8990      1.253      3.911      0.000       2.443       7.355
C(fips_code)[T.06009]                       -2.3789      1.657     -1.436      0.151      -5.627       0.869
C(fips_code)[T.06013]                       -8.8569      1.254     -7.063      0.000     -11.315      -6.399
C(fips_code)[T.06017]                       -8.2958      1.253     -6.622      0.000     -10.751      -5.840
C(fips_code)[T.06019]                        5.2485      1.254      4.185      0.000       2.791       7.706
C(fips_code)[T.06021]                       14.1153      2.703      5.223      0.000       8.818      19.413
C(fips_code)[T.06023]                       -5.2592      1.254     -4.193      0.000      -7.718      -2.800
C(fips_code)[T.06025]                        3.4574      1.320      2.620      0.009       0.870       6.044
C(fips_code)[T.06029]                       10.5289      1.258      8.368      0.000       8.063      12.995
C(fips_code)[T.06031]                       13.6475      1.256     10.865      0.000      11.185      16.110
C(fips_code)[T.06033]                       12.9921      1.253     10.371      0.000      10.537      15.448
C(fips_code)[T.06037]                       -4.4591      1.259     -3.543      0.000      -6.926      -1.992
C(fips_code)[T.06039]                        5.7519      1.254      4.588      0.000       3.295       8.209
C(fips_code)[T.06041]                      -18.0963      1.260    -14.366      0.000     -20.565     -15.627
C(fips_code)[T.06045]                        3.2524      1.253      2.595      0.009       0.796       5.709
C(fips_code)[T.06047]                       14.0539      1.257     11.178      0.000      11.589      16.518
C(fips_code)[T.06053]                       -0.2227      1.258     -0.177      0.859      -2.689       2.244
C(fips_code)[T.06055]                       -4.4490      1.253     -3.551      0.000      -6.905      -1.993
C(fips_code)[T.06057]                       -7.7488      1.253     -6.184      0.000     -10.205      -5.293
C(fips_code)[T.06059]                       -9.3388      1.254     -7.450      0.000     -11.796      -6.882
C(fips_code)[T.06061]                       -6.8634      1.255     -5.468      0.000      -9.324      -4.403
C(fips_code)[T.06065]                       -1.6717      1.253     -1.335      0.182      -4.127       0.784
C(fips_code)[T.06067]                       -2.1566      1.253     -1.721      0.085      -4.613       0.300
C(fips_code)[T.06069]                        7.5886      1.253      6.054      0.000       5.132      10.046
C(fips_code)[T.06071]                       -0.7008      1.253     -0.559      0.576      -3.157       1.756
C(fips_code)[T.06073]                       -8.3750      1.253     -6.685      0.000     -10.831      -5.919
C(fips_code)[T.06075]                      -30.8307      1.260    -24.470      0.000     -33.300     -28.361
C(fips_code)[T.06077]                        6.8983      1.254      5.502      0.000       4.441       9.356
C(fips_code)[T.06079]                       -7.8048      1.254     -6.222      0.000     -10.264      -5.346
C(fips_code)[T.06081]                      -17.3379      1.258    -13.778      0.000     -19.804     -14.871
C(fips_code)[T.06083]                       -5.8412      1.254     -4.659      0.000      -8.298      -3.384
C(fips_code)[T.06085]                      -20.9233      1.258    -16.631      0.000     -23.389     -18.457
C(fips_code)[T.06087]                       -7.7762      1.257     -6.185      0.000     -10.240      -5.312
C(fips_code)[T.06089]                        8.1899      1.253      6.537      0.000       5.734      10.646
C(fips_code)[T.06093]                       19.2744      1.253     15.385      0.000      16.819      21.730
C(fips_code)[T.06095]                       -0.9425      1.253     -0.752      0.452      -3.398       1.513
C(fips_code)[T.06097]                       -8.6601      1.254     -6.905      0.000     -11.119      -6.202
C(fips_code)[T.06099]                        6.3964      1.254      5.103      0.000       3.939       8.853
C(fips_code)[T.06101]                       17.2546      1.256     13.742      0.000      14.793      19.716
C(fips_code)[T.06103]                       11.6455      1.253      9.295      0.000       9.190      14.101
C(fips_code)[T.06107]                       10.3543      1.262      8.206      0.000       7.881      12.827
C(fips_code)[T.06109]                        7.8159      1.253      6.238      0.000       5.360      10.272
C(fips_code)[T.06111]                       -5.2635      1.254     -4.199      0.000      -7.721      -2.806
C(fips_code)[T.06113]                       -4.9792      1.256     -3.964      0.000      -7.442      -2.517
C(fips_code)[T.06115]                       26.4275      1.253     21.093      0.000      23.972      28.883
C(fips_code)[T.08001]                        1.7638      1.234      1.429      0.153      -0.655       4.183
C(fips_code)[T.08005]                       -3.9735      1.234     -3.220      0.001      -6.393      -1.554
C(fips_code)[T.08013]                      -13.9002      1.235    -11.258      0.000     -16.320     -11.480
C(fips_code)[T.08014]                       -9.5617      1.234     -7.748      0.000     -11.981      -7.143
C(fips_code)[T.08031]                      -14.6183      1.235    -11.841      0.000     -17.038     -12.198
C(fips_code)[T.08035]                       -6.0315      1.235     -4.885      0.000      -8.452      -3.611
C(fips_code)[T.08037]                      -19.0524      1.397    -13.637      0.000     -21.791     -16.314
C(fips_code)[T.08041]                        3.1400      1.234      2.545      0.011       0.721       5.559
C(fips_code)[T.08043]                       19.1969      1.235     15.544      0.000      16.776      21.618
C(fips_code)[T.08045]                        4.3917      1.234      3.558      0.000       1.972       6.811
C(fips_code)[T.08059]                       -3.7977      1.234     -3.078      0.002      -6.216      -1.379
C(fips_code)[T.08067]                        0.0200      1.234      0.016      0.987      -2.400       2.439
C(fips_code)[T.08069]                       -4.4205      1.234     -3.581      0.000      -6.840      -2.001
C(fips_code)[T.08077]                       12.4456      1.234     10.083      0.000      10.026      14.865
C(fips_code)[T.08085]                       11.4409      1.234      9.272      0.000       9.022      13.860
C(fips_code)[T.08087]                        6.8715      1.341      5.126      0.000       4.244       9.499
C(fips_code)[T.08101]                        9.2097      1.234      7.463      0.000       6.791      11.629
C(fips_code)[T.08123]                        4.4441      1.234      3.600      0.000       2.024       6.864
C(fips_code)[T.09001]                       -6.3003      0.912     -6.911      0.000      -8.087      -4.513
C(fips_code)[T.09003]                       -4.4431      1.205     -3.686      0.000      -6.806      -2.081
C(fips_code)[T.09005]                        5.4947      1.203      4.566      0.000       3.136       7.854
C(fips_code)[T.09007]                       -0.9321      1.204     -0.774      0.439      -3.292       1.427
C(fips_code)[T.09009]                       -0.5220      1.204     -0.433      0.665      -2.882       1.838
C(fips_code)[T.09011]                       -4.4064      1.208     -3.646      0.000      -6.775      -2.038
C(fips_code)[T.09013]                       -5.8544      1.205     -4.859      0.000      -8.216      -3.493
C(fips_code)[T.09015]                       12.3282      1.206     10.222      0.000       9.964      14.692
C(fips_code)[T.10001]                        2.1826      1.294      1.686      0.092      -0.354       4.720
C(fips_code)[T.10003]                       -7.4183      1.294     -5.734      0.000      -9.954      -4.882
C(fips_code)[T.10005]                        3.7404      1.295      2.889      0.004       1.203       6.278
C(fips_code)[T.12001]                      -10.7809      1.255     -8.589      0.000     -13.241      -8.321
C(fips_code)[T.12005]                       10.9340      1.252      8.732      0.000       8.480      13.388
C(fips_code)[T.12009]                       -0.8218      1.252     -0.656      0.512      -3.276       1.632
C(fips_code)[T.12011]                       -8.7040      1.254     -6.942      0.000     -11.162      -6.246
C(fips_code)[T.12015]                       -7.1261      1.253     -5.687      0.000      -9.582      -4.670
C(fips_code)[T.12017]                        3.3530      1.254      2.675      0.007       0.896       5.810
C(fips_code)[T.12019]                        5.7928      1.254      4.621      0.000       3.335       8.250
C(fips_code)[T.12021]                      -13.1891      1.252    -10.533      0.000     -15.643     -10.735
C(fips_code)[T.12023]                        6.2009      1.254      4.945      0.000       3.743       8.659
C(fips_code)[T.12027]                       -0.9960      1.257     -0.792      0.428      -3.460       1.468
C(fips_code)[T.12031]                        2.0589      1.252      1.644      0.100      -0.396       4.514
C(fips_code)[T.12033]                        5.7881      1.252      4.623      0.000       3.334       8.242
C(fips_code)[T.12035]                        2.1112      1.253      1.685      0.092      -0.345       4.568
C(fips_code)[T.12039]                        9.7378      1.254      7.763      0.000       7.279      12.197
C(fips_code)[T.12051]                        5.6747      1.254      4.525      0.000       3.217       8.133
C(fips_code)[T.12053]                        6.4263      1.253      5.128      0.000       3.970       8.883
C(fips_code)[T.12055]                       -4.9701      1.252     -3.969      0.000      -7.424      -2.516
C(fips_code)[T.12057]                       -4.8411      1.252     -3.866      0.000      -7.296      -2.386
C(fips_code)[T.12061]                       -4.8677      1.252     -3.887      0.000      -7.322      -2.413
C(fips_code)[T.12063]                       12.0690      1.255      9.616      0.000       9.609      14.529
C(fips_code)[T.12069]                        2.1370      1.256      1.701      0.089      -0.326       4.600
C(fips_code)[T.12071]                       -9.1942      1.252     -7.341      0.000     -11.649      -6.739
C(fips_code)[T.12073]                       -5.8715      1.255     -4.678      0.000      -8.331      -3.412
C(fips_code)[T.12075]                       14.4354      1.253     11.522      0.000      11.980      16.891
C(fips_code)[T.12081]                       -5.6562      1.252     -4.517      0.000      -8.111      -3.202
C(fips_code)[T.12083]                        3.1251      1.252      2.496      0.013       0.671       5.579
C(fips_code)[T.12085]                       -7.5446      1.252     -6.024      0.000      -9.999      -5.090
C(fips_code)[T.12086]                      -13.0537      1.253    -10.421      0.000     -15.509     -10.598
C(fips_code)[T.12087]                      -25.8217      1.255    -20.581      0.000     -28.281     -23.362
C(fips_code)[T.12089]                        5.3861      1.252      4.301      0.000       2.931       7.841
C(fips_code)[T.12091]                        7.1640      1.252      5.720      0.000       4.709       9.619
C(fips_code)[T.12093]                       -0.5582      1.256     -0.444      0.657      -3.020       1.903
C(fips_code)[T.12095]                      -16.4002      1.262    -12.995      0.000     -18.874     -13.926
C(fips_code)[T.12097]                       -4.8073      1.285     -3.742      0.000      -7.325      -2.289
C(fips_code)[T.12099]                       -8.6035      1.253     -6.868      0.000     -11.059      -6.148
C(fips_code)[T.12101]                        2.9013      1.252      2.317      0.021       0.447       5.356
C(fips_code)[T.12103]                       -5.1283      1.252     -4.095      0.000      -7.583      -2.673
C(fips_code)[T.12105]                        4.1511      1.255      3.308      0.001       1.691       6.611
C(fips_code)[T.12107]                       13.6304      1.252     10.886      0.000      11.176      16.085
C(fips_code)[T.12109]                        0.1369      1.253      0.109      0.913      -2.319       2.593
C(fips_code)[T.12111]                        1.7945      1.253      1.433      0.152      -0.661       4.250
C(fips_code)[T.12113]                       16.9314      1.254     13.507      0.000      14.474      19.388
C(fips_code)[T.12115]                      -10.4015      1.252     -8.306      0.000     -12.856      -7.947
C(fips_code)[T.12117]                       -4.1712      1.252     -3.331      0.001      -6.626      -1.717
C(fips_code)[T.12119]                      -10.8843      1.252     -8.692      0.000     -13.339      -8.430
C(fips_code)[T.12121]                        8.8173      1.255      7.025      0.000       6.357      11.278
C(fips_code)[T.12127]                        3.4120      1.253      2.724      0.006       0.957       5.867
C(fips_code)[T.12129]                       17.1930      1.356     12.681      0.000      14.535      19.851
C(fips_code)[T.12131]                        8.7689      1.252      7.004      0.000       6.315      11.223
C(fips_code)[T.13009]                        2.2600      1.254      1.802      0.071      -0.198       4.718
C(fips_code)[T.13013]                        3.0316      1.253      2.419      0.016       0.575       5.488
C(fips_code)[T.13015]                        0.4170      1.253      0.333      0.739      -2.040       2.874
C(fips_code)[T.13021]                       -3.4734      1.253     -2.772      0.006      -5.929      -1.017
C(fips_code)[T.13029]                       -2.1628      1.253     -1.726      0.084      -4.619       0.293
C(fips_code)[T.13031]                       -3.6750      1.253     -2.933      0.003      -6.131      -1.219
C(fips_code)[T.13039]                       -0.9240      1.254     -0.737      0.461      -3.382       1.534
C(fips_code)[T.13045]                        4.1317      1.253      3.297      0.001       1.676       6.588
C(fips_code)[T.13047]                        0.8838      1.255      0.704      0.481      -1.576       3.343
C(fips_code)[T.13051]                       -5.0298      1.256     -4.005      0.000      -7.491      -2.568
C(fips_code)[T.13057]                       -4.1780      1.253     -3.334      0.001      -6.635      -1.721
C(fips_code)[T.13059]                      -15.7757      1.253    -12.591      0.000     -18.232     -13.320
C(fips_code)[T.13063]                        6.3968      1.258      5.084      0.000       3.930       8.863
C(fips_code)[T.13067]                      -13.3858      1.253    -10.683      0.000     -15.842     -10.930
C(fips_code)[T.13069]                        5.7361      1.254      4.575      0.000       3.279       8.194
C(fips_code)[T.13071]                       -0.8306      1.256     -0.661      0.509      -3.293       1.632
C(fips_code)[T.13073]                       -0.1078      1.255     -0.086      0.932      -2.568       2.352
C(fips_code)[T.13077]                       -3.0799      1.253     -2.458      0.014      -5.536      -0.624
C(fips_code)[T.13089]                      -10.4441      1.254     -8.330      0.000     -12.902      -7.987
C(fips_code)[T.13095]                       -2.8523      1.267     -2.252      0.024      -5.335      -0.369
C(fips_code)[T.13097]                        1.5951      1.253      1.273      0.203      -0.862       4.052
C(fips_code)[T.13103]                        7.1525      1.253      5.707      0.000       4.696       9.609
C(fips_code)[T.13113]                       -5.1068      1.253     -4.075      0.000      -7.563      -2.650
C(fips_code)[T.13115]                        1.3817      1.253      1.103      0.270      -1.074       3.838
C(fips_code)[T.13117]                      -10.1444      1.254     -8.092      0.000     -12.602      -7.687
C(fips_code)[T.13121]                      -18.2374      1.254    -14.538      0.000     -20.696     -15.779
C(fips_code)[T.13127]                       -0.7405      1.256     -0.590      0.555      -3.202       1.721
C(fips_code)[T.13129]                        3.5337      1.253      2.820      0.005       1.077       5.990
C(fips_code)[T.13135]                       -9.8262      1.253     -7.842      0.000     -12.282      -7.370
C(fips_code)[T.13137]                        2.5904      1.254      2.065      0.039       0.132       5.049
C(fips_code)[T.13139]                       -2.7089      1.254     -2.160      0.031      -5.167      -0.250
C(fips_code)[T.13151]                       -1.1426      1.254     -0.911      0.362      -3.600       1.315
C(fips_code)[T.13153]                       -2.8085      1.254     -2.240      0.025      -5.266      -0.351
C(fips_code)[T.13157]                        4.7733      1.254      3.808      0.000       2.316       7.231
C(fips_code)[T.13175]                        0.8907      1.253      0.711      0.477      -1.566       3.347
C(fips_code)[T.13179]                        0.7157      1.253      0.571      0.568      -1.741       3.172
C(fips_code)[T.13185]                        0.3145      1.253      0.251      0.802      -2.142       2.771
C(fips_code)[T.13213]                       12.2306      1.658      7.375      0.000       8.980      15.481
C(fips_code)[T.13215]                       -1.8876      1.254     -1.505      0.132      -4.345       0.570
C(fips_code)[T.13217]                        5.4204      1.253      4.325      0.000       2.964       7.877
C(fips_code)[T.13223]                        1.9274      1.253      1.538      0.124      -0.529       4.384
C(fips_code)[T.13233]                       10.2726      1.253      8.198      0.000       7.816      12.729
C(fips_code)[T.13245]                       -3.6538      1.253     -2.916      0.004      -6.110      -1.197
C(fips_code)[T.13247]                        0.1454      1.253      0.116      0.908      -2.311       2.602
C(fips_code)[T.13255]                        7.6989      1.253      6.143      0.000       5.242      10.155
C(fips_code)[T.13275]                        2.6996      1.255      2.152      0.031       0.241       5.159
C(fips_code)[T.13277]                       -1.0076      1.255     -0.803      0.422      -3.467       1.452
C(fips_code)[T.13285]                        4.9493      1.254      3.948      0.000       2.492       7.407
C(fips_code)[T.13295]                        9.8407      1.254      7.849      0.000       7.383      12.298
C(fips_code)[T.13297]                        7.2803      1.253      5.810      0.000       4.824       9.736
C(fips_code)[T.13299]                        6.8520      1.354      5.062      0.000       4.198       9.505
C(fips_code)[T.13313]                        5.2384      1.257      4.167      0.000       2.774       7.703
C(fips_code)[T.16001]                      -10.4311      1.223     -8.527      0.000     -12.829      -8.033
C(fips_code)[T.16005]                       -1.3489      1.223     -1.103      0.270      -3.746       1.049
C(fips_code)[T.16011]                        6.9703      1.225      5.689      0.000       4.569       9.372
C(fips_code)[T.16017]                       12.1520      1.230      9.876      0.000       9.740      14.564
C(fips_code)[T.16019]                        0.3180      1.224      0.260      0.795      -2.082       2.718
C(fips_code)[T.16027]                        2.1988      1.223      1.797      0.072      -0.199       4.597
C(fips_code)[T.16039]                        7.9861      1.441      5.542      0.000       5.161      10.811
C(fips_code)[T.16055]                        3.9182      1.227      3.192      0.001       1.512       6.324
C(fips_code)[T.16057]                      -11.2322      1.268     -8.860      0.000     -13.717      -8.747
C(fips_code)[T.16065]                      -11.7723      1.228     -9.584      0.000     -14.180      -9.364
C(fips_code)[T.16069]                       -0.4884      1.224     -0.399      0.690      -2.888       1.911
C(fips_code)[T.16083]                       -0.6535      1.223     -0.534      0.593      -3.052       1.745
C(fips_code)[T.17001]                       -3.3431      1.265     -2.643      0.008      -5.822      -0.864
C(fips_code)[T.17007]                       10.6949      1.270      8.420      0.000       8.205      13.185
C(fips_code)[T.17011]                        2.4248      2.704      0.897      0.370      -2.875       7.725
C(fips_code)[T.17019]                      -11.2893      1.265     -8.922      0.000     -13.770      -8.809
C(fips_code)[T.17021]                        1.2854      2.704      0.475      0.635      -4.015       6.585
C(fips_code)[T.17027]                       15.5492      1.491     10.431      0.000      12.627      18.471
C(fips_code)[T.17029]                        5.3452      1.264      4.230      0.000       2.868       7.822
C(fips_code)[T.17031]                      -10.5199      1.266     -8.312      0.000     -13.001      -8.039
C(fips_code)[T.17037]                       -6.7156      1.261     -5.324      0.000      -9.188      -4.243
C(fips_code)[T.17043]                      -14.1855      1.262    -11.243      0.000     -16.659     -11.712
C(fips_code)[T.17049]                       -1.2877      1.662     -0.775      0.438      -4.546       1.970
C(fips_code)[T.17055]                       14.0412      1.268     11.070      0.000      11.555      16.527
C(fips_code)[T.17057]                        6.2543      1.261      4.958      0.000       3.782       8.727
C(fips_code)[T.17063]                        8.4807      1.262      6.722      0.000       6.008      10.954
C(fips_code)[T.17073]                        0.0893      1.261      0.071      0.944      -2.383       2.562
C(fips_code)[T.17075]                        4.9037      2.704      1.814      0.070      -0.396      10.204
C(fips_code)[T.17077]                       -9.1038      1.262     -7.216      0.000     -11.577      -6.631
C(fips_code)[T.17081]                        8.8134      1.271      6.935      0.000       6.322      11.305
C(fips_code)[T.17089]                       -7.5025      1.262     -5.944      0.000      -9.977      -5.028
C(fips_code)[T.17091]                        0.7427      1.262      0.589      0.556      -1.731       3.216
C(fips_code)[T.17093]                        7.6378      1.261      6.055      0.000       5.165      10.110
C(fips_code)[T.17095]                       -1.4765      1.262     -1.170      0.242      -3.949       0.996
C(fips_code)[T.17097]                       -9.1200      1.262     -7.224      0.000     -11.594      -6.646
C(fips_code)[T.17099]                        0.5612      1.262      0.445      0.657      -1.913       3.035
C(fips_code)[T.17103]                        3.8701      1.362      2.841      0.004       1.200       6.540
C(fips_code)[T.17105]                       -1.0454      1.264     -0.827      0.408      -3.523       1.433
C(fips_code)[T.17107]                        5.5987      2.704      2.071      0.038       0.299      10.899
C(fips_code)[T.17111]                       -2.5462      1.261     -2.019      0.044      -5.018      -0.074
C(fips_code)[T.17113]                      -10.8961      1.263     -8.626      0.000     -13.372      -8.420
C(fips_code)[T.17115]                        3.4652      1.261      2.747      0.006       0.993       5.937
C(fips_code)[T.17117]                       -4.8498      1.568     -3.093      0.002      -7.923      -1.776
C(fips_code)[T.17119]                       -0.1760      1.262     -0.139      0.889      -2.649       2.297
C(fips_code)[T.17121]                        3.2473      1.265      2.567      0.010       0.768       5.727
C(fips_code)[T.17133]                       -2.4722      2.704     -0.914      0.361      -7.773       2.828
C(fips_code)[T.17137]                        4.2939      2.704      1.588      0.112      -1.006       9.594
C(fips_code)[T.17141]                       -6.3917      1.261     -5.067      0.000      -8.864      -3.919
C(fips_code)[T.17143]                       -4.6364      1.263     -3.670      0.000      -7.113      -2.160
C(fips_code)[T.17157]                        6.3714      2.343      2.719      0.007       1.778      10.965
C(fips_code)[T.17161]                       -7.7100      1.262     -6.109      0.000     -10.184      -5.236
C(fips_code)[T.17163]                       -1.8179      1.261     -1.441      0.150      -4.290       0.654
C(fips_code)[T.17167]                       -5.8843      1.262     -4.664      0.000      -8.357      -3.411
C(fips_code)[T.17177]                        0.9319      1.263      0.738      0.461      -1.544       3.408
C(fips_code)[T.17179]                        1.5408      1.261      1.221      0.222      -0.932       4.013
C(fips_code)[T.17183]                        8.6639      1.261      6.868      0.000       6.191      11.136
C(fips_code)[T.17195]                        1.3887      1.261      1.101      0.271      -1.083       3.861
C(fips_code)[T.17197]                       -3.8842      1.262     -3.077      0.002      -6.359      -1.410
C(fips_code)[T.17199]                        6.3891      1.263      5.060      0.000       3.914       8.864
C(fips_code)[T.17201]                        3.3478      1.268      2.641      0.008       0.863       5.832
C(fips_code)[T.17203]                       -7.3544      2.344     -3.138      0.002     -11.948      -2.761
C(fips_code)[T.18003]                       -6.2825      1.254     -5.010      0.000      -8.740      -3.825
C(fips_code)[T.18005]                       -4.8027      1.253     -3.832      0.000      -7.259      -2.346
C(fips_code)[T.18011]                       -7.6353      1.261     -6.054      0.000     -10.108      -5.163
C(fips_code)[T.18017]                        0.0382      1.271      0.030      0.976      -2.453       2.529
C(fips_code)[T.18019]                       -3.7468      1.253     -2.990      0.003      -6.203      -1.290
C(fips_code)[T.18023]                       -0.3375      1.254     -0.269      0.788      -2.796       2.121
C(fips_code)[T.18029]                        0.5015      1.253      0.400      0.689      -1.955       2.958
C(fips_code)[T.18033]                       -0.0647      1.255     -0.052      0.959      -2.524       2.395
C(fips_code)[T.18035]                       -5.6884      1.253     -4.540      0.000      -8.145      -3.232
C(fips_code)[T.18037]                       -7.0982      1.261     -5.630      0.000      -9.569      -4.627
C(fips_code)[T.18039]                        8.7958      1.258      6.994      0.000       6.331      11.261
C(fips_code)[T.18043]                       -3.4261      1.253     -2.733      0.006      -5.883      -0.969
C(fips_code)[T.18053]                        4.1843      1.253      3.338      0.001       1.727       6.641
C(fips_code)[T.18057]                      -15.7929      1.259    -12.545      0.000     -18.261     -13.325
C(fips_code)[T.18059]                       -3.6938      1.254     -2.945      0.003      -6.152      -1.235
C(fips_code)[T.18061]                       -1.0575      1.253     -0.844      0.399      -3.514       1.399
C(fips_code)[T.18063]                       -3.4911      1.258     -2.776      0.006      -5.957      -1.026
C(fips_code)[T.18065]                        7.9845      1.253      6.373      0.000       5.529      10.441
C(fips_code)[T.18067]                        3.5276      1.284      2.747      0.006       1.010       6.045
C(fips_code)[T.18069]                        2.0882      1.254      1.665      0.096      -0.369       4.546
C(fips_code)[T.18071]                       15.9360      1.257     12.682      0.000      13.473      18.399
C(fips_code)[T.18073]                        2.8410      1.253      2.267      0.023       0.384       5.297
C(fips_code)[T.18077]                        4.6753      1.414      3.306      0.001       1.903       7.447
C(fips_code)[T.18081]                       -6.0754      1.256     -4.838      0.000      -8.537      -3.614
C(fips_code)[T.18083]                        4.9966      1.413      3.535      0.000       2.226       7.767
C(fips_code)[T.18085]                        1.9604      1.253      1.564      0.118      -0.497       4.417
C(fips_code)[T.18087]                        5.8964      1.657      3.559      0.000       2.649       9.144
C(fips_code)[T.18089]                       -0.5283      1.257     -0.420      0.674      -2.993       1.936
C(fips_code)[T.18091]                        4.6242      1.256      3.681      0.000       2.162       7.086
C(fips_code)[T.18093]                        5.2235      1.253      4.168      0.000       2.767       7.680
C(fips_code)[T.18095]                        3.7969      1.254      3.028      0.002       1.339       6.255
C(fips_code)[T.18097]                      -11.9059      1.254     -9.493      0.000     -14.364      -9.447
C(fips_code)[T.18099]                        7.8006      1.253      6.225      0.000       5.344      10.257
C(fips_code)[T.18103]                        7.7572      1.771      4.380      0.000       4.285      11.229
C(fips_code)[T.18105]                      -19.7726      1.258    -15.717      0.000     -22.238     -17.307
C(fips_code)[T.18107]                        4.6572      1.256      3.709      0.000       2.196       7.119
C(fips_code)[T.18109]                        3.5490      1.256      2.825      0.005       1.087       6.011
C(fips_code)[T.18113]                       11.5652      1.262      9.165      0.000       9.092      14.039
C(fips_code)[T.18127]                       -4.1846      1.254     -3.338      0.001      -6.642      -1.727
C(fips_code)[T.18141]                       -3.4449      1.254     -2.747      0.006      -5.903      -0.987
C(fips_code)[T.18145]                       -0.3689      1.254     -0.294      0.769      -2.828       2.090
C(fips_code)[T.18151]                        9.5234      1.253      7.599      0.000       7.067      11.980
C(fips_code)[T.18157]                      -13.6933      1.254    -10.918      0.000     -16.152     -11.235
C(fips_code)[T.18163]                       -6.0684      1.253     -4.842      0.000      -8.525      -3.612
C(fips_code)[T.18167]                       -0.7155      1.253     -0.571      0.568      -3.172       1.741
C(fips_code)[T.18173]                        3.0567      1.255      2.435      0.015       0.596       5.518
C(fips_code)[T.18177]                       -2.7839      1.254     -2.221      0.026      -5.241      -0.326
C(fips_code)[T.18179]                        6.3233      1.771      3.571      0.000       2.853       9.794
C(fips_code)[T.18183]                        2.4551      1.253      1.959      0.050      -0.001       4.912
C(fips_code)[T.20015]                        9.3996      1.241      7.572      0.000       6.966      11.833
C(fips_code)[T.20035]                        9.1197      1.241      7.349      0.000       6.687      11.552
C(fips_code)[T.20037]                        0.6440      1.240      0.519      0.604      -1.787       3.075
C(fips_code)[T.20045]                      -12.5300      1.237    -10.127      0.000     -14.955     -10.105
C(fips_code)[T.20055]                       -2.8316      1.255     -2.255      0.024      -5.292      -0.371
C(fips_code)[T.20057]                       -8.0584      1.307     -6.165      0.000     -10.621      -5.496
C(fips_code)[T.20059]                        5.5772      1.283      4.346      0.000       3.062       8.093
C(fips_code)[T.20061]                        1.9616      1.237      1.585      0.113      -0.464       4.387
C(fips_code)[T.20079]                       -0.4267      1.237     -0.345      0.730      -2.851       1.998
C(fips_code)[T.20091]                      -12.8493      1.237    -10.387      0.000     -15.274     -10.425
C(fips_code)[T.20103]                        3.7059      1.238      2.994      0.003       1.280       6.132
C(fips_code)[T.20111]                       -6.1643      1.238     -4.979      0.000      -8.591      -3.738
C(fips_code)[T.20113]                       -2.1782      1.241     -1.755      0.079      -4.611       0.254
C(fips_code)[T.20121]                        8.2484      1.237      6.667      0.000       5.823      10.673
C(fips_code)[T.20125]                       16.1849      1.238     13.072      0.000      13.758      18.612
C(fips_code)[T.20155]                        4.8985      1.237      3.960      0.000       2.474       7.323
C(fips_code)[T.20161]                      -15.9080      1.237    -12.856      0.000     -18.334     -13.483
C(fips_code)[T.20169]                        1.5169      1.237      1.226      0.220      -0.908       3.942
C(fips_code)[T.20173]                        1.3394      1.246      1.075      0.282      -1.102       3.781
C(fips_code)[T.20177]                        0.7674      1.237      0.620      0.535      -1.657       3.192
C(fips_code)[T.20209]                       -1.1777      0.903     -1.304      0.192      -2.948       0.592
C(fips_code)[T.21015]                       -5.1443      0.750     -6.863      0.000      -6.614      -3.675
C(fips_code)[T.21019]                       -2.4872      1.245     -1.998      0.046      -4.928      -0.047
C(fips_code)[T.21029]                        5.6470      1.244      4.540      0.000       3.209       8.085
C(fips_code)[T.21035]                       -2.7351      1.893     -1.445      0.149      -6.446       0.976
C(fips_code)[T.21037]                       -9.9843      1.244     -8.023      0.000     -12.424      -7.545
C(fips_code)[T.21047]                        7.7637      1.244      6.239      0.000       5.325      10.203
C(fips_code)[T.21049]                        0.9519      1.547      0.615      0.538      -2.081       3.985
C(fips_code)[T.21059]                       -3.6992      1.244     -2.975      0.003      -6.137      -1.262
C(fips_code)[T.21067]                      -10.9469      1.244     -8.799      0.000     -13.386      -8.508
C(fips_code)[T.21073]                        0.2082      1.244      0.167      0.867      -2.229       2.646
C(fips_code)[T.21083]                        2.7539      1.469      1.875      0.061      -0.126       5.633
C(fips_code)[T.21093]                       -0.8363      1.244     -0.672      0.501      -3.275       1.602
C(fips_code)[T.21101]                        2.0545      1.243      1.652      0.098      -0.383       4.492
C(fips_code)[T.21107]                        8.6972      1.248      6.968      0.000       6.251      11.144
C(fips_code)[T.21111]                       -6.7518      1.244     -5.429      0.000      -9.189      -4.314
C(fips_code)[T.21113]                        0.8006      1.244      0.643      0.520      -1.638       3.240
C(fips_code)[T.21117]                       -4.4325      1.244     -3.562      0.000      -6.871      -1.994
C(fips_code)[T.21125]                        7.8894      1.244      6.342      0.000       5.451      10.328
C(fips_code)[T.21145]                       -2.6384      1.244     -2.122      0.034      -5.076      -0.201
C(fips_code)[T.21151]                        0.3055      1.244      0.246      0.806      -2.132       2.743
C(fips_code)[T.21179]                        6.0996      1.245      4.901      0.000       3.660       8.539
C(fips_code)[T.21185]                       -0.4442      1.245     -0.357      0.721      -2.884       1.995
C(fips_code)[T.21199]                        9.5425      1.244      7.672      0.000       7.105      11.981
C(fips_code)[T.21209]                        2.4364      1.244      1.958      0.050      -0.003       4.875
C(fips_code)[T.21211]                       -2.4335      1.244     -1.957      0.050      -4.871       0.004
C(fips_code)[T.21227]                       -1.6638      1.245     -1.337      0.181      -4.104       0.776
C(fips_code)[T.22001]                        9.0549      1.243      7.286      0.000       6.619      11.491
C(fips_code)[T.22005]                       -3.7618      1.243     -3.027      0.002      -6.198      -1.326
C(fips_code)[T.22015]                       -6.7802      1.244     -5.450      0.000      -9.219      -4.342
C(fips_code)[T.22017]                        1.6443      1.243      1.323      0.186      -0.791       4.080
C(fips_code)[T.22019]                       -1.6755      0.897     -1.869      0.062      -3.433       0.082
C(fips_code)[T.22033]                       -8.6967      1.242     -7.001      0.000     -11.131      -6.262
C(fips_code)[T.22045]                       13.2344      1.242     10.654      0.000      10.800      15.669
C(fips_code)[T.22051]                       -7.9751      1.247     -6.398      0.000     -10.419      -5.532
C(fips_code)[T.22055]                       -5.3658      1.243     -4.316      0.000      -7.803      -2.929
C(fips_code)[T.22057]                        4.4669      1.244      3.591      0.000       2.029       6.905
C(fips_code)[T.22063]                        3.3212      1.243      2.671      0.008       0.884       5.758
C(fips_code)[T.22071]                      -25.0926      1.256    -19.981      0.000     -27.554     -22.631
C(fips_code)[T.22073]                       -1.7938      1.243     -1.444      0.149      -4.230       0.642
C(fips_code)[T.22079]                        0.0768      1.245      0.062      0.951      -2.363       2.517
C(fips_code)[T.22087]                        7.2488      1.247      5.814      0.000       4.805       9.693
C(fips_code)[T.22089]                      -10.4309      1.243     -8.393      0.000     -12.867      -7.995
C(fips_code)[T.22095]                        2.0212      1.258      1.607      0.108      -0.444       4.486
C(fips_code)[T.22097]                        7.3703      1.242      5.932      0.000       4.935       9.805
C(fips_code)[T.22099]                        2.3595      1.242      1.900      0.057      -0.075       4.794
C(fips_code)[T.22101]                        3.9099      1.242      3.147      0.002       1.475       6.345
C(fips_code)[T.22103]                       -4.3887      1.242     -3.533      0.000      -6.823      -1.954
C(fips_code)[T.22105]                        9.5049      1.245      7.637      0.000       7.065      11.944
C(fips_code)[T.22109]                       -4.5774      1.242     -3.685      0.000      -7.012      -2.143
C(fips_code)[T.22113]                       11.2989      1.244      9.083      0.000       8.861      13.737
C(fips_code)[T.22115]                       -2.0028      1.244     -1.610      0.107      -4.441       0.435
C(fips_code)[T.22119]                        9.0437      1.641      5.511      0.000       5.827      12.260
C(fips_code)[T.23001]                       -3.3355      1.256     -2.656      0.008      -5.797      -0.874
C(fips_code)[T.23003]                        2.2335      1.256      1.779      0.075      -0.228       4.695
C(fips_code)[T.23005]                      -14.3914      1.256    -11.460      0.000     -16.853     -11.930
C(fips_code)[T.23009]                        5.6036      1.256      4.462      0.000       3.142       8.065
C(fips_code)[T.23011]                       -2.1381      1.256     -1.703      0.089      -4.599       0.323
C(fips_code)[T.23013]                        1.2757      2.040      0.625      0.532      -2.722       5.274
C(fips_code)[T.23015]                        1.9225      2.277      0.844      0.399      -2.541       6.386
C(fips_code)[T.23017]                        2.1606      1.256      1.720      0.085      -0.302       4.623
C(fips_code)[T.23019]                       -4.7601      1.256     -3.791      0.000      -7.221      -2.299
C(fips_code)[T.23023]                       -2.5446      1.866     -1.364      0.173      -6.203       1.113
C(fips_code)[T.23025]                       11.4992      1.256      9.152      0.000       9.036      13.962
C(fips_code)[T.23027]                        3.9102      1.626      2.405      0.016       0.724       7.097
C(fips_code)[T.23031]                       -2.5856      1.256     -2.059      0.039      -5.047      -0.125
C(fips_code)[T.24001]                        1.0261      1.238      0.828      0.407      -1.401       3.454
C(fips_code)[T.24003]                       -8.9452      1.237     -7.232      0.000     -11.370      -6.521
C(fips_code)[T.24005]                       -2.8137      1.237     -2.275      0.023      -5.238      -0.389
C(fips_code)[T.24009]                        1.4386      1.238      1.162      0.245      -0.988       3.865
C(fips_code)[T.24011]                        5.1544      1.540      3.347      0.001       2.135       8.173
C(fips_code)[T.24013]                       -0.3138      1.238     -0.253      0.800      -2.740       2.113
C(fips_code)[T.24015]                       10.9400      1.237      8.844      0.000       8.515      13.365
C(fips_code)[T.24017]                        0.1978      1.237      0.160      0.873      -2.227       2.622
C(fips_code)[T.24019]                       13.0702      1.283     10.186      0.000      10.555      15.585
C(fips_code)[T.24021]                       -7.0118      1.237     -5.669      0.000      -9.436      -4.587
C(fips_code)[T.24025]                       -2.2846      1.237     -1.847      0.065      -4.709       0.140
C(fips_code)[T.24027]                      -16.2037      1.238    -13.090      0.000     -18.630     -13.777
C(fips_code)[T.24031]                      -14.6483      1.238    -11.834      0.000     -17.075     -12.222
C(fips_code)[T.24033]                        1.1469      1.238      0.926      0.354      -1.281       3.574
C(fips_code)[T.24035]                       -1.0888      1.237     -0.880      0.379      -3.514       1.336
C(fips_code)[T.24037]                       -0.7484      1.239     -0.604      0.546      -3.177       1.680
C(fips_code)[T.24043]                       -0.1289      1.237     -0.104      0.917      -2.554       2.296
C(fips_code)[T.24045]                       -0.8331      1.238     -0.673      0.501      -3.260       1.594
C(fips_code)[T.24047]                       20.9768      1.261     16.629      0.000      18.504      23.449
C(fips_code)[T.24510]                       -3.7481      1.238     -3.029      0.002      -6.174      -1.322
C(fips_code)[T.25001]                        6.7903      1.226      5.539      0.000       4.388       9.193
C(fips_code)[T.25003]                        4.3980      1.223      3.596      0.000       2.000       6.796
C(fips_code)[T.25005]                        8.6189      1.223      7.045      0.000       6.221      11.017
C(fips_code)[T.25009]                        1.4616      1.222      1.196      0.232      -0.934       3.857
C(fips_code)[T.25011]                        1.9531      1.223      1.597      0.110      -0.445       4.351
C(fips_code)[T.25013]                        5.9995      1.222      4.908      0.000       3.604       8.396
C(fips_code)[T.25015]                       -6.6396      1.225     -5.421      0.000      -9.040      -4.239
C(fips_code)[T.25017]                      -11.5734      1.223     -9.461      0.000     -13.971      -9.176
C(fips_code)[T.25021]                       -5.5014      1.221     -4.504      0.000      -7.896      -3.107
C(fips_code)[T.25023]                        5.3717      1.222      4.397      0.000       2.977       7.766
C(fips_code)[T.25025]                      -18.8721      1.222    -15.439      0.000     -21.268     -16.476
C(fips_code)[T.25027]                        2.2330      1.221      1.829      0.067      -0.160       4.626
C(fips_code)[T.26005]                        5.0979      1.252      4.071      0.000       2.643       7.553
C(fips_code)[T.26015]                        4.5690      1.253      3.647      0.000       2.113       7.025
C(fips_code)[T.26017]                       -0.7356      1.251     -0.588      0.557      -3.188       1.716
C(fips_code)[T.26021]                        4.6894      1.252      3.746      0.000       2.235       7.143
C(fips_code)[T.26023]                        6.7420      1.251      5.388      0.000       4.289       9.195
C(fips_code)[T.26025]                        1.7240      1.251      1.378      0.168      -0.729       4.177
C(fips_code)[T.26027]                        0.0419      1.654      0.025      0.980      -3.200       3.283
C(fips_code)[T.26037]                       -4.1679      1.256     -3.319      0.001      -6.629      -1.706
C(fips_code)[T.26045]                       -5.5112      1.252     -4.402      0.000      -7.965      -3.057
C(fips_code)[T.26049]                        2.3800      1.257      1.894      0.058      -0.084       4.844
C(fips_code)[T.26055]                       -5.8173      1.251     -4.649      0.000      -8.270      -3.365
C(fips_code)[T.26057]                        2.5594      1.252      2.045      0.041       0.106       5.013
C(fips_code)[T.26059]                        7.5725      1.253      6.045      0.000       5.117      10.028
C(fips_code)[T.26065]                      -17.7842      1.255    -14.168      0.000     -20.245     -15.324
C(fips_code)[T.26067]                        8.7080      1.251      6.959      0.000       6.255      11.161
C(fips_code)[T.26073]                       -6.4651      1.252     -5.164      0.000      -8.919      -4.011
C(fips_code)[T.26075]                        0.3330      1.251      0.266      0.790      -2.119       2.785
C(fips_code)[T.26077]                      -11.3263      1.257     -9.011      0.000     -13.790      -8.863
C(fips_code)[T.26081]                      -12.8218      1.253    -10.235      0.000     -15.277     -10.366
C(fips_code)[T.26087]                        5.9561      1.261      4.721      0.000       3.483       8.429
C(fips_code)[T.26091]                       -0.7527      1.253     -0.601      0.548      -3.208       1.702
C(fips_code)[T.26093]                      -11.5169      1.251     -9.205      0.000     -13.969      -9.064
C(fips_code)[T.26099]                       -4.2841      1.258     -3.405      0.001      -6.750      -1.818
C(fips_code)[T.26103]                       -8.2812      1.252     -6.616      0.000     -10.735      -5.828
C(fips_code)[T.26107]                        7.7693      1.479      5.253      0.000       4.870      10.669
C(fips_code)[T.26111]                       -9.2257      1.254     -7.358      0.000     -11.683      -6.768
C(fips_code)[T.26115]                       -2.0617      1.251     -1.648      0.099      -4.514       0.391
C(fips_code)[T.26117]                       12.9653      1.251     10.360      0.000      10.512      15.418
C(fips_code)[T.26121]                        6.2291      1.255      4.962      0.000       3.768       8.690
C(fips_code)[T.26123]                       10.3575      1.251      8.278      0.000       7.905      12.810
C(fips_code)[T.26125]                      -16.3811      1.254    -13.068      0.000     -18.838     -13.924
C(fips_code)[T.26139]                       -4.4708      1.254     -3.566      0.000      -6.928      -2.013
C(fips_code)[T.26145]                       -5.1113      1.252     -4.084      0.000      -7.565      -2.658
C(fips_code)[T.26147]                        2.5760      1.258      2.048      0.041       0.111       5.041
C(fips_code)[T.26149]                       13.3852      1.252     10.690      0.000      10.931      15.839
C(fips_code)[T.26151]                        8.0860      1.560      5.184      0.000       5.028      11.144
C(fips_code)[T.26155]                        8.2162      1.253      6.559      0.000       5.761      10.672
C(fips_code)[T.26157]                        5.6999      1.257      4.535      0.000       3.236       8.164
C(fips_code)[T.26159]                       12.9231      1.252     10.319      0.000      10.468      15.378
C(fips_code)[T.26161]                      -22.6363      1.262    -17.938      0.000     -25.110     -20.163
C(fips_code)[T.26163]                       -2.2749      1.264     -1.800      0.072      -4.752       0.202
C(fips_code)[T.26165]                       14.2705      1.560      9.150      0.000      11.214      17.327
C(fips_code)[T.27003]                       -1.0023      1.249     -0.802      0.422      -3.451       1.446
C(fips_code)[T.27005]                        5.5601      1.349      4.122      0.000       2.916       8.204
C(fips_code)[T.27007]                        4.7772      1.249      3.824      0.000       2.329       7.226
C(fips_code)[T.27009]                        1.8718      1.249      1.498      0.134      -0.577       4.320
C(fips_code)[T.27013]                      -11.8986      1.249     -9.526      0.000     -14.347      -9.450
C(fips_code)[T.27019]                       -3.9376      1.249     -3.152      0.002      -6.386      -1.489
C(fips_code)[T.27025]                       -3.7789      1.249     -3.025      0.002      -6.228      -1.330
C(fips_code)[T.27027]                        0.8107      1.253      0.647      0.518      -1.645       3.266
C(fips_code)[T.27035]                        5.8521      1.250      4.680      0.000       3.401       8.303
C(fips_code)[T.27037]                       -4.5259      1.249     -3.623      0.000      -6.974      -2.078
C(fips_code)[T.27041]                        1.3284      1.249      1.063      0.288      -1.121       3.777
C(fips_code)[T.27047]                        2.3303      1.249      1.866      0.062      -0.118       4.779
C(fips_code)[T.27049]                        3.5254      1.249      2.822      0.005       1.077       5.974
C(fips_code)[T.27053]                      -16.4643      1.250    -13.171      0.000     -18.915     -14.014
C(fips_code)[T.27059]                        6.0756      1.250      4.862      0.000       3.626       8.525
C(fips_code)[T.27061]                        4.6895      1.253      3.743      0.000       2.234       7.145
C(fips_code)[T.27067]                       -2.4011      1.251     -1.920      0.055      -4.853       0.051
C(fips_code)[T.27079]                       55.6096      2.087     26.642      0.000      51.518      59.701
C(fips_code)[T.27085]                        3.1798      1.249      2.545      0.011       0.731       5.628
C(fips_code)[T.27097]                        8.0720      1.296      6.228      0.000       5.532      10.612
C(fips_code)[T.27099]                       -2.6125      1.250     -2.089      0.037      -5.063      -0.162
C(fips_code)[T.27103]                       -8.4017      1.557     -5.397      0.000     -11.453      -5.350
C(fips_code)[T.27109]                      -12.6301      1.249    -10.112      0.000     -15.078     -10.182
C(fips_code)[T.27111]                        5.3584      1.249      4.289      0.000       2.910       7.807
C(fips_code)[T.27119]                       -7.8214      1.556     -5.026      0.000     -10.872      -4.771
C(fips_code)[T.27123]                      -15.9321      1.249    -12.752      0.000     -18.381     -13.483
C(fips_code)[T.27131]                       -3.3472      1.250     -2.679      0.007      -5.796      -0.898
C(fips_code)[T.27137]                       -6.9562      1.250     -5.564      0.000      -9.407      -4.506
C(fips_code)[T.27139]                       -4.5814      1.249     -3.668      0.000      -7.030      -2.133
C(fips_code)[T.27141]                       10.1695      1.249      8.142      0.000       7.721      12.618
C(fips_code)[T.27145]                       -7.2630      1.250     -5.810      0.000      -9.713      -4.813
C(fips_code)[T.27147]                        0.9531      1.249      0.763      0.446      -1.496       3.402
C(fips_code)[T.27163]                       -4.0220      1.249     -3.220      0.001      -6.470      -1.574
C(fips_code)[T.27169]                       -4.5212      1.250     -3.618      0.000      -6.971      -2.072
C(fips_code)[T.27171]                        0.4546      1.249      0.364      0.716      -1.994       2.903
C(fips_code)[T.28033]                       -4.1427      1.243     -3.334      0.001      -6.578      -1.707
C(fips_code)[T.28035]                       -2.3394      1.241     -1.885      0.059      -4.772       0.093
C(fips_code)[T.28045]                        7.0072      1.242      5.644      0.000       4.574       9.441
C(fips_code)[T.28047]                        1.5603      1.247      1.251      0.211      -0.884       4.005
C(fips_code)[T.28049]                        2.6571      1.241      2.141      0.032       0.224       5.090
C(fips_code)[T.28059]                        6.2498      1.245      5.019      0.000       3.809       8.691
C(fips_code)[T.28067]                        5.5573      1.243      4.471      0.000       3.121       7.994
C(fips_code)[T.28071]                       -6.7448      2.693     -2.505      0.012     -12.023      -1.466
C(fips_code)[T.28073]                      -10.0677      1.243     -8.102      0.000     -12.503      -7.632
C(fips_code)[T.28075]                       -1.9744      1.244     -1.587      0.112      -4.412       0.464
C(fips_code)[T.28081]                       -3.5456      1.242     -2.854      0.004      -5.981      -1.110
C(fips_code)[T.28087]                       -3.7915      1.240     -3.057      0.002      -6.223      -1.360
C(fips_code)[T.28089]                       -2.8230      1.241     -2.276      0.023      -5.255      -0.391
C(fips_code)[T.28109]                        6.1491      1.242      4.953      0.000       3.716       8.583
C(fips_code)[T.28121]                       -7.4541      1.243     -5.995      0.000      -9.891      -5.017
C(fips_code)[T.28149]                        6.9089      1.241      5.567      0.000       4.476       9.342
C(fips_code)[T.28151]                        9.1976      1.241      7.411      0.000       6.765      11.630
C(fips_code)[T.29009]                        8.9096      1.247      7.143      0.000       6.465      11.355
C(fips_code)[T.29019]                      -16.7121      1.251    -13.362      0.000     -19.164     -14.260
C(fips_code)[T.29021]                       -2.2270      1.249     -1.783      0.075      -4.676       0.222
C(fips_code)[T.29023]                        9.7734      1.248      7.834      0.000       7.328      12.219
C(fips_code)[T.29027]                       -1.7728      1.249     -1.419      0.156      -4.222       0.676
C(fips_code)[T.29029]                       17.9492      1.249     14.373      0.000      15.501      20.397
C(fips_code)[T.29031]                       -5.1278      1.247     -4.111      0.000      -7.573      -2.683
C(fips_code)[T.29037]                        3.5421      1.249      2.837      0.005       1.095       5.990
C(fips_code)[T.29043]                        4.3744      1.248      3.506      0.000       1.929       6.820
C(fips_code)[T.29047]                       -6.3658      1.249     -5.096      0.000      -8.814      -3.917
C(fips_code)[T.29051]                       -3.3807      1.250     -2.704      0.007      -5.832      -0.930
C(fips_code)[T.29071]                        2.4395      1.248      1.955      0.051      -0.006       4.885
C(fips_code)[T.29077]                       -6.8879      1.248     -5.517      0.000      -9.335      -4.441
C(fips_code)[T.29091]                        3.7444      1.247      3.002      0.003       1.299       6.189
C(fips_code)[T.29095]                      -11.2665      1.249     -9.021      0.000     -13.715      -8.818
C(fips_code)[T.29097]                        4.3274      1.247      3.469      0.001       1.882       6.772
C(fips_code)[T.29099]                       -0.7952      1.248     -0.637      0.524      -3.242       1.652
C(fips_code)[T.29101]                       -1.5068      1.248     -1.208      0.227      -3.952       0.939
C(fips_code)[T.29105]                       12.2176      1.251      9.765      0.000       9.765      14.670
C(fips_code)[T.29109]                       15.0697      1.248     12.080      0.000      12.624      17.515
C(fips_code)[T.29113]                       -0.9849      1.248     -0.789      0.430      -3.431       1.462
C(fips_code)[T.29127]                        1.2280      1.249      0.983      0.326      -1.220       3.677
C(fips_code)[T.29145]                        4.6247      1.247      3.708      0.000       2.180       7.070
C(fips_code)[T.29159]                        5.5997      1.248      4.488      0.000       3.154       8.045
C(fips_code)[T.29161]                       -4.2987      1.249     -3.441      0.001      -6.747      -1.850
C(fips_code)[T.29165]                      -18.6782      1.248    -14.963      0.000     -21.125     -16.231
C(fips_code)[T.29167]                        3.7540      1.250      3.004      0.003       1.305       6.203
C(fips_code)[T.29169]                       -3.0987      1.248     -2.483      0.013      -5.545      -0.653
C(fips_code)[T.29183]                      -12.5525      1.247    -10.062      0.000     -14.998     -10.107
C(fips_code)[T.29187]                        8.6555      1.248      6.938      0.000       6.210      11.101
C(fips_code)[T.29189]                      -17.1784      1.249    -13.750      0.000     -19.627     -14.730
C(fips_code)[T.29213]                       12.8604      1.286     10.000      0.000      10.340      15.381
C(fips_code)[T.29219]                        3.8734      1.247      3.105      0.002       1.428       6.318
C(fips_code)[T.29225]                       14.3318      1.248     11.487      0.000      11.886      16.777
C(fips_code)[T.29510]                      -19.2710      1.250    -15.413      0.000     -21.722     -16.820
C(fips_code)[T.30013]                        2.4386      1.211      2.014      0.044       0.065       4.812
C(fips_code)[T.30029]                        2.3665      1.215      1.948      0.051      -0.015       4.748
C(fips_code)[T.30031]                      -15.1850      1.211    -12.540      0.000     -17.558     -12.811
C(fips_code)[T.30049]                        2.8366      1.211      2.341      0.019       0.462       5.211
C(fips_code)[T.30063]                       -8.9194      1.211     -7.365      0.000     -11.293      -6.546
C(fips_code)[T.30081]                        7.3421      1.302      5.640      0.000       4.790       9.894
C(fips_code)[T.30093]                        5.2351      1.211      4.322      0.000       2.861       7.609
C(fips_code)[T.30111]                        1.8338      1.211      1.514      0.130      -0.540       4.208
C(fips_code)[T.32003]                       -6.9177      1.240     -5.578      0.000      -9.349      -4.487
C(fips_code)[T.32005]                      -11.2394      1.207     -9.314      0.000     -13.605      -8.874
C(fips_code)[T.32007]                       -2.1265      1.213     -1.754      0.080      -4.503       0.250
C(fips_code)[T.32019]                       12.0811      1.207     10.012      0.000       9.716      14.446
C(fips_code)[T.32023]                        9.3769      1.207      7.772      0.000       7.012      11.742
C(fips_code)[T.32031]                       -6.5136      1.207     -5.398      0.000      -8.879      -4.149
C(fips_code)[T.32510]                        1.0869      1.207      0.901      0.368      -1.278       3.452
C(fips_code)[T.33001]                        8.9435      1.226      7.295      0.000       6.540      11.347
C(fips_code)[T.33003]                       -2.1663      1.231     -1.760      0.078      -4.579       0.247
C(fips_code)[T.33005]                        0.3319      1.226      0.271      0.787      -2.071       2.735
C(fips_code)[T.33007]                        5.9023      1.844      3.202      0.001       2.289       9.516
C(fips_code)[T.33009]                       -9.2698      1.225     -7.566      0.000     -11.671      -6.868
C(fips_code)[T.33011]                       -9.7404      1.225     -7.949      0.000     -12.142      -7.339
C(fips_code)[T.33013]                       -5.7369      1.225     -4.682      0.000      -8.139      -3.335
C(fips_code)[T.33015]                       -3.8900      1.225     -3.176      0.001      -6.291      -1.489
C(fips_code)[T.33017]                       -0.3959      1.225     -0.323      0.747      -2.797       2.005
C(fips_code)[T.33019]                       10.8407      1.228      8.830      0.000       8.434      13.247
C(fips_code)[T.34001]                        3.8775      1.298      2.988      0.003       1.334       6.421
C(fips_code)[T.34003]                      -11.1777      1.239     -9.023      0.000     -13.606      -8.750
C(fips_code)[T.34005]                       -5.4848      1.240     -4.424      0.000      -7.915      -3.054
C(fips_code)[T.34007]                        0.7674      1.238      0.620      0.535      -1.659       3.194
C(fips_code)[T.34009]                       25.2895      1.263     20.028      0.000      22.814      27.764
C(fips_code)[T.34011]                       15.8605      1.239     12.801      0.000      13.432      18.289
C(fips_code)[T.34013]                       -4.1561      1.242     -3.345      0.001      -6.591      -1.721
C(fips_code)[T.34015]                        1.1219      1.239      0.905      0.365      -1.307       3.551
C(fips_code)[T.34017]                       -3.0308      1.240     -2.444      0.015      -5.461      -0.600
C(fips_code)[T.34019]                       -7.3027      1.243     -5.874      0.000      -9.740      -4.866
C(fips_code)[T.34021]                       -9.2462      1.243     -7.441      0.000     -11.682      -6.810
C(fips_code)[T.34023]                       -9.4659      1.238     -7.646      0.000     -11.893      -7.039
C(fips_code)[T.34025]                       -5.8237      1.237     -4.707      0.000      -8.249      -3.398
C(fips_code)[T.34027]                      -11.1104      1.240     -8.962      0.000     -13.540      -8.680
C(fips_code)[T.34029]                        0.6647      1.237      0.537      0.591      -1.760       3.089
C(fips_code)[T.34031]                       -3.0562      1.244     -2.456      0.014      -5.495      -0.617
C(fips_code)[T.34033]                        9.9406      1.238      8.029      0.000       7.514      12.367
C(fips_code)[T.34035]                      -11.7603      1.240     -9.482      0.000     -14.191      -9.329
C(fips_code)[T.34037]                        7.5124      1.238      6.070      0.000       5.087       9.938
C(fips_code)[T.34039]                       -0.9767      1.241     -0.787      0.431      -3.410       1.457
C(fips_code)[T.34041]                       12.7721      1.238     10.313      0.000      10.345      15.200
C(fips_code)[T.35001]                       -6.9964      1.226     -5.707      0.000      -9.400      -4.593
C(fips_code)[T.35005]                        0.9608      1.226      0.784      0.433      -1.442       3.364
C(fips_code)[T.35009]                        2.0179      1.229      1.642      0.101      -0.390       4.426
C(fips_code)[T.35013]                       -3.4821      1.226     -2.839      0.005      -5.886      -1.078
C(fips_code)[T.35015]                       -5.9002      1.229     -4.801      0.000      -8.309      -3.491
C(fips_code)[T.35025]                       -5.8286      1.226     -4.754      0.000      -8.232      -3.425
C(fips_code)[T.35031]                       -4.0782      1.245     -3.275      0.001      -6.519      -1.637
C(fips_code)[T.35035]                        3.2620      1.226      2.660      0.008       0.858       5.666
C(fips_code)[T.35039]                       16.4572      1.522     10.811      0.000      13.473      19.441
C(fips_code)[T.35043]                        6.7466      1.226      5.501      0.000       4.343       9.150
C(fips_code)[T.35045]                        4.0828      1.232      3.315      0.001       1.669       6.497
C(fips_code)[T.35049]                      -11.7003      1.226     -9.542      0.000     -14.104      -9.297
C(fips_code)[T.35061]                        5.0501      1.226      4.119      0.000       2.647       7.453
C(fips_code)[T.36001]                      -17.1673      1.256    -13.673      0.000     -19.628     -14.706
C(fips_code)[T.36003]                       -1.6945      1.563     -1.084      0.278      -4.758       1.369
C(fips_code)[T.36007]                       -1.0246      1.255     -0.817      0.414      -3.484       1.434
C(fips_code)[T.36009]                        7.2411      1.257      5.761      0.000       4.777       9.705
C(fips_code)[T.36011]                        6.3903      1.255      5.091      0.000       3.930       8.851
C(fips_code)[T.36013]                        9.1709      1.255      7.306      0.000       6.710      11.631
C(fips_code)[T.36015]                       -4.0972      1.255     -3.264      0.001      -6.558      -1.636
C(fips_code)[T.36017]                        5.0938      1.256      4.055      0.000       2.631       7.556
C(fips_code)[T.36019]                       -0.8391      1.255     -0.669      0.504      -3.299       1.621
C(fips_code)[T.36021]                       -5.1717      1.257     -4.114      0.000      -7.636      -2.708
C(fips_code)[T.36023]                        2.9055      1.255      2.315      0.021       0.445       5.366
C(fips_code)[T.36025]                       -4.2160      2.343     -1.799      0.072      -8.809       0.377
C(fips_code)[T.36027]                      -10.1831      1.255     -8.115      0.000     -12.643      -7.724
C(fips_code)[T.36029]                       -7.0558      1.256     -5.616      0.000      -9.519      -4.593
C(fips_code)[T.36033]                        0.8405      1.255      0.670      0.503      -1.620       3.301
C(fips_code)[T.36035]                        8.9097      1.254      7.103      0.000       6.451      11.369
C(fips_code)[T.36037]                        4.1159      1.255      3.280      0.001       1.656       6.576
C(fips_code)[T.36039]                       13.1788      1.301     10.127      0.000      10.628      15.730
C(fips_code)[T.36043]                       12.6351      1.255     10.068      0.000      10.175      15.095
C(fips_code)[T.36045]                        5.5421      1.256      4.411      0.000       3.079       8.005
C(fips_code)[T.36051]                        3.8101      1.255      3.036      0.002       1.350       6.270
C(fips_code)[T.36053]                        9.4705      1.255      7.548      0.000       7.011      11.930
C(fips_code)[T.36055]                       -7.6193      1.254     -6.074      0.000     -10.078      -5.161
C(fips_code)[T.36057]                        6.5897      1.255      5.251      0.000       4.130       9.050
C(fips_code)[T.36059]                      -13.1025      1.265    -10.359      0.000     -15.582     -10.623
C(fips_code)[T.36061]                      -39.5599      1.281    -30.884      0.000     -42.071     -37.049
C(fips_code)[T.36063]                        4.3811      1.261      3.473      0.001       1.909       6.854
C(fips_code)[T.36065]                       -1.3660      1.254     -1.089      0.276      -3.825       1.093
C(fips_code)[T.36067]                       -7.0613      1.254     -5.629      0.000      -9.520      -4.602
C(fips_code)[T.36069]                       -4.5161      1.255     -3.599      0.000      -6.976      -2.056
C(fips_code)[T.36071]                       -5.2257      1.261     -4.145      0.000      -7.697      -2.754
C(fips_code)[T.36073]                       17.1622      1.257     13.657      0.000      14.699      19.625
C(fips_code)[T.36075]                       15.9009      1.256     12.659      0.000      13.439      18.363
C(fips_code)[T.36077]                        1.4968      1.256      1.192      0.233      -0.965       3.959
C(fips_code)[T.36079]                      -11.4089      1.255     -9.092      0.000     -13.868      -8.949
C(fips_code)[T.36083]                       -1.4176      1.256     -1.129      0.259      -3.879       1.043
C(fips_code)[T.36087]                      -13.1092      1.276    -10.272      0.000     -15.611     -10.608
C(fips_code)[T.36089]                        4.7220      1.255      3.762      0.000       2.262       7.182
C(fips_code)[T.36091]                       -8.6988      1.256     -6.928      0.000     -11.160      -6.238
C(fips_code)[T.36093]                       -0.9152      1.255     -0.729      0.466      -3.374       1.544
C(fips_code)[T.36099]                        7.0960      1.772      4.005      0.000       3.623      10.569
C(fips_code)[T.36101]                        3.5127      1.255      2.800      0.005       1.054       5.972
C(fips_code)[T.36103]                       -6.1517      1.262     -4.876      0.000      -8.625      -3.679
C(fips_code)[T.36105]                       12.6069      1.256     10.038      0.000      10.145      15.069
C(fips_code)[T.36107]                        4.9628      1.563      3.176      0.001       1.899       8.026
C(fips_code)[T.36109]                      -17.3435      1.259    -13.781      0.000     -19.810     -14.877
C(fips_code)[T.36111]                       -2.0109      1.254     -1.603      0.109      -4.469       0.448
C(fips_code)[T.36113]                        2.6473      1.255      2.109      0.035       0.187       5.108
C(fips_code)[T.36115]                       11.0532      1.563      7.072      0.000       7.990      14.117
C(fips_code)[T.36117]                       10.9169      1.255      8.698      0.000       8.457      13.377
C(fips_code)[T.36119]                      -13.9838      1.266    -11.046      0.000     -16.465     -11.502
C(fips_code)[T.36121]                       11.9593      1.658      7.215      0.000       8.710      15.208
C(fips_code)[T.37001]                       -5.9759      1.256     -4.758      0.000      -8.438      -3.514
C(fips_code)[T.37003]                        3.4368      1.258      2.731      0.006       0.970       5.903
C(fips_code)[T.37013]                        2.1803      1.258      1.733      0.083      -0.286       4.647
C(fips_code)[T.37017]                        4.2983      1.918      2.241      0.025       0.539       8.057
C(fips_code)[T.37019]                        3.5548      1.259      2.824      0.005       1.087       6.022
C(fips_code)[T.37021]                      -15.3966      1.259    -12.228      0.000     -17.865     -12.929
C(fips_code)[T.37023]                        5.1790      1.257      4.122      0.000       2.716       7.642
C(fips_code)[T.37025]                       -7.8239      1.256     -6.229      0.000     -10.286      -5.362
C(fips_code)[T.37027]                        9.9944      1.257      7.951      0.000       7.530      12.458
C(fips_code)[T.37031]                        7.6968      1.257      6.125      0.000       5.234      10.160
C(fips_code)[T.37035]                       -4.5739      1.259     -3.633      0.000      -7.042      -2.106
C(fips_code)[T.37037]                       -1.0503      1.259     -0.834      0.404      -3.519       1.418
C(fips_code)[T.37045]                        5.4588      1.256      4.346      0.000       2.997       7.921
C(fips_code)[T.37047]                        9.2180      1.257      7.334      0.000       6.754      11.682
C(fips_code)[T.37049]                       -5.6381      1.257     -4.487      0.000      -8.101      -3.175
C(fips_code)[T.37051]                       -0.6181      1.258     -0.491      0.623      -3.084       1.848
C(fips_code)[T.37055]                       29.4065      1.494     19.688      0.000      26.479      32.334
C(fips_code)[T.37057]                        2.5536      1.256      2.033      0.042       0.092       5.016
C(fips_code)[T.37059]                        3.1311      1.256      2.493      0.013       0.669       5.593
C(fips_code)[T.37061]                       -1.6903      1.262     -1.340      0.180      -4.163       0.783
C(fips_code)[T.37063]                      -18.6236      1.257    -14.817      0.000     -21.087     -16.160
C(fips_code)[T.37065]                       10.3858      1.257      8.260      0.000       7.921      12.851
C(fips_code)[T.37067]                       -6.3933      1.256     -5.090      0.000      -8.855      -3.931
C(fips_code)[T.37069]                        2.6489      1.257      2.108      0.035       0.186       5.112
C(fips_code)[T.37071]                        2.4256      1.256      1.931      0.054      -0.037       4.888
C(fips_code)[T.37077]                        3.0825      1.258      2.450      0.014       0.616       5.548
C(fips_code)[T.37081]                       -8.1918      1.257     -6.517      0.000     -10.656      -5.728
C(fips_code)[T.37083]                        8.6984      1.256      6.925      0.000       6.236      11.160
C(fips_code)[T.37085]                        4.7582      1.256      3.788      0.000       2.296       7.220
C(fips_code)[T.37087]                        0.7937      1.257      0.632      0.528      -1.670       3.257
C(fips_code)[T.37089]                       -4.7242      1.257     -3.760      0.000      -7.187      -2.261
C(fips_code)[T.37093]                        5.5096      1.566      3.517      0.000       2.439       8.580
C(fips_code)[T.37097]                       -3.7505      1.256     -2.986      0.003      -6.213      -1.288
C(fips_code)[T.37099]                       -5.5850      1.256     -4.446      0.000      -8.047      -3.123
C(fips_code)[T.37101]                       -1.0098      1.257     -0.804      0.422      -3.473       1.453
C(fips_code)[T.37105]                        3.8186      1.257      3.039      0.002       1.355       6.282
C(fips_code)[T.37107]                       -0.4753      1.260     -0.377      0.706      -2.945       1.994
C(fips_code)[T.37109]                        6.3724      1.256      5.073      0.000       3.910       8.835
C(fips_code)[T.37111]                        8.8573      1.256      7.051      0.000       6.395      11.320
C(fips_code)[T.37113]                       -1.6058      1.256     -1.278      0.201      -4.068       0.857
C(fips_code)[T.37119]                      -16.4786      1.256    -13.119      0.000     -18.941     -14.017
C(fips_code)[T.37125]                      -10.8038      1.256     -8.602      0.000     -13.266      -8.342
C(fips_code)[T.37127]                        0.3892      1.256      0.310      0.757      -2.073       2.851
C(fips_code)[T.37129]                      -11.4058      1.256     -9.078      0.000     -13.869      -8.943
C(fips_code)[T.37133]                       -0.1459      1.256     -0.116      0.908      -2.608       2.316
C(fips_code)[T.37135]                      -24.3047      1.259    -19.300      0.000     -26.773     -21.836
C(fips_code)[T.37139]                        2.5935      1.257      2.064      0.039       0.130       5.057
C(fips_code)[T.37141]                        8.0299      1.256      6.393      0.000       5.568      10.492
C(fips_code)[T.37145]                        2.2406      1.256      1.784      0.074      -0.222       4.703
C(fips_code)[T.37147]                       -7.9936      1.257     -6.359      0.000     -10.458      -5.530
C(fips_code)[T.37151]                       -1.0369      1.256     -0.825      0.409      -3.499       1.426
C(fips_code)[T.37153]                       11.1522      1.256      8.879      0.000       8.690      13.614
C(fips_code)[T.37155]                        5.2215      1.256      4.157      0.000       2.759       7.684
C(fips_code)[T.37157]                        6.2520      1.257      4.973      0.000       3.788       8.716
C(fips_code)[T.37159]                       -0.7549      1.257     -0.601      0.548      -3.218       1.709
C(fips_code)[T.37161]                        3.8381      1.257      3.053      0.002       1.374       6.302
C(fips_code)[T.37163]                        2.4735      1.260      1.963      0.050       0.003       4.944
C(fips_code)[T.37165]                       13.9789      1.259     11.100      0.000      11.511      16.447
C(fips_code)[T.37167]                        5.7651      1.257      4.586      0.000       3.301       8.229
C(fips_code)[T.37169]                       -3.6143      1.257     -2.876      0.004      -6.077      -1.151
C(fips_code)[T.37171]                        2.9540      1.256      2.352      0.019       0.492       5.416
C(fips_code)[T.37179]                       -4.2152      1.257     -3.353      0.001      -6.679      -1.751
C(fips_code)[T.37181]                        3.9392      1.258      3.132      0.002       1.474       6.404
C(fips_code)[T.37183]                      -17.5502      1.256    -13.968      0.000     -20.013     -15.087
C(fips_code)[T.37189]                      -21.0920      1.417    -14.883      0.000     -23.870     -18.314
C(fips_code)[T.37191]                       -0.4306      1.257     -0.342      0.732      -2.895       2.034
C(fips_code)[T.37193]                       -2.4543      1.256     -1.953      0.051      -4.917       0.008
C(fips_code)[T.37195]                        1.8234      1.256      1.451      0.147      -0.639       4.286
C(fips_code)[T.37197]                       -0.1925      1.661     -0.116      0.908      -3.448       3.063
C(fips_code)[T.39003]                       -1.0601      1.258     -0.843      0.399      -3.526       1.406
C(fips_code)[T.39005]                        2.1823      1.260      1.732      0.083      -0.287       4.652
C(fips_code)[T.39007]                        7.2532      1.257      5.769      0.000       4.789       9.717
C(fips_code)[T.39009]                      -17.4796      1.260    -13.868      0.000     -19.950     -15.009
C(fips_code)[T.39011]                        3.1352      1.257      2.494      0.013       0.672       5.599
C(fips_code)[T.39013]                       -3.2511      1.258     -2.584      0.010      -5.718      -0.785
C(fips_code)[T.39015]                        6.7053      1.417      4.730      0.000       3.927       9.484
C(fips_code)[T.39017]                       -8.6637      1.258     -6.888      0.000     -11.129      -6.198
C(fips_code)[T.39021]                        3.4960      1.257      2.781      0.005       1.032       5.960
C(fips_code)[T.39023]                        4.6504      1.257      3.700      0.000       2.187       7.114
C(fips_code)[T.39025]                       -5.5054      1.258     -4.375      0.000      -7.972      -3.039
C(fips_code)[T.39027]                       -3.9003      1.257     -3.103      0.002      -6.364      -1.437
C(fips_code)[T.39029]                        5.0382      1.259      4.000      0.000       2.569       7.507
C(fips_code)[T.39031]                        5.4629      1.257      4.346      0.000       2.999       7.927
C(fips_code)[T.39033]                        6.8235      1.263      5.403      0.000       4.348       9.299
C(fips_code)[T.39035]                      -10.3358      1.263     -8.181      0.000     -12.812      -7.859
C(fips_code)[T.39037]                        7.2525      1.258      5.767      0.000       4.788       9.717
C(fips_code)[T.39039]                        3.4534      1.259      2.743      0.006       0.986       5.921
C(fips_code)[T.39041]                      -18.9943      1.264    -15.033      0.000     -21.471     -16.518
C(fips_code)[T.39043]                        1.4704      1.267      1.160      0.246      -1.014       3.954
C(fips_code)[T.39045]                       -7.1619      1.259     -5.689      0.000      -9.629      -4.694
C(fips_code)[T.39047]                        5.8999      2.102      2.807      0.005       1.780      10.019
C(fips_code)[T.39049]                      -13.8099      1.258    -10.975      0.000     -16.276     -11.343
C(fips_code)[T.39051]                        0.7149      1.258      0.568      0.570      -1.750       3.180
C(fips_code)[T.39055]                       -2.3551      1.257     -1.874      0.061      -4.819       0.109
C(fips_code)[T.39057]                      -13.9102      1.260    -11.039      0.000     -16.380     -11.440
C(fips_code)[T.39059]                        4.0985      1.257      3.261      0.001       1.635       6.562
C(fips_code)[T.39061]                      -12.1259      1.258     -9.642      0.000     -14.591      -9.661
C(fips_code)[T.39063]                       -5.4657      1.257     -4.348      0.000      -7.930      -3.002
C(fips_code)[T.39065]                        5.2601      1.776      2.961      0.003       1.778       8.742
C(fips_code)[T.39069]                       -1.9526      1.777     -1.099      0.272      -5.435       1.530
C(fips_code)[T.39071]                       14.5442      1.258     11.562      0.000      12.078      17.010
C(fips_code)[T.39077]                        1.4941      1.260      1.186      0.236      -0.976       3.964
C(fips_code)[T.39081]                        3.5969      1.258      2.859      0.004       1.131       6.063
C(fips_code)[T.39083]                        2.8229      1.260      2.240      0.025       0.352       5.294
C(fips_code)[T.39085]                       -6.7069      1.259     -5.327      0.000      -9.175      -4.239
C(fips_code)[T.39087]                       11.9671      1.257      9.521      0.000       9.503      14.431
C(fips_code)[T.39089]                       -2.4698      1.259     -1.961      0.050      -4.938      -0.002
C(fips_code)[T.39091]                       14.2030      1.262     11.252      0.000      11.729      16.677
C(fips_code)[T.39093]                        1.8895      1.267      1.492      0.136      -0.593       4.372
C(fips_code)[T.39095]                       -5.4833      1.264     -4.337      0.000      -7.961      -3.005
C(fips_code)[T.39097]                       -7.4244      1.260     -5.891      0.000      -9.895      -4.954
C(fips_code)[T.39099]                       -1.9559      1.262     -1.550      0.121      -4.429       0.517
C(fips_code)[T.39101]                        5.3883      1.274      4.230      0.000       2.891       7.885
C(fips_code)[T.39103]                       -4.3257      1.258     -3.439      0.001      -6.791      -1.860
C(fips_code)[T.39107]                       -3.0494      1.260     -2.420      0.016      -5.520      -0.579
C(fips_code)[T.39109]                        0.2050      1.258      0.163      0.870      -2.260       2.670
C(fips_code)[T.39113]                       -7.8678      1.257     -6.260      0.000     -10.331      -5.404
C(fips_code)[T.39117]                        5.3109      2.102      2.527      0.012       1.191       9.431
C(fips_code)[T.39119]                        2.3681      1.257      1.884      0.060      -0.096       4.832
C(fips_code)[T.39123]                       28.1250      1.259     22.344      0.000      25.658      30.592
C(fips_code)[T.39129]                        8.5275      1.271      6.707      0.000       6.035      11.020
C(fips_code)[T.39133]                       -4.8201      1.258     -3.832      0.000      -7.286      -2.354
C(fips_code)[T.39135]                        7.6034      1.258      6.045      0.000       5.138      10.069
C(fips_code)[T.39137]                       -6.1069      1.777     -3.436      0.001      -9.591      -2.623
C(fips_code)[T.39139]                       -2.2471      1.258     -1.786      0.074      -4.713       0.219
C(fips_code)[T.39141]                        1.9928      1.257      1.585      0.113      -0.471       4.457
C(fips_code)[T.39143]                        6.7719      1.258      5.384      0.000       4.307       9.237
C(fips_code)[T.39145]                        6.3277      1.257      5.034      0.000       3.864       8.792
C(fips_code)[T.39147]                        6.8061      1.258      5.410      0.000       4.340       9.272
C(fips_code)[T.39149]                       11.7600      1.257      9.354      0.000       9.296      14.224
C(fips_code)[T.39151]                       -4.8763      1.257     -3.879      0.000      -7.340      -2.413
C(fips_code)[T.39153]                       -8.2799      1.257     -6.587      0.000     -10.744      -5.816
C(fips_code)[T.39155]                        1.0893      1.260      0.864      0.387      -1.381       3.560
C(fips_code)[T.39157]                       -0.8503      1.257     -0.676      0.499      -3.315       1.614
C(fips_code)[T.39159]                       -0.7628      1.259     -0.606      0.545      -3.231       1.705
C(fips_code)[T.39161]                        1.6612      1.257      1.321      0.186      -0.803       4.126
C(fips_code)[T.39165]                      -10.4646      1.259     -8.309      0.000     -12.933      -7.996
C(fips_code)[T.39167]                       -1.6446      1.257     -1.308      0.191      -4.109       0.819
C(fips_code)[T.39169]                      -10.0806      1.262     -7.986      0.000     -12.555      -7.606
C(fips_code)[T.39171]                        7.5120      1.258      5.973      0.000       5.047       9.977
C(fips_code)[T.39173]                       -7.7021      1.257     -6.126      0.000     -10.166      -5.238
C(fips_code)[T.41003]                      -21.1754      1.242    -17.056      0.000     -23.609     -18.742
C(fips_code)[T.41005]                       -8.8147      1.237     -7.126      0.000     -11.239      -6.390
C(fips_code)[T.41007]                        5.1601      1.249      4.131      0.000       2.712       7.609
C(fips_code)[T.41009]                        4.4993      1.237      3.639      0.000       2.076       6.923
C(fips_code)[T.41011]                        7.1971      1.239      5.811      0.000       4.769       9.625
C(fips_code)[T.41017]                       -3.4202      1.237     -2.764      0.006      -5.845      -0.995
C(fips_code)[T.41019]                        8.8432      1.237      7.151      0.000       6.419      11.267
C(fips_code)[T.41029]                       -1.5354      1.237     -1.242      0.214      -3.959       0.888
C(fips_code)[T.41033]                        7.2799      1.237      5.887      0.000       4.856       9.704
C(fips_code)[T.41035]                        8.5059      1.237      6.877      0.000       6.081      10.930
C(fips_code)[T.41039]                       -5.7534      1.237     -4.653      0.000      -8.177      -3.330
C(fips_code)[T.41041]                        4.3758      1.254      3.490      0.000       1.918       6.834
C(fips_code)[T.41043]                        5.0334      1.237      4.070      0.000       2.610       7.457
C(fips_code)[T.41045]                       11.5997      1.544      7.512      0.000       8.573      14.627
C(fips_code)[T.41047]                       -4.8264      1.237     -3.901      0.000      -7.252      -2.401
C(fips_code)[T.41051]                      -20.2143      1.237    -16.346      0.000     -22.638     -17.790
C(fips_code)[T.41053]                        2.2907      1.238      1.850      0.064      -0.136       4.717
C(fips_code)[T.41059]                       10.1115      1.237      8.173      0.000       7.686      12.536
C(fips_code)[T.41067]                      -13.3732      1.238    -10.799      0.000     -15.801     -10.946
C(fips_code)[T.41071]                        1.7649      1.237      1.426      0.154      -0.660       4.190
C(fips_code)[T.42001]                       -8.6101      1.255     -6.859      0.000     -11.071      -6.150
C(fips_code)[T.42003]                      -13.7243      1.254    -10.944      0.000     -16.182     -11.266
C(fips_code)[T.42005]                        5.7793      1.255      4.605      0.000       3.319       8.239
C(fips_code)[T.42007]                       -1.2348      1.256     -0.983      0.325      -3.696       1.226
C(fips_code)[T.42009]                        8.9384      1.255      7.122      0.000       6.478      11.398
C(fips_code)[T.42011]                       -1.8682      1.260     -1.483      0.138      -4.337       0.601
C(fips_code)[T.42013]                       -0.1403      1.255     -0.112      0.911      -2.600       2.320
C(fips_code)[T.42015]                        7.6758      1.260      6.091      0.000       5.206      10.146
C(fips_code)[T.42017]                       -7.0310      1.260     -5.581      0.000      -9.500      -4.561
C(fips_code)[T.42019]                       -5.4874      1.254     -4.375      0.000      -7.946      -3.029
C(fips_code)[T.42021]                        0.1731      1.255      0.138      0.890      -2.287       2.634
C(fips_code)[T.42025]                        6.6814      1.255      5.323      0.000       4.221       9.142
C(fips_code)[T.42027]                      -24.9666      1.264    -19.751      0.000     -27.444     -22.489
C(fips_code)[T.42029]                      -13.7902      1.262    -10.928      0.000     -16.264     -11.317
C(fips_code)[T.42033]                        7.4503      1.259      5.919      0.000       4.983       9.918
C(fips_code)[T.42035]                        7.8239      1.256      6.228      0.000       5.361      10.286
C(fips_code)[T.42037]                        0.0454      1.254      0.036      0.971      -2.413       2.504
C(fips_code)[T.42039]                        9.7367      1.259      7.735      0.000       7.269      12.204
C(fips_code)[T.42041]                      -11.4628      1.257     -9.118      0.000     -13.927      -8.999
C(fips_code)[T.42043]                      -12.1598      1.255     -9.693      0.000     -14.619      -9.701
C(fips_code)[T.42045]                       -5.1151      1.261     -4.055      0.000      -7.588      -2.643
C(fips_code)[T.42049]                       -3.9159      1.255     -3.120      0.002      -6.376      -1.456
C(fips_code)[T.42051]                        6.9261      1.259      5.500      0.000       4.458       9.394
C(fips_code)[T.42055]                       -0.7376      1.256     -0.587      0.557      -3.199       1.723
C(fips_code)[T.42061]                        7.6443      1.255      6.090      0.000       5.184      10.105
C(fips_code)[T.42063]                        0.3712      1.255      0.296      0.767      -2.088       2.831
C(fips_code)[T.42065]                        7.0025      1.259      5.563      0.000       4.535       9.470
C(fips_code)[T.42069]                       -3.6266      1.261     -2.877      0.004      -6.098      -1.156
C(fips_code)[T.42071]                       -8.6551      1.259     -6.873      0.000     -11.123      -6.187
C(fips_code)[T.42073]                        6.8358      1.260      5.426      0.000       4.366       9.305
C(fips_code)[T.42075]                       -1.4779      1.255     -1.177      0.239      -3.938       0.983
C(fips_code)[T.42077]                       -9.1412      1.259     -7.259      0.000     -11.610      -6.673
C(fips_code)[T.42079]                       -2.0792      1.259     -1.652      0.099      -4.546       0.388
C(fips_code)[T.42081]                       -3.4584      1.256     -2.753      0.006      -5.921      -0.996
C(fips_code)[T.42083]                        5.3580      1.260      4.252      0.000       2.888       7.828
C(fips_code)[T.42085]                       -0.1218      1.257     -0.097      0.923      -2.585       2.342
C(fips_code)[T.42087]                        7.6199      1.254      6.075      0.000       5.161      10.079
C(fips_code)[T.42089]                       -2.7039      1.262     -2.143      0.032      -5.177      -0.231
C(fips_code)[T.42091]                      -15.4716      1.261    -12.271      0.000     -17.943     -13.000
C(fips_code)[T.42095]                       -8.4002      1.259     -6.670      0.000     -10.869      -5.932
C(fips_code)[T.42097]                        4.5525      1.257      3.623      0.000       2.089       7.016
C(fips_code)[T.42099]                        9.1048      1.659      5.487      0.000       5.852      12.357
C(fips_code)[T.42101]                      -11.2225      1.263     -8.889      0.000     -13.697      -8.748
C(fips_code)[T.42103]                       11.1935      1.259      8.892      0.000       8.726      13.661
C(fips_code)[T.42107]                        7.7475      1.254      6.177      0.000       5.289      10.206
C(fips_code)[T.42109]                        3.3026      1.572      2.101      0.036       0.222       6.383
C(fips_code)[T.42111]                        3.0980      1.255      2.468      0.014       0.637       5.559
C(fips_code)[T.42115]                        6.0678      1.659      3.658      0.000       2.816       9.319
C(fips_code)[T.42117]                        5.7037      1.259      4.532      0.000       3.237       8.171
C(fips_code)[T.42121]                       15.0102      1.259     11.925      0.000      12.543      17.477
C(fips_code)[T.42123]                        9.4999      1.260      7.542      0.000       7.031      11.969
C(fips_code)[T.42125]                       -5.6652      1.254     -4.516      0.000      -8.124      -3.206
C(fips_code)[T.42127]                       13.2157      1.255     10.530      0.000      10.756      15.676
C(fips_code)[T.42129]                        0.5884      1.254      0.469      0.639      -1.870       3.047
C(fips_code)[T.42133]                       -2.3848      1.255     -1.900      0.057      -4.844       0.075
C(fips_code)[T.44001]                       -1.0645      1.455     -0.732      0.464      -3.917       1.788
C(fips_code)[T.44003]                       -1.3052      1.211     -1.078      0.281      -3.679       1.068
C(fips_code)[T.44005]                        0.6675      0.966      0.691      0.489      -1.225       2.560
C(fips_code)[T.44007]                       -2.3440      1.214     -1.931      0.053      -4.723       0.035
C(fips_code)[T.44009]                        1.0910      1.211      0.901      0.367      -1.282       3.464
C(fips_code)[T.45003]                       -0.1545      1.244     -0.124      0.901      -2.593       2.284
C(fips_code)[T.45007]                       -4.1539      1.243     -3.341      0.001      -6.591      -1.717
C(fips_code)[T.45013]                       -3.1687      1.243     -2.549      0.011      -5.606      -0.732
C(fips_code)[T.45015]                       -2.6851      1.243     -2.159      0.031      -5.122      -0.248
C(fips_code)[T.45019]                      -12.5739      1.243    -10.113      0.000     -15.011     -10.137
C(fips_code)[T.45021]                        3.5747      1.246      2.869      0.004       1.132       6.017
C(fips_code)[T.45023]                        2.3955      1.246      1.922      0.055      -0.047       4.838
C(fips_code)[T.45025]                        4.0325      1.244      3.243      0.001       1.595       6.470
C(fips_code)[T.45029]                        3.3268      1.245      2.672      0.008       0.887       5.767
C(fips_code)[T.45031]                        5.7007      1.244      4.584      0.000       3.263       8.138
C(fips_code)[T.45035]                       -1.5163      1.243     -1.220      0.223      -3.953       0.921
C(fips_code)[T.45041]                       -3.1800      1.245     -2.554      0.011      -5.620      -0.740
C(fips_code)[T.45043]                        3.6370      1.244      2.923      0.003       1.198       6.076
C(fips_code)[T.45045]                       -7.8168      1.243     -6.288      0.000     -10.254      -5.380
C(fips_code)[T.45047]                        0.7052      1.243      0.567      0.571      -1.732       3.142
C(fips_code)[T.45051]                        4.8961      1.255      3.900      0.000       2.435       7.357
C(fips_code)[T.45055]                       -3.5584      1.244     -2.860      0.004      -5.997      -1.119
C(fips_code)[T.45057]                        2.5112      1.244      2.019      0.043       0.074       4.949
C(fips_code)[T.45059]                       11.4370      1.244      9.193      0.000       8.998      13.876
C(fips_code)[T.45063]                       -7.5481      1.246     -6.055      0.000      -9.991      -5.105
C(fips_code)[T.45071]                        0.4896      1.245      0.393      0.694      -1.951       2.930
C(fips_code)[T.45073]                        6.7186      1.243      5.404      0.000       4.282       9.156
C(fips_code)[T.45075]                        3.8874      1.245      3.123      0.002       1.448       6.327
C(fips_code)[T.45077]                       -0.0194      1.243     -0.016      0.988      -2.457       2.418
C(fips_code)[T.45079]                       -9.8345      1.244     -7.904      0.000     -12.273      -7.396
C(fips_code)[T.45083]                        1.0754      1.244      0.864      0.387      -1.363       3.514
C(fips_code)[T.45085]                        0.6169      1.243      0.496      0.620      -1.820       3.054
C(fips_code)[T.45091]                       -2.2968      1.243     -1.847      0.065      -4.734       0.140
C(fips_code)[T.47001]                       -5.6218      1.253     -4.487      0.000      -8.078      -3.166
C(fips_code)[T.47003]                        7.0664      1.254      5.636      0.000       4.609       9.524
C(fips_code)[T.47009]                       -5.3067      1.253     -4.235      0.000      -7.763      -2.850
C(fips_code)[T.47011]                       -3.8555      1.253     -3.076      0.002      -6.312      -1.399
C(fips_code)[T.47013]                       11.2949      1.253      9.017      0.000       8.840      13.750
C(fips_code)[T.47019]                        9.6377      1.254      7.687      0.000       7.180      12.095
C(fips_code)[T.47021]                       -8.6554      1.255     -6.895      0.000     -11.116      -6.195
C(fips_code)[T.47029]                       14.3321      1.262     11.360      0.000      11.859      16.805
C(fips_code)[T.47031]                        3.1117      1.254      2.481      0.013       0.653       5.570
C(fips_code)[T.47035]                        2.8308      1.253      2.259      0.024       0.374       5.287
C(fips_code)[T.47037]                      -19.8205      1.253    -15.813      0.000     -22.277     -17.364
C(fips_code)[T.47043]                       -2.1684      1.254     -1.730      0.084      -4.626       0.289
C(fips_code)[T.47045]                        6.6422      1.253      5.303      0.000       4.187       9.097
C(fips_code)[T.47047]                       -1.1321      1.912     -0.592      0.554      -4.880       2.615
C(fips_code)[T.47051]                       12.8841      1.255     10.263      0.000      10.423      15.345
C(fips_code)[T.47053]                        3.8703      1.254      3.087      0.002       1.413       6.328
C(fips_code)[T.47059]                        3.6744      1.253      2.933      0.003       1.219       6.130
C(fips_code)[T.47063]                       -2.7054      1.253     -2.160      0.031      -5.161      -0.250
C(fips_code)[T.47065]                      -13.2202      1.253    -10.552      0.000     -15.676     -10.764
C(fips_code)[T.47073]                        9.0385      1.253      7.216      0.000       6.583      11.494
C(fips_code)[T.47079]                        4.1905      1.253      3.345      0.001       1.735       6.646
C(fips_code)[T.47089]                        6.1899      1.253      4.941      0.000       3.734       8.646
C(fips_code)[T.47093]                      -13.8516      1.254    -11.043      0.000     -16.310     -11.393
C(fips_code)[T.47099]                        5.2897      1.253      4.221      0.000       2.833       7.746
C(fips_code)[T.47103]                        1.8429      1.770      1.041      0.298      -1.627       5.313
C(fips_code)[T.47105]                       -1.9929      1.253     -1.591      0.112      -4.449       0.463
C(fips_code)[T.47107]                        2.1056      1.253      1.681      0.093      -0.350       4.561
C(fips_code)[T.47113]                       -5.9566      1.253     -4.755      0.000      -8.412      -3.501
C(fips_code)[T.47117]                       11.3708      1.358      8.374      0.000       8.709      14.032
C(fips_code)[T.47119]                       -3.7744      1.256     -3.005      0.003      -6.237      -1.312
C(fips_code)[T.47123]                       10.6195      1.255      8.465      0.000       8.160      13.079
C(fips_code)[T.47125]                       -3.5215      1.253     -2.811      0.005      -5.977      -1.066
C(fips_code)[T.47131]                       -2.7526      1.562     -1.762      0.078      -5.815       0.310
C(fips_code)[T.47141]                       -4.0657      1.253     -3.244      0.001      -6.522      -1.609
C(fips_code)[T.47143]                       10.4792      1.483      7.066      0.000       7.572      13.386
C(fips_code)[T.47145]                        5.5591      1.255      4.431      0.000       3.100       8.018
C(fips_code)[T.47147]                        1.1327      1.254      0.903      0.367      -1.326       3.591
C(fips_code)[T.47149]                       -8.3873      1.253     -6.696      0.000     -10.843      -5.932
C(fips_code)[T.47155]                        5.4219      1.268      4.276      0.000       2.936       7.908
C(fips_code)[T.47157]                      -10.4920      1.253     -8.375      0.000     -12.948      -8.036
C(fips_code)[T.47163]                       -1.0002      1.253     -0.798      0.425      -3.456       1.456
C(fips_code)[T.47165]                       -7.2738      1.253     -5.805      0.000      -9.730      -4.818
C(fips_code)[T.47167]                        6.6626      1.255      5.309      0.000       4.203       9.122
C(fips_code)[T.47177]                       13.4315      1.262     10.640      0.000      10.957      15.906
C(fips_code)[T.47179]                      -10.8377      1.254     -8.645      0.000     -13.295      -8.380
C(fips_code)[T.47187]                      -23.0341      1.257    -18.324      0.000     -25.498     -20.570
C(fips_code)[T.47189]                       -8.4089      1.253     -6.710      0.000     -10.865      -5.953
C(fips_code)[T.48001]                        1.6664      1.262      1.320      0.187      -0.808       4.140
C(fips_code)[T.48005]                        0.7990      1.258      0.635      0.526      -1.668       3.266
C(fips_code)[T.48007]                        9.5647      1.664      5.747      0.000       6.302      12.827
C(fips_code)[T.48013]                        4.7778      1.259      3.796      0.000       2.310       7.245
C(fips_code)[T.48021]                        2.3421      1.259      1.860      0.063      -0.126       4.811
C(fips_code)[T.48027]                        0.0442      1.259      0.035      0.972      -2.424       2.512
C(fips_code)[T.48029]                       -9.8147      1.259     -7.796      0.000     -12.282      -7.347
C(fips_code)[T.48037]                        2.4466      1.258      1.944      0.052      -0.020       4.913
C(fips_code)[T.48039]                        1.3199      1.259      1.048      0.294      -1.148       3.787
C(fips_code)[T.48041]                      -13.8919      1.262    -11.010      0.000     -16.365     -11.419
C(fips_code)[T.48049]                        6.9292      1.259      5.502      0.000       4.461       9.398
C(fips_code)[T.48053]                        3.6189      1.262      2.867      0.004       1.145       6.093
C(fips_code)[T.48055]                       -2.2805      1.259     -1.811      0.070      -4.749       0.188
C(fips_code)[T.48061]                      -11.2514      1.264     -8.905      0.000     -13.728      -8.775
C(fips_code)[T.48067]                        3.9765      2.353      1.690      0.091      -0.636       8.590
C(fips_code)[T.48071]                        1.3971      1.260      1.109      0.268      -1.073       3.867
C(fips_code)[T.48073]                        9.3021      1.259      7.391      0.000       6.835      11.769
C(fips_code)[T.48085]                      -14.1623      1.259    -11.249      0.000     -16.630     -11.695
C(fips_code)[T.48091]                       -4.8038      1.259     -3.817      0.000      -7.271      -2.337
C(fips_code)[T.48097]                        7.0376      1.258      5.592      0.000       4.571       9.504
C(fips_code)[T.48099]                        2.4518      1.259      1.947      0.052      -0.016       4.920
C(fips_code)[T.48113]                      -10.0829      1.259     -8.009      0.000     -12.551      -7.615
C(fips_code)[T.48121]                       -7.9241      1.258     -6.296      0.000     -10.391      -5.457
C(fips_code)[T.48135]                       -4.7395      1.260     -3.762      0.000      -7.209      -2.270
C(fips_code)[T.48139]                       -0.3413      1.259     -0.271      0.786      -2.810       2.127
C(fips_code)[T.48141]                      -10.4234      1.260     -8.272      0.000     -12.893      -7.954
C(fips_code)[T.48143]                       -8.3399      1.421     -5.868      0.000     -11.126      -5.554
C(fips_code)[T.48147]                        5.6636      1.311      4.321      0.000       3.094       8.233
C(fips_code)[T.48157]                       -8.5172      1.259     -6.766      0.000     -10.985      -6.050
C(fips_code)[T.48167]                        5.0957      1.260      4.043      0.000       2.625       7.566
C(fips_code)[T.48181]                        3.0283      1.260      2.404      0.016       0.559       5.497
C(fips_code)[T.48183]                       -0.0191      1.259     -0.015      0.988      -2.486       2.448
C(fips_code)[T.48187]                       -0.0641      1.259     -0.051      0.959      -2.532       2.403
C(fips_code)[T.48189]                        8.3380      1.420      5.873      0.000       5.555      11.121
C(fips_code)[T.48199]                        4.5208      1.261      3.586      0.000       2.050       6.992
C(fips_code)[T.48201]                       -7.1107      1.260     -5.644      0.000      -9.580      -4.641
C(fips_code)[T.48203]                        4.6685      1.259      3.708      0.000       2.201       7.136
C(fips_code)[T.48209]                       -6.7610      1.259     -5.372      0.000      -9.228      -4.294
C(fips_code)[T.48213]                        7.9261      1.259      6.294      0.000       5.458      10.395
C(fips_code)[T.48215]                       -8.4127      1.268     -6.633      0.000     -10.899      -5.927
C(fips_code)[T.48217]                        4.0641      1.259      3.227      0.001       1.596       6.533
C(fips_code)[T.48221]                        3.9332      1.259      3.124      0.002       1.466       6.401
C(fips_code)[T.48223]                        4.0090      1.262      3.177      0.001       1.535       6.483
C(fips_code)[T.48227]                       -5.7713      1.259     -4.585      0.000      -8.239      -3.304
C(fips_code)[T.48231]                        2.2146      1.259      1.759      0.079      -0.253       4.682
C(fips_code)[T.48241]                       14.7433      1.670      8.830      0.000      11.471      18.016
C(fips_code)[T.48245]                       -1.9909      1.270     -1.567      0.117      -4.481       0.499
C(fips_code)[T.48249]                        6.8971      1.314      5.250      0.000       4.322       9.472
C(fips_code)[T.48251]                        4.7588      1.259      3.781      0.000       2.292       7.226
C(fips_code)[T.48257]                        1.3785      1.259      1.095      0.273      -1.089       3.846
C(fips_code)[T.48259]                       -5.9416      1.923     -3.090      0.002      -9.710      -2.173
C(fips_code)[T.48265]                        1.6018      1.259      1.272      0.203      -0.866       4.070
C(fips_code)[T.48273]                       -1.0459      1.489     -0.702      0.482      -3.964       1.873
C(fips_code)[T.48277]                        7.5709      1.260      6.010      0.000       5.102      10.040
C(fips_code)[T.48291]                        5.0859      1.262      4.030      0.000       2.612       7.560
C(fips_code)[T.48303]                       -1.0679      1.260     -0.847      0.397      -3.538       1.402
C(fips_code)[T.48309]                       -5.5306      1.260     -4.391      0.000      -8.000      -3.061
C(fips_code)[T.48321]                        3.2799      1.261      2.601      0.009       0.808       5.752
C(fips_code)[T.48323]                      -10.4152      1.282     -8.121      0.000     -12.929      -7.901
C(fips_code)[T.48325]                       10.6851      1.259      8.486      0.000       8.217      13.153
C(fips_code)[T.48329]                      -10.1776      1.259     -8.085      0.000     -12.645      -7.710
C(fips_code)[T.48339]                       -5.8718      1.258     -4.666      0.000      -8.339      -3.405
C(fips_code)[T.48347]                       -6.4348      1.259     -5.110      0.000      -8.903      -3.966
C(fips_code)[T.48349]                       -2.6573      1.260     -2.110      0.035      -5.126      -0.188
C(fips_code)[T.48355]                        1.9038      1.260      1.511      0.131      -0.566       4.374
C(fips_code)[T.48361]                        9.4182      1.265      7.446      0.000       6.939      11.898
C(fips_code)[T.48363]                        9.5618      1.779      5.374      0.000       6.074      13.049
C(fips_code)[T.48367]                        2.2653      1.260      1.798      0.072      -0.204       4.734
C(fips_code)[T.48373]                       14.2474      1.259     11.319      0.000      11.780      16.715
C(fips_code)[T.48375]                       -2.2061      1.265     -1.744      0.081      -4.685       0.273
C(fips_code)[T.48381]                        3.4117      1.262      2.702      0.007       0.937       5.886
C(fips_code)[T.48397]                       -1.7738      1.259     -1.409      0.159      -4.241       0.694
C(fips_code)[T.48401]                        9.4277      1.259      7.489      0.000       6.960      11.895
C(fips_code)[T.48409]                        5.4151      1.263      4.289      0.000       2.940       7.890
C(fips_code)[T.48423]                       -1.4875      1.259     -1.182      0.237      -3.954       0.979
C(fips_code)[T.48427]                        0.4980      1.310      0.380      0.704      -2.069       3.065
C(fips_code)[T.48439]                       -7.8934      1.259     -6.270      0.000     -10.361      -5.426
C(fips_code)[T.48441]                       -0.9246      1.261     -0.733      0.463      -3.396       1.547
C(fips_code)[T.48449]                        5.6349      1.262      4.465      0.000       3.161       8.109
C(fips_code)[T.48451]                       -6.0079      1.259     -4.771      0.000      -8.476      -3.540
C(fips_code)[T.48453]                      -20.6553      1.259    -16.411      0.000     -23.122     -18.188
C(fips_code)[T.48459]                       10.7383      1.258      8.533      0.000       8.272      13.205
C(fips_code)[T.48465]                       -4.0093      1.259     -3.184      0.001      -6.477      -1.541
C(fips_code)[T.48467]                        5.3136      1.259      4.219      0.000       2.845       7.782
C(fips_code)[T.48469]                       -3.3388      1.259     -2.652      0.008      -5.806      -0.871
C(fips_code)[T.48471]                       -1.1702      1.267     -0.924      0.356      -3.654       1.313
C(fips_code)[T.48473]                       -0.6516      1.258     -0.518      0.605      -3.118       1.815
C(fips_code)[T.48479]                      -14.9275      1.259    -11.857      0.000     -17.395     -12.460
C(fips_code)[T.48481]                        5.3441      1.259      4.243      0.000       2.875       7.813
C(fips_code)[T.48485]                        0.0608      1.259      0.048      0.961      -2.407       2.528
C(fips_code)[T.48491]                      -11.9471      1.259     -9.490      0.000     -14.415      -9.480
C(fips_code)[T.48493]                       -4.1834      1.261     -3.319      0.001      -6.654      -1.713
C(fips_code)[T.48497]                        5.6657      1.259      4.502      0.000       3.199       8.133
C(fips_code)[T.48499]                       10.3656      1.259      8.233      0.000       7.898      12.833
C(fips_code)[T.49035]                       -1.8065      2.482     -0.728      0.467      -6.671       3.058
C(fips_code)[T.50007]                       -8.2835      1.211     -6.838      0.000     -10.658      -5.909
C(fips_code)[T.50011]                        8.7289      1.211      7.209      0.000       6.356      11.102
C(fips_code)[T.50021]                        5.4251      1.214      4.468      0.000       3.045       7.805
C(fips_code)[T.50023]                       -6.5825      1.211     -5.436      0.000      -8.956      -4.209
C(fips_code)[T.50027]                       -3.7135      1.211     -3.067      0.002      -6.087      -1.340
C(fips_code)[T.51001]                        5.0446      1.409      3.581      0.000       2.283       7.806
C(fips_code)[T.51013]                      -33.6414      1.251    -26.897      0.000     -36.093     -31.190
C(fips_code)[T.51019]                        3.7239      1.248      2.985      0.003       1.278       6.170
C(fips_code)[T.51033]                       -6.6045      1.647     -4.010      0.000      -9.833      -3.376
C(fips_code)[T.51041]                       -3.4443      1.247     -2.762      0.006      -5.889      -1.000
C(fips_code)[T.51047]                        0.6261      1.249      0.501      0.616      -1.822       3.074
C(fips_code)[T.51061]                        3.4231      1.248      2.743      0.006       0.977       5.869
C(fips_code)[T.51067]                       11.5453      1.248      9.254      0.000       9.100      13.991
C(fips_code)[T.51073]                        4.2248      1.248      3.387      0.001       1.779       6.670
C(fips_code)[T.51083]                        6.4493      1.474      4.375      0.000       3.560       9.339
C(fips_code)[T.51085]                       -0.2803      1.248     -0.225      0.822      -2.727       2.166
C(fips_code)[T.51087]                       -8.5167      1.247     -6.827      0.000     -10.962      -6.071
C(fips_code)[T.51093]                        2.3805      1.248      1.907      0.057      -0.066       4.827
C(fips_code)[T.51107]                      -15.0978      1.247    -12.104      0.000     -17.543     -12.653
C(fips_code)[T.51109]                       13.3477      1.248     10.697      0.000      10.902      15.793
C(fips_code)[T.51137]                       10.3793      1.760      5.896      0.000       6.929      13.830
C(fips_code)[T.51155]                       16.0780      1.253     12.835      0.000      13.623      18.533
C(fips_code)[T.51171]                        2.6387      1.248      2.114      0.035       0.192       5.086
C(fips_code)[T.51179]                        0.1862      1.247      0.149      0.881      -2.259       2.631
C(fips_code)[T.51185]                       15.5760      1.406     11.075      0.000      12.819      18.333
C(fips_code)[T.51187]                       10.4350      1.247      8.367      0.000       7.990      12.880
C(fips_code)[T.51510]                      -18.3765      1.248    -14.724      0.000     -20.823     -15.930
C(fips_code)[T.51550]                       -1.0200      1.247     -0.818      0.413      -3.465       1.425
C(fips_code)[T.51650]                       -0.6716      1.249     -0.538      0.591      -3.120       1.777
C(fips_code)[T.51700]                       -3.8113      1.250     -3.050      0.002      -6.261      -1.362
C(fips_code)[T.51710]                       -5.6455      1.249     -4.521      0.000      -8.093      -3.198
C(fips_code)[T.51740]                       12.6001      1.249     10.090      0.000      10.152      15.048
C(fips_code)[T.51760]                      -12.9758      1.249    -10.386      0.000     -15.425     -10.527
C(fips_code)[T.51770]                       -7.6569      1.248     -6.135      0.000     -10.103      -5.211
C(fips_code)[T.51800]                       -1.5782      1.248     -1.265      0.206      -4.024       0.867
C(fips_code)[T.51810]                       -3.5464      1.247     -2.843      0.004      -5.991      -1.102
C(fips_code)[T.53003]                        4.7673      2.078      2.294      0.022       0.694       8.840
C(fips_code)[T.53005]                       -5.6986      0.899     -6.338      0.000      -7.461      -3.936
C(fips_code)[T.53007]                        1.3407      1.244      1.077      0.281      -1.099       3.780
C(fips_code)[T.53009]                        4.7559      1.246      3.816      0.000       2.313       7.198
C(fips_code)[T.53011]                       -6.3301      1.244     -5.087      0.000      -8.769      -3.891
C(fips_code)[T.53015]                        6.3968      1.245      5.139      0.000       3.957       8.837
C(fips_code)[T.53017]                      -10.6789      1.244     -8.581      0.000     -13.118      -8.240
C(fips_code)[T.53021]                        5.1888      1.245      4.167      0.000       2.748       7.629
C(fips_code)[T.53025]                       10.4748      1.245      8.416      0.000       8.035      12.915
C(fips_code)[T.53027]                       11.1527      1.249      8.929      0.000       8.704      13.601
C(fips_code)[T.53029]                        4.6122      1.244      3.706      0.000       2.173       7.051
C(fips_code)[T.53033]                      -22.7055      1.245    -18.244      0.000     -25.145     -20.266
C(fips_code)[T.53035]                       -4.0702      1.245     -3.270      0.001      -6.510      -1.630
C(fips_code)[T.53037]                       -4.4444      1.244     -3.571      0.000      -6.884      -2.005
C(fips_code)[T.53041]                        2.1616      1.245      1.736      0.083      -0.279       4.602
C(fips_code)[T.53045]                       13.9688      1.245     11.223      0.000      11.529      16.409
C(fips_code)[T.53047]                       16.1618      1.343     12.032      0.000      13.529      18.795
C(fips_code)[T.53053]                       -4.0196      1.246     -3.226      0.001      -6.462      -1.578
C(fips_code)[T.53057]                       -2.8638      1.246     -2.298      0.022      -5.306      -0.422
C(fips_code)[T.53061]                       -6.7333      1.246     -5.404      0.000      -9.176      -4.291
C(fips_code)[T.53063]                       -1.2017      1.245     -0.966      0.334      -3.641       1.238
C(fips_code)[T.53065]                       18.4857      1.550     11.929      0.000      15.448      21.523
C(fips_code)[T.53067]                       -6.2197      1.244     -4.998      0.000      -8.659      -3.780
C(fips_code)[T.53071]                       -1.0740      1.247     -0.861      0.389      -3.518       1.370
C(fips_code)[T.53073]                      -14.4660      1.245    -11.620      0.000     -16.906     -12.026
C(fips_code)[T.53075]                      -14.0061      1.254    -11.166      0.000     -16.465     -11.548
C(fips_code)[T.53077]                        2.1826      1.247      1.750      0.080      -0.262       4.627
C(fips_code)[T.54003]                        3.4840      1.222      2.851      0.004       1.088       5.880
C(fips_code)[T.54011]                       -2.2817      1.221     -1.869      0.062      -4.675       0.111
C(fips_code)[T.54033]                       -3.3454      1.221     -2.741      0.006      -5.738      -0.953
C(fips_code)[T.54037]                       -3.0218      1.223     -2.471      0.013      -5.419      -0.624
C(fips_code)[T.54039]                       -1.8729      1.222     -1.533      0.125      -4.268       0.522
C(fips_code)[T.54049]                        1.7732      1.222      1.452      0.147      -0.621       4.168
C(fips_code)[T.54055]                        6.7507      1.222      5.526      0.000       4.356       9.145
C(fips_code)[T.54061]                      -11.8398      1.222     -9.686      0.000     -14.236      -9.444
C(fips_code)[T.54069]                       -3.7732      1.221     -3.090      0.002      -6.167      -1.379
C(fips_code)[T.54079]                        7.2211      1.221      5.916      0.000       4.828       9.614
C(fips_code)[T.54081]                        5.4314      1.222      4.446      0.000       3.037       7.826
C(fips_code)[T.54107]                        1.0431      1.221      0.854      0.393      -1.351       3.437
C(fips_code)[T.55005]                        8.9982      1.254      7.174      0.000       6.539      11.457
C(fips_code)[T.55009]                       -8.1406      1.255     -6.487      0.000     -10.600      -5.681
C(fips_code)[T.55015]                      -11.7001      1.256     -9.317      0.000     -14.162      -9.239
C(fips_code)[T.55017]                        4.2732      1.254      3.407      0.001       1.815       6.732
C(fips_code)[T.55019]                       -6.3565      2.093     -3.037      0.002     -10.459      -2.254
C(fips_code)[T.55021]                        0.3652      1.254      0.291      0.771      -2.093       2.824
C(fips_code)[T.55025]                      -21.2058      1.256    -16.881      0.000     -23.668     -18.744
C(fips_code)[T.55027]                        5.2591      1.256      4.189      0.000       2.798       7.720
C(fips_code)[T.55031]                        8.6009      1.264      6.807      0.000       6.124      11.078
C(fips_code)[T.55033]                        0.1808      1.255      0.144      0.885      -2.279       2.641
C(fips_code)[T.55035]                       -7.3615      1.255     -5.867      0.000      -9.821      -4.902
C(fips_code)[T.55039]                       -0.7714      1.254     -0.615      0.539      -3.230       1.687
C(fips_code)[T.55043]                        5.8898      1.255      4.693      0.000       3.430       8.350
C(fips_code)[T.55045]                        1.2494      1.256      0.995      0.320      -1.213       3.711
C(fips_code)[T.55055]                       -0.6632      1.255     -0.528      0.597      -3.123       1.797
C(fips_code)[T.55057]                        3.0818      2.339      1.317      0.188      -1.503       7.667
C(fips_code)[T.55059]                       -1.0613      1.255     -0.846      0.398      -3.522       1.399
C(fips_code)[T.55063]                       -7.7233      1.255     -6.156      0.000     -10.183      -5.264
C(fips_code)[T.55069]                        5.8306      1.657      3.519      0.000       2.583       9.079
C(fips_code)[T.55071]                       -1.0369      1.254     -0.827      0.408      -3.496       1.422
C(fips_code)[T.55073]                       -4.9161      1.256     -3.915      0.000      -7.377      -2.455
C(fips_code)[T.55075]                        5.3685      1.258      4.269      0.000       2.904       7.833
C(fips_code)[T.55079]                       -6.1624      1.256     -4.906      0.000      -8.624      -3.701
C(fips_code)[T.55081]                        4.8230      1.255      3.843      0.000       2.363       7.283
C(fips_code)[T.55083]                       -9.1359      2.339     -3.905      0.000     -13.721      -4.551
C(fips_code)[T.55085]                        4.2113      1.257      3.351      0.001       1.748       6.675
C(fips_code)[T.55087]                      -12.8818      1.254    -10.269      0.000     -15.341     -10.423
C(fips_code)[T.55089]                       -9.6431      1.255     -7.683      0.000     -12.103      -7.183
C(fips_code)[T.55093]                        5.6748      1.656      3.426      0.001       2.428       8.921
C(fips_code)[T.55095]                       18.3305      1.255     14.602      0.000      15.870      20.791
C(fips_code)[T.55097]                       -2.7687      1.255     -2.206      0.027      -5.229      -0.308
C(fips_code)[T.55101]                        0.6126      1.255      0.488      0.626      -1.848       3.073
C(fips_code)[T.55105]                        1.4099      1.255      1.123      0.261      -1.050       3.870
C(fips_code)[T.55109]                        5.2202      1.255      4.158      0.000       2.760       7.681
C(fips_code)[T.55111]                        2.5937      1.258      2.062      0.039       0.129       5.059
C(fips_code)[T.55115]                        5.0842      1.254      4.053      0.000       2.625       7.543
C(fips_code)[T.55117]                        0.2610      1.254      0.208      0.835      -2.198       2.720
C(fips_code)[T.55121]                        1.2038      2.093      0.575      0.565      -2.899       5.306
C(fips_code)[T.55127]                        8.8269      1.254      7.037      0.000       6.368      11.286
C(fips_code)[T.55131]                        2.6115      1.255      2.082      0.037       0.152       5.071
C(fips_code)[T.55133]                       -8.7840      1.255     -7.000      0.000     -11.244      -6.324
C(fips_code)[T.55135]                        7.9702      1.255      6.353      0.000       5.511      10.429
C(fips_code)[T.55139]                       -5.7234      1.254     -4.562      0.000      -8.182      -3.264
C(fips_code)[T.55141]                        6.0651      1.254      4.835      0.000       3.606       8.524
C(fips_code)[T.56001]                       -7.5428      1.228     -6.145      0.000      -9.949      -5.137
C(fips_code)[T.56005]                        0.4564      1.225      0.373      0.709      -1.945       2.857
C(fips_code)[T.56013]                        5.9940      1.225      4.893      0.000       3.593       8.395
C(fips_code)[T.56021]                       -6.0638      1.225     -4.952      0.000      -8.464      -3.663
C(fips_code)[T.56025]                        0.0867      1.227      0.071      0.944      -2.318       2.492
C(fips_code)[T.56029]                        6.3180      1.824      3.464      0.001       2.743       9.893
C(fips_code)[T.56033]                       -0.7191      1.225     -0.587      0.557      -3.120       1.681
C(fips_code)[T.56037]                        0.8077      1.225      0.659      0.510      -1.594       3.210
C(week)[T.11]:C(state)[T.Alaska]            -4.5764      3.822     -1.198      0.231     -12.067       2.914
C(week)[T.12]:C(state)[T.Alaska]             0.2361      3.551      0.066      0.947      -6.723       7.196
C(week)[T.13]:C(state)[T.Alaska]            -4.7905      3.579     -1.338      0.181     -11.806       2.225
C(week)[T.14]:C(state)[T.Alaska]            -5.9809      3.549     -1.685      0.092     -12.937       0.975
C(week)[T.15]:C(state)[T.Alaska]             0.9287      3.556      0.261      0.794      -6.042       7.899
C(week)[T.16]:C(state)[T.Alaska]            -0.4184      3.556     -0.118      0.906      -7.389       6.552
C(week)[T.17]:C(state)[T.Alaska]             2.8931      3.552      0.814      0.415      -4.070       9.856
C(week)[T.18]:C(state)[T.Alaska]             3.6605      3.581      1.022      0.307      -3.360      10.681
C(week)[T.19]:C(state)[T.Alaska]             9.9149      3.549      2.794      0.005       2.959      16.871
C(week)[T.20]:C(state)[T.Alaska]            11.5482      3.549      3.254      0.001       4.592      18.504
C(week)[T.21]:C(state)[T.Alaska]            13.2319      3.549      3.729      0.000       6.276      20.188
C(week)[T.22]:C(state)[T.Alaska]            10.1622      3.822      2.659      0.008       2.670      17.654
C(week)[T.23]:C(state)[T.Alaska]             8.2499      3.822      2.158      0.031       0.758      15.742
C(week)[T.11]:C(state)[T.Arizona]           -7.7166      2.618     -2.947      0.003     -12.849      -2.584
C(week)[T.12]:C(state)[T.Arizona]            1.3074      2.201      0.594      0.552      -3.006       5.621
C(week)[T.13]:C(state)[T.Arizona]           -4.0278      2.201     -1.830      0.067      -8.342       0.286
C(week)[T.14]:C(state)[T.Arizona]           -5.4474      2.203     -2.473      0.013      -9.765      -1.130
C(week)[T.15]:C(state)[T.Arizona]           -4.8000      2.216     -2.166      0.030      -9.144      -0.456
C(week)[T.16]:C(state)[T.Arizona]          -10.7731      2.216     -4.861      0.000     -15.117      -6.429
C(week)[T.17]:C(state)[T.Arizona]          -10.8698      2.210     -4.918      0.000     -15.202      -6.537
C(week)[T.18]:C(state)[T.Arizona]          -14.1730      2.205     -6.428      0.000     -18.495      -9.851
C(week)[T.19]:C(state)[T.Arizona]          -11.9889      2.257     -5.311      0.000     -16.413      -7.565
C(week)[T.20]:C(state)[T.Arizona]          -11.8030      2.257     -5.229      0.000     -16.228      -7.378
C(week)[T.21]:C(state)[T.Arizona]           -9.7904      2.205     -4.439      0.000     -14.113      -5.468
C(week)[T.22]:C(state)[T.Arizona]           -8.8484      2.206     -4.012      0.000     -13.172      -4.525
C(week)[T.23]:C(state)[T.Arizona]          -12.0622      2.203     -5.475      0.000     -16.381      -7.744
C(week)[T.11]:C(state)[T.California]        -2.8295      1.540     -1.837      0.066      -5.849       0.190
C(week)[T.12]:C(state)[T.California]        -1.1673      1.614     -0.723      0.470      -4.332       1.997
C(week)[T.13]:C(state)[T.California]        -2.9311      1.618     -1.811      0.070      -6.103       0.241
C(week)[T.14]:C(state)[T.California]        -2.4968      1.549     -1.611      0.107      -5.534       0.540
C(week)[T.15]:C(state)[T.California]        -0.6908      1.566     -0.441      0.659      -3.761       2.379
C(week)[T.16]:C(state)[T.California]       -14.8128      1.566     -9.458      0.000     -17.883     -11.743
C(week)[T.17]:C(state)[T.California]       -20.9586      1.558    -13.454      0.000     -24.012     -17.905
C(week)[T.18]:C(state)[T.California]       -23.0337      1.549    -14.869      0.000     -26.070     -19.997
C(week)[T.19]:C(state)[T.California]       -19.5486      1.620    -12.065      0.000     -22.725     -16.373
C(week)[T.20]:C(state)[T.California]       -24.2901      1.597    -15.214      0.000     -27.419     -21.161
C(week)[T.21]:C(state)[T.California]       -24.1129      1.546    -15.595      0.000     -27.144     -21.082
C(week)[T.22]:C(state)[T.California]       -19.5910      1.546    -12.670      0.000     -22.622     -16.560
C(week)[T.23]:C(state)[T.California]       -24.6840      1.547    -15.959      0.000     -27.716     -21.652
C(week)[T.11]:C(state)[T.Colorado]          -4.5064      1.971     -2.286      0.022      -8.371      -0.642
C(week)[T.12]:C(state)[T.Colorado]          -9.6355      1.971     -4.888      0.000     -13.500      -5.771
C(week)[T.13]:C(state)[T.Colorado]          -7.6596      2.026     -3.780      0.000     -11.631      -3.688
C(week)[T.14]:C(state)[T.Colorado]         -10.0631      1.993     -5.048      0.000     -13.970      -6.156
C(week)[T.15]:C(state)[T.Colorado]          -6.2322      2.008     -3.104      0.002     -10.168      -2.296
C(week)[T.16]:C(state)[T.Colorado]         -17.2583      2.030     -8.503      0.000     -21.237     -13.280
C(week)[T.17]:C(state)[T.Colorado]         -12.8478      2.001     -6.421      0.000     -16.770      -8.926
C(week)[T.18]:C(state)[T.Colorado]         -16.5015      2.032     -8.119      0.000     -20.485     -12.518
C(week)[T.19]:C(state)[T.Colorado]         -12.3751      1.972     -6.275      0.000     -16.241      -8.509
C(week)[T.20]:C(state)[T.Colorado]         -16.3585      1.973     -8.293      0.000     -20.225     -12.492
C(week)[T.21]:C(state)[T.Colorado]         -14.0100      1.972     -7.103      0.000     -17.876     -10.144
C(week)[T.22]:C(state)[T.Colorado]          -9.1706      1.972     -4.650      0.000     -13.037      -5.305
C(week)[T.23]:C(state)[T.Colorado]         -11.4231      1.972     -5.794      0.000     -15.287      -7.559
C(week)[T.11]:C(state)[T.Connecticut]      -10.0816      2.896     -3.481      0.001     -15.758      -4.405
C(week)[T.12]:C(state)[T.Connecticut]       -5.4279      2.525     -2.150      0.032     -10.377      -0.479
C(week)[T.13]:C(state)[T.Connecticut]       -7.7420      2.568     -3.015      0.003     -12.775      -2.709
C(week)[T.14]:C(state)[T.Connecticut]       -8.7228      2.528     -3.450      0.001     -13.679      -3.767
C(week)[T.15]:C(state)[T.Connecticut]       -5.7342      2.544     -2.254      0.024     -10.720      -0.749
C(week)[T.16]:C(state)[T.Connecticut]      -14.2977      2.547     -5.614      0.000     -19.289      -9.306
C(week)[T.17]:C(state)[T.Connecticut]      -16.1137      2.548     -6.325      0.000     -21.108     -11.120
C(week)[T.18]:C(state)[T.Connecticut]      -16.6034      2.531     -6.559      0.000     -21.565     -11.642
C(week)[T.19]:C(state)[T.Connecticut]      -11.9728      2.575     -4.650      0.000     -17.020      -6.926
C(week)[T.20]:C(state)[T.Connecticut]      -14.1662      2.573     -5.505      0.000     -19.210      -9.122
C(week)[T.21]:C(state)[T.Connecticut]      -10.8412      2.572     -4.216      0.000     -15.882      -5.801
C(week)[T.22]:C(state)[T.Connecticut]       -9.7658      2.526     -3.866      0.000     -14.717      -4.814
C(week)[T.23]:C(state)[T.Connecticut]      -11.4626      2.525     -4.539      0.000     -16.412      -6.513
C(week)[T.11]:C(state)[T.Delaware]          -0.0861      4.040     -0.021      0.983      -8.005       7.833
C(week)[T.12]:C(state)[T.Delaware]          -1.1448      4.040     -0.283      0.777      -9.064       6.774
C(week)[T.13]:C(state)[T.Delaware]          -6.2044      4.067     -1.525      0.127     -14.177       1.768
C(week)[T.14]:C(state)[T.Delaware]          -4.5145      4.040     -1.117      0.264     -12.434       3.405
C(week)[T.15]:C(state)[T.Delaware]          -0.3581      4.047     -0.088      0.929      -8.290       7.574
C(week)[T.16]:C(state)[T.Delaware]          -9.1043      4.047     -2.250      0.024     -17.037      -1.172
C(week)[T.17]:C(state)[T.Delaware]         -10.2042      4.046     -2.522      0.012     -18.134      -2.274
C(week)[T.18]:C(state)[T.Delaware]         -12.7530      4.043     -3.155      0.002     -20.677      -4.829
C(week)[T.19]:C(state)[T.Delaware]          -6.7736      4.073     -1.663      0.096     -14.757       1.210
C(week)[T.20]:C(state)[T.Delaware]          -8.4705      4.074     -2.079      0.038     -16.457      -0.484
C(week)[T.21]:C(state)[T.Delaware]         -12.7269      4.044     -3.147      0.002     -20.653      -4.801
C(week)[T.22]:C(state)[T.Delaware]          -7.9767      4.043     -1.973      0.049     -15.902      -0.051
C(week)[T.23]:C(state)[T.Delaware]          -6.3555      4.041     -1.573      0.116     -14.277       1.566
C(week)[T.11]:C(state)[T.Florida]           -6.4838      2.080     -3.118      0.002     -10.560      -2.407
C(week)[T.12]:C(state)[T.Florida]            3.2833      1.521      2.159      0.031       0.302       6.264
C(week)[T.13]:C(state)[T.Florida]           -3.0134      1.523     -1.979      0.048      -5.998      -0.029
C(week)[T.14]:C(state)[T.Florida]           -5.3639      1.522     -3.525      0.000      -8.347      -2.381
C(week)[T.15]:C(state)[T.Florida]           -5.4990      1.542     -3.565      0.000      -8.522      -2.476
C(week)[T.16]:C(state)[T.Florida]          -12.3340      1.542     -7.997      0.000     -15.357      -9.311
C(week)[T.17]:C(state)[T.Florida]          -13.7728      1.531     -8.998      0.000     -16.773     -10.773
C(week)[T.18]:C(state)[T.Florida]          -14.6886      1.521     -9.657      0.000     -17.670     -11.707
C(week)[T.19]:C(state)[T.Florida]           -7.3925      1.597     -4.630      0.000     -10.522      -4.263
C(week)[T.20]:C(state)[T.Florida]           -9.2916      1.597     -5.820      0.000     -12.421      -6.162
C(week)[T.21]:C(state)[T.Florida]           -8.2156      1.597     -5.146      0.000     -11.345      -5.086
C(week)[T.22]:C(state)[T.Florida]           -5.2584      1.597     -3.293      0.001      -8.388      -2.129
C(week)[T.23]:C(state)[T.Florida]          -11.6896      1.596     -7.325      0.000     -14.818      -8.562
C(week)[T.11]:C(state)[T.Georgia]           -8.1091      2.067     -3.923      0.000     -12.161      -4.058
C(week)[T.12]:C(state)[T.Georgia]            2.1549      1.503      1.433      0.152      -0.792       5.102
C(week)[T.13]:C(state)[T.Georgia]           -1.2433      1.503     -0.827      0.408      -4.190       1.704
C(week)[T.14]:C(state)[T.Georgia]           -0.9687      1.504     -0.644      0.520      -3.918       1.980
C(week)[T.15]:C(state)[T.Georgia]           -0.3243      1.529     -0.212      0.832      -3.321       2.672
C(week)[T.16]:C(state)[T.Georgia]           -3.1774      1.529     -2.078      0.038      -6.174      -0.181
C(week)[T.17]:C(state)[T.Georgia]           -3.4145      1.517     -2.251      0.024      -6.388      -0.441
C(week)[T.18]:C(state)[T.Georgia]           -0.9470      1.508     -0.628      0.530      -3.903       2.009
C(week)[T.19]:C(state)[T.Georgia]           -0.0271      1.507     -0.018      0.986      -2.982       2.928
C(week)[T.20]:C(state)[T.Georgia]           -3.6705      1.504     -2.440      0.015      -6.619      -0.722
C(week)[T.21]:C(state)[T.Georgia]           -5.1080      1.504     -3.395      0.001      -8.057      -2.159
C(week)[T.22]:C(state)[T.Georgia]           -3.3916      1.504     -2.254      0.024      -6.340      -0.443
C(week)[T.23]:C(state)[T.Georgia]           -4.5623      1.507     -3.027      0.002      -7.517      -1.608
C(week)[T.11]:C(state)[T.Idaho]             -8.9410      2.673     -3.346      0.001     -14.179      -3.702
C(week)[T.12]:C(state)[T.Idaho]             -1.3911      2.673     -0.521      0.603      -6.630       3.847
C(week)[T.13]:C(state)[T.Idaho]             -0.2203      2.313     -0.095      0.924      -4.754       4.313
C(week)[T.14]:C(state)[T.Idaho]             -5.2210      2.353     -2.219      0.027      -9.834      -0.608
C(week)[T.15]:C(state)[T.Idaho]              2.5709      2.318      1.109      0.267      -1.972       7.114
C(week)[T.16]:C(state)[T.Idaho]             -1.6879      2.318     -0.728      0.466      -6.231       2.855
C(week)[T.17]:C(state)[T.Idaho]             -1.8138      2.312     -0.784      0.433      -6.346       2.718
C(week)[T.18]:C(state)[T.Idaho]             -3.2037      2.266     -1.414      0.157      -7.645       1.237
C(week)[T.19]:C(state)[T.Idaho]             -0.8926      2.265     -0.394      0.694      -5.333       3.548
C(week)[T.20]:C(state)[T.Idaho]             -5.6881      2.265     -2.511      0.012     -10.128      -1.248
C(week)[T.21]:C(state)[T.Idaho]              0.1398      2.265      0.062      0.951      -4.300       4.580
C(week)[T.22]:C(state)[T.Idaho]              5.5488      2.266      2.449      0.014       1.108       9.990
C(week)[T.23]:C(state)[T.Idaho]              2.5028      2.266      1.104      0.269      -1.940       6.945
C(week)[T.11]:C(state)[T.Illinois]         -12.9333      2.080     -6.219      0.000     -17.010      -8.857
C(week)[T.12]:C(state)[T.Illinois]         -11.9568      1.596     -7.493      0.000     -15.085      -8.829
C(week)[T.13]:C(state)[T.Illinois]         -13.6654      1.642     -8.322      0.000     -16.884     -10.447
C(week)[T.14]:C(state)[T.Illinois]          -7.5797      1.564     -4.846      0.000     -10.645      -4.514
C(week)[T.15]:C(state)[T.Illinois]          -3.5745      1.592     -2.245      0.025      -6.695      -0.454
C(week)[T.16]:C(state)[T.Illinois]         -10.1250      1.597     -6.338      0.000     -13.256      -6.994
C(week)[T.17]:C(state)[T.Illinois]         -10.2407      1.579     -6.487      0.000     -13.335      -7.146
C(week)[T.18]:C(state)[T.Illinois]         -11.8023      1.573     -7.504      0.000     -14.885      -8.720
C(week)[T.19]:C(state)[T.Illinois]         -11.5981      1.644     -7.056      0.000     -14.820      -8.376
C(week)[T.20]:C(state)[T.Illinois]         -17.4857      1.639    -10.667      0.000     -20.699     -14.273
C(week)[T.21]:C(state)[T.Illinois]         -15.8352      1.639     -9.661      0.000     -19.048     -12.622
C(week)[T.22]:C(state)[T.Illinois]          -9.2273      1.639     -5.630      0.000     -12.440      -6.015
C(week)[T.23]:C(state)[T.Illinois]         -14.3520      1.559     -9.204      0.000     -17.409     -11.295
C(week)[T.11]:C(state)[T.Indiana]           -5.9170      1.521     -3.891      0.000      -8.898      -2.936
C(week)[T.12]:C(state)[T.Indiana]           -9.7633      1.521     -6.420      0.000     -12.744      -6.782
C(week)[T.13]:C(state)[T.Indiana]           -9.8062      1.591     -6.163      0.000     -12.925      -6.687
C(week)[T.14]:C(state)[T.Indiana]           -5.3460      1.542     -3.466      0.001      -8.369      -2.323
C(week)[T.15]:C(state)[T.Indiana]            0.3815      1.561      0.244      0.807      -2.678       3.441
C(week)[T.16]:C(state)[T.Indiana]           -5.8743      1.561     -3.764      0.000      -8.934      -2.815
C(week)[T.17]:C(state)[T.Indiana]           -3.3601      1.545     -2.175      0.030      -6.389      -0.332
C(week)[T.18]:C(state)[T.Indiana]           -4.9295      1.537     -3.207      0.001      -7.942      -1.917
C(week)[T.19]:C(state)[T.Indiana]           -6.2151      1.535     -4.050      0.000      -9.223      -3.207
C(week)[T.20]:C(state)[T.Indiana]           -5.2592      1.524     -3.452      0.001      -8.246      -2.273
C(week)[T.21]:C(state)[T.Indiana]           -2.1241      1.524     -1.394      0.163      -5.111       0.863
C(week)[T.22]:C(state)[T.Indiana]            4.0643      1.524      2.668      0.008       1.078       7.051
C(week)[T.23]:C(state)[T.Indiana]            1.0366      1.531      0.677      0.498      -1.964       4.038
C(week)[T.11]:C(state)[T.Kansas]           -12.3985      2.334     -5.313      0.000     -16.973      -7.824
C(week)[T.12]:C(state)[T.Kansas]            -4.7538      1.863     -2.551      0.011      -8.406      -1.102
C(week)[T.13]:C(state)[T.Kansas]            -4.6965      1.857     -2.529      0.011      -8.336      -1.057
C(week)[T.14]:C(state)[T.Kansas]           -11.2521      1.854     -6.070      0.000     -14.886      -7.618
C(week)[T.15]:C(state)[T.Kansas]            -4.1248      1.882     -2.192      0.028      -7.814      -0.436
C(week)[T.16]:C(state)[T.Kansas]           -10.1242      1.868     -5.419      0.000     -13.786      -6.462
C(week)[T.17]:C(state)[T.Kansas]            -8.8149      1.875     -4.701      0.000     -12.491      -5.139
C(week)[T.18]:C(state)[T.Kansas]            -8.5920      1.856     -4.630      0.000     -12.229      -4.955
C(week)[T.19]:C(state)[T.Kansas]            -5.3056      1.917     -2.767      0.006      -9.064      -1.547
C(week)[T.20]:C(state)[T.Kansas]           -10.5689      1.916     -5.517      0.000     -14.324      -6.814
C(week)[T.21]:C(state)[T.Kansas]           -13.9285      2.334     -5.968      0.000     -18.503      -9.354
C(week)[T.22]:C(state)[T.Kansas]           -12.4271      2.334     -5.324      0.000     -17.002      -7.852
C(week)[T.23]:C(state)[T.Kansas]           -14.9330      2.334     -6.399      0.000     -19.507     -10.359
C(week)[T.11]:C(state)[T.Kentucky]         -14.4220      2.238     -6.443      0.000     -18.810     -10.034
C(week)[T.12]:C(state)[T.Kentucky]          -9.0927      1.731     -5.251      0.000     -12.487      -5.699
C(week)[T.13]:C(state)[T.Kentucky]          -3.6947      1.803     -2.049      0.040      -7.229      -0.160
C(week)[T.14]:C(state)[T.Kentucky]          -4.1133      1.763     -2.334      0.020      -7.568      -0.659
C(week)[T.15]:C(state)[T.Kentucky]          -1.4701      1.777     -0.827      0.408      -4.953       2.013
C(week)[T.16]:C(state)[T.Kentucky]          -6.8631      1.777     -3.862      0.000     -10.346      -3.380
C(week)[T.17]:C(state)[T.Kentucky]          -8.2854      1.770     -4.682      0.000     -11.754      -4.817
C(week)[T.18]:C(state)[T.Kentucky]          -9.5042      1.752     -5.426      0.000     -12.938      -6.071
C(week)[T.19]:C(state)[T.Kentucky]          -9.1038      1.807     -5.037      0.000     -12.646      -5.561
C(week)[T.20]:C(state)[T.Kentucky]         -13.9081      1.807     -7.695      0.000     -17.451     -10.365
C(week)[T.21]:C(state)[T.Kentucky]         -12.1533      1.798     -6.760      0.000     -15.677      -8.630
C(week)[T.22]:C(state)[T.Kentucky]          -1.9377      1.798     -1.078      0.281      -5.462       1.586
C(week)[T.23]:C(state)[T.Kentucky]          -9.6493      1.738     -5.552      0.000     -13.056      -6.243
C(week)[T.11]:C(state)[T.Louisiana]         -9.2272      2.252     -4.098      0.000     -13.641      -4.814
C(week)[T.12]:C(state)[T.Louisiana]         -4.1673      1.749     -2.383      0.017      -7.595      -0.740
C(week)[T.13]:C(state)[T.Louisiana]         -4.8737      1.820     -2.677      0.007      -8.442      -1.306
C(week)[T.14]:C(state)[T.Louisiana]         -3.8832      1.765     -2.200      0.028      -7.342      -0.424
C(week)[T.15]:C(state)[T.Louisiana]         -4.8111      1.779     -2.705      0.007      -8.297      -1.325
C(week)[T.16]:C(state)[T.Louisiana]         -7.2445      1.778     -4.074      0.000     -10.730      -3.759
C(week)[T.17]:C(state)[T.Louisiana]         -6.1124      1.770     -3.453      0.001      -9.582      -2.642
C(week)[T.18]:C(state)[T.Louisiana]         -8.0826      1.761     -4.591      0.000     -11.534      -4.631
C(week)[T.19]:C(state)[T.Louisiana]         -4.8576      1.815     -2.677      0.007      -8.415      -1.300
C(week)[T.20]:C(state)[T.Louisiana]         -8.9437      1.815     -4.928      0.000     -12.501      -5.387
C(week)[T.21]:C(state)[T.Louisiana]         -7.1092      1.749     -4.064      0.000     -10.538      -3.680
C(week)[T.22]:C(state)[T.Louisiana]         -5.6965      1.749     -3.257      0.001      -9.125      -2.268
C(week)[T.23]:C(state)[T.Louisiana]         -7.9254      1.749     -4.531      0.000     -11.354      -4.497
C(week)[T.11]:C(state)[T.Maine]             -9.8318      2.618     -3.755      0.000     -14.964      -4.700
C(week)[T.12]:C(state)[T.Maine]             -7.9194      2.201     -3.599      0.000     -12.233      -3.606
C(week)[T.13]:C(state)[T.Maine]             -9.5414      2.337     -4.082      0.000     -14.123      -4.960
C(week)[T.14]:C(state)[T.Maine]            -12.3512      2.398     -5.151      0.000     -17.051      -7.651
C(week)[T.15]:C(state)[T.Maine]             -9.4993      2.410     -3.942      0.000     -14.222      -4.776
C(week)[T.16]:C(state)[T.Maine]             -9.5008      2.409     -3.943      0.000     -14.224      -4.778
C(week)[T.17]:C(state)[T.Maine]            -11.2055      2.404     -4.661      0.000     -15.918      -6.493
C(week)[T.18]:C(state)[T.Maine]            -14.6415      2.397     -6.107      0.000     -19.341      -9.942
C(week)[T.19]:C(state)[T.Maine]             -9.6157      2.445     -3.933      0.000     -14.408      -4.823
C(week)[T.20]:C(state)[T.Maine]             -9.8191      2.387     -4.114      0.000     -14.498      -5.140
C(week)[T.21]:C(state)[T.Maine]             -3.4328      2.337     -1.469      0.142      -8.013       1.147
C(week)[T.22]:C(state)[T.Maine]             -0.9349      2.293     -0.408      0.683      -5.429       3.560
C(week)[T.23]:C(state)[T.Maine]             -5.1219      2.254     -2.272      0.023      -9.540      -0.704
C(week)[T.11]:C(state)[T.Maryland]           0.2023      1.907      0.106      0.916      -3.536       3.941
C(week)[T.12]:C(state)[T.Maryland]          -0.7907      1.907     -0.415      0.678      -4.529       2.948
C(week)[T.13]:C(state)[T.Maryland]          -5.9925      1.907     -3.142      0.002      -9.731      -2.254
C(week)[T.14]:C(state)[T.Maryland]          -6.3560      1.909     -3.330      0.001     -10.098      -2.614
C(week)[T.15]:C(state)[T.Maryland]          -2.4995      1.941     -1.288      0.198      -6.304       1.305
C(week)[T.16]:C(state)[T.Maryland]         -11.2976      1.941     -5.821      0.000     -15.102      -7.493
C(week)[T.17]:C(state)[T.Maryland]         -13.6011      1.952     -6.966      0.000     -17.428      -9.774
C(week)[T.18]:C(state)[T.Maryland]         -15.7284      1.927     -8.162      0.000     -19.506     -11.951
C(week)[T.19]:C(state)[T.Maryland]         -11.3931      1.985     -5.741      0.000     -15.283      -7.503
C(week)[T.20]:C(state)[T.Maryland]         -12.5854      1.968     -6.394      0.000     -16.443      -8.727
C(week)[T.21]:C(state)[T.Maryland]         -11.2562      1.968     -5.719      0.000     -15.114      -7.398
C(week)[T.22]:C(state)[T.Maryland]          -4.0581      1.968     -2.062      0.039      -7.916      -0.200
C(week)[T.23]:C(state)[T.Maryland]         -10.8606      1.908     -5.694      0.000     -14.600      -7.122
C(week)[T.11]:C(state)[T.Massachusetts]    -10.3887      2.673     -3.887      0.000     -15.627      -5.150
C(week)[T.12]:C(state)[T.Massachusetts]     -9.0551      2.265     -3.998      0.000     -13.495      -4.615
C(week)[T.13]:C(state)[T.Massachusetts]    -10.4092      2.313     -4.500      0.000     -14.943      -5.875
C(week)[T.14]:C(state)[T.Massachusetts]     -8.7239      2.267     -3.848      0.000     -13.168      -4.280
C(week)[T.15]:C(state)[T.Massachusetts]     -2.9764      2.282     -1.304      0.192      -7.449       1.497
C(week)[T.16]:C(state)[T.Massachusetts]    -13.1284      2.284     -5.748      0.000     -17.605      -8.652
C(week)[T.17]:C(state)[T.Massachusetts]    -13.9551      2.282     -6.116      0.000     -18.427      -9.483
C(week)[T.18]:C(state)[T.Massachusetts]    -15.8107      2.282     -6.929      0.000     -20.283     -11.338
C(week)[T.19]:C(state)[T.Massachusetts]    -12.1074      2.334     -5.187      0.000     -16.682      -7.532
C(week)[T.20]:C(state)[T.Massachusetts]    -14.5445      2.333     -6.235      0.000     -19.117      -9.972
C(week)[T.21]:C(state)[T.Massachusetts]    -15.2344      2.279     -6.684      0.000     -19.702     -10.767
C(week)[T.22]:C(state)[T.Massachusetts]    -11.0915      2.278     -4.868      0.000     -15.558      -6.625
C(week)[T.23]:C(state)[T.Massachusetts]    -15.6149      2.288     -6.826      0.000     -20.099     -11.131
C(week)[T.11]:C(state)[T.Michigan]         -13.9290      2.116     -6.583      0.000     -18.077      -9.781
C(week)[T.12]:C(state)[T.Michigan]         -15.4039      1.570     -9.811      0.000     -18.481     -12.326
C(week)[T.13]:C(state)[T.Michigan]         -20.1594      1.638    -12.304      0.000     -23.371     -16.948
C(week)[T.14]:C(state)[T.Michigan]         -12.7377      1.601     -7.956      0.000     -15.876      -9.599
C(week)[T.15]:C(state)[T.Michigan]          -8.3326      1.637     -5.090      0.000     -11.542      -5.123
C(week)[T.16]:C(state)[T.Michigan]         -15.2655      1.637     -9.323      0.000     -18.475     -12.056
C(week)[T.17]:C(state)[T.Michigan]         -13.0113      1.629     -7.987      0.000     -16.204      -9.818
C(week)[T.18]:C(state)[T.Michigan]         -12.5069      1.686     -7.417      0.000     -15.812      -9.202
C(week)[T.19]:C(state)[T.Michigan]         -11.0918      1.607     -6.902      0.000     -14.242      -7.942
C(week)[T.20]:C(state)[T.Michigan]         -15.5219      1.602     -9.688      0.000     -18.662     -12.381
C(week)[T.21]:C(state)[T.Michigan]         -10.9724      1.602     -6.848      0.000     -14.113      -7.832
C(week)[T.22]:C(state)[T.Michigan]          -6.2935      1.602     -3.928      0.000      -9.434      -3.153
C(week)[T.23]:C(state)[T.Michigan]         -11.3114      1.583     -7.145      0.000     -14.415      -8.208
C(week)[T.11]:C(state)[T.Minnesota]         -8.7282      2.166     -4.030      0.000     -12.973      -4.483
C(week)[T.12]:C(state)[T.Minnesota]         -9.8852      1.637     -6.040      0.000     -13.093      -6.677
C(week)[T.13]:C(state)[T.Minnesota]         -7.7957      1.702     -4.580      0.000     -11.132      -4.459
C(week)[T.14]:C(state)[T.Minnesota]        -11.6978      1.654     -7.071      0.000     -14.941      -8.455
C(week)[T.15]:C(state)[T.Minnesota]         -7.9762      1.697     -4.700      0.000     -11.303      -4.650
C(week)[T.16]:C(state)[T.Minnesota]        -12.1114      1.682     -7.201      0.000     -15.408      -8.814
C(week)[T.17]:C(state)[T.Minnesota]         -9.8285      1.674     -5.870      0.000     -13.110      -6.547
C(week)[T.18]:C(state)[T.Minnesota]        -10.6065      1.661     -6.385      0.000     -13.863      -7.350
C(week)[T.19]:C(state)[T.Minnesota]         -8.6852      1.720     -5.049      0.000     -12.057      -5.314
C(week)[T.20]:C(state)[T.Minnesota]        -13.0770      1.713     -7.632      0.000     -16.436      -9.718
C(week)[T.21]:C(state)[T.Minnesota]        -11.1585      1.644     -6.788      0.000     -14.381      -7.936
C(week)[T.22]:C(state)[T.Minnesota]         -8.4928      1.644     -5.166      0.000     -11.715      -5.271
C(week)[T.23]:C(state)[T.Minnesota]         -5.6326      1.644     -3.427      0.001      -8.854      -2.411
C(week)[T.11]:C(state)[T.Mississippi]       -8.4373      2.459     -3.432      0.001     -13.257      -3.618
C(week)[T.12]:C(state)[T.Mississippi]       -8.4750      2.412     -3.514      0.000     -13.203      -3.747
C(week)[T.13]:C(state)[T.Mississippi]       -4.0006      2.036     -1.965      0.049      -7.990      -0.011
C(week)[T.14]:C(state)[T.Mississippi]       -5.2576      2.083     -2.523      0.012      -9.341      -1.174
C(week)[T.15]:C(state)[T.Mississippi]       -4.5742      2.048     -2.233      0.026      -8.589      -0.559
C(week)[T.16]:C(state)[T.Mississippi]       -5.5616      2.048     -2.716      0.007      -9.576      -1.547
C(week)[T.17]:C(state)[T.Mississippi]       -3.8096      2.042     -1.866      0.062      -7.812       0.192
C(week)[T.18]:C(state)[T.Mississippi]       -7.5325      2.090     -3.604      0.000     -11.630      -3.435
C(week)[T.19]:C(state)[T.Mississippi]       -3.6367      2.036     -1.786      0.074      -7.627       0.354
C(week)[T.20]:C(state)[T.Mississippi]       -5.5204      2.036     -2.712      0.007      -9.510      -1.530
C(week)[T.21]:C(state)[T.Mississippi]       -4.1042      2.036     -2.016      0.044      -8.095      -0.114
C(week)[T.22]:C(state)[T.Mississippi]       -3.1518      2.036     -1.548      0.122      -7.143       0.839
C(week)[T.23]:C(state)[T.Mississippi]       -9.0621      2.466     -3.675      0.000     -13.895      -4.229
C(week)[T.11]:C(state)[T.Missouri]         -11.6558      2.166     -5.382      0.000     -15.901      -7.411
C(week)[T.12]:C(state)[T.Missouri]          -5.2597      1.637     -3.214      0.001      -8.467      -2.052
C(week)[T.13]:C(state)[T.Missouri]          -9.2843      1.640     -5.660      0.000     -12.499      -6.069
C(week)[T.14]:C(state)[T.Missouri]         -11.5837      1.678     -6.901      0.000     -14.874      -8.294
C(week)[T.15]:C(state)[T.Missouri]          -9.9321      1.658     -5.992      0.000     -13.181      -6.683
C(week)[T.16]:C(state)[T.Missouri]         -10.5613      1.658     -6.372      0.000     -13.810      -7.312
C(week)[T.17]:C(state)[T.Missouri]         -10.6303      1.650     -6.444      0.000     -13.864      -7.397
C(week)[T.18]:C(state)[T.Missouri]          -8.4601      1.639     -5.163      0.000     -11.672      -5.248
C(week)[T.19]:C(state)[T.Missouri]          -1.4878      1.637     -0.909      0.363      -4.696       1.721
C(week)[T.20]:C(state)[T.Missouri]          -7.3204      1.637     -4.472      0.000     -10.529      -4.112
C(week)[T.21]:C(state)[T.Missouri]          -2.1119      1.637     -1.290      0.197      -5.320       1.097
C(week)[T.22]:C(state)[T.Missouri]          -0.4227      1.637     -0.258      0.796      -3.632       2.786
C(week)[T.23]:C(state)[T.Missouri]          -3.7148      1.637     -2.269      0.023      -6.923      -0.506
C(week)[T.11]:C(state)[T.Montana]          -13.3134      3.001     -4.436      0.000     -19.196      -7.430
C(week)[T.12]:C(state)[T.Montana]           -6.4543      2.645     -2.440      0.015     -11.639      -1.270
C(week)[T.13]:C(state)[T.Montana]           -5.6720      2.686     -2.112      0.035     -10.937      -0.407
C(week)[T.14]:C(state)[T.Montana]          -11.8377      2.645     -4.475      0.000     -17.023      -6.653
C(week)[T.15]:C(state)[T.Montana]           -3.9497      2.655     -1.488      0.137      -9.154       1.255
C(week)[T.16]:C(state)[T.Montana]          -10.2231      2.655     -3.850      0.000     -15.427      -5.019
C(week)[T.17]:C(state)[T.Montana]           -9.2410      2.650     -3.487      0.000     -14.436      -4.046
C(week)[T.18]:C(state)[T.Montana]           -7.3912      2.726     -2.711      0.007     -12.734      -2.048
C(week)[T.19]:C(state)[T.Montana]            2.1774      2.769      0.786      0.432      -3.249       7.604
C(week)[T.20]:C(state)[T.Montana]           -3.3564      2.645     -1.269      0.205      -8.542       1.829
C(week)[T.21]:C(state)[T.Montana]           -0.9033      2.645     -0.341      0.733      -6.089       4.282
C(week)[T.22]:C(state)[T.Montana]            4.4514      2.646      1.683      0.092      -0.734       9.637
C(week)[T.23]:C(state)[T.Montana]            4.2726      2.645      1.615      0.106      -0.913       9.458
C(week)[T.11]:C(state)[T.Nevada]            -7.7674      3.132     -2.480      0.013     -13.906      -1.629
C(week)[T.12]:C(state)[T.Nevada]            -0.3736      2.792     -0.134      0.894      -5.846       5.099
C(week)[T.13]:C(state)[T.Nevada]            -1.1334      2.792     -0.406      0.685      -6.606       4.339
C(week)[T.14]:C(state)[T.Nevada]             3.9364      2.793      1.409      0.159      -1.539       9.412
C(week)[T.15]:C(state)[T.Nevada]             7.1188      2.804      2.539      0.011       1.623      12.614
C(week)[T.16]:C(state)[T.Nevada]             3.3267      2.804      1.187      0.235      -2.169       8.822
C(week)[T.17]:C(state)[T.Nevada]             2.9802      2.799      1.065      0.287      -2.506       8.467
C(week)[T.18]:C(state)[T.Nevada]             0.1537      2.795      0.055      0.956      -5.324       5.631
C(week)[T.19]:C(state)[T.Nevada]             2.2659      2.836      0.799      0.424      -3.294       7.826
C(week)[T.20]:C(state)[T.Nevada]            -0.5755      2.836     -0.203      0.839      -6.135       4.984
C(week)[T.21]:C(state)[T.Nevada]             1.2833      2.836      0.452      0.651      -4.276       6.843
C(week)[T.22]:C(state)[T.Nevada]             6.1674      2.836      2.174      0.030       0.608      11.727
C(week)[T.23]:C(state)[T.Nevada]            -0.1564      2.834     -0.055      0.956      -5.712       5.399
C(week)[T.11]:C(state)[T.New Hampshire]     -8.2933      2.809     -2.953      0.003     -13.799      -2.788
C(week)[T.12]:C(state)[T.New Hampshire]     -3.1700      2.424     -1.308      0.191      -7.921       1.582
C(week)[T.13]:C(state)[T.New Hampshire]     -3.2339      2.527     -1.280      0.201      -8.187       1.719
C(week)[T.14]:C(state)[T.New Hampshire]     -2.6094      2.486     -1.050      0.294      -7.481       2.262
C(week)[T.15]:C(state)[T.New Hampshire]      3.5661      2.498      1.428      0.153      -1.330       8.462
C(week)[T.16]:C(state)[T.New Hampshire]     -2.1619      2.498     -0.866      0.387      -7.058       2.734
C(week)[T.17]:C(state)[T.New Hampshire]     -2.5431      2.493     -1.020      0.308      -7.429       2.343
C(week)[T.18]:C(state)[T.New Hampshire]     -4.6195      2.489     -1.856      0.064      -9.499       0.260
C(week)[T.19]:C(state)[T.New Hampshire]     -1.0925      2.539     -0.430      0.667      -6.070       3.885
C(week)[T.20]:C(state)[T.New Hampshire]     -0.5810      2.539     -0.229      0.819      -5.558       4.396
C(week)[T.21]:C(state)[T.New Hampshire]     10.3172      2.482      4.157      0.000       5.452      15.182
C(week)[T.22]:C(state)[T.New Hampshire]     12.9499      2.482      5.217      0.000       8.085      17.815
C(week)[T.23]:C(state)[T.New Hampshire]      3.2726      2.476      1.322      0.186      -1.581       8.127
C(week)[T.11]:C(state)[T.New Jersey]        -8.7542      2.354     -3.718      0.000     -13.369      -4.139
C(week)[T.12]:C(state)[T.New Jersey]        -5.6995      1.940     -2.937      0.003      -9.503      -1.896
C(week)[T.13]:C(state)[T.New Jersey]       -12.8828      1.937     -6.650      0.000     -16.680      -9.086
C(week)[T.14]:C(state)[T.New Jersey]       -13.1259      1.886     -6.961      0.000     -16.822      -9.430
C(week)[T.15]:C(state)[T.New Jersey]       -12.8080      1.906     -6.718      0.000     -16.545      -9.071
C(week)[T.16]:C(state)[T.New Jersey]       -23.4785      1.913    -12.276      0.000     -27.227     -19.730
C(week)[T.17]:C(state)[T.New Jersey]       -25.6699      1.901    -13.503      0.000     -29.396     -21.944
C(week)[T.18]:C(state)[T.New Jersey]       -26.5220      1.903    -13.936      0.000     -30.252     -22.792
C(week)[T.19]:C(state)[T.New Jersey]       -22.6313      1.957    -11.564      0.000     -26.467     -18.795
C(week)[T.20]:C(state)[T.New Jersey]       -23.4519      1.954    -12.000      0.000     -27.283     -19.621
C(week)[T.21]:C(state)[T.New Jersey]       -23.5196      1.952    -12.050      0.000     -27.346     -19.694
C(week)[T.22]:C(state)[T.New Jersey]       -18.4250      1.950     -9.450      0.000     -22.247     -14.603
C(week)[T.23]:C(state)[T.New Jersey]       -24.8976      1.957    -12.720      0.000     -28.734     -21.061
C(week)[T.11]:C(state)[T.New Mexico]        -9.2104      2.618     -3.518      0.000     -14.343      -4.078
C(week)[T.12]:C(state)[T.New Mexico]         0.1959      2.201      0.089      0.929      -4.118       4.509
C(week)[T.13]:C(state)[T.New Mexico]        -4.0250      2.250     -1.789      0.074      -8.435       0.385
C(week)[T.14]:C(state)[T.New Mexico]        -3.6161      2.240     -1.614      0.107      -8.007       0.775
C(week)[T.15]:C(state)[T.New Mexico]        -3.4726      2.255     -1.540      0.124      -7.892       0.947
C(week)[T.16]:C(state)[T.New Mexico]        -9.8810      2.255     -4.382      0.000     -14.301      -5.461
C(week)[T.17]:C(state)[T.New Mexico]        -8.5695      2.249     -3.810      0.000     -12.978      -4.161
C(week)[T.18]:C(state)[T.New Mexico]       -11.8331      2.206     -5.364      0.000     -16.157      -7.509
C(week)[T.19]:C(state)[T.New Mexico]       -13.4787      2.291     -5.884      0.000     -17.969      -8.988
C(week)[T.20]:C(state)[T.New Mexico]       -17.6849      2.204     -8.023      0.000     -22.005     -13.364
C(week)[T.21]:C(state)[T.New Mexico]       -15.9180      2.204     -7.222      0.000     -20.238     -11.598
C(week)[T.22]:C(state)[T.New Mexico]       -14.4150      2.204     -6.540      0.000     -18.735     -10.095
C(week)[T.23]:C(state)[T.New Mexico]        -9.6221      2.202     -4.370      0.000     -13.937      -5.307
C(week)[T.11]:C(state)[T.New York]          -2.8281      1.503     -1.881      0.060      -5.775       0.119
C(week)[T.12]:C(state)[T.New York]          -5.0225      1.579     -3.180      0.001      -8.118      -1.927
C(week)[T.13]:C(state)[T.New York]         -12.1017      1.582     -7.651      0.000     -15.202      -9.001
C(week)[T.14]:C(state)[T.New York]          -8.5139      1.512     -5.632      0.000     -11.477      -5.551
C(week)[T.15]:C(state)[T.New York]         -11.0687      1.548     -7.152      0.000     -14.102      -8.035
C(week)[T.16]:C(state)[T.New York]         -20.6160      1.542    -13.366      0.000     -23.639     -17.593
C(week)[T.17]:C(state)[T.New York]         -22.0917      1.534    -14.398      0.000     -25.099     -19.084
C(week)[T.18]:C(state)[T.New York]         -22.4059      1.525    -14.692      0.000     -25.395     -19.417
C(week)[T.19]:C(state)[T.New York]         -22.0435      1.600    -13.781      0.000     -25.179     -18.908
C(week)[T.20]:C(state)[T.New York]         -22.5458      1.584    -14.238      0.000     -25.650     -19.442
C(week)[T.21]:C(state)[T.New York]         -17.9110      1.583    -11.317      0.000     -21.013     -14.809
C(week)[T.22]:C(state)[T.New York]         -16.9321      1.579    -10.720      0.000     -20.028     -13.836
C(week)[T.23]:C(state)[T.New York]         -21.7905      1.587    -13.732      0.000     -24.901     -18.680
C(week)[T.11]:C(state)[T.North Carolina]     0.8796      1.431      0.615      0.539      -1.925       3.685
C(week)[T.12]:C(state)[T.North Carolina]     1.3622      1.431      0.952      0.341      -1.443       4.167
C(week)[T.13]:C(state)[T.North Carolina]     1.9308      1.431      1.349      0.177      -0.874       4.736
C(week)[T.14]:C(state)[T.North Carolina]    -0.5141      1.436     -0.358      0.720      -3.328       2.300
C(week)[T.15]:C(state)[T.North Carolina]     3.0377      1.460      2.080      0.038       0.176       5.900
C(week)[T.16]:C(state)[T.North Carolina]    -0.2426      1.460     -0.166      0.868      -3.105       2.619
C(week)[T.17]:C(state)[T.North Carolina]    -3.4706      1.451     -2.392      0.017      -6.315      -0.626
C(week)[T.18]:C(state)[T.North Carolina]    -2.0202      1.437     -1.406      0.160      -4.837       0.796
C(week)[T.19]:C(state)[T.North Carolina]     1.3208      1.518      0.870      0.384      -1.654       4.296
C(week)[T.20]:C(state)[T.North Carolina]    -1.9081      1.512     -1.262      0.207      -4.872       1.056
C(week)[T.21]:C(state)[T.North Carolina]    -5.7854      1.514     -3.821      0.000      -8.753      -2.818
C(week)[T.22]:C(state)[T.North Carolina]     0.1099      1.433      0.077      0.939      -2.698       2.918
C(week)[T.23]:C(state)[T.North Carolina]    -2.7223      1.436     -1.896      0.058      -5.536       0.092
C(week)[T.11]:C(state)[T.Ohio]              -4.0020      1.422     -2.815      0.005      -6.788      -1.215
C(week)[T.12]:C(state)[T.Ohio]             -10.0353      1.422     -7.059      0.000     -12.822      -7.249
C(week)[T.13]:C(state)[T.Ohio]              -9.5448      1.502     -6.355      0.000     -12.489      -6.601
C(week)[T.14]:C(state)[T.Ohio]              -4.7021      1.430     -3.289      0.001      -7.504      -1.900
C(week)[T.15]:C(state)[T.Ohio]              -0.6500      1.450     -0.448      0.654      -3.492       2.192
C(week)[T.16]:C(state)[T.Ohio]              -5.6634      1.450     -3.905      0.000      -8.506      -2.821
C(week)[T.17]:C(state)[T.Ohio]              -4.4618      1.443     -3.092      0.002      -7.290      -1.634
C(week)[T.18]:C(state)[T.Ohio]              -4.5764      1.434     -3.191      0.001      -7.388      -1.765
C(week)[T.19]:C(state)[T.Ohio]              -5.7030      1.513     -3.768      0.000      -8.669      -2.736
C(week)[T.20]:C(state)[T.Ohio]              -5.8717      1.506     -3.898      0.000      -8.824      -2.919
C(week)[T.21]:C(state)[T.Ohio]              -1.6961      1.506     -1.126      0.260      -4.649       1.257
C(week)[T.22]:C(state)[T.Ohio]               6.5713      1.505      4.367      0.000       3.622       9.521
C(week)[T.23]:C(state)[T.Ohio]               2.2277      1.505      1.480      0.139      -0.722       5.178
C(week)[T.11]:C(state)[T.Oregon]           -10.8086      2.377     -4.547      0.000     -15.468      -6.149
C(week)[T.12]:C(state)[T.Oregon]            -0.7928      1.907     -0.416      0.678      -4.531       2.946
C(week)[T.13]:C(state)[T.Oregon]            -8.3819      1.980     -4.232      0.000     -12.264      -4.500
C(week)[T.14]:C(state)[T.Oregon]            -4.4954      1.925     -2.336      0.020      -8.268      -0.723
C(week)[T.15]:C(state)[T.Oregon]             2.9373      1.938      1.516      0.130      -0.861       6.736
C(week)[T.16]:C(state)[T.Oregon]            -2.1681      1.938     -1.119      0.263      -5.967       1.631
C(week)[T.17]:C(state)[T.Oregon]            -5.9654      1.931     -3.089      0.002      -9.751      -2.180
C(week)[T.18]:C(state)[T.Oregon]            -7.9041      1.909     -4.141      0.000     -11.646      -4.163
C(week)[T.19]:C(state)[T.Oregon]            -3.5134      1.972     -1.781      0.075      -7.380       0.353
C(week)[T.20]:C(state)[T.Oregon]           -13.3479      1.912     -6.982      0.000     -17.095      -9.601
C(week)[T.21]:C(state)[T.Oregon]            -6.8539      1.912     -3.585      0.000     -10.601      -3.107
C(week)[T.22]:C(state)[T.Oregon]            -0.9735      1.912     -0.509      0.611      -4.721       2.774
C(week)[T.23]:C(state)[T.Oregon]            -2.1452      1.909     -1.124      0.261      -5.887       1.596
C(week)[T.11]:C(state)[T.Pennsylvania]      -9.3314      2.056     -4.540      0.000     -13.361      -5.302
C(week)[T.12]:C(state)[T.Pennsylvania]     -10.0203      1.488     -6.736      0.000     -12.936      -7.104
C(week)[T.13]:C(state)[T.Pennsylvania]     -12.6973      1.501     -8.457      0.000     -15.640      -9.755
C(week)[T.14]:C(state)[T.Pennsylvania]      -9.8816      1.494     -6.616      0.000     -12.809      -6.954
C(week)[T.15]:C(state)[T.Pennsylvania]      -6.9358      1.514     -4.582      0.000      -9.903      -3.969
C(week)[T.16]:C(state)[T.Pennsylvania]     -13.5862      1.514     -8.975      0.000     -16.553     -10.619
C(week)[T.17]:C(state)[T.Pennsylvania]     -15.2320      1.505    -10.121      0.000     -18.182     -12.282
C(week)[T.18]:C(state)[T.Pennsylvania]     -14.5270      1.494     -9.723      0.000     -17.456     -11.598
C(week)[T.19]:C(state)[T.Pennsylvania]     -13.2636      1.570     -8.447      0.000     -16.341     -10.186
C(week)[T.20]:C(state)[T.Pennsylvania]     -14.5365      1.527     -9.521      0.000     -17.529     -11.544
C(week)[T.21]:C(state)[T.Pennsylvania]     -11.7530      1.508     -7.792      0.000     -14.710      -8.796
C(week)[T.22]:C(state)[T.Pennsylvania]      -8.6672      1.497     -5.791      0.000     -11.601      -5.733
C(week)[T.23]:C(state)[T.Pennsylvania]     -11.6765      1.522     -7.670      0.000     -14.661      -8.692
C(week)[T.11]:C(state)[T.Rhode Island]      -7.7242      3.297     -2.342      0.019     -14.188      -1.261
C(week)[T.12]:C(state)[T.Rhode Island]      -8.4937      2.977     -2.853      0.004     -14.329      -2.659
C(week)[T.13]:C(state)[T.Rhode Island]      -7.3698      3.013     -2.446      0.014     -13.276      -1.463
C(week)[T.14]:C(state)[T.Rhode Island]      -9.6077      3.113     -3.086      0.002     -15.710      -3.505
C(week)[T.15]:C(state)[T.Rhode Island]      -3.3503      3.122     -1.073      0.283      -9.469       2.769
C(week)[T.16]:C(state)[T.Rhode Island]     -11.9840      3.122     -3.838      0.000     -18.104      -5.864
C(week)[T.17]:C(state)[T.Rhode Island]     -13.5041      3.119     -4.330      0.000     -19.617      -7.391
C(week)[T.18]:C(state)[T.Rhode Island]     -14.4397      2.979     -4.848      0.000     -20.279      -8.601
C(week)[T.19]:C(state)[T.Rhode Island]     -10.3094      3.154     -3.269      0.001     -16.491      -4.128
C(week)[T.20]:C(state)[T.Rhode Island]      -7.9858      3.020     -2.645      0.008     -13.905      -2.067
C(week)[T.21]:C(state)[T.Rhode Island]      -3.5199      3.019     -1.166      0.244      -9.438       2.398
C(week)[T.22]:C(state)[T.Rhode Island]       3.8640      3.019      1.280      0.201      -2.053       9.781
C(week)[T.23]:C(state)[T.Rhode Island]      -5.2830      2.977     -1.774      0.076     -11.119       0.553
C(week)[T.11]:C(state)[T.South Carolina]    -4.7503      2.238     -2.122      0.034      -9.138      -0.363
C(week)[T.12]:C(state)[T.South Carolina]     2.0422      1.731      1.179      0.238      -1.352       5.436
C(week)[T.13]:C(state)[T.South Carolina]     0.5137      1.732      0.297      0.767      -2.880       3.908
C(week)[T.14]:C(state)[T.South Carolina]     0.0679      1.798      0.038      0.970      -3.457       3.592
C(week)[T.15]:C(state)[T.South Carolina]     0.6570      1.747      0.376      0.707      -2.767       4.081
C(week)[T.16]:C(state)[T.South Carolina]     0.2164      1.747      0.124      0.901      -3.207       3.640
C(week)[T.17]:C(state)[T.South Carolina]    -1.4113      1.739     -0.812      0.417      -4.820       1.998
C(week)[T.18]:C(state)[T.South Carolina]     1.7460      1.732      1.008      0.313      -1.648       5.140
C(week)[T.19]:C(state)[T.South Carolina]     2.6920      1.734      1.552      0.121      -0.707       6.091
C(week)[T.20]:C(state)[T.South Carolina]     1.1019      1.734      0.635      0.525      -2.297       4.500
C(week)[T.21]:C(state)[T.South Carolina]     0.0132      1.734      0.008      0.994      -3.385       3.412
C(week)[T.22]:C(state)[T.South Carolina]     2.9853      1.734      1.722      0.085      -0.413       6.384
C(week)[T.23]:C(state)[T.South Carolina]     2.0404      1.732      1.178      0.239      -1.355       5.435
C(week)[T.11]:C(state)[T.Tennessee]        -11.1442      2.089     -5.335      0.000     -15.239      -7.049
C(week)[T.12]:C(state)[T.Tennessee]         -6.0830      2.089     -2.912      0.004     -10.178      -1.988
C(week)[T.13]:C(state)[T.Tennessee]         -0.4693      1.546     -0.304      0.761      -3.499       2.560
C(week)[T.14]:C(state)[T.Tennessee]          0.0873      1.551      0.056      0.955      -2.952       3.127
C(week)[T.15]:C(state)[T.Tennessee]          2.3772      1.572      1.512      0.130      -0.704       5.459
C(week)[T.16]:C(state)[T.Tennessee]          3.0590      1.572      1.946      0.052      -0.022       6.140
C(week)[T.17]:C(state)[T.Tennessee]          0.4201      1.560      0.269      0.788      -2.637       3.477
C(week)[T.18]:C(state)[T.Tennessee]          5.9365      1.543      3.848      0.000       2.912       8.961
C(week)[T.19]:C(state)[T.Tennessee]          8.8456      1.617      5.471      0.000       5.677      12.015
C(week)[T.20]:C(state)[T.Tennessee]          6.6281      1.609      4.120      0.000       3.475       9.782
C(week)[T.21]:C(state)[T.Tennessee]          2.2201      1.534      1.447      0.148      -0.787       5.227
C(week)[T.22]:C(state)[T.Tennessee]          5.5696      1.535      3.630      0.000       2.562       8.577
C(week)[T.23]:C(state)[T.Tennessee]          3.9628      1.538      2.576      0.010       0.947       6.978
C(week)[T.11]:C(state)[T.Texas]              0.7472      1.369      0.546      0.585      -1.937       3.431
C(week)[T.12]:C(state)[T.Texas]              1.6120      1.369      1.177      0.239      -1.072       4.296
C(week)[T.13]:C(state)[T.Texas]             -0.9178      1.376     -0.667      0.505      -3.615       1.779
C(week)[T.14]:C(state)[T.Texas]             -4.7604      1.381     -3.448      0.001      -7.467      -2.054
C(week)[T.15]:C(state)[T.Texas]             -0.6318      1.402     -0.451      0.652      -3.380       2.117
C(week)[T.16]:C(state)[T.Texas]             -4.3913      1.401     -3.135      0.002      -7.137      -1.645
C(week)[T.17]:C(state)[T.Texas]             -3.7586      1.389     -2.705      0.007      -6.482      -1.035
C(week)[T.18]:C(state)[T.Texas]             -4.1124      1.377     -2.986      0.003      -6.812      -1.413
C(week)[T.19]:C(state)[T.Texas]              1.5114      1.456      1.038      0.299      -1.342       4.365
C(week)[T.20]:C(state)[T.Texas]             -3.1896      1.455     -2.193      0.028      -6.041      -0.338
C(week)[T.21]:C(state)[T.Texas]             -1.3100      1.454     -0.901      0.368      -4.159       1.539
C(week)[T.22]:C(state)[T.Texas]              1.8980      1.454      1.306      0.192      -0.951       4.747
C(week)[T.23]:C(state)[T.Texas]             -3.2165      1.453     -2.214      0.027      -6.064      -0.369
C(week)[T.11]:C(state)[T.Utah]              -6.2516      6.795     -0.920      0.358     -19.570       7.067
C(week)[T.12]:C(state)[T.Utah]             -11.1658      6.795     -1.643      0.100     -24.485       2.153
C(week)[T.13]:C(state)[T.Utah]              -9.0969      6.795     -1.339      0.181     -22.416       4.222
C(week)[T.14]:C(state)[T.Utah]             -10.1373      6.795     -1.492      0.136     -23.457       3.182
C(week)[T.15]:C(state)[T.Utah]              -5.4039      6.799     -0.795      0.427     -18.731       7.923
C(week)[T.16]:C(state)[T.Utah]             -14.5767      6.799     -2.144      0.032     -27.904      -1.249
C(week)[T.17]:C(state)[T.Utah]             -13.6668      6.797     -2.011      0.044     -26.990      -0.343
C(week)[T.18]:C(state)[T.Utah]             -17.3884      6.795     -2.559      0.011     -30.708      -4.069
C(week)[T.19]:C(state)[T.Utah]             -10.5393      6.812     -1.547      0.122     -23.892       2.813
C(week)[T.20]:C(state)[T.Utah]             -14.1930      6.812     -2.083      0.037     -27.546      -0.840
C(week)[T.21]:C(state)[T.Utah]             -13.3373      6.812     -1.958      0.050     -26.690       0.015
C(week)[T.22]:C(state)[T.Utah]              -9.7268      6.812     -1.428      0.153     -23.079       3.626
C(week)[T.23]:C(state)[T.Utah]             -15.3481      6.812     -2.253      0.024     -28.701      -1.995
C(week)[T.11]:C(state)[T.Vermont]          -10.8790      3.516     -3.094      0.002     -17.772      -3.986
C(week)[T.12]:C(state)[T.Vermont]           -9.3681      3.218     -2.911      0.004     -15.675      -3.061
C(week)[T.13]:C(state)[T.Vermont]          -12.9988      3.252     -3.998      0.000     -19.372      -6.625
C(week)[T.14]:C(state)[T.Vermont]          -13.6416      3.219     -4.238      0.000     -19.950      -7.333
C(week)[T.15]:C(state)[T.Vermont]           -8.5999      3.227     -2.665      0.008     -14.926      -2.274
C(week)[T.16]:C(state)[T.Vermont]          -17.6817      3.227     -5.479      0.000     -24.007     -11.356
C(week)[T.17]:C(state)[T.Vermont]          -15.6749      3.223     -4.863      0.000     -21.993      -9.357
C(week)[T.18]:C(state)[T.Vermont]          -19.1814      3.219     -5.958      0.000     -25.492     -12.871
C(week)[T.19]:C(state)[T.Vermont]          -21.4758      3.220     -6.670      0.000     -27.787     -15.164
C(week)[T.20]:C(state)[T.Vermont]          -21.2887      3.220     -6.612      0.000     -27.600     -14.977
C(week)[T.21]:C(state)[T.Vermont]          -11.3970      3.220     -3.540      0.000     -17.708      -5.086
C(week)[T.22]:C(state)[T.Vermont]          -10.1296      3.220     -3.146      0.002     -16.441      -3.818
C(week)[T.23]:C(state)[T.Vermont]          -16.3367      3.218     -5.076      0.000     -22.645     -10.028
C(week)[T.11]:C(state)[T.Virginia]          -7.9819      2.204     -3.622      0.000     -12.301      -3.663
C(week)[T.12]:C(state)[T.Virginia]           2.3851      1.686      1.414      0.157      -0.920       5.690
C(week)[T.13]:C(state)[T.Virginia]           0.3348      1.694      0.198      0.843      -2.987       3.656
C(week)[T.14]:C(state)[T.Virginia]          -0.1011      1.704     -0.059      0.953      -3.441       3.239
C(week)[T.15]:C(state)[T.Virginia]           3.4418      1.738      1.980      0.048       0.035       6.849
C(week)[T.16]:C(state)[T.Virginia]          -4.1296      1.738     -2.376      0.018      -7.537      -0.722
C(week)[T.17]:C(state)[T.Virginia]          -6.8644      1.731     -3.966      0.000     -10.257      -3.472
C(week)[T.18]:C(state)[T.Virginia]          -7.7140      1.713     -4.504      0.000     -11.071      -4.357
C(week)[T.19]:C(state)[T.Virginia]          -5.4289      1.779     -3.052      0.002      -8.915      -1.942
C(week)[T.20]:C(state)[T.Virginia]          -8.4007      1.762     -4.769      0.000     -11.854      -4.948
C(week)[T.21]:C(state)[T.Virginia]         -14.1553      1.686     -8.394      0.000     -17.461     -10.850
C(week)[T.22]:C(state)[T.Virginia]          -7.9869      1.686     -4.736      0.000     -11.292      -4.681
C(week)[T.23]:C(state)[T.Virginia]         -10.3699      1.694     -6.120      0.000     -13.691      -7.049
C(week)[T.11]:C(state)[T.Washington]        -3.4143      1.731     -1.972      0.049      -6.808      -0.020
C(week)[T.12]:C(state)[T.Washington]        -1.5457      1.732     -0.893      0.372      -4.940       1.848
C(week)[T.13]:C(state)[T.Washington]        -4.2337      1.804     -2.347      0.019      -7.769      -0.698
C(week)[T.14]:C(state)[T.Washington]        -2.5391      1.752     -1.450      0.147      -5.973       0.894
C(week)[T.15]:C(state)[T.Washington]         5.0163      1.777      2.823      0.005       1.533       8.499
C(week)[T.16]:C(state)[T.Washington]        -0.7113      1.766     -0.403      0.687      -4.173       2.751
C(week)[T.17]:C(state)[T.Washington]        -3.6265      1.759     -2.062      0.039      -7.074      -0.179
C(week)[T.18]:C(state)[T.Washington]        -6.0277      1.752     -3.440      0.001      -9.462      -2.593
C(week)[T.19]:C(state)[T.Washington]        -0.3548      1.820     -0.195      0.845      -3.922       3.212
C(week)[T.20]:C(state)[T.Washington]        -8.0879      1.810     -4.469      0.000     -11.636      -4.540
C(week)[T.21]:C(state)[T.Washington]        -6.7597      1.810     -3.735      0.000     -10.307      -3.212
C(week)[T.22]:C(state)[T.Washington]        -3.1019      1.800     -1.723      0.085      -6.630       0.426
C(week)[T.23]:C(state)[T.Washington]       -10.4775      1.732     -6.051      0.000     -13.872      -7.083
C(week)[T.11]:C(state)[T.West Virginia]     -9.6174      2.673     -3.599      0.000     -14.856      -4.379
C(week)[T.12]:C(state)[T.West Virginia]    -12.8285      2.673     -4.800      0.000     -18.067      -7.590
C(week)[T.13]:C(state)[T.West Virginia]     -9.1415      2.313     -3.952      0.000     -13.675      -4.608
C(week)[T.14]:C(state)[T.West Virginia]     -6.0208      2.265     -2.658      0.008     -10.460      -1.581
C(week)[T.15]:C(state)[T.West Virginia]     -4.8676      2.276     -2.138      0.032      -9.329      -0.406
C(week)[T.16]:C(state)[T.West Virginia]     -9.8551      2.276     -4.330      0.000     -14.317      -5.393
C(week)[T.17]:C(state)[T.West Virginia]    -10.7408      2.270     -4.731      0.000     -15.191      -6.290
C(week)[T.18]:C(state)[T.West Virginia]    -10.0506      2.265     -4.437      0.000     -14.490      -5.611
C(week)[T.19]:C(state)[T.West Virginia]     -9.6449      2.266     -4.257      0.000     -14.086      -5.204
C(week)[T.20]:C(state)[T.West Virginia]    -10.6775      2.265     -4.713      0.000     -15.118      -6.237
C(week)[T.21]:C(state)[T.West Virginia]     -8.1458      2.265     -3.596      0.000     -12.586      -3.705
C(week)[T.22]:C(state)[T.West Virginia]      0.0986      2.266      0.044      0.965      -4.342       4.540
C(week)[T.23]:C(state)[T.West Virginia]     -1.5352      2.265     -0.678      0.498      -5.975       2.905
C(week)[T.11]:C(state)[T.Wisconsin]        -11.2830      2.105     -5.361      0.000     -15.408      -7.158
C(week)[T.12]:C(state)[T.Wisconsin]        -13.3247      1.555     -8.571      0.000     -16.372     -10.278
C(week)[T.13]:C(state)[T.Wisconsin]        -15.4303      1.628     -9.480      0.000     -18.621     -12.240
C(week)[T.14]:C(state)[T.Wisconsin]        -11.6925      1.569     -7.452      0.000     -14.768      -8.617
C(week)[T.15]:C(state)[T.Wisconsin]         -8.3352      1.601     -5.206      0.000     -11.473      -5.197
C(week)[T.16]:C(state)[T.Wisconsin]        -11.8218      1.601     -7.384      0.000     -14.960      -8.684
C(week)[T.17]:C(state)[T.Wisconsin]        -11.3865      1.593     -7.149      0.000     -14.509      -8.264
C(week)[T.18]:C(state)[T.Wisconsin]        -13.5378      1.585     -8.540      0.000     -16.645     -10.431
C(week)[T.19]:C(state)[T.Wisconsin]        -11.9968      1.654     -7.253      0.000     -15.239      -8.755
C(week)[T.20]:C(state)[T.Wisconsin]        -10.9461      1.649     -6.638      0.000     -14.178      -7.714
C(week)[T.21]:C(state)[T.Wisconsin]         -3.3585      1.576     -2.131      0.033      -6.448      -0.269
C(week)[T.22]:C(state)[T.Wisconsin]          4.5571      1.576      2.891      0.004       1.467       7.647
C(week)[T.23]:C(state)[T.Wisconsin]         -3.5340      1.580     -2.237      0.025      -6.630      -0.438
C(week)[T.11]:C(state)[T.Wyoming]          -11.6149      3.001     -3.870      0.000     -17.498      -5.732
C(week)[T.12]:C(state)[T.Wyoming]           -3.1851      2.645     -1.204      0.229      -8.369       1.999
C(week)[T.13]:C(state)[T.Wyoming]            0.0229      2.771      0.008      0.993      -5.410       5.455
C(week)[T.14]:C(state)[T.Wyoming]           -3.4171      2.734     -1.250      0.211      -8.776       1.942
C(week)[T.15]:C(state)[T.Wyoming]           -0.2442      2.746     -0.089      0.929      -5.627       5.138
C(week)[T.16]:C(state)[T.Wyoming]           -9.0346      2.746     -3.290      0.001     -14.417      -3.652
C(week)[T.17]:C(state)[T.Wyoming]           -4.4956      2.741     -1.640      0.101      -9.869       0.877
C(week)[T.18]:C(state)[T.Wyoming]           -5.5454      2.734     -2.028      0.043     -10.905      -0.186
C(week)[T.19]:C(state)[T.Wyoming]           -1.8779      2.775     -0.677      0.499      -7.317       3.561
C(week)[T.20]:C(state)[T.Wyoming]           -0.6022      2.775     -0.217      0.828      -6.041       4.836
C(week)[T.21]:C(state)[T.Wyoming]            4.0454      2.689      1.504      0.133      -1.226       9.317
C(week)[T.22]:C(state)[T.Wyoming]            8.4989      2.689      3.160      0.002       3.227      13.770
C(week)[T.23]:C(state)[T.Wyoming]            8.1829      2.689      3.043      0.002       2.912      13.454
cases                                       -0.0465      0.006     -8.383      0.000      -0.057      -0.036
deaths                                      -0.5280      0.116     -4.565      0.000      -0.755      -0.301
unemployment                                -0.6994      0.031    -22.535      0.000      -0.760      -0.639
b_rest_sum                                  -7.8955      1.419     -5.566      0.000     -10.676      -5.115
stay                                        -3.1084      0.483     -6.432      0.000      -4.056      -2.161
==============================================================================
Omnibus:                     5786.058   Durbin-Watson:                   0.749
Prob(Omnibus):                  0.000   Jarque-Bera (JB):           174482.837
Skew:                           1.075   Prob(JB):                         0.00
Kurtosis:                      18.813   Cond. No.                     7.58e+17
==============================================================================

Notes:
[1] Standard Errors assume that the covariance matrix of the errors is correctly specified.
[2] The smallest eigenvalue is 4.85e-30. This might indicate that there are
strong multicollinearity problems or that the design matrix is singular.
```

In [86]:

```
import statsmodels.formula.api as smf

reg = smf.ols('Retail ~ cases + deaths + unemployment + C(week) +\
C(week) * C(state) + C(fips_code) + b_rest_sum + stay*conservative',
              data = df).fit()

# print(reg.summary())
pd.DataFrame({'coef' : reg.params[-10:] , 'std err': reg.HC0_se[-10:] , 'tval' : reg.tvalues[-10:] , 'pval' : reg.pvalues[-7:]})
```

Out[86]:

|  | coef | std err | tval | pval |
| --- | --- | --- | --- | --- |
| C(week)[T.21]:C(state)[T.Wyoming] | 3.069452 | 2.555861 | 1.146119 | NaN |
| C(week)[T.22]:C(state)[T.Wyoming] | 7.529814 | 2.802123 | 2.811457 | NaN |
| C(week)[T.23]:C(state)[T.Wyoming] | 7.204870 | 3.215312 | 2.690484 | NaN |
| b\_rest\_sum | -7.838997 | 1.961705 | -5.551552 | 2.880575e-08 |
| cases | -0.044674 | 0.011309 | -8.084673 | 6.717297e-16 |
| conservative | 28.240397 | 0.649890 | 49.549280 | 0.000000e+00 |
| deaths | -0.438178 | 0.159214 | -3.797389 | 1.468177e-04 |
| stay | -8.070145 | 0.682097 | -12.587002 | 3.827573e-36 |
| stay:conservative | 7.655315 | 0.793804 | 11.706301 | 1.634040e-31 |
| unemployment | -0.691033 | 0.045015 | -22.362499 | 5.994554e-109 |

In [87]:

```
print(reg.summary())
```

```
                            OLS Regression Results                            
==============================================================================
Dep. Variable:                 Retail   R-squared:                       0.950
Model:                            OLS   Adj. R-squared:                  0.944
Method:                 Least Squares   F-statistic:                     156.8
Date:                Wed, 12 Apr 2023   Prob (F-statistic):               0.00
Time:                        20:06:58   Log-Likelihood:                -47872.
No. Observations:               16443   AIC:                         9.930e+04
Df Residuals:                   14667   BIC:                         1.130e+05
Df Model:                        1775                                         
Covariance Type:            nonrobust                                         
============================================================================================================
                                               coef    std err          t      P>|t|      [0.025      0.975]
------------------------------------------------------------------------------------------------------------
Intercept                                   -6.1037      1.255     -4.865      0.000      -8.563      -3.644
C(week)[T.11]                                4.4418      1.839      2.416      0.016       0.838       8.045
C(week)[T.12]                              -22.1851      1.839    -12.067      0.000     -25.789     -18.581
C(week)[T.13]                              -33.4196      1.838    -18.179      0.000     -37.023     -29.816
C(week)[T.14]                              -29.1411      1.904    -15.305      0.000     -32.873     -25.409
C(week)[T.15]                              -32.7571      1.929    -16.979      0.000     -36.539     -28.975
C(week)[T.16]                              -19.9209      1.929    -10.326      0.000     -23.702     -16.139
C(week)[T.17]                              -16.6682      1.923     -8.669      0.000     -20.437     -12.899
C(week)[T.18]                               -9.2976      1.908     -4.873      0.000     -13.037      -5.558
C(week)[T.19]                               -8.4085      1.846     -4.555      0.000     -12.026      -4.790
C(week)[T.20]                               -4.7543      1.846     -2.576      0.010      -8.372      -1.137
C(week)[T.21]                               -1.5698      1.846     -0.850      0.395      -5.188       2.048
C(week)[T.22]                               -4.5938      1.846     -2.488      0.013      -8.213      -0.975
C(week)[T.23]                                4.6073      1.842      2.501      0.012       0.996       8.218
C(state)[T.Alaska]                          -0.7351      2.231     -0.330      0.742      -5.107       3.637
C(state)[T.Arizona]                          3.2169      1.838      1.750      0.080      -0.386       6.820
C(state)[T.California]                       1.8257      1.536      1.189      0.234      -1.184       4.836
C(state)[T.Colorado]                         0.9931      1.729      0.575      0.566      -2.395       4.381
C(state)[T.Connecticut]                      2.5319      1.863      1.359      0.174      -1.119       6.183
C(state)[T.Delaware]                         3.7546      2.292      1.638      0.101      -0.737       8.246
C(state)[T.Florida]                         -1.1542      1.568     -0.736      0.462      -4.229       1.920
C(state)[T.Georgia]                         -1.7348      1.586     -1.094      0.274      -4.844       1.374
C(state)[T.Idaho]                           -2.2323      1.862     -1.199      0.231      -5.882       1.417
C(state)[T.Illinois]                         5.1301      1.564      3.281      0.001       2.065       8.195
C(state)[T.Indiana]                          2.8807      1.583      1.820      0.069      -0.222       5.983
C(state)[T.Kansas]                           4.8779      1.678      2.908      0.004       1.590       8.166
C(state)[T.Kentucky]                         3.1931      1.651      1.934      0.053      -0.043       6.429
C(state)[T.Louisiana]                        1.3072      1.694      0.772      0.440      -2.014       4.628
C(state)[T.Maine]                            4.1059      1.790      2.293      0.022       0.597       7.615
C(state)[T.Maryland]                         0.5573      1.683      0.331      0.741      -2.741       3.856
C(state)[T.Massachusetts]                    4.1524      1.769      2.348      0.019       0.685       7.620
C(state)[T.Michigan]                         5.0136      1.577      3.179      0.001       1.922       8.105
C(state)[T.Minnesota]                        2.6433      1.597      1.655      0.098      -0.488       5.775
C(state)[T.Mississippi]                      2.9825      1.770      1.685      0.092      -0.486       6.451
C(state)[T.Missouri]                         5.5313      1.641      3.371      0.001       2.315       8.747
C(state)[T.Montana]                          0.0161      1.970      0.008      0.993      -3.845       3.877
C(state)[T.Nevada]                          -2.5432      2.022     -1.258      0.208      -6.506       1.419
C(state)[T.New Hampshire]                   -0.1583      1.870     -0.085      0.933      -3.823       3.507
C(state)[T.New Jersey]                       1.7274      1.661      1.040      0.298      -1.528       4.983
C(state)[T.New Mexico]                       5.3662      1.798      2.985      0.003       1.843       8.890
C(state)[T.New York]                         5.8743      1.524      3.855      0.000       2.888       8.861
C(state)[T.North Carolina]                   1.6443      1.537      1.070      0.285      -1.368       4.656
C(state)[T.Ohio]                             3.2465      1.555      2.088      0.037       0.199       6.294
C(state)[T.Oregon]                           2.1432      1.691      1.267      0.205      -1.172       5.458
C(state)[T.Pennsylvania]                     2.8702      1.569      1.829      0.067      -0.205       5.946
C(state)[T.Rhode Island]                     4.3972      1.973      2.229      0.026       0.531       8.264
C(state)[T.South Carolina]                  -1.6935      1.657     -1.022      0.307      -4.942       1.555
C(state)[T.Tennessee]                        0.5456      1.612      0.338      0.735      -2.615       3.706
C(state)[T.Texas]                           -0.7846      1.558     -0.504      0.615      -3.838       2.269
C(state)[T.Utah]                             2.1895      2.451      0.893      0.372      -2.615       6.994
C(state)[T.Vermont]                          1.6634      2.103      0.791      0.429      -2.459       5.786
C(state)[T.Virginia]                         0.0779      1.613      0.048      0.961      -3.085       3.240
C(state)[T.Washington]                       2.0254      1.624      1.247      0.212      -1.158       5.209
C(state)[T.West Virginia]                    2.0735      1.841      1.127      0.260      -1.534       5.681
C(state)[T.Wisconsin]                        1.8480      1.570      1.177      0.239      -1.230       4.926
C(state)[T.Wyoming]                         -1.3329      1.994     -0.668      0.504      -5.242       2.576
C(fips_code)[T.01003]                       -7.3177      1.803     -4.058      0.000     -10.853      -3.783
C(fips_code)[T.01009]                       -3.5538      1.808     -1.965      0.049      -7.098      -0.009
C(fips_code)[T.01015]                        0.8891      1.776      0.501      0.617      -2.592       4.371
C(fips_code)[T.01017]                        4.9871      1.875      2.661      0.008       1.313       8.661
C(fips_code)[T.01021]                        1.6657      1.776      0.938      0.348      -1.816       5.148
C(fips_code)[T.01031]                        2.0799      1.795      1.159      0.246      -1.438       5.598
C(fips_code)[T.01033]                       -0.6152      1.796     -0.343      0.732      -4.136       2.905
C(fips_code)[T.01039]                        1.1664      1.922      0.607      0.544      -2.602       4.935
C(fips_code)[T.01043]                       -9.2300      1.824     -5.060      0.000     -12.805      -5.655
C(fips_code)[T.01045]                       -7.6615      1.794     -4.270      0.000     -11.179      -4.144
C(fips_code)[T.01049]                       -4.2142      1.802     -2.339      0.019      -7.746      -0.682
C(fips_code)[T.01051]                       -5.3463      1.811     -2.952      0.003      -8.896      -1.797
C(fips_code)[T.01055]                       -5.2699      1.806     -2.919      0.004      -8.809      -1.731
C(fips_code)[T.01069]                       -8.2355      1.790     -4.601      0.000     -11.744      -4.727
C(fips_code)[T.01071]                       -0.6272      1.785     -0.351      0.725      -4.126       2.871
C(fips_code)[T.01073]                       -8.0718      1.736     -4.650      0.000     -11.474      -4.669
C(fips_code)[T.01077]                       -3.4308      1.785     -1.922      0.055      -6.930       0.068
C(fips_code)[T.01081]                      -11.5355      1.761     -6.550      0.000     -14.988      -8.083
C(fips_code)[T.01083]                       -5.3728      1.814     -2.963      0.003      -8.928      -1.818
C(fips_code)[T.01089]                      -14.7619      1.779     -8.296      0.000     -18.250     -11.274
C(fips_code)[T.01095]                       -1.8682      1.796     -1.040      0.298      -5.389       1.652
C(fips_code)[T.01097]                        1.0948      1.767      0.620      0.536      -2.369       4.558
C(fips_code)[T.01101]                       -4.3081      1.752     -2.459      0.014      -7.742      -0.874
C(fips_code)[T.01103]                       -6.1583      1.795     -3.432      0.001      -9.676      -2.641
C(fips_code)[T.01113]                        6.9934      1.748      4.001      0.000       3.568      10.419
C(fips_code)[T.01115]                       -2.5463      1.812     -1.405      0.160      -6.098       1.005
C(fips_code)[T.01117]                      -14.1506      1.800     -7.863      0.000     -17.678     -10.623
C(fips_code)[T.01121]                        7.3040      1.778      4.108      0.000       3.819      10.789
C(fips_code)[T.01123]                        4.3589      1.794      2.429      0.015       0.842       7.876
C(fips_code)[T.01125]                       -6.2774      1.773     -3.542      0.000      -9.752      -2.803
C(fips_code)[T.01127]                        1.2219      1.785      0.685      0.494      -2.277       4.720
C(fips_code)[T.02020]                       -5.0640      1.212     -4.179      0.000      -7.439      -2.689
C(fips_code)[T.02090]                       -2.1066      1.216     -1.732      0.083      -4.491       0.278
C(fips_code)[T.02122]                        3.9787      1.234      3.225      0.001       1.561       6.397
C(fips_code)[T.02170]                        2.4569      1.242      1.979      0.048       0.023       4.890
C(fips_code)[T.04001]                      -10.0539      1.233     -8.155      0.000     -12.470      -7.637
C(fips_code)[T.04003]                       -7.8535      1.223     -6.421      0.000     -10.251      -5.456
C(fips_code)[T.04005]                        2.9367      1.214      2.419      0.016       0.557       5.316
C(fips_code)[T.04007]                       20.4016      1.220     16.721      0.000      18.010      22.793
C(fips_code)[T.04009]                        2.1313      1.228      1.735      0.083      -0.276       4.539
C(fips_code)[T.04013]                       -5.4149      1.217     -4.450      0.000      -7.800      -3.030
C(fips_code)[T.04015]                        5.0169      1.222      4.104      0.000       2.621       7.413
C(fips_code)[T.04017]                       17.2387      1.222     14.113      0.000      14.844      19.633
C(fips_code)[T.04019]                       -3.3556      1.214     -2.764      0.006      -5.735      -0.976
C(fips_code)[T.04021]                        0.8957      1.221      0.734      0.463      -1.497       3.289
C(fips_code)[T.04023]                      -13.4979      1.217    -11.091      0.000     -15.883     -11.113
C(fips_code)[T.04025]                       -2.3017      1.220     -1.887      0.059      -4.693       0.090
C(fips_code)[T.04027]                       -2.9266      1.246     -2.348      0.019      -5.369      -0.484
C(fips_code)[T.06001]                       -7.4686      1.248     -5.984      0.000      -9.915      -5.022
C(fips_code)[T.06007]                        3.0090      1.248      2.412      0.016       0.563       5.455
C(fips_code)[T.06009]                       -5.5210      1.650     -3.346      0.001      -8.755      -2.287
C(fips_code)[T.06013]                       -6.6704      1.248     -5.346      0.000      -9.116      -4.225
C(fips_code)[T.06017]                      -13.6229      1.249    -10.903      0.000     -16.072     -11.174
C(fips_code)[T.06019]                        3.9562      1.249      3.168      0.002       1.508       6.404
C(fips_code)[T.06021]                        6.0856      2.685      2.266      0.023       0.822      11.349
C(fips_code)[T.06023]                        0.1557      1.248      0.125      0.901      -2.290       2.602
C(fips_code)[T.06025]                        4.3850      1.313      3.339      0.001       1.811       6.959
C(fips_code)[T.06029]                        6.3082      1.255      5.027      0.000       3.849       8.768
C(fips_code)[T.06031]                       10.7792      1.252      8.612      0.000       8.326      13.233
C(fips_code)[T.06033]                       18.0512      1.246     14.487      0.000      15.609      20.494
C(fips_code)[T.06037]                       -0.2207      1.251     -0.176      0.860      -2.674       2.232
C(fips_code)[T.06039]                       -0.1906      1.251     -0.152      0.879      -2.643       2.262
C(fips_code)[T.06041]                       -8.7572      1.254     -6.983      0.000     -11.215      -6.299
C(fips_code)[T.06045]                        7.3086      1.247      5.863      0.000       4.865       9.752
C(fips_code)[T.06047]                       13.0538      1.252     10.427      0.000      10.600      15.508
C(fips_code)[T.06053]                        0.2370      1.252      0.189      0.850      -2.218       2.692
C(fips_code)[T.06055]                       -0.6668      1.246     -0.535      0.593      -3.110       1.776
C(fips_code)[T.06057]                       -5.9683      1.247     -4.787      0.000      -8.412      -3.525
C(fips_code)[T.06059]                      -11.5812      1.249     -9.276      0.000     -14.028      -9.134
C(fips_code)[T.06061]                      -10.5648      1.251     -8.447      0.000     -13.016      -8.113
C(fips_code)[T.06065]                       -2.9834      1.247     -2.392      0.017      -5.428      -0.538
C(fips_code)[T.06067]                       -1.6187      1.247     -1.298      0.194      -4.063       0.826
C(fips_code)[T.06069]                        7.1520      1.248      5.731      0.000       4.706       9.598
C(fips_code)[T.06071]                       -3.5332      1.249     -2.830      0.005      -5.981      -1.086
C(fips_code)[T.06073]                       -7.5569      1.247     -6.061      0.000     -10.001      -5.113
C(fips_code)[T.06075]                      -13.1405      1.263    -10.401      0.000     -15.617     -10.664
C(fips_code)[T.06077]                        4.7858      1.249      3.832      0.000       2.338       7.234
C(fips_code)[T.06079]                       -5.9396      1.248     -4.758      0.000      -8.386      -3.493
C(fips_code)[T.06081]                       -8.8088      1.253     -7.033      0.000     -11.264      -6.354
C(fips_code)[T.06083]                       -7.7830      1.249     -6.234      0.000     -10.230      -5.336
C(fips_code)[T.06085]                      -15.0809      1.252    -12.047      0.000     -17.535     -12.627
C(fips_code)[T.06087]                        3.1893      1.253      2.545      0.011       0.733       5.646
C(fips_code)[T.06089]                       -1.9250      1.253     -1.536      0.125      -4.381       0.531
C(fips_code)[T.06093]                       12.5489      1.251     10.034      0.000      10.098      15.000
C(fips_code)[T.06095]                       -0.6609      1.247     -0.530      0.596      -3.105       1.783
C(fips_code)[T.06097]                        1.9609      1.249      1.570      0.116      -0.487       4.408
C(fips_code)[T.06099]                        2.8646      1.249      2.293      0.022       0.416       5.314
C(fips_code)[T.06101]                       13.4037      1.252     10.708      0.000      10.950      15.857
C(fips_code)[T.06103]                        5.6904      1.250      4.552      0.000       3.240       8.141
C(fips_code)[T.06107]                        4.5910      1.260      3.645      0.000       2.122       7.060
C(fips_code)[T.06109]                        0.7802      1.251      0.624      0.533      -1.672       3.233
C(fips_code)[T.06111]                       -5.7945      1.248     -4.643      0.000      -8.241      -3.348
C(fips_code)[T.06113]                       -1.9065      1.250     -1.525      0.127      -4.356       0.543
C(fips_code)[T.06115]                       19.4937      1.251     15.583      0.000      17.042      21.946
C(fips_code)[T.08001]                        4.8600      1.226      3.963      0.000       2.457       7.264
C(fips_code)[T.08005]                       -1.6249      1.227     -1.325      0.185      -4.030       0.780
C(fips_code)[T.08013]                       -5.2705      1.225     -4.302      0.000      -7.672      -2.869
C(fips_code)[T.08014]                      -10.5918      1.229     -8.617      0.000     -13.001      -8.183
C(fips_code)[T.08031]                       -4.0089      1.224     -3.276      0.001      -6.408      -1.610
C(fips_code)[T.08035]                       -9.1111      1.232     -7.398      0.000     -11.525      -6.697
C(fips_code)[T.08037]                      -15.4398      1.388    -11.122      0.000     -18.161     -12.719
C(fips_code)[T.08041]                       -1.1949      1.232     -0.970      0.332      -3.610       1.220
C(fips_code)[T.08043]                       11.0769      1.238      8.951      0.000       8.651      13.503
C(fips_code)[T.08045]                        2.0069      1.231      1.631      0.103      -0.405       4.419
C(fips_code)[T.08059]                       -1.8151      1.227     -1.480      0.139      -4.220       0.589
C(fips_code)[T.08067]                        0.9994      1.228      0.814      0.416      -1.408       3.406
C(fips_code)[T.08069]                        1.0877      1.226      0.887      0.375      -1.315       3.490
C(fips_code)[T.08077]                        8.1058      1.233      6.577      0.000       5.690      10.522
C(fips_code)[T.08085]                       11.1323      1.228      9.062      0.000       8.724      13.540
C(fips_code)[T.08087]                        2.0367      1.339      1.521      0.128      -0.587       4.661
C(fips_code)[T.08101]                        8.3393      1.229      6.785      0.000       5.930      10.748
C(fips_code)[T.08123]                        0.4049      1.232      0.329      0.743      -2.011       2.821
C(fips_code)[T.09001]                       -3.9864      0.902     -4.418      0.000      -5.755      -2.218
C(fips_code)[T.09003]                       -1.8850      1.195     -1.577      0.115      -4.228       0.458
C(fips_code)[T.09005]                        0.8744      1.206      0.725      0.468      -1.489       3.238
C(fips_code)[T.09007]                       -0.2329      1.197     -0.195      0.846      -2.579       2.113
C(fips_code)[T.09009]                        1.9656      1.194      1.646      0.100      -0.375       4.307
C(fips_code)[T.09011]                       -3.0274      1.200     -2.522      0.012      -5.380      -0.674
C(fips_code)[T.09013]                       -2.4613      1.194     -2.061      0.039      -4.802      -0.120
C(fips_code)[T.09015]                       11.2849      1.202      9.388      0.000       8.929      13.641
C(fips_code)[T.10001]                        4.6528      1.279      3.639      0.000       2.147       7.159
C(fips_code)[T.10003]                       -2.5627      1.270     -2.018      0.044      -5.052      -0.074
C(fips_code)[T.10005]                        1.6646      1.295      1.285      0.199      -0.875       4.204
C(fips_code)[T.12001]                       -4.8030      1.250     -3.844      0.000      -7.252      -2.354
C(fips_code)[T.12005]                       10.1304      1.247      8.126      0.000       7.687      12.574
C(fips_code)[T.12009]                       -2.1016      1.247     -1.686      0.092      -4.545       0.342
C(fips_code)[T.12011]                        1.1892      1.248      0.953      0.341      -1.257       3.636
C(fips_code)[T.12015]                       -8.0777      1.248     -6.475      0.000     -10.523      -5.632
C(fips_code)[T.12017]                        0.6692      1.249      0.536      0.592      -1.779       3.117
C(fips_code)[T.12019]                        1.3431      1.250      1.075      0.282      -1.106       3.792
C(fips_code)[T.12021]                      -15.4345      1.247    -12.375      0.000     -17.879     -12.990
C(fips_code)[T.12023]                        1.6193      1.250      1.296      0.195      -0.830       4.069
C(fips_code)[T.12027]                       -5.5457      1.253     -4.427      0.000      -8.001      -3.090
C(fips_code)[T.12031]                        0.6232      1.247      0.500      0.617      -1.821       3.068
C(fips_code)[T.12033]                        5.4383      1.246      4.363      0.000       2.995       7.881
C(fips_code)[T.12035]                        4.1062      1.247      3.293      0.001       1.662       6.550
C(fips_code)[T.12039]                       13.1278      1.248     10.517      0.000      10.681      15.575
C(fips_code)[T.12051]                        7.5396      1.248      6.043      0.000       5.094       9.985
C(fips_code)[T.12053]                        4.2746      1.248      3.425      0.001       1.828       6.721
C(fips_code)[T.12055]                       -6.9062      1.247     -5.538      0.000      -9.351      -4.462
C(fips_code)[T.12057]                       -2.2204      1.246     -1.782      0.075      -4.663       0.222
C(fips_code)[T.12061]                       -9.3817      1.249     -7.514      0.000     -11.829      -6.934
C(fips_code)[T.12063]                        9.5519      1.250      7.641      0.000       7.102      12.002
C(fips_code)[T.12069]                        0.5030      1.251      0.402      0.688      -1.949       2.956
C(fips_code)[T.12071]                       -9.2537      1.247     -7.423      0.000     -11.697      -6.810
C(fips_code)[T.12073]                        1.1529      1.250      0.923      0.356      -1.296       3.602
C(fips_code)[T.12075]                       12.5982      1.248     10.098      0.000      10.153      15.044
C(fips_code)[T.12081]                       -4.0613      1.246     -3.259      0.001      -6.504      -1.619
C(fips_code)[T.12083]                        2.8069      1.246      2.252      0.024       0.364       5.250
C(fips_code)[T.12085]                      -11.1716      1.248     -8.952      0.000     -13.618      -8.725
C(fips_code)[T.12086]                       -5.0114      1.247     -4.020      0.000      -7.455      -2.568
C(fips_code)[T.12087]                      -20.7905      1.248    -16.658      0.000     -23.237     -18.344
C(fips_code)[T.12089]                        6.0559      1.246      4.858      0.000       3.613       8.499
C(fips_code)[T.12091]                        1.6001      1.249      1.281      0.200      -0.848       4.048
C(fips_code)[T.12093]                       -1.4348      1.250     -1.148      0.251      -3.885       1.016
C(fips_code)[T.12095]                      -13.0496      1.255    -10.395      0.000     -15.510     -10.589
C(fips_code)[T.12097]                       -2.1888      1.278     -1.713      0.087      -4.693       0.315
C(fips_code)[T.12099]                       -1.8831      1.246     -1.511      0.131      -4.326       0.559
C(fips_code)[T.12101]                        4.6431      1.246      3.726      0.000       2.201       7.086
C(fips_code)[T.12103]                        0.0705      1.246      0.057      0.955      -2.372       2.513
C(fips_code)[T.12105]                        4.2016      1.249      3.364      0.001       1.753       6.650
C(fips_code)[T.12107]                       14.5888      1.246     11.708      0.000      12.146      17.031
C(fips_code)[T.12109]                       -3.3745      1.248     -2.703      0.007      -5.821      -0.928
C(fips_code)[T.12111]                        4.2067      1.246      3.375      0.001       1.764       6.649
C(fips_code)[T.12113]                       12.1991      1.249      9.764      0.000       9.750      14.648
C(fips_code)[T.12115]                       -8.3107      1.246     -6.669      0.000     -10.753      -5.868
C(fips_code)[T.12117]                       -0.4655      1.246     -0.374      0.709      -2.908       1.977
C(fips_code)[T.12119]                      -13.4892      1.247    -10.814      0.000     -15.934     -11.044
C(fips_code)[T.12121]                        6.1494      1.250      4.919      0.000       3.699       8.600
C(fips_code)[T.12127]                        5.3103      1.246      4.260      0.000       2.867       7.754
C(fips_code)[T.12129]                        9.5740      1.352      7.082      0.000       6.924      12.224
C(fips_code)[T.12131]                        2.5283      1.249      2.024      0.043       0.080       4.977
C(fips_code)[T.13009]                        6.2157      1.247      4.983      0.000       3.771       8.661
C(fips_code)[T.13013]                        2.6898      1.247      2.156      0.031       0.245       5.135
C(fips_code)[T.13015]                       -2.3393      1.249     -1.873      0.061      -4.787       0.108
C(fips_code)[T.13021]                        0.3305      1.247      0.265      0.791      -2.113       2.774
C(fips_code)[T.13029]                       -3.6498      1.248     -2.925      0.003      -6.095      -1.204
C(fips_code)[T.13031]                       -3.4785      1.247     -2.789      0.005      -5.923      -1.034
C(fips_code)[T.13039]                       -2.6323      1.249     -2.108      0.035      -5.080      -0.184
C(fips_code)[T.13045]                        1.4072      1.248      1.127      0.260      -1.039       3.854
C(fips_code)[T.13047]                       -1.6957      1.250     -1.357      0.175      -4.145       0.754
C(fips_code)[T.13051]                       -0.0471      1.249     -0.038      0.970      -2.496       2.401
C(fips_code)[T.13057]                       -6.4622      1.248     -5.177      0.000      -8.909      -4.015
C(fips_code)[T.13059]                       -8.0976      1.247     -6.493      0.000     -10.542      -5.653
C(fips_code)[T.13063]                       15.5971      1.252     12.459      0.000      13.143      18.051
C(fips_code)[T.13067]                      -10.9789      1.247     -8.806      0.000     -13.423      -8.535
C(fips_code)[T.13069]                        5.9022      1.248      4.729      0.000       3.456       8.348
C(fips_code)[T.13071]                       -2.0262      1.251     -1.620      0.105      -4.478       0.426
C(fips_code)[T.13073]                       -4.7273      1.251     -3.779      0.000      -7.179      -2.275
C(fips_code)[T.13077]                       -4.0706      1.248     -3.263      0.001      -6.516      -1.625
C(fips_code)[T.13089]                        1.8328      1.249      1.467      0.142      -0.615       4.281
C(fips_code)[T.13095]                       -0.8963      1.260     -0.711      0.477      -3.366       1.573
C(fips_code)[T.13097]                        1.7164      1.248      1.376      0.169      -0.729       4.162
C(fips_code)[T.13103]                        2.9113      1.249      2.331      0.020       0.463       5.360
C(fips_code)[T.13113]                       -2.9560      1.247     -2.370      0.018      -5.400      -0.512
C(fips_code)[T.13115]                       -0.6457      1.248     -0.517      0.605      -3.092       1.800
C(fips_code)[T.13117]                      -16.9963      1.251    -13.587      0.000     -19.448     -14.544
C(fips_code)[T.13121]                       -7.7109      1.248     -6.177      0.000     -10.158      -5.264
C(fips_code)[T.13127]                       -3.9189      1.251     -3.132      0.002      -6.371      -1.466
C(fips_code)[T.13129]                        1.0805      1.248      0.866      0.387      -1.366       3.527
C(fips_code)[T.13135]                       -7.9032      1.247     -6.339      0.000     -10.347      -5.459
C(fips_code)[T.13137]                       -5.7392      1.253     -4.580      0.000      -8.195      -3.283
C(fips_code)[T.13139]                       -6.1444      1.250     -4.916      0.000      -8.594      -3.695
C(fips_code)[T.13151]                        1.7570      1.247      1.409      0.159      -0.688       4.202
C(fips_code)[T.13153]                       -2.5328      1.248     -2.030      0.042      -4.979      -0.087
C(fips_code)[T.13157]                       -2.8029      1.251     -2.240      0.025      -5.256      -0.350
C(fips_code)[T.13175]                        2.4340      1.247      1.952      0.051      -0.010       4.878
C(fips_code)[T.13179]                        5.2846      1.247      4.238      0.000       2.840       7.729
C(fips_code)[T.13185]                        3.3196      1.247      2.662      0.008       0.875       5.764
C(fips_code)[T.13213]                        9.0261      1.651      5.466      0.000       5.789      12.263
C(fips_code)[T.13215]                        2.8525      1.247      2.287      0.022       0.407       5.298
C(fips_code)[T.13217]                        8.4645      1.247      6.789      0.000       6.020      10.909
C(fips_code)[T.13223]                       -0.1678      1.248     -0.134      0.893      -2.614       2.279
C(fips_code)[T.13233]                        7.0106      1.248      5.615      0.000       4.563       9.458
C(fips_code)[T.13245]                        2.8450      1.247      2.282      0.023       0.401       5.289
C(fips_code)[T.13247]                        1.5189      1.247      1.218      0.223      -0.926       3.964
C(fips_code)[T.13255]                        4.1843      1.249      3.350      0.001       1.736       6.632
C(fips_code)[T.13275]                       -0.5396      1.250     -0.432      0.666      -2.990       1.911
C(fips_code)[T.13277]                       -0.7001      1.249     -0.561      0.575      -3.148       1.748
C(fips_code)[T.13285]                        3.3648      1.248      2.695      0.007       0.918       5.812
C(fips_code)[T.13295]                        3.8836      1.250      3.106      0.002       1.433       6.335
C(fips_code)[T.13297]                        4.8707      1.248      3.903      0.000       2.424       7.317
C(fips_code)[T.13299]                        4.5542      1.348      3.378      0.001       1.912       7.197
C(fips_code)[T.13313]                        3.0710      1.252      2.452      0.014       0.616       5.526
C(fips_code)[T.16001]                       -7.1579      1.214     -5.895      0.000      -9.538      -4.778
C(fips_code)[T.16005]                        3.4035      1.213      2.806      0.005       1.026       5.781
C(fips_code)[T.16011]                        5.0537      1.222      4.136      0.000       2.659       7.448
C(fips_code)[T.16017]                       13.0130      1.224     10.634      0.000      10.614      15.412
C(fips_code)[T.16019]                        3.6278      1.215      2.985      0.003       1.245       6.010
C(fips_code)[T.16027]                       -0.6459      1.221     -0.529      0.597      -3.040       1.748
C(fips_code)[T.16039]                        5.9494      1.436      4.142      0.000       3.134       8.765
C(fips_code)[T.16055]                        2.4131      1.224      1.972      0.049       0.015       4.812
C(fips_code)[T.16057]                       -7.7550      1.258     -6.163      0.000     -10.222      -5.288
C(fips_code)[T.16065]                      -18.7959      1.231    -15.264      0.000     -21.210     -16.382
C(fips_code)[T.16069]                        0.5891      1.217      0.484      0.628      -1.797       2.975
C(fips_code)[T.16083]                       -1.9272      1.219     -1.581      0.114      -4.317       0.463
C(fips_code)[T.17001]                       -5.9464      1.260     -4.721      0.000      -8.416      -3.477
C(fips_code)[T.17007]                       11.9841      1.264      9.483      0.000       9.507      14.461
C(fips_code)[T.17011]                        5.0270      2.690      1.868      0.062      -0.247      10.301
C(fips_code)[T.17019]                       -8.2256      1.259     -6.533      0.000     -10.694      -5.757
C(fips_code)[T.17021]                        0.7419      2.691      0.276      0.783      -4.534       6.017
C(fips_code)[T.17027]                       14.8830      1.484     10.029      0.000      11.974      17.792
C(fips_code)[T.17029]                        6.3638      1.258      5.061      0.000       3.899       8.829
C(fips_code)[T.17031]                        0.4491      1.259      0.357      0.721      -2.018       2.916
C(fips_code)[T.17037]                        0.1694      1.255      0.135      0.893      -2.291       2.630
C(fips_code)[T.17043]                       -9.6249      1.255     -7.669      0.000     -12.085      -7.165
C(fips_code)[T.17049]                       -6.7248      1.655     -4.063      0.000      -9.969      -3.481
C(fips_code)[T.17055]                        9.8588      1.265      7.794      0.000       7.380      12.338
C(fips_code)[T.17057]                        7.6475      1.255      6.092      0.000       5.187      10.108
C(fips_code)[T.17063]                        6.2900      1.257      5.005      0.000       3.827       8.753
C(fips_code)[T.17073]                        2.1146      1.255      1.685      0.092      -0.346       4.575
C(fips_code)[T.17075]                        1.2001      2.691      0.446      0.656      -4.074       6.474
C(fips_code)[T.17077]                       -4.6189      1.255     -3.680      0.000      -7.079      -2.158
C(fips_code)[T.17081]                       10.9204      1.264      8.638      0.000       8.442      13.398
C(fips_code)[T.17089]                       -4.0424      1.256     -3.220      0.001      -6.503      -1.581
C(fips_code)[T.17091]                        2.5055      1.255      1.996      0.046       0.045       4.966
C(fips_code)[T.17093]                        7.4338      1.256      5.920      0.000       4.973       9.895
C(fips_code)[T.17095]                        3.9554      1.255      3.151      0.002       1.495       6.416
C(fips_code)[T.17097]                       -4.2105      1.256     -3.353      0.001      -6.672      -1.749
C(fips_code)[T.17099]                       -4.6103      1.259     -3.662      0.000      -7.078      -2.142
C(fips_code)[T.17103]                        2.6676      1.356      1.967      0.049       0.010       5.326
C(fips_code)[T.17105]                       -1.3428      1.258     -1.067      0.286      -3.810       1.124
C(fips_code)[T.17107]                        4.5410      2.691      1.687      0.092      -0.735       9.817
C(fips_code)[T.17111]                        0.7204      1.255      0.574      0.566      -1.739       3.180
C(fips_code)[T.17113]                       -8.0528      1.257     -6.407      0.000     -10.517      -5.589
C(fips_code)[T.17115]                       -1.2539      1.258     -0.997      0.319      -3.719       1.212
C(fips_code)[T.17117]                       -6.6472      1.561     -4.258      0.000      -9.707      -3.587
C(fips_code)[T.17119]                       -1.2838      1.256     -1.022      0.307      -3.746       1.178
C(fips_code)[T.17121]                        3.5029      1.259      2.782      0.005       1.035       5.971
C(fips_code)[T.17133]                       -8.3975      2.689     -3.123      0.002     -13.668      -3.127
C(fips_code)[T.17137]                        5.4719      2.691      2.033      0.042       0.197      10.747
C(fips_code)[T.17141]                       -8.2103      1.256     -6.536      0.000     -10.673      -5.748
C(fips_code)[T.17143]                       -7.1634      1.259     -5.691      0.000      -9.631      -4.696
C(fips_code)[T.17157]                        4.0539      2.333      1.738      0.082      -0.518       8.626
C(fips_code)[T.17161]                       -6.9885      1.256     -5.564      0.000      -9.450      -4.527
C(fips_code)[T.17163]                       -1.2817      1.255     -1.021      0.307      -3.742       1.179
C(fips_code)[T.17167]                       -8.1822      1.257     -6.511      0.000     -10.645      -5.719
C(fips_code)[T.17177]                        0.5322      1.257      0.423      0.672      -1.933       2.997
C(fips_code)[T.17179]                       -0.6269      1.257     -0.499      0.618      -3.090       1.836
C(fips_code)[T.17183]                        7.4776      1.256      5.953      0.000       5.016       9.940
C(fips_code)[T.17195]                       -0.8770      1.256     -0.698      0.485      -3.340       1.585
C(fips_code)[T.17197]                       -3.7414      1.256     -2.978      0.003      -6.204      -1.279
C(fips_code)[T.17199]                        1.7154      1.259      1.362      0.173      -0.753       4.184
C(fips_code)[T.17201]                        1.7253      1.263      1.367      0.172      -0.749       4.200
C(fips_code)[T.17203]                       -6.7692      2.333     -2.902      0.004     -11.342      -2.197
C(fips_code)[T.18003]                       -3.6099      1.248     -2.894      0.004      -6.055      -1.165
C(fips_code)[T.18005]                       -1.0204      1.247     -0.818      0.413      -3.464       1.423
C(fips_code)[T.18011]                      -10.8575      1.256     -8.642      0.000     -13.320      -8.395
C(fips_code)[T.18017]                        0.1479      1.265      0.117      0.907      -2.331       2.627
C(fips_code)[T.18019]                       -0.2743      1.247     -0.220      0.826      -2.718       2.169
C(fips_code)[T.18023]                       -2.0057      1.249     -1.606      0.108      -4.454       0.442
C(fips_code)[T.18029]                       -7.1192      1.251     -5.689      0.000      -9.572      -4.666
C(fips_code)[T.18033]                       -0.6234      1.249     -0.499      0.618      -3.072       1.825
C(fips_code)[T.18035]                       -4.6733      1.247     -3.748      0.000      -7.118      -2.229
C(fips_code)[T.18037]                       -2.5713      1.255     -2.049      0.040      -5.031      -0.112
C(fips_code)[T.18039]                        7.7320      1.252      6.174      0.000       5.277      10.187
C(fips_code)[T.18043]                        1.7363      1.247      1.393      0.164      -0.708       4.180
C(fips_code)[T.18053]                        1.7592      1.248      1.409      0.159      -0.688       4.206
C(fips_code)[T.18057]                      -19.3630      1.254    -15.439      0.000     -21.821     -16.905
C(fips_code)[T.18059]                       -2.1433      1.248     -1.717      0.086      -4.590       0.303
C(fips_code)[T.18061]                       -6.8345      1.250     -5.466      0.000      -9.285      -4.384
C(fips_code)[T.18063]                       -9.2397      1.254     -7.367      0.000     -11.698      -6.781
C(fips_code)[T.18065]                        4.9360      1.248      3.954      0.000       2.489       7.383
C(fips_code)[T.18067]                        2.4488      1.279      1.915      0.056      -0.058       4.956
C(fips_code)[T.18069]                       -1.6097      1.250     -1.288      0.198      -4.059       0.840
C(fips_code)[T.18071]                       10.7384      1.253      8.568      0.000       8.282      13.195
C(fips_code)[T.18073]                       -0.6547      1.249     -0.524      0.600      -3.102       1.793
C(fips_code)[T.18077]                        5.6196      1.407      3.993      0.000       2.861       8.378
C(fips_code)[T.18081]                       -7.8079      1.251     -6.244      0.000     -10.259      -5.357
C(fips_code)[T.18083]                        5.0880      1.407      3.617      0.000       2.330       7.845
C(fips_code)[T.18085]                       -2.6901      1.249     -2.153      0.031      -5.139      -0.241
C(fips_code)[T.18087]                        8.9011      1.648      5.400      0.000       5.670      12.132
C(fips_code)[T.18089]                        5.5954      1.250      4.476      0.000       3.145       8.046
C(fips_code)[T.18091]                        6.7824      1.250      5.427      0.000       4.333       9.232
C(fips_code)[T.18093]                        4.1188      1.248      3.300      0.001       1.673       6.565
C(fips_code)[T.18095]                        2.5069      1.249      2.008      0.045       0.059       4.954
C(fips_code)[T.18097]                       -6.2993      1.248     -5.049      0.000      -8.745      -3.854
C(fips_code)[T.18099]                        2.6820      1.249      2.147      0.032       0.233       5.131
C(fips_code)[T.18103]                        7.9446      1.763      4.506      0.000       4.489      11.400
C(fips_code)[T.18105]                      -10.3383      1.253     -8.250      0.000     -12.795      -7.882
C(fips_code)[T.18107]                        6.0701      1.250      4.857      0.000       3.621       8.520
C(fips_code)[T.18109]                        0.9882      1.251      0.790      0.430      -1.464       3.441
C(fips_code)[T.18113]                       10.4216      1.257      8.294      0.000       7.959      12.885
C(fips_code)[T.18127]                       -0.5678      1.247     -0.455      0.649      -3.012       1.877
C(fips_code)[T.18141]                        0.7879      1.248      0.632      0.528      -1.658       3.233
C(fips_code)[T.18145]                        0.3406      1.248      0.273      0.785      -2.106       2.788
C(fips_code)[T.18151]                        9.2412      1.248      7.408      0.000       6.796      11.686
C(fips_code)[T.18157]                       -7.7028      1.248     -6.171      0.000     -10.149      -5.256
C(fips_code)[T.18163]                       -6.8565      1.248     -5.495      0.000      -9.302      -4.411
C(fips_code)[T.18167]                        1.8609      1.247      1.492      0.136      -0.583       4.305
C(fips_code)[T.18173]                        1.3724      1.250      1.098      0.272      -1.078       3.823
C(fips_code)[T.18177]                        1.7349      1.247      1.391      0.164      -0.710       4.180
C(fips_code)[T.18179]                        3.7299      1.763      2.116      0.034       0.275       7.185
C(fips_code)[T.18183]                        2.4583      1.247      1.971      0.049       0.013       4.904
C(fips_code)[T.20015]                        7.4725      1.237      6.041      0.000       5.048       9.897
C(fips_code)[T.20035]                       10.0388      1.235      8.131      0.000       7.619      12.459
C(fips_code)[T.20037]                        0.4591      1.235      0.372      0.710      -1.961       2.879
C(fips_code)[T.20045]                       -2.5730      1.228     -2.095      0.036      -4.980      -0.166
C(fips_code)[T.20055]                       -2.9008      1.250     -2.321      0.020      -5.350      -0.451
C(fips_code)[T.20057]                       -7.1794      1.301     -5.520      0.000      -9.729      -4.630
C(fips_code)[T.20059]                        3.4026      1.279      2.661      0.008       0.896       5.909
C(fips_code)[T.20061]                        2.0412      1.232      1.657      0.097      -0.373       4.455
C(fips_code)[T.20079]                        1.7139      1.230      1.393      0.164      -0.697       4.125
C(fips_code)[T.20091]                      -11.5339      1.231     -9.373      0.000     -13.946      -9.122
C(fips_code)[T.20103]                        1.5452      1.234      1.253      0.210      -0.873       3.963
C(fips_code)[T.20111]                       -1.1084      1.230     -0.901      0.367      -3.519       1.302
C(fips_code)[T.20113]                       -3.6919      1.236     -2.987      0.003      -6.115      -1.269
C(fips_code)[T.20121]                        2.7377      1.236      2.216      0.027       0.316       5.160
C(fips_code)[T.20125]                        6.1079      1.242      4.918      0.000       3.674       8.542
C(fips_code)[T.20155]                        3.9699      1.232      3.223      0.001       1.555       6.385
C(fips_code)[T.20161]                      -12.8143      1.230    -10.417      0.000     -15.226     -10.403
C(fips_code)[T.20169]                        2.3161      1.231      1.882      0.060      -0.097       4.729
C(fips_code)[T.20173]                        2.5624      1.239      2.068      0.039       0.134       4.991
C(fips_code)[T.20177]                        2.9091      1.230      2.365      0.018       0.498       5.320
C(fips_code)[T.20209]                       -0.5968      0.898     -0.664      0.506      -2.357       1.164
C(fips_code)[T.21015]                      -10.5262      0.754    -13.968      0.000     -12.003      -9.049
C(fips_code)[T.21019]                        0.0536      1.238      0.043      0.965      -2.373       2.481
C(fips_code)[T.21029]                        2.2576      1.240      1.820      0.069      -0.174       4.689
C(fips_code)[T.21035]                       -3.1932      1.885     -1.694      0.090      -6.888       0.501
C(fips_code)[T.21037]                       -8.0534      1.238     -6.506      0.000     -10.480      -5.627
C(fips_code)[T.21047]                        9.9949      1.238      8.076      0.000       7.569      12.421
C(fips_code)[T.21049]                       -0.1155      1.541     -0.075      0.940      -3.135       2.904
C(fips_code)[T.21059]                       -0.8885      1.237     -0.718      0.472      -3.313       1.536
C(fips_code)[T.21067]                       -6.1261      1.237     -4.953      0.000      -8.551      -3.702
C(fips_code)[T.21073]                        2.6935      1.237      2.178      0.029       0.269       5.118
C(fips_code)[T.21083]                       -2.0044      1.464     -1.369      0.171      -4.875       0.866
C(fips_code)[T.21093]                       -1.4752      1.239     -1.191      0.234      -3.903       0.953
C(fips_code)[T.21101]                        4.4122      1.237      3.568      0.000       1.988       6.836
C(fips_code)[T.21107]                       13.4074      1.240     10.812      0.000      10.977      15.838
C(fips_code)[T.21111]                       -2.0106      1.236     -1.627      0.104      -4.433       0.412
C(fips_code)[T.21113]                       -1.8669      1.240     -1.506      0.132      -4.298       0.564
C(fips_code)[T.21117]                       -3.7409      1.238     -3.021      0.003      -6.168      -1.314
C(fips_code)[T.21125]                        5.5857      1.240      4.506      0.000       3.156       8.015
C(fips_code)[T.21145]                       -5.0819      1.239     -4.101      0.000      -7.511      -2.653
C(fips_code)[T.21151]                       -1.4256      1.239     -1.151      0.250      -3.854       1.002
C(fips_code)[T.21179]                        5.0096      1.239      4.042      0.000       2.580       7.439
C(fips_code)[T.21185]                       -1.3502      1.239     -1.090      0.276      -3.779       1.079
C(fips_code)[T.21199]                        6.8320      1.240      5.511      0.000       4.402       9.262
C(fips_code)[T.21209]                        2.8959      1.238      2.338      0.019       0.469       5.323
C(fips_code)[T.21211]                       -3.1356      1.238     -2.532      0.011      -5.563      -0.708
C(fips_code)[T.21227]                        1.0449      1.238      0.844      0.399      -1.381       3.471
C(fips_code)[T.22001]                        4.5467      1.240      3.668      0.000       2.117       6.977
C(fips_code)[T.22005]                       -5.0037      1.238     -4.043      0.000      -7.430      -2.578
C(fips_code)[T.22015]                      -10.2902      1.240     -8.298      0.000     -12.721      -7.859
C(fips_code)[T.22017]                        4.0512      1.236      3.278      0.001       1.629       6.473
C(fips_code)[T.22019]                       -0.7242      0.892     -0.812      0.417      -2.472       1.024
C(fips_code)[T.22033]                       -2.5848      1.234     -2.094      0.036      -5.004      -0.165
C(fips_code)[T.22045]                       13.7572      1.236     11.129      0.000      11.334      16.180
C(fips_code)[T.22051]                       -4.7322      1.239     -3.820      0.000      -7.161      -2.304
C(fips_code)[T.22055]                       -6.2588      1.238     -5.055      0.000      -8.686      -3.832
C(fips_code)[T.22057]                        2.6196      1.239      2.114      0.035       0.191       5.049
C(fips_code)[T.22063]                       -3.8725      1.242     -3.118      0.002      -6.307      -1.438
C(fips_code)[T.22071]                      -11.2544      1.245     -9.040      0.000     -13.695      -8.814
C(fips_code)[T.22073]                       -1.5752      1.237     -1.274      0.203      -4.000       0.849
C(fips_code)[T.22079]                       -0.4846      1.239     -0.391      0.696      -2.914       1.945
C(fips_code)[T.22087]                        5.4549      1.242      4.391      0.000       3.020       7.890
C(fips_code)[T.22089]                      -13.3573      1.239    -10.781      0.000     -15.786     -10.929
C(fips_code)[T.22095]                        7.9459      1.248      6.366      0.000       5.500      10.392
C(fips_code)[T.22097]                        7.3314      1.237      5.928      0.000       4.907       9.755
C(fips_code)[T.22099]                        6.5289      1.235      5.287      0.000       4.108       8.949
C(fips_code)[T.22101]                        4.4821      1.236      3.625      0.000       2.059       6.906
C(fips_code)[T.22103]                       -8.4927      1.239     -6.855      0.000     -10.921      -6.064
C(fips_code)[T.22105]                        7.0520      1.240      5.685      0.000       4.621       9.483
C(fips_code)[T.22109]                       -8.3694      1.239     -6.757      0.000     -10.797      -5.941
C(fips_code)[T.22113]                       10.4646      1.239      8.448      0.000       8.037      12.893
C(fips_code)[T.22115]                       -6.4211      1.241     -5.176      0.000      -8.853      -3.989
C(fips_code)[T.22119]                       10.4939      1.633      6.427      0.000       7.294      13.694
C(fips_code)[T.23001]                       -3.6630      1.250     -2.930      0.003      -6.113      -1.213
C(fips_code)[T.23003]                       -3.6938      1.256     -2.940      0.003      -6.156      -1.231
C(fips_code)[T.23005]                       -9.0473      1.246     -7.263      0.000     -11.489      -6.606
C(fips_code)[T.23009]                        7.4934      1.248      6.003      0.000       5.046       9.940
C(fips_code)[T.23011]                       -0.8459      1.249     -0.677      0.498      -3.294       1.602
C(fips_code)[T.23013]                        5.2399      2.027      2.585      0.010       1.267       9.213
C(fips_code)[T.23015]                        1.0412      2.267      0.459      0.646      -3.403       5.485
C(fips_code)[T.23017]                        1.9608      1.251      1.568      0.117      -0.491       4.412
C(fips_code)[T.23019]                       -5.1296      1.250     -4.103      0.000      -7.580      -2.679
C(fips_code)[T.23023]                       -0.3032      1.856     -0.163      0.870      -3.941       3.335
C(fips_code)[T.23025]                       10.2770      1.252      8.209      0.000       7.823      12.731
C(fips_code)[T.23027]                        5.9300      1.617      3.668      0.000       2.761       9.099
C(fips_code)[T.23031]                       -5.1536      1.252     -4.115      0.000      -7.608      -2.699
C(fips_code)[T.24001]                       -4.1815      1.237     -3.380      0.001      -6.606      -1.757
C(fips_code)[T.24003]                       -9.7426      1.232     -7.910      0.000     -12.157      -7.328
C(fips_code)[T.24005]                        0.0215      1.229      0.018      0.986      -2.388       2.431
C(fips_code)[T.24009]                       -4.3285      1.237     -3.500      0.000      -6.753      -1.904
C(fips_code)[T.24011]                        2.0040      1.535      1.306      0.192      -1.004       5.012
C(fips_code)[T.24013]                       -6.7076      1.238     -5.420      0.000      -9.133      -4.282
C(fips_code)[T.24015]                        6.7263      1.234      5.449      0.000       4.307       9.146
C(fips_code)[T.24017]                        3.8510      1.229      3.133      0.002       1.442       6.260
C(fips_code)[T.24019]                        7.7749      1.281      6.069      0.000       5.264      10.286
C(fips_code)[T.24021]                       -6.3704      1.231     -5.176      0.000      -8.783      -3.958
C(fips_code)[T.24025]                       -5.3141      1.233     -4.308      0.000      -7.732      -2.896
C(fips_code)[T.24027]                       -9.2044      1.229     -7.487      0.000     -11.614      -6.795
C(fips_code)[T.24031]                       -4.2303      1.228     -3.445      0.001      -6.637      -1.823
C(fips_code)[T.24033]                       12.0787      1.228      9.836      0.000       9.672      14.486
C(fips_code)[T.24035]                       -9.5316      1.239     -7.695      0.000     -11.960      -7.104
C(fips_code)[T.24037]                       -1.7012      1.234     -1.379      0.168      -4.119       0.717
C(fips_code)[T.24043]                       -1.3634      1.232     -1.106      0.269      -3.779       1.052
C(fips_code)[T.24045]                       -4.3478      1.235     -3.520      0.000      -6.769      -1.927
C(fips_code)[T.24047]                       26.3836      1.252     21.075      0.000      23.930      28.837
C(fips_code)[T.24510]                        8.7405      1.227      7.121      0.000       6.335      11.146
C(fips_code)[T.25001]                        4.3343      1.223      3.544      0.000       1.937       6.732
C(fips_code)[T.25003]                        9.7055      1.213      8.002      0.000       7.328      12.083
C(fips_code)[T.25005]                        5.3895      1.222      4.412      0.000       2.995       7.784
C(fips_code)[T.25009]                        0.8623      1.217      0.708      0.479      -1.524       3.248
C(fips_code)[T.25011]                        3.5845      1.216      2.948      0.003       1.201       5.968
C(fips_code)[T.25013]                        3.0022      1.220      2.460      0.014       0.610       5.394
C(fips_code)[T.25015]                        0.5683      1.213      0.468      0.640      -1.810       2.947
C(fips_code)[T.25017]                       -8.5314      1.215     -7.024      0.000     -10.912      -6.151
C(fips_code)[T.25021]                       -5.9016      1.216     -4.853      0.000      -8.285      -3.518
C(fips_code)[T.25023]                        2.6562      1.219      2.178      0.029       0.266       5.046
C(fips_code)[T.25025]                      -11.1566      1.209     -9.226      0.000     -13.527      -8.786
C(fips_code)[T.25027]                       -0.3607      1.218     -0.296      0.767      -2.748       2.027
C(fips_code)[T.26005]                        6.5883      1.246      5.287      0.000       4.146       9.031
C(fips_code)[T.26015]                        2.1165      1.248      1.696      0.090      -0.329       4.563
C(fips_code)[T.26017]                       -2.2617      1.246     -1.815      0.069      -4.704       0.180
C(fips_code)[T.26021]                        5.9052      1.246      4.740      0.000       3.463       8.347
C(fips_code)[T.26023]                        2.2942      1.248      1.839      0.066      -0.151       4.740
C(fips_code)[T.26025]                        6.4123      1.245      5.152      0.000       3.973       8.852
C(fips_code)[T.26027]                       -1.8249      1.647     -1.108      0.268      -5.052       1.402
C(fips_code)[T.26037]                       -4.9645      1.250     -3.971      0.000      -7.415      -2.514
C(fips_code)[T.26045]                       -7.4438      1.247     -5.970      0.000      -9.888      -5.000
C(fips_code)[T.26049]                        5.2482      1.250      4.199      0.000       2.798       7.698
C(fips_code)[T.26055]                       -3.0348      1.245     -2.438      0.015      -5.475      -0.595
C(fips_code)[T.26057]                        2.3029      1.246      1.848      0.065      -0.140       4.745
C(fips_code)[T.26059]                        7.0017      1.247      5.614      0.000       4.557       9.446
C(fips_code)[T.26065]                      -11.1095      1.249     -8.893      0.000     -13.558      -8.661
C(fips_code)[T.26067]                        6.1507      1.247      4.934      0.000       3.707       8.594
C(fips_code)[T.26073]                       -3.5961      1.246     -2.887      0.004      -6.038      -1.155
C(fips_code)[T.26075]                        0.1576      1.245      0.127      0.899      -2.283       2.598
C(fips_code)[T.26077]                       -8.7430      1.251     -6.990      0.000     -11.195      -6.291
C(fips_code)[T.26081]                      -11.2787      1.247     -9.047      0.000     -13.722      -8.835
C(fips_code)[T.26087]                        7.4881      1.255      5.966      0.000       5.028       9.948
C(fips_code)[T.26091]                       -1.4448      1.247     -1.159      0.247      -3.889       1.000
C(fips_code)[T.26093]                      -14.3772      1.247    -11.534      0.000     -16.821     -11.934
C(fips_code)[T.26099]                       -4.2816      1.252     -3.419      0.001      -6.736      -1.827
C(fips_code)[T.26103]                       -8.9490      1.246     -7.181      0.000     -11.392      -6.506
C(fips_code)[T.26107]                        3.7223      1.474      2.525      0.012       0.833       6.611
C(fips_code)[T.26111]                      -12.3287      1.249     -9.870      0.000     -14.777      -9.880
C(fips_code)[T.26115]                       -5.8829      1.247     -4.717      0.000      -8.328      -3.438
C(fips_code)[T.26117]                        8.1181      1.248      6.505      0.000       5.672      10.564
C(fips_code)[T.26121]                        8.1918      1.249      6.559      0.000       5.744      10.640
C(fips_code)[T.26123]                        6.6493      1.247      5.332      0.000       4.205       9.094
C(fips_code)[T.26125]                      -13.6628      1.247    -10.956      0.000     -16.107     -11.218
C(fips_code)[T.26139]                       -8.1380      1.249     -6.514      0.000     -10.587      -5.689
C(fips_code)[T.26145]                       -2.0885      1.245     -1.677      0.093      -4.529       0.352
C(fips_code)[T.26147]                        2.0467      1.252      1.635      0.102      -0.408       4.501
C(fips_code)[T.26149]                       16.7711      1.245     13.466      0.000      14.330      19.212
C(fips_code)[T.26151]                        5.0033      1.554      3.220      0.001       1.958       8.049
C(fips_code)[T.26155]                        5.3061      1.248      4.251      0.000       2.859       7.753
C(fips_code)[T.26157]                        3.8479      1.252      3.073      0.002       1.394       6.302
C(fips_code)[T.26159]                       12.8638      1.247     10.319      0.000      10.420      15.307
C(fips_code)[T.26161]                      -12.7346      1.257    -10.130      0.000     -15.199     -10.271
C(fips_code)[T.26163]                        3.7744      1.256      3.006      0.003       1.313       6.236
C(fips_code)[T.26165]                       15.1983      1.552      9.792      0.000      12.156      18.241
C(fips_code)[T.27003]                       -2.1101      1.244     -1.696      0.090      -4.548       0.328
C(fips_code)[T.27005]                        5.3409      1.343      3.978      0.000       2.709       7.973
C(fips_code)[T.27007]                        4.5451      1.244      3.655      0.000       2.108       6.983
C(fips_code)[T.27009]                       -0.2002      1.244     -0.161      0.872      -2.639       2.239
C(fips_code)[T.27013]                      -11.0959      1.243     -8.926      0.000     -13.533      -8.659
C(fips_code)[T.27019]                       -2.4989      1.243     -2.010      0.044      -4.935      -0.062
C(fips_code)[T.27025]                       -8.0725      1.246     -6.480      0.000     -10.514      -5.631
C(fips_code)[T.27027]                        2.2151      1.246      1.777      0.076      -0.228       4.658
C(fips_code)[T.27035]                        5.7665      1.245      4.633      0.000       3.327       8.206
C(fips_code)[T.27037]                       -2.8787      1.243     -2.316      0.021      -5.315      -0.443
C(fips_code)[T.27041]                        0.3489      1.244      0.280      0.779      -2.090       2.787
C(fips_code)[T.27047]                        1.8136      1.244      1.458      0.145      -0.624       4.251
C(fips_code)[T.27049]                        2.1104      1.244      1.697      0.090      -0.328       4.549
C(fips_code)[T.27053]                       -6.2300      1.242     -5.014      0.000      -8.665      -3.795
C(fips_code)[T.27059]                        1.5897      1.246      1.276      0.202      -0.853       4.032
C(fips_code)[T.27061]                        2.6447      1.248      2.119      0.034       0.198       5.091
C(fips_code)[T.27067]                       -3.0839      1.245     -2.476      0.013      -5.525      -0.643
C(fips_code)[T.27079]                       53.9130      2.078     25.946      0.000      49.840      57.986
C(fips_code)[T.27085]                        0.6949      1.245      0.558      0.577      -1.744       3.134
C(fips_code)[T.27097]                        8.4333      1.290      6.538      0.000       5.905      10.962
C(fips_code)[T.27099]                       -6.1041      1.246     -4.898      0.000      -8.547      -3.661
C(fips_code)[T.27103]                       -4.7514      1.548     -3.069      0.002      -7.786      -1.716
C(fips_code)[T.27109]                       -7.8698      1.242     -6.336      0.000     -10.305      -5.435
C(fips_code)[T.27111]                        2.5343      1.245      2.036      0.042       0.095       4.974
C(fips_code)[T.27119]                       -7.3196      1.549     -4.726      0.000     -10.356      -4.284
C(fips_code)[T.27123]                       -5.8489      1.242     -4.709      0.000      -8.284      -3.414
C(fips_code)[T.27131]                       -1.2429      1.243     -1.000      0.317      -3.680       1.194
C(fips_code)[T.27137]                       -7.2298      1.245     -5.809      0.000      -9.669      -4.790
C(fips_code)[T.27139]                       -7.3468      1.245     -5.903      0.000      -9.786      -4.907
C(fips_code)[T.27141]                        7.5527      1.244      6.069      0.000       5.113       9.992
C(fips_code)[T.27145]                      -10.1437      1.246     -8.143      0.000     -12.586      -7.702
C(fips_code)[T.27147]                       -0.4778      1.244     -0.384      0.701      -2.917       1.961
C(fips_code)[T.27163]                       -0.2540      1.242     -0.205      0.838      -2.689       2.181
C(fips_code)[T.27169]                        1.9141      1.243      1.540      0.124      -0.522       4.350
C(fips_code)[T.27171]                       -4.0147      1.245     -3.224      0.001      -6.456      -1.573
C(fips_code)[T.28033]                       -4.1414      1.237     -3.348      0.001      -6.566      -1.717
C(fips_code)[T.28035]                        2.8163      1.232      2.286      0.022       0.401       5.231
C(fips_code)[T.28045]                        6.7338      1.236      5.448      0.000       4.311       9.157
C(fips_code)[T.28047]                        2.0793      1.241      1.675      0.094      -0.353       4.512
C(fips_code)[T.28049]                       11.5877      1.232      9.406      0.000       9.173      14.002
C(fips_code)[T.28059]                        2.6376      1.243      2.122      0.034       0.201       5.075
C(fips_code)[T.28067]                       -1.4668      1.245     -1.178      0.239      -3.907       0.973
C(fips_code)[T.28071]                       -4.2551      2.679     -1.588      0.112      -9.506       0.996
C(fips_code)[T.28073]                       -8.7874      1.236     -7.109      0.000     -11.210      -6.365
C(fips_code)[T.28075]                       -2.5062      1.238     -2.024      0.043      -4.934      -0.079
C(fips_code)[T.28081]                       -6.5767      1.240     -5.305      0.000      -9.006      -4.147
C(fips_code)[T.28087]                       -4.4457      1.235     -3.599      0.000      -6.867      -2.024
C(fips_code)[T.28089]                       -0.0249      1.233     -0.020      0.984      -2.442       2.392
C(fips_code)[T.28109]                       -0.2196      1.243     -0.177      0.860      -2.655       2.216
C(fips_code)[T.28121]                      -10.5129      1.240     -8.476      0.000     -12.944      -8.082
C(fips_code)[T.28149]                        6.4610      1.236      5.228      0.000       4.039       8.883
C(fips_code)[T.28151]                       13.6036      1.233     11.036      0.000      11.187      16.020
C(fips_code)[T.29009]                        3.6423      1.245      2.926      0.003       1.202       6.082
C(fips_code)[T.29019]                       -9.7068      1.244     -7.803      0.000     -12.145      -7.269
C(fips_code)[T.29021]                       -1.5039      1.243     -1.210      0.226      -3.941       0.933
C(fips_code)[T.29023]                        7.3237      1.243      5.892      0.000       4.887       9.760
C(fips_code)[T.29027]                       -4.9945      1.245     -4.011      0.000      -7.435      -2.554
C(fips_code)[T.29029]                       19.1429      1.243     15.406      0.000      16.707      21.578
C(fips_code)[T.29031]                      -10.0537      1.244     -8.079      0.000     -12.493      -7.614
C(fips_code)[T.29037]                        0.7360      1.244      0.592      0.554      -1.703       3.175
C(fips_code)[T.29043]                        0.6552      1.244      0.527      0.598      -1.783       3.093
C(fips_code)[T.29047]                       -2.3636      1.242     -1.903      0.057      -4.798       0.071
C(fips_code)[T.29051]                       -3.4638      1.245     -2.783      0.005      -5.904      -1.024
C(fips_code)[T.29071]                        0.6354      1.243      0.511      0.609      -1.801       3.071
C(fips_code)[T.29077]                       -4.4729      1.242     -3.602      0.000      -6.907      -2.039
C(fips_code)[T.29091]                        1.5406      1.243      1.240      0.215      -0.895       3.976
C(fips_code)[T.29095]                       -3.4008      1.241     -2.739      0.006      -5.834      -0.967
C(fips_code)[T.29097]                        1.5357      1.243      1.235      0.217      -0.901       3.972
C(fips_code)[T.29099]                       -1.0423      1.243     -0.839      0.402      -3.478       1.394
C(fips_code)[T.29101]                       -0.7740      1.242     -0.623      0.533      -3.208       1.660
C(fips_code)[T.29105]                       10.9533      1.246      8.791      0.000       8.511      13.396
C(fips_code)[T.29109]                       12.7501      1.243     10.259      0.000      10.314      15.186
C(fips_code)[T.29113]                       -0.0040      1.242     -0.003      0.997      -2.438       2.430
C(fips_code)[T.29127]                        1.0553      1.243      0.849      0.396      -1.382       3.493
C(fips_code)[T.29145]                        0.9989      1.244      0.803      0.422      -1.439       3.436
C(fips_code)[T.29159]                        5.6919      1.242      4.583      0.000       3.258       8.126
C(fips_code)[T.29161]                       -5.1606      1.244     -4.149      0.000      -7.599      -2.722
C(fips_code)[T.29165]                      -18.6078      1.242    -14.976      0.000     -21.043     -16.172
C(fips_code)[T.29167]                        1.3116      1.245      1.054      0.292      -1.129       3.752
C(fips_code)[T.29169]                       -1.6258      1.242     -1.309      0.190      -4.060       0.808
C(fips_code)[T.29183]                      -11.1506      1.241     -8.983      0.000     -13.584      -8.718
C(fips_code)[T.29187]                        5.6325      1.243      4.530      0.000       3.195       8.070
C(fips_code)[T.29189]                       -8.6062      1.242     -6.931      0.000     -11.040      -6.172
C(fips_code)[T.29213]                        9.4511      1.283      7.369      0.000       6.937      11.965
C(fips_code)[T.29219]                       -0.1316      1.244     -0.106      0.916      -2.570       2.306
C(fips_code)[T.29225]                       13.8850      1.242     11.179      0.000      11.450      16.320
C(fips_code)[T.29510]                       -4.3474      1.245     -3.491      0.000      -6.789      -1.906
C(fips_code)[T.30013]                        2.7336      1.205      2.269      0.023       0.372       5.095
C(fips_code)[T.30029]                        1.0473      1.212      0.864      0.387      -1.327       3.422
C(fips_code)[T.30031]                      -15.8296      1.206    -13.122      0.000     -18.194     -13.465
C(fips_code)[T.30049]                        3.4536      1.205      2.866      0.004       1.092       5.815
C(fips_code)[T.30063]                       -3.6979      1.198     -3.088      0.002      -6.045      -1.351
C(fips_code)[T.30081]                        6.1270      1.298      4.722      0.000       3.583       8.671
C(fips_code)[T.30093]                        4.1210      1.207      3.413      0.001       1.754       6.488
C(fips_code)[T.30111]                        2.0610      1.205      1.710      0.087      -0.301       4.423
C(fips_code)[T.32003]                       -3.0027      1.227     -2.447      0.014      -5.408      -0.598
C(fips_code)[T.32005]                      -17.1342      1.212    -14.132      0.000     -19.511     -14.758
C(fips_code)[T.32007]                       -3.7328      1.210     -3.086      0.002      -6.104      -1.362
C(fips_code)[T.32019]                        9.7779      1.205      8.113      0.000       7.416      12.140
C(fips_code)[T.32023]                       11.2238      1.198      9.371      0.000       8.876      13.571
C(fips_code)[T.32031]                       -2.2765      1.194     -1.907      0.057      -4.617       0.064
C(fips_code)[T.32510]                        2.6013      1.198      2.171      0.030       0.253       4.950
C(fips_code)[T.33001]                        4.0846      1.228      3.327      0.001       1.678       6.491
C(fips_code)[T.33003]                       -0.2546      1.223     -0.208      0.835      -2.651       2.142
C(fips_code)[T.33005]                        1.3081      1.219      1.073      0.283      -1.081       3.697
C(fips_code)[T.33007]                        1.5430      1.838      0.839      0.401      -2.060       5.146
C(fips_code)[T.33009]                       -5.0203      1.215     -4.133      0.000      -7.401      -2.639
C(fips_code)[T.33011]                      -10.0686      1.220     -8.253      0.000     -12.460      -7.677
C(fips_code)[T.33013]                       -3.2385      1.216     -2.662      0.008      -5.623      -0.854
C(fips_code)[T.33015]                       -4.4299      1.220     -3.632      0.000      -6.821      -2.039
C(fips_code)[T.33017]                        1.7190      1.216      1.413      0.158      -0.665       4.103
C(fips_code)[T.33019]                       14.1989      1.218     11.655      0.000      11.811      16.587
C(fips_code)[T.34001]                        2.9411      1.293      2.275      0.023       0.408       5.475
C(fips_code)[T.34003]                       -8.9842      1.232     -7.294      0.000     -11.399      -6.570
C(fips_code)[T.34005]                       -3.5702      1.233     -2.895      0.004      -5.988      -1.153
C(fips_code)[T.34007]                        3.7253      1.231      3.026      0.002       1.312       6.138
C(fips_code)[T.34009]                       19.9536      1.262     15.815      0.000      17.480      22.427
C(fips_code)[T.34011]                       18.4661      1.232     14.987      0.000      16.051      20.881
C(fips_code)[T.34013]                        3.3059      1.233      2.681      0.007       0.889       5.723
C(fips_code)[T.34015]                        0.5877      1.234      0.476      0.634      -1.831       3.006
C(fips_code)[T.34017]                        4.4051      1.231      3.578      0.000       1.992       6.818
C(fips_code)[T.34019]                      -14.0936      1.242    -11.344      0.000     -16.529     -11.658
C(fips_code)[T.34021]                       -2.9271      1.235     -2.370      0.018      -5.348      -0.506
C(fips_code)[T.34023]                       -6.5582      1.231     -5.328      0.000      -8.971      -4.145
C(fips_code)[T.34025]                       -7.2307      1.232     -5.867      0.000      -9.646      -4.815
C(fips_code)[T.34027]                      -13.2885      1.235    -10.757      0.000     -15.710     -10.867
C(fips_code)[T.34029]                       -1.5570      1.233     -1.263      0.207      -3.974       0.860
C(fips_code)[T.34031]                       -0.2866      1.237     -0.232      0.817      -2.711       2.137
C(fips_code)[T.34033]                        6.0252      1.235      4.879      0.000       3.604       8.446
C(fips_code)[T.34035]                      -12.5138      1.235    -10.133      0.000     -14.935     -10.093
C(fips_code)[T.34037]                        2.7475      1.236      2.224      0.026       0.326       5.169
C(fips_code)[T.34039]                        2.8874      1.233      2.341      0.019       0.470       5.305
C(fips_code)[T.34041]                        7.6923      1.237      6.220      0.000       5.268      10.116
C(fips_code)[T.35001]                       -1.7669      1.216     -1.453      0.146      -4.150       0.616
C(fips_code)[T.35005]                        0.7096      1.221      0.581      0.561      -1.683       3.102
C(fips_code)[T.35009]                        0.9818      1.224      0.802      0.422      -1.417       3.381
C(fips_code)[T.35013]                       -3.1561      1.220     -2.586      0.010      -5.548      -0.764
C(fips_code)[T.35015]                       -7.8974      1.225     -6.446      0.000     -10.299      -5.496
C(fips_code)[T.35025]                       -7.1653      1.222     -5.865      0.000      -9.560      -4.771
C(fips_code)[T.35031]                       -6.0378      1.242     -4.862      0.000      -8.472      -3.604
C(fips_code)[T.35035]                       -0.0903      1.224     -0.074      0.941      -2.490       2.309
C(fips_code)[T.35039]                       20.2140      1.512     13.368      0.000      17.250      23.178
C(fips_code)[T.35043]                        6.6902      1.221      5.480      0.000       4.297       9.083
C(fips_code)[T.35045]                       -2.2740      1.234     -1.842      0.065      -4.693       0.145
C(fips_code)[T.35049]                       -0.3710      1.213     -0.306      0.760      -2.748       2.006
C(fips_code)[T.35061]                        5.5292      1.220      4.533      0.000       3.138       7.920
C(fips_code)[T.36001]                       -9.2185      1.251     -7.372      0.000     -11.670      -6.767
C(fips_code)[T.36003]                       -8.1609      1.557     -5.241      0.000     -11.213      -5.109
C(fips_code)[T.36007]                        2.5494      1.248      2.042      0.041       0.102       4.996
C(fips_code)[T.36009]                       -0.5847      1.256     -0.466      0.642      -3.046       1.877
C(fips_code)[T.36011]                        3.4093      1.250      2.727      0.006       0.958       5.860
C(fips_code)[T.36013]                       11.5726      1.249      9.264      0.000       9.124      14.021
C(fips_code)[T.36015]                       -3.3232      1.249     -2.660      0.008      -5.772      -0.874
C(fips_code)[T.36017]                        3.3948      1.251      2.714      0.007       0.943       5.847
C(fips_code)[T.36019]                       -0.2401      1.249     -0.192      0.848      -2.689       2.208
C(fips_code)[T.36021]                       -8.0772      1.252     -6.450      0.000     -10.532      -5.623
C(fips_code)[T.36023]                        4.7708      1.249      3.819      0.000       2.322       7.220
C(fips_code)[T.36025]                       -6.2700      2.332     -2.688      0.007     -10.841      -1.699
C(fips_code)[T.36027]                       -8.2797      1.249     -6.631      0.000     -10.727      -5.832
C(fips_code)[T.36029]                       -5.5703      1.250     -4.456      0.000      -8.021      -3.120
C(fips_code)[T.36033]                        4.4654      1.249      3.574      0.000       2.017       6.914
C(fips_code)[T.36035]                        9.0129      1.249      7.218      0.000       6.565      11.460
C(fips_code)[T.36037]                        0.8109      1.250      0.649      0.517      -1.640       3.262
C(fips_code)[T.36039]                        5.6963      1.299      4.384      0.000       3.150       8.243
C(fips_code)[T.36043]                        5.5097      1.253      4.398      0.000       3.054       7.965
C(fips_code)[T.36045]                        3.9929      1.251      3.191      0.001       1.541       6.445
C(fips_code)[T.36051]                        4.1469      1.249      3.320      0.001       1.698       6.596
C(fips_code)[T.36053]                        9.3565      1.249      7.491      0.000       6.908      11.805
C(fips_code)[T.36055]                       -4.8282      1.248     -3.868      0.000      -7.275      -2.382
C(fips_code)[T.36057]                        8.2167      1.249      6.579      0.000       5.769      10.665
C(fips_code)[T.36059]                       -9.9772      1.258     -7.932      0.000     -12.443      -7.512
C(fips_code)[T.36061]                      -24.5264      1.275    -19.230      0.000     -27.026     -22.026
C(fips_code)[T.36063]                       -0.4211      1.258     -0.335      0.738      -2.888       2.045
C(fips_code)[T.36065]                       -0.1915      1.248     -0.153      0.878      -2.639       2.255
C(fips_code)[T.36067]                       -3.6683      1.248     -2.939      0.003      -6.115      -1.222
C(fips_code)[T.36069]                      -10.2724      1.252     -8.207      0.000     -12.726      -7.819
C(fips_code)[T.36071]                       -5.6489      1.255     -4.500      0.000      -8.109      -3.188
C(fips_code)[T.36073]                       15.0230      1.252     12.001      0.000      12.569      17.477
C(fips_code)[T.36075]                       12.8183      1.251     10.243      0.000      10.365      15.271
C(fips_code)[T.36077]                        4.0113      1.250      3.209      0.001       1.561       6.461
C(fips_code)[T.36079]                       -9.5757      1.249     -7.669      0.000     -12.023      -7.128
C(fips_code)[T.36083]                        2.4091      1.250      1.928      0.054      -0.040       4.859
C(fips_code)[T.36087]                      -10.5842      1.269     -8.339      0.000     -13.072      -8.096
C(fips_code)[T.36089]                        3.4771      1.250      2.783      0.005       1.028       5.927
C(fips_code)[T.36091]                       -9.8261      1.250     -7.861      0.000     -12.276      -7.376
C(fips_code)[T.36093]                       -0.2460      1.249     -0.197      0.844      -2.694       2.202
C(fips_code)[T.36099]                       12.3789      1.762      7.024      0.000       8.924      15.833
C(fips_code)[T.36101]                        1.5210      1.249      1.217      0.224      -0.928       3.970
C(fips_code)[T.36103]                       -5.0457      1.255     -4.019      0.000      -7.506      -2.585
C(fips_code)[T.36105]                       13.8459      1.250     11.078      0.000      11.396      16.296
C(fips_code)[T.36107]                        1.9736      1.556      1.268      0.205      -1.077       5.024
C(fips_code)[T.36109]                       -9.9412      1.254     -7.927      0.000     -12.399      -7.483
C(fips_code)[T.36111]                       -0.9110      1.248     -0.730      0.465      -3.358       1.536
C(fips_code)[T.36113]                        4.8199      1.249      3.859      0.000       2.372       7.268
C(fips_code)[T.36115]                        7.3873      1.556      4.746      0.000       4.337      10.438
C(fips_code)[T.36117]                        4.7338      1.252      3.780      0.000       2.279       7.188
C(fips_code)[T.36119]                       -7.6582      1.259     -6.084      0.000     -10.126      -5.191
C(fips_code)[T.36121]                        7.6166      1.651      4.614      0.000       4.381      10.852
C(fips_code)[T.37001]                       -7.0969      1.251     -5.675      0.000      -9.548      -4.646
C(fips_code)[T.37003]                        2.4372      1.253      1.945      0.052      -0.018       4.893
C(fips_code)[T.37013]                        5.1424      1.252      4.106      0.000       2.688       7.597
C(fips_code)[T.37017]                        4.7026      1.909      2.464      0.014       0.961       8.444
C(fips_code)[T.37019]                        1.0253      1.254      0.818      0.414      -1.433       3.483
C(fips_code)[T.37021]                       -8.8025      1.253     -7.027      0.000     -11.258      -6.347
C(fips_code)[T.37023]                        3.7889      1.251      3.028      0.002       1.337       6.241
C(fips_code)[T.37025]                      -10.3444      1.251     -8.270      0.000     -12.796      -7.893
C(fips_code)[T.37027]                        6.2332      1.252      4.977      0.000       3.778       8.688
C(fips_code)[T.37031]                        4.8403      1.252      3.867      0.000       2.387       7.293
C(fips_code)[T.37035]                       -6.2428      1.254     -4.979      0.000      -8.700      -3.785
C(fips_code)[T.37037]                        7.4605      1.254      5.949      0.000       5.002       9.918
C(fips_code)[T.37045]                        0.7137      1.252      0.570      0.569      -1.740       3.167
C(fips_code)[T.37047]                        8.8089      1.251      7.040      0.000       6.356      11.261
C(fips_code)[T.37049]                       -7.7332      1.251     -6.181      0.000     -10.186      -5.281
C(fips_code)[T.37051]                        2.3772      1.252      1.899      0.058      -0.076       4.830
C(fips_code)[T.37055]                       26.0410      1.488     17.505      0.000      23.125      28.957
C(fips_code)[T.37057]                       -0.1504      1.251     -0.120      0.904      -2.602       2.302
C(fips_code)[T.37059]                        0.7020      1.251      0.561      0.575      -1.750       3.154
C(fips_code)[T.37061]                       -2.4990      1.256     -1.990      0.047      -4.961      -0.037
C(fips_code)[T.37063]                       -6.5750      1.253     -5.248      0.000      -9.031      -4.119
C(fips_code)[T.37065]                       14.7093      1.251     11.757      0.000      12.257      17.162
C(fips_code)[T.37067]                       -2.7403      1.250     -2.192      0.028      -5.190      -0.290
C(fips_code)[T.37069]                        3.8696      1.251      3.094      0.002       1.418       6.321
C(fips_code)[T.37071]                       -2.6561      1.252     -2.121      0.034      -5.111      -0.201
C(fips_code)[T.37077]                        4.9561      1.252      3.958      0.000       2.502       7.410
C(fips_code)[T.37081]                       -3.2999      1.251     -2.639      0.008      -5.751      -0.848
C(fips_code)[T.37083]                       12.5771      1.250     10.062      0.000      10.127      15.027
C(fips_code)[T.37085]                        0.4599      1.252      0.367      0.713      -1.994       2.913
C(fips_code)[T.37087]                        1.8517      1.251      1.480      0.139      -0.600       4.303
C(fips_code)[T.37089]                       -1.0737      1.250     -0.859      0.390      -3.524       1.377
C(fips_code)[T.37093]                        4.4740      1.559      2.869      0.004       1.417       7.531
C(fips_code)[T.37097]                       -5.7950      1.251     -4.633      0.000      -8.247      -3.343
C(fips_code)[T.37099]                       -4.5683      1.250     -3.654      0.000      -7.019      -2.118
C(fips_code)[T.37101]                       -2.4677      1.251     -1.972      0.049      -4.920      -0.015
C(fips_code)[T.37105]                        1.7996      1.251      1.438      0.150      -0.653       4.252
C(fips_code)[T.37107]                        0.8374      1.254      0.668      0.504      -1.620       3.295
C(fips_code)[T.37109]                        1.5012      1.252      1.199      0.230      -0.952       3.955
C(fips_code)[T.37111]                        5.7115      1.251      4.565      0.000       3.259       8.164
C(fips_code)[T.37113]                       -1.5151      1.251     -1.212      0.226      -3.966       0.936
C(fips_code)[T.37119]                      -11.1537      1.250     -8.923      0.000     -13.604      -8.704
C(fips_code)[T.37125]                      -15.1782      1.252    -12.127      0.000     -17.631     -12.725
C(fips_code)[T.37127]                       -0.7793      1.251     -0.623      0.533      -3.231       1.672
C(fips_code)[T.37129]                       -9.2999      1.250     -7.438      0.000     -11.751      -6.849
C(fips_code)[T.37133]                       -5.2920      1.252     -4.227      0.000      -7.746      -2.838
C(fips_code)[T.37135]                       -9.7350      1.257     -7.744      0.000     -12.199      -7.271
C(fips_code)[T.37139]                        7.5455      1.250      6.034      0.000       5.095       9.996
C(fips_code)[T.37141]                        9.4168      1.250      7.532      0.000       6.966      11.867
C(fips_code)[T.37145]                       -1.1440      1.251     -0.914      0.361      -3.597       1.309
C(fips_code)[T.37147]                       -4.6035      1.251     -3.680      0.000      -7.056      -2.151
C(fips_code)[T.37151]                       -5.3343      1.252     -4.261      0.000      -7.788      -2.880
C(fips_code)[T.37153]                       11.3337      1.250      9.065      0.000       8.883      13.784
C(fips_code)[T.37155]                        6.9698      1.250      5.575      0.000       4.519       9.420
C(fips_code)[T.37157]                        3.2979      1.252      2.633      0.008       0.843       5.753
C(fips_code)[T.37159]                       -2.1989      1.251     -1.757      0.079      -4.652       0.254
C(fips_code)[T.37161]                        0.1612      1.253      0.129      0.898      -2.294       2.616
C(fips_code)[T.37163]                        1.5043      1.254      1.199      0.231      -0.955       3.963
C(fips_code)[T.37165]                       10.6856      1.255      8.517      0.000       8.226      13.145
C(fips_code)[T.37167]                        3.8733      1.252      3.094      0.002       1.420       6.327
C(fips_code)[T.37169]                      -10.6378      1.253     -8.489      0.000     -13.094      -8.182
C(fips_code)[T.37171]                        1.8132      1.251      1.450      0.147      -0.638       4.264
C(fips_code)[T.37179]                       -9.6540      1.253     -7.705      0.000     -12.110      -7.198
C(fips_code)[T.37181]                        5.9092      1.251      4.722      0.000       3.456       8.362
C(fips_code)[T.37183]                      -11.4998      1.251     -9.194      0.000     -13.951      -9.048
C(fips_code)[T.37189]                      -18.0880      1.410    -12.825      0.000     -20.852     -15.324
C(fips_code)[T.37191]                       -0.6977      1.252     -0.557      0.577      -3.151       1.756
C(fips_code)[T.37193]                       -0.8362      1.250     -0.669      0.504      -3.287       1.615
C(fips_code)[T.37195]                        2.4785      1.250      1.982      0.047       0.028       4.929
C(fips_code)[T.37197]                       -0.6727      1.653     -0.407      0.684      -3.914       2.568
C(fips_code)[T.39003]                       -4.2424      1.253     -3.385      0.001      -6.699      -1.786
C(fips_code)[T.39005]                        3.5846      1.254      2.858      0.004       1.126       6.043
C(fips_code)[T.39007]                       11.5460      1.251      9.228      0.000       9.094      13.998
C(fips_code)[T.39009]                      -11.8126      1.255     -9.410      0.000     -14.273      -9.352
C(fips_code)[T.39011]                       -3.0185      1.254     -2.408      0.016      -5.476      -0.561
C(fips_code)[T.39013]                       -7.2208      1.254     -5.757      0.000      -9.679      -4.762
C(fips_code)[T.39015]                        4.6597      1.411      3.302      0.001       1.893       7.426
C(fips_code)[T.39017]                      -11.1018      1.253     -8.863      0.000     -13.557      -8.646
C(fips_code)[T.39021]                        0.6568      1.252      0.525      0.600      -1.797       3.111
C(fips_code)[T.39023]                        6.5040      1.251      5.200      0.000       4.052       8.956
C(fips_code)[T.39025]                       -3.8363      1.253     -3.063      0.002      -6.291      -1.381
C(fips_code)[T.39027]                       -8.6127      1.253     -6.875      0.000     -11.068      -6.157
C(fips_code)[T.39029]                        3.5320      1.254      2.816      0.005       1.074       5.990
C(fips_code)[T.39031]                        6.3406      1.251      5.068      0.000       3.888       8.793
C(fips_code)[T.39033]                        4.7963      1.258      3.813      0.000       2.331       7.262
C(fips_code)[T.39035]                       -0.5112      1.258     -0.406      0.685      -2.977       1.955
C(fips_code)[T.39037]                        3.6720      1.253      2.931      0.003       1.216       6.128
C(fips_code)[T.39039]                        5.5329      1.253      4.416      0.000       3.077       7.989
C(fips_code)[T.39041]                      -18.0957      1.258    -14.388      0.000     -20.561     -15.631
C(fips_code)[T.39043]                        4.8716      1.261      3.864      0.000       2.400       7.343
C(fips_code)[T.39045]                       -9.0381      1.253     -7.211      0.000     -11.495      -6.581
C(fips_code)[T.39047]                        8.3231      2.091      3.980      0.000       4.224      12.422
C(fips_code)[T.39049]                       -6.0043      1.253     -4.790      0.000      -8.461      -3.547
C(fips_code)[T.39051]                       -2.4126      1.253     -1.926      0.054      -4.869       0.043
C(fips_code)[T.39055]                        1.5490      1.251      1.238      0.216      -0.903       4.001
C(fips_code)[T.39057]                      -12.9351      1.254    -10.313      0.000     -15.394     -10.477
C(fips_code)[T.39059]                        5.1236      1.251      4.096      0.000       2.672       7.576
C(fips_code)[T.39061]                       -8.0777      1.252     -6.453      0.000     -10.531      -5.624
C(fips_code)[T.39063]                       -9.0656      1.252     -7.239      0.000     -11.520      -6.611
C(fips_code)[T.39065]                        3.1470      1.768      1.780      0.075      -0.319       6.613
C(fips_code)[T.39069]                       -1.2579      1.768     -0.711      0.477      -4.724       2.208
C(fips_code)[T.39071]                       12.3628      1.253      9.868      0.000       9.907      14.818
C(fips_code)[T.39077]                        1.6364      1.254      1.305      0.192      -0.822       4.095
C(fips_code)[T.39081]                        1.6569      1.253      1.323      0.186      -0.799       4.113
C(fips_code)[T.39083]                        3.6113      1.255      2.878      0.004       1.152       6.071
C(fips_code)[T.39085]                       -6.0093      1.253     -4.796      0.000      -8.465      -3.553
C(fips_code)[T.39087]                       12.3531      1.251      9.874      0.000       9.901      14.805
C(fips_code)[T.39089]                       -3.9968      1.254     -3.188      0.001      -6.454      -1.539
C(fips_code)[T.39091]                       12.9566      1.257     10.309      0.000      10.493      15.420
C(fips_code)[T.39093]                        7.5765      1.260      6.013      0.000       5.107      10.046
C(fips_code)[T.39095]                       -0.8375      1.258     -0.666      0.505      -3.303       1.627
C(fips_code)[T.39097]                       -6.4082      1.255     -5.108      0.000      -8.867      -3.949
C(fips_code)[T.39099]                        3.1575      1.255      2.516      0.012       0.698       5.617
C(fips_code)[T.39101]                        5.6708      1.268      4.473      0.000       3.186       8.156
C(fips_code)[T.39103]                       -6.2425      1.253     -4.983      0.000      -8.698      -3.787
C(fips_code)[T.39107]                       -9.3220      1.257     -7.417      0.000     -11.785      -6.859
C(fips_code)[T.39109]                       -4.1510      1.253     -3.312      0.001      -6.607      -1.695
C(fips_code)[T.39113]                       -2.8984      1.251     -2.317      0.021      -5.351      -0.446
C(fips_code)[T.39117]                        0.6252      2.091      0.299      0.765      -3.474       4.725
C(fips_code)[T.39119]                        1.1011      1.252      0.880      0.379      -1.352       3.554
C(fips_code)[T.39123]                       29.6824      1.253     23.697      0.000      27.227      32.138
C(fips_code)[T.39129]                        7.7500      1.266      6.122      0.000       5.269      10.231
C(fips_code)[T.39133]                       -1.7778      1.252     -1.420      0.156      -4.232       0.676
C(fips_code)[T.39135]                        8.5714      1.252      6.846      0.000       6.117      11.025
C(fips_code)[T.39137]                      -13.3332      1.769     -7.539      0.000     -16.800      -9.867
C(fips_code)[T.39139]                       -6.1935      1.254     -4.939      0.000      -8.651      -3.736
C(fips_code)[T.39141]                        2.1387      1.251      1.709      0.087      -0.314       4.591
C(fips_code)[T.39143]                        8.3660      1.252      6.684      0.000       5.913      10.819
C(fips_code)[T.39145]                        4.8268      1.252      3.857      0.000       2.374       7.280
C(fips_code)[T.39147]                        8.5312      1.252      6.814      0.000       6.077      10.985
C(fips_code)[T.39149]                       10.0054      1.252      7.992      0.000       7.552      12.459
C(fips_code)[T.39151]                       -1.6133      1.251     -1.290      0.197      -4.065       0.839
C(fips_code)[T.39153]                       -1.2189      1.252     -0.974      0.330      -3.672       1.234
C(fips_code)[T.39155]                        1.9779      1.254      1.577      0.115      -0.481       4.437
C(fips_code)[T.39157]                       -2.1816      1.252     -1.743      0.081      -4.635       0.272
C(fips_code)[T.39159]                        1.4055      1.253      1.122      0.262      -1.051       3.862
C(fips_code)[T.39161]                       -1.7244      1.252     -1.377      0.169      -4.179       0.731
C(fips_code)[T.39165]                      -11.0258      1.254     -8.795      0.000     -13.483      -8.569
C(fips_code)[T.39167]                       -4.4785      1.252     -3.577      0.000      -6.933      -2.024
C(fips_code)[T.39169]                       -9.2460      1.256     -7.359      0.000     -11.709      -6.783
C(fips_code)[T.39171]                       -2.4561      1.257     -1.953      0.051      -4.921       0.009
C(fips_code)[T.39173]                       -4.1982      1.251     -3.355      0.001      -6.651      -1.746
C(fips_code)[T.41003]                      -14.1742      1.233    -11.496      0.000     -16.591     -11.757
C(fips_code)[T.41005]                       -6.6587      1.230     -5.414      0.000      -9.070      -4.248
C(fips_code)[T.41007]                        7.5806      1.242      6.106      0.000       5.147      10.014
C(fips_code)[T.41009]                        4.1390      1.231      3.362      0.001       1.726       6.552
C(fips_code)[T.41011]                       -1.4390      1.241     -1.160      0.246      -3.872       0.994
C(fips_code)[T.41017]                       -6.9681      1.234     -5.645      0.000      -9.388      -4.548
C(fips_code)[T.41019]                        0.8955      1.238      0.723      0.470      -1.531       3.322
C(fips_code)[T.41029]                       -3.8084      1.233     -3.090      0.002      -6.224      -1.392
C(fips_code)[T.41033]                        0.1466      1.237      0.119      0.906      -2.278       2.571
C(fips_code)[T.41035]                        7.5030      1.232      6.091      0.000       5.088       9.918
C(fips_code)[T.41039]                       -0.0957      1.228     -0.078      0.938      -2.502       2.311
C(fips_code)[T.41041]                        7.8796      1.246      6.326      0.000       5.438      10.321
C(fips_code)[T.41043]                        4.2137      1.231      3.422      0.001       1.800       6.627
C(fips_code)[T.41045]                        9.5987      1.538      6.240      0.000       6.584      12.614
C(fips_code)[T.41047]                       -4.8274      1.232     -3.920      0.000      -7.241      -2.413
C(fips_code)[T.41051]                       -7.4325      1.226     -6.060      0.000      -9.837      -5.028
C(fips_code)[T.41053]                        5.0171      1.231      4.077      0.000       2.605       7.429
C(fips_code)[T.41059]                        7.1378      1.234      5.786      0.000       4.720       9.556
C(fips_code)[T.41067]                      -10.0508      1.231     -8.166      0.000     -12.463      -7.638
C(fips_code)[T.41071]                        3.4863      1.231      2.833      0.005       1.074       5.898
C(fips_code)[T.42001]                       -5.4061      1.249     -4.328      0.000      -7.855      -2.958
C(fips_code)[T.42003]                       -8.6806      1.248     -6.957      0.000     -11.126      -6.235
C(fips_code)[T.42005]                        1.7557      1.251      1.404      0.160      -0.696       4.207
C(fips_code)[T.42007]                       -5.8370      1.252     -4.663      0.000      -8.291      -3.383
C(fips_code)[T.42009]                        1.6433      1.252      1.312      0.189      -0.811       4.098
C(fips_code)[T.42011]                        0.7624      1.253      0.609      0.543      -1.693       3.218
C(fips_code)[T.42013]                       -2.1201      1.250     -1.697      0.090      -4.570       0.329
C(fips_code)[T.42015]                       11.2874      1.254      8.998      0.000       8.829      13.746
C(fips_code)[T.42017]                       -3.7867      1.253     -3.022      0.003      -6.243      -1.330
C(fips_code)[T.42019]                       -8.9374      1.250     -7.152      0.000     -11.387      -6.488
C(fips_code)[T.42021]                        0.7485      1.249      0.599      0.549      -1.700       3.197
C(fips_code)[T.42025]                        1.9485      1.251      1.557      0.119      -0.504       4.401
C(fips_code)[T.42027]                      -18.0269      1.259    -14.320      0.000     -20.494     -15.559
C(fips_code)[T.42029]                       -8.5179      1.256     -6.784      0.000     -10.979      -6.057
C(fips_code)[T.42033]                        6.5181      1.253      5.201      0.000       4.062       8.974
C(fips_code)[T.42035]                        7.9943      1.250      6.393      0.000       5.543      10.445
C(fips_code)[T.42037]                       -3.0293      1.250     -2.424      0.015      -5.479      -0.580
C(fips_code)[T.42039]                        6.1177      1.254      4.879      0.000       3.660       8.576
C(fips_code)[T.42041]                      -10.9852      1.251     -8.779      0.000     -13.438      -8.533
C(fips_code)[T.42043]                       -3.1659      1.249     -2.536      0.011      -5.613      -0.719
C(fips_code)[T.42045]                        1.8477      1.254      1.473      0.141      -0.611       4.306
C(fips_code)[T.42049]                        0.0990      1.249      0.079      0.937      -2.349       2.547
C(fips_code)[T.42051]                        7.7292      1.253      6.167      0.000       5.273      10.186
C(fips_code)[T.42055]                       -0.1078      1.250     -0.086      0.931      -2.557       2.341
C(fips_code)[T.42061]                        1.6750      1.252      1.338      0.181      -0.780       4.130
C(fips_code)[T.42063]                       -1.4051      1.249     -1.125      0.261      -3.854       1.044
C(fips_code)[T.42065]                        3.6622      1.254      2.921      0.003       1.205       6.120
C(fips_code)[T.42069]                       -0.0364      1.254     -0.029      0.977      -2.494       2.421
C(fips_code)[T.42071]                       -9.2830      1.254     -7.404      0.000     -11.740      -6.826
C(fips_code)[T.42073]                        8.6390      1.254      6.891      0.000       6.182      11.096
C(fips_code)[T.42075]                       -3.3889      1.250     -2.711      0.007      -5.839      -0.939
C(fips_code)[T.42077]                       -6.1893      1.253     -4.941      0.000      -8.644      -3.734
C(fips_code)[T.42079]                       -1.7561      1.253     -1.402      0.161      -4.212       0.699
C(fips_code)[T.42081]                       -4.8349      1.251     -3.865      0.000      -7.287      -2.383
C(fips_code)[T.42083]                        3.8664      1.255      3.081      0.002       1.407       6.326
C(fips_code)[T.42085]                        0.6783      1.251      0.542      0.588      -1.773       3.130
C(fips_code)[T.42087]                        3.1777      1.250      2.542      0.011       0.727       5.628
C(fips_code)[T.42089]                        2.5580      1.255      2.039      0.041       0.099       5.017
C(fips_code)[T.42091]                       -9.4502      1.254     -7.535      0.000     -11.908      -6.992
C(fips_code)[T.42095]                       -3.8183      1.253     -3.049      0.002      -6.273      -1.363
C(fips_code)[T.42097]                        0.8493      1.252      0.678      0.498      -1.605       3.303
C(fips_code)[T.42099]                        6.6917      1.652      4.051      0.000       3.454       9.930
C(fips_code)[T.42101]                        2.3779      1.257      1.892      0.059      -0.086       4.841
C(fips_code)[T.42103]                        8.7899      1.254      7.009      0.000       6.332      11.248
C(fips_code)[T.42107]                        3.6379      1.250      2.910      0.004       1.187       6.088
C(fips_code)[T.42109]                        2.6770      1.565      1.711      0.087      -0.390       5.744
C(fips_code)[T.42111]                        1.0300      1.250      0.824      0.410      -1.420       3.480
C(fips_code)[T.42115]                        6.1052      1.651      3.698      0.000       2.869       9.342
C(fips_code)[T.42117]                        0.6296      1.254      0.502      0.616      -1.829       3.088
C(fips_code)[T.42121]                        9.9832      1.254      7.958      0.000       7.524      12.442
C(fips_code)[T.42123]                       10.4706      1.254      8.352      0.000       8.013      12.928
C(fips_code)[T.42125]                       -8.0741      1.249     -6.462      0.000     -10.523      -5.625
C(fips_code)[T.42127]                        7.9349      1.252      6.340      0.000       5.482      10.388
C(fips_code)[T.42129]                       -2.6336      1.250     -2.108      0.035      -5.083      -0.184
C(fips_code)[T.42133]                       -1.5445      1.249     -1.237      0.216      -3.993       0.903
C(fips_code)[T.44001]                        1.6826      1.442      1.166      0.243      -1.145       4.510
C(fips_code)[T.44003]                       -1.6849      1.206     -1.397      0.162      -4.048       0.679
C(fips_code)[T.44005]                       -1.1293      0.966     -1.169      0.243      -3.023       0.765
C(fips_code)[T.44007]                        1.1458      1.199      0.955      0.339      -1.205       3.496
C(fips_code)[T.44009]                        4.3830      1.197      3.662      0.000       2.037       6.729
C(fips_code)[T.45003]                       -2.2089      1.239     -1.782      0.075      -4.638       0.220
C(fips_code)[T.45007]                       -4.5410      1.238     -3.669      0.000      -6.967      -2.115
C(fips_code)[T.45013]                       -4.7565      1.238     -3.841      0.000      -7.184      -2.329
C(fips_code)[T.45015]                       -5.6785      1.240     -4.581      0.000      -8.108      -3.249
C(fips_code)[T.45019]                       -8.6131      1.236     -6.968      0.000     -11.036      -6.190
C(fips_code)[T.45021]                        0.1332      1.243      0.107      0.915      -2.302       2.569
C(fips_code)[T.45023]                        1.1063      1.241      0.891      0.373      -1.327       3.540
C(fips_code)[T.45025]                        4.8517      1.237      3.921      0.000       2.426       7.277
C(fips_code)[T.45029]                        3.1075      1.239      2.508      0.012       0.679       5.537
C(fips_code)[T.45031]                        5.9137      1.238      4.778      0.000       3.488       8.340
C(fips_code)[T.45035]                       -0.8911      1.237     -0.720      0.471      -3.316       1.534
C(fips_code)[T.45041]                        1.6122      1.238      1.303      0.193      -0.814       4.038
C(fips_code)[T.45043]                        5.7336      1.238      4.632      0.000       3.307       8.160
C(fips_code)[T.45045]                       -8.5034      1.238     -6.869      0.000     -10.930      -6.077
C(fips_code)[T.45047]                       -2.3144      1.239     -1.867      0.062      -4.744       0.115
C(fips_code)[T.45051]                        5.3407      1.249      4.275      0.000       2.892       7.790
C(fips_code)[T.45055]                       -3.0165      1.238     -2.436      0.015      -5.444      -0.589
C(fips_code)[T.45057]                        0.6807      1.239      0.549      0.583      -1.748       3.109
C(fips_code)[T.45059]                       10.8343      1.239      8.747      0.000       8.406      13.262
C(fips_code)[T.45063]                       -8.9019      1.241     -7.171      0.000     -11.335      -6.468
C(fips_code)[T.45071]                        1.8937      1.239      1.528      0.126      -0.535       4.322
C(fips_code)[T.45073]                        1.8707      1.241      1.508      0.132      -0.562       4.303
C(fips_code)[T.45075]                        9.8434      1.237      7.958      0.000       7.419      12.268
C(fips_code)[T.45077]                       -0.9943      1.238     -0.803      0.422      -3.421       1.433
C(fips_code)[T.45079]                       -1.9520      1.237     -1.578      0.115      -4.377       0.473
C(fips_code)[T.45083]                       -0.6181      1.240     -0.499      0.618      -3.048       1.812
C(fips_code)[T.45085]                        0.3918      1.238      0.317      0.752      -2.034       2.818
C(fips_code)[T.45091]                       -2.0173      1.237     -1.630      0.103      -4.443       0.408
C(fips_code)[T.47001]                       -5.6326      1.247     -4.517      0.000      -8.077      -3.188
C(fips_code)[T.47003]                        3.5547      1.250      2.845      0.004       1.105       6.004
C(fips_code)[T.47009]                       -7.6637      1.248     -6.140      0.000     -10.110      -5.217
C(fips_code)[T.47011]                       -5.4240      1.248     -4.346      0.000      -7.870      -2.978
C(fips_code)[T.47013]                        7.4933      1.248      6.002      0.000       5.046       9.940
C(fips_code)[T.47019]                       12.3740      1.248      9.919      0.000       9.929      14.819
C(fips_code)[T.47021]                      -12.8311      1.251    -10.257      0.000     -15.283     -10.379
C(fips_code)[T.47029]                       14.8014      1.256     11.788      0.000      12.340      17.263
C(fips_code)[T.47031]                        1.6466      1.249      1.318      0.187      -0.802       4.095
C(fips_code)[T.47035]                        0.6591      1.248      0.528      0.597      -1.787       3.106
C(fips_code)[T.47037]                      -11.0686      1.247     -8.879      0.000     -13.512      -8.625
C(fips_code)[T.47043]                       -5.8297      1.249     -4.666      0.000      -8.278      -3.381
C(fips_code)[T.47045]                        9.9684      1.246      8.000      0.000       7.526      12.411
C(fips_code)[T.47047]                       -2.8214      1.903     -1.482      0.138      -6.552       0.909
C(fips_code)[T.47051]                       13.3671      1.249     10.698      0.000      10.918      15.816
C(fips_code)[T.47053]                        4.4245      1.248      3.545      0.000       1.978       6.871
C(fips_code)[T.47059]                        6.2908      1.246      5.048      0.000       3.848       8.734
C(fips_code)[T.47063]                       -0.2348      1.246     -0.188      0.851      -2.678       2.208
C(fips_code)[T.47065]                       -9.6166      1.246     -7.715      0.000     -12.060      -7.173
C(fips_code)[T.47073]                        3.7363      1.249      2.991      0.003       1.288       6.185
C(fips_code)[T.47079]                        2.1917      1.248      1.757      0.079      -0.254       4.637
C(fips_code)[T.47089]                        9.0657      1.246      7.274      0.000       6.623      11.509
C(fips_code)[T.47093]                      -10.3906      1.248     -8.326      0.000     -12.837      -7.944
C(fips_code)[T.47099]                        4.4404      1.248      3.559      0.000       1.995       6.886
C(fips_code)[T.47103]                       -1.3469      1.763     -0.764      0.445      -4.802       2.108
C(fips_code)[T.47105]                       -0.5334      1.247     -0.428      0.669      -2.977       1.911
C(fips_code)[T.47107]                        2.7777      1.247      2.228      0.026       0.334       5.222
C(fips_code)[T.47113]                       -2.1078      1.246     -1.691      0.091      -4.551       0.335
C(fips_code)[T.47117]                       13.2097      1.351      9.777      0.000      10.561      15.858
C(fips_code)[T.47119]                        2.1156      1.249      1.694      0.090      -0.333       4.564
C(fips_code)[T.47123]                        6.8726      1.251      5.496      0.000       4.421       9.324
C(fips_code)[T.47125]                       -2.5693      1.247     -2.061      0.039      -5.013      -0.126
C(fips_code)[T.47131]                       -5.0568      1.556     -3.251      0.001      -8.106      -2.008
C(fips_code)[T.47141]                       -3.2965      1.247     -2.643      0.008      -5.742      -0.852
C(fips_code)[T.47143]                        8.1509      1.477      5.519      0.000       5.256      11.046
C(fips_code)[T.47145]                       12.1929      1.248      9.767      0.000       9.746      14.640
C(fips_code)[T.47147]                        3.0874      1.248      2.474      0.013       0.641       5.534
C(fips_code)[T.47149]                       -7.1058      1.247     -5.701      0.000      -9.549      -4.662
C(fips_code)[T.47155]                        1.7341      1.264      1.372      0.170      -0.744       4.212
C(fips_code)[T.47157]                       -1.3035      1.246     -1.046      0.296      -3.747       1.139
C(fips_code)[T.47163]                       -2.7995      1.248     -2.244      0.025      -5.245      -0.354
C(fips_code)[T.47165]                       -9.6346      1.248     -7.719      0.000     -12.081      -7.188
C(fips_code)[T.47167]                        1.8299      1.251      1.463      0.144      -0.622       4.282
C(fips_code)[T.47177]                        9.6599      1.258      7.676      0.000       7.193      12.127
C(fips_code)[T.47179]                      -10.5581      1.248     -8.461      0.000     -13.004      -8.112
C(fips_code)[T.47187]                      -26.2601      1.252    -20.971      0.000     -28.715     -23.806
C(fips_code)[T.47189]                      -11.0137      1.248     -8.823      0.000     -13.461      -8.567
C(fips_code)[T.48001]                       -2.2331      1.257     -1.776      0.076      -4.697       0.231
C(fips_code)[T.48005]                       -1.9476      1.253     -1.554      0.120      -4.404       0.509
C(fips_code)[T.48007]                        4.6831      1.657      2.826      0.005       1.435       7.931
C(fips_code)[T.48013]                        5.1884      1.253      4.141      0.000       2.733       7.644
C(fips_code)[T.48021]                        4.9670      1.253      3.963      0.000       2.510       7.424
C(fips_code)[T.48027]                        3.4892      1.253      2.785      0.005       1.033       5.945
C(fips_code)[T.48029]                       -2.0797      1.254     -1.659      0.097      -4.537       0.378
C(fips_code)[T.48037]                       -0.6981      1.254     -0.557      0.578      -3.155       1.759
C(fips_code)[T.48039]                       -0.7641      1.254     -0.609      0.542      -3.221       1.693
C(fips_code)[T.48041]                      -10.8719      1.256     -8.657      0.000     -13.334      -8.410
C(fips_code)[T.48049]                        2.1461      1.255      1.710      0.087      -0.314       4.606
C(fips_code)[T.48053]                        1.0604      1.257      0.844      0.399      -1.403       3.524
C(fips_code)[T.48055]                        1.4553      1.253      1.161      0.246      -1.002       3.912
C(fips_code)[T.48061]                       -3.5042      1.258     -2.786      0.005      -5.970      -1.039
C(fips_code)[T.48067]                        1.5460      2.342      0.660      0.509      -3.045       6.137
C(fips_code)[T.48071]                       -0.0032      1.255     -0.003      0.998      -2.463       2.456
C(fips_code)[T.48073]                       10.1288      1.253      8.086      0.000       7.673      12.584
C(fips_code)[T.48085]                      -14.2161      1.253    -11.344      0.000     -16.673     -11.760
C(fips_code)[T.48091]                      -10.2445      1.255     -8.166      0.000     -12.703      -7.785
C(fips_code)[T.48097]                        3.3381      1.254      2.663      0.008       0.881       5.795
C(fips_code)[T.48099]                        2.4136      1.253      1.926      0.054      -0.043       4.870
C(fips_code)[T.48113]                       -0.3947      1.255     -0.315      0.753      -2.854       2.064
C(fips_code)[T.48121]                       -5.5254      1.253     -4.411      0.000      -7.981      -3.070
C(fips_code)[T.48135]                       -6.2526      1.254     -4.984      0.000      -8.712      -3.794
C(fips_code)[T.48139]                       -2.4689      1.254     -1.969      0.049      -4.927      -0.011
C(fips_code)[T.48141]                       -3.0945      1.254     -2.467      0.014      -5.553      -0.636
C(fips_code)[T.48143]                      -11.8760      1.415     -8.392      0.000     -14.650      -9.102
C(fips_code)[T.48147]                        0.9267      1.306      0.710      0.478      -1.632       3.486
C(fips_code)[T.48157]                       -5.4631      1.253     -4.361      0.000      -7.919      -3.007
C(fips_code)[T.48167]                        4.6208      1.255      3.683      0.000       2.161       7.080
C(fips_code)[T.48181]                        4.5243      1.254      3.609      0.000       2.067       6.982
C(fips_code)[T.48183]                       -3.2700      1.254     -2.608      0.009      -5.727      -0.813
C(fips_code)[T.48187]                       -1.9958      1.253     -1.592      0.111      -4.453       0.461
C(fips_code)[T.48189]                        8.5996      1.413      6.086      0.000       5.830      11.370
C(fips_code)[T.48199]                        0.0928      1.256      0.074      0.941      -2.370       2.556
C(fips_code)[T.48201]                       -0.8499      1.254     -0.678      0.498      -3.308       1.608
C(fips_code)[T.48203]                        0.6132      1.255      0.489      0.625      -1.846       3.072
C(fips_code)[T.48209]                       -0.9050      1.253     -0.722      0.470      -3.361       1.551
C(fips_code)[T.48213]                        4.6068      1.254      3.673      0.000       2.148       7.065
C(fips_code)[T.48215]                       -1.0545      1.262     -0.835      0.403      -3.529       1.419
C(fips_code)[T.48217]                        5.3846      1.253      4.296      0.000       2.928       7.841
C(fips_code)[T.48221]                        1.8421      1.253      1.470      0.142      -0.615       4.299
C(fips_code)[T.48223]                        1.4836      1.257      1.181      0.238      -0.979       3.946
C(fips_code)[T.48227]                       -2.8926      1.253     -2.309      0.021      -5.348      -0.437
C(fips_code)[T.48231]                        2.9098      1.253      2.322      0.020       0.454       5.366
C(fips_code)[T.48241]                       11.5507      1.662      6.948      0.000       8.292      14.809
C(fips_code)[T.48245]                       -1.3297      1.264     -1.052      0.293      -3.808       1.148
C(fips_code)[T.48249]                       12.3785      1.307      9.471      0.000       9.817      14.941
C(fips_code)[T.48251]                       -0.5973      1.254     -0.476      0.634      -3.056       1.862
C(fips_code)[T.48257]                        0.3477      1.253      0.277      0.781      -2.109       2.804
C(fips_code)[T.48259]                       -9.8285      1.914     -5.136      0.000     -13.579      -6.078
C(fips_code)[T.48265]                       -1.2178      1.254     -0.971      0.332      -3.676       1.240
C(fips_code)[T.48273]                       -1.2312      1.482     -0.831      0.406      -4.136       1.674
C(fips_code)[T.48277]                        2.1353      1.256      1.701      0.089      -0.326       4.596
C(fips_code)[T.48291]                        4.3894      1.256      3.494      0.000       1.927       6.852
C(fips_code)[T.48303]                       -1.7216      1.254     -1.372      0.170      -4.181       0.737
C(fips_code)[T.48309]                       -4.8583      1.254     -3.875      0.000      -7.316      -2.401
C(fips_code)[T.48321]                        4.1072      1.255      3.272      0.001       1.647       6.567
C(fips_code)[T.48323]                       -2.4124      1.276     -1.891      0.059      -4.913       0.088
C(fips_code)[T.48325]                        9.9903      1.253      7.970      0.000       7.533      12.447
C(fips_code)[T.48329]                      -13.0393      1.254    -10.401      0.000     -15.497     -10.582
C(fips_code)[T.48339]                       -9.1212      1.254     -7.276      0.000     -11.578      -6.664
C(fips_code)[T.48347]                       -7.6151      1.254     -6.074      0.000     -10.073      -5.157
C(fips_code)[T.48349]                       -5.3199      1.254     -4.242      0.000      -7.778      -2.861
C(fips_code)[T.48355]                        6.8835      1.254      5.489      0.000       4.425       9.342
C(fips_code)[T.48361]                        5.0019      1.261      3.967      0.000       2.531       7.473
C(fips_code)[T.48363]                        7.7001      1.771      4.348      0.000       4.229      11.172
C(fips_code)[T.48367]                        0.5603      1.254      0.447      0.655      -1.898       3.018
C(fips_code)[T.48373]                       15.5632      1.253     12.423      0.000      13.108      18.019
C(fips_code)[T.48375]                       -3.1295      1.259     -2.486      0.013      -5.597      -0.662
C(fips_code)[T.48381]                       -1.6496      1.258     -1.311      0.190      -4.115       0.816
C(fips_code)[T.48397]                        0.2078      1.253      0.166      0.868      -2.248       2.664
C(fips_code)[T.48401]                        3.3122      1.255      2.639      0.008       0.852       5.772
C(fips_code)[T.48409]                        2.7547      1.258      2.191      0.029       0.290       5.220
C(fips_code)[T.48423]                       -7.8917      1.255     -6.289      0.000     -10.351      -5.432
C(fips_code)[T.48427]                        4.7655      1.302      3.659      0.000       2.212       7.319
C(fips_code)[T.48439]                       -4.1659      1.253     -3.325      0.001      -6.622      -1.710
C(fips_code)[T.48441]                       -0.4456      1.255     -0.355      0.723      -2.906       2.014
C(fips_code)[T.48449]                        5.2190      1.256      4.154      0.000       2.757       7.681
C(fips_code)[T.48451]                       -5.9423      1.253     -4.741      0.000      -8.399      -3.486
C(fips_code)[T.48453]                       -4.8519      1.259     -3.855      0.000      -7.319      -2.385
C(fips_code)[T.48459]                        4.5852      1.255      3.654      0.000       2.126       7.045
C(fips_code)[T.48465]                        2.3448      1.254      1.871      0.061      -0.112       4.802
C(fips_code)[T.48467]                       -0.8624      1.256     -0.687      0.492      -3.323       1.599
C(fips_code)[T.48469]                       -0.6547      1.253     -0.523      0.601      -3.110       1.801
C(fips_code)[T.48471]                       -4.1829      1.262     -3.314      0.001      -6.657      -1.709
C(fips_code)[T.48473]                        0.6927      1.253      0.553      0.580      -1.762       3.148
C(fips_code)[T.48479]                       -5.0238      1.254     -4.005      0.000      -7.483      -2.565
C(fips_code)[T.48481]                        7.8295      1.254      6.246      0.000       5.372      10.287
C(fips_code)[T.48485]                        4.3414      1.253      3.464      0.001       1.885       6.798
C(fips_code)[T.48491]                       -8.3391      1.253     -6.655      0.000     -10.795      -5.883
C(fips_code)[T.48493]                       -5.1443      1.255     -4.100      0.000      -7.604      -2.685
C(fips_code)[T.48497]                        4.3395      1.253      3.463      0.001       1.883       6.796
C(fips_code)[T.48499]                        5.3761      1.255      4.285      0.000       2.917       7.835
C(fips_code)[T.49035]                        2.1895      2.451      0.893      0.372      -2.615       6.994
C(fips_code)[T.50007]                       -3.0025      1.193     -2.517      0.012      -5.340      -0.665
C(fips_code)[T.50011]                        7.1834      1.209      5.942      0.000       4.814       9.553
C(fips_code)[T.50021]                        3.5270      1.213      2.907      0.004       1.149       5.905
C(fips_code)[T.50023]                       -1.9169      1.194     -1.606      0.108      -4.257       0.423
C(fips_code)[T.50027]                       -4.1276      1.206     -3.423      0.001      -6.491      -1.764
C(fips_code)[T.51001]                        0.0170      1.405      0.012      0.990      -2.737       2.771
C(fips_code)[T.51013]                      -19.4822      1.244    -15.660      0.000     -21.921     -17.044
C(fips_code)[T.51019]                       -1.3086      1.245     -1.051      0.293      -3.748       1.131
C(fips_code)[T.51033]                      -11.1761      1.641     -6.811      0.000     -14.392      -7.960
C(fips_code)[T.51041]                       -3.1684      1.241     -2.553      0.011      -5.601      -0.735
C(fips_code)[T.51047]                       -2.5673      1.245     -2.062      0.039      -5.007      -0.127
C(fips_code)[T.51061]                        1.9024      1.243      1.530      0.126      -0.534       4.339
C(fips_code)[T.51067]                        5.0817      1.246      4.080      0.000       2.640       7.523
C(fips_code)[T.51073]                        3.4475      1.242      2.776      0.006       1.013       5.882
C(fips_code)[T.51083]                        7.0061      1.467      4.775      0.000       4.130       9.882
C(fips_code)[T.51085]                       -2.8290      1.244     -2.275      0.023      -5.267      -0.391
C(fips_code)[T.51087]                       -6.4407      1.241     -5.191      0.000      -8.873      -4.009
C(fips_code)[T.51093]                        1.5196      1.243      1.223      0.221      -0.916       3.955
C(fips_code)[T.51107]                      -12.9944      1.241    -10.472      0.000     -15.427     -10.562
C(fips_code)[T.51109]                       11.6821      1.243      9.400      0.000       9.246      14.118
C(fips_code)[T.51137]                        8.5272      1.753      4.865      0.000       5.091      11.963
C(fips_code)[T.51155]                       12.2373      1.249      9.796      0.000       9.789      14.686
C(fips_code)[T.51171]                        1.6120      1.243      1.297      0.195      -0.825       4.049
C(fips_code)[T.51179]                       -2.1716      1.243     -1.747      0.081      -4.607       0.264
C(fips_code)[T.51185]                        5.9772      1.405      4.254      0.000       3.223       8.731
C(fips_code)[T.51187]                        7.4550      1.243      5.998      0.000       5.019       9.891
C(fips_code)[T.51510]                       -7.7888      1.240     -6.280      0.000     -10.220      -5.358
C(fips_code)[T.51550]                       -1.6579      1.242     -1.335      0.182      -4.092       0.776
C(fips_code)[T.51650]                        1.8890      1.242      1.521      0.128      -0.546       4.324
C(fips_code)[T.51700]                       -1.4195      1.243     -1.142      0.253      -3.855       1.016
C(fips_code)[T.51710]                       -1.4230      1.241     -1.146      0.252      -3.856       1.010
C(fips_code)[T.51740]                       18.4563      1.241     14.872      0.000      16.024      20.889
C(fips_code)[T.51760]                       -1.9184      1.241     -1.546      0.122      -4.351       0.515
C(fips_code)[T.51770]                       -6.1935      1.242     -4.988      0.000      -8.627      -3.760
C(fips_code)[T.51800]                        0.1976      1.241      0.159      0.874      -2.235       2.630
C(fips_code)[T.51810]                       -4.3910      1.242     -3.536      0.000      -6.825      -1.957
C(fips_code)[T.53003]                        0.9875      2.069      0.477      0.633      -3.068       5.042
C(fips_code)[T.53005]                       -9.2882      0.898    -10.338      0.000     -11.049      -7.527
C(fips_code)[T.53007]                        0.5135      1.239      0.414      0.679      -1.915       2.942
C(fips_code)[T.53009]                        6.7444      1.239      5.442      0.000       4.315       9.174
C(fips_code)[T.53011]                       -4.2789      1.238     -3.457      0.001      -6.705      -1.853
C(fips_code)[T.53015]                        5.5527      1.239      4.480      0.000       3.123       7.982
C(fips_code)[T.53017]                      -11.1056      1.239     -8.964      0.000     -13.534      -8.677
C(fips_code)[T.53021]                        4.4832      1.240      3.616      0.000       2.053       6.913
C(fips_code)[T.53025]                        8.8885      1.240      7.169      0.000       6.458      11.319
C(fips_code)[T.53027]                       12.4998      1.243     10.060      0.000      10.064      14.935
C(fips_code)[T.53029]                        6.3175      1.238      5.103      0.000       3.891       8.744
C(fips_code)[T.53033]                      -11.7622      1.237     -9.510      0.000     -14.186      -9.338
C(fips_code)[T.53035]                       -2.9221      1.239     -2.359      0.018      -5.350      -0.494
C(fips_code)[T.53037]                       -5.9647      1.240     -4.812      0.000      -8.394      -3.535
C(fips_code)[T.53041]                       -2.8095      1.243     -2.261      0.024      -5.245      -0.374
C(fips_code)[T.53045]                       16.5087      1.238     13.337      0.000      14.082      18.935
C(fips_code)[T.53047]                       11.9816      1.339      8.946      0.000       9.356      14.607
C(fips_code)[T.53053]                       -3.3509      1.240     -2.703      0.007      -5.781      -0.921
C(fips_code)[T.53057]                        0.5029      1.239      0.406      0.685      -1.925       2.931
C(fips_code)[T.53061]                       -4.7810      1.239     -3.858      0.000      -7.210      -2.352
C(fips_code)[T.53063]                       -1.8635      1.239     -1.504      0.133      -4.293       0.565
C(fips_code)[T.53065]                       13.9185      1.544      9.013      0.000      10.892      16.945
C(fips_code)[T.53067]                       -1.8607      1.237     -1.504      0.133      -4.286       0.564
C(fips_code)[T.53071]                       -1.4092      1.241     -1.135      0.256      -3.842       1.024
C(fips_code)[T.53073]                       -9.2703      1.237     -7.493      0.000     -11.695      -6.845
C(fips_code)[T.53075]                      -16.0077      1.249    -12.812      0.000     -18.457     -13.559
C(fips_code)[T.53077]                       -0.1988      1.243     -0.160      0.873      -2.635       2.238
C(fips_code)[T.54003]                        3.4281      1.217      2.818      0.005       1.043       5.813
C(fips_code)[T.54011]                       -0.6734      1.213     -0.555      0.579      -3.052       1.705
C(fips_code)[T.54033]                       -4.8308      1.217     -3.970      0.000      -7.216      -2.446
C(fips_code)[T.54037]                        1.7451      1.213      1.439      0.150      -0.633       4.123
C(fips_code)[T.54039]                       -0.2585      1.214     -0.213      0.831      -2.639       2.122
C(fips_code)[T.54049]                        4.7988      1.213      3.957      0.000       2.422       7.176
C(fips_code)[T.54055]                        1.6570      1.223      1.355      0.175      -0.739       4.053
C(fips_code)[T.54061]                       -7.2926      1.212     -6.015      0.000      -9.669      -4.916
C(fips_code)[T.54069]                       -4.8278      1.217     -3.967      0.000      -7.213      -2.443
C(fips_code)[T.54079]                        6.8004      1.216      5.595      0.000       4.418       9.183
C(fips_code)[T.54081]                        3.2416      1.219      2.660      0.008       0.853       5.630
C(fips_code)[T.54107]                       -1.7144      1.219     -1.406      0.160      -4.104       0.675
C(fips_code)[T.55005]                        2.8242      1.251      2.257      0.024       0.371       5.277
C(fips_code)[T.55009]                       -9.8519      1.250     -7.883      0.000     -12.301      -7.402
C(fips_code)[T.55015]                      -11.6208      1.250     -9.297      0.000     -14.071      -9.171
C(fips_code)[T.55017]                        0.7402      1.250      0.592      0.554      -1.710       3.190
C(fips_code)[T.55019]                       -2.9548      2.082     -1.419      0.156      -7.036       1.126
C(fips_code)[T.55021]                        5.1456      1.248      4.124      0.000       2.700       7.591
C(fips_code)[T.55025]                       -9.4326      1.251     -7.538      0.000     -11.885      -6.980
C(fips_code)[T.55027]                        5.9640      1.250      4.773      0.000       3.515       8.413
C(fips_code)[T.55031]                        7.2009      1.258      5.722      0.000       4.734       9.667
C(fips_code)[T.55033]                        7.3123      1.249      5.856      0.000       4.865       9.760
C(fips_code)[T.55035]                       -7.0039      1.249     -5.609      0.000      -9.452      -4.556
C(fips_code)[T.55039]                       -1.1725      1.249     -0.939      0.348      -3.620       1.275
C(fips_code)[T.55043]                        7.6333      1.249      6.112      0.000       5.185      10.081
C(fips_code)[T.55045]                        1.7135      1.250      1.371      0.171      -0.737       4.164
C(fips_code)[T.55055]                       -0.4737      1.249     -0.379      0.705      -2.922       1.975
C(fips_code)[T.55057]                        5.6468      2.327      2.426      0.015       1.085      10.209
C(fips_code)[T.55059]                        0.4465      1.249      0.358      0.721      -2.001       2.895
C(fips_code)[T.55063]                       -5.9228      1.248     -4.744      0.000      -8.370      -3.476
C(fips_code)[T.55069]                        3.8674      1.650      2.344      0.019       0.634       7.101
C(fips_code)[T.55071]                       -3.0718      1.249     -2.459      0.014      -5.521      -0.623
C(fips_code)[T.55073]                       -6.7286      1.250     -5.381      0.000      -9.179      -4.278
C(fips_code)[T.55075]                        2.8697      1.253      2.291      0.022       0.414       5.325
C(fips_code)[T.55079]                       -0.9903      1.249     -0.793      0.428      -3.438       1.458
C(fips_code)[T.55081]                        2.2856      1.250      1.828      0.068      -0.165       4.736
C(fips_code)[T.55083]                      -13.5057      2.328     -5.802      0.000     -18.069      -8.943
C(fips_code)[T.55085]                       -0.1274      1.253     -0.102      0.919      -2.584       2.329
C(fips_code)[T.55087]                      -11.1806      1.248     -8.957      0.000     -13.627      -8.734
C(fips_code)[T.55089]                      -14.2415      1.251    -11.383      0.000     -16.694     -11.789
C(fips_code)[T.55093]                        7.4139      1.648      4.499      0.000       4.183      10.644
C(fips_code)[T.55095]                       19.4533      1.249     15.572      0.000      17.005      21.902
C(fips_code)[T.55097]                        0.2490      1.249      0.199      0.842      -2.199       2.697
C(fips_code)[T.55101]                        0.1111      1.250      0.089      0.929      -2.338       2.560
C(fips_code)[T.55105]                        6.2757      1.248      5.027      0.000       3.829       8.723
C(fips_code)[T.55109]                        6.1494      1.249      4.922      0.000       3.701       8.598
C(fips_code)[T.55111]                        6.6625      1.251      5.327      0.000       4.211       9.114
C(fips_code)[T.55115]                        1.9153      1.250      1.532      0.125      -0.535       4.365
C(fips_code)[T.55117]                       -3.0630      1.250     -2.451      0.014      -5.513      -0.613
C(fips_code)[T.55121]                        5.3863      2.082      2.588      0.010       1.306       9.467
C(fips_code)[T.55127]                       10.4928      1.248      8.407      0.000       8.046      12.939
C(fips_code)[T.55131]                       -3.5486      1.251     -2.836      0.005      -6.002      -1.096
C(fips_code)[T.55133]                      -14.1088      1.251    -11.276      0.000     -16.561     -11.656
C(fips_code)[T.55135]                        5.4800      1.250      4.385      0.000       3.030       7.929
C(fips_code)[T.55139]                       -6.7729      1.249     -5.423      0.000      -9.221      -4.325
C(fips_code)[T.55141]                        4.3809      1.249      3.507      0.000       1.932       6.829
C(fips_code)[T.56001]                       -5.9460      1.220     -4.875      0.000      -8.337      -3.555
C(fips_code)[T.56005]                       -2.1600      1.224     -1.765      0.078      -4.558       0.238
C(fips_code)[T.56013]                        5.4270      1.220      4.448      0.000       3.035       7.819
C(fips_code)[T.56021]                       -2.6298      1.214     -2.166      0.030      -5.009      -0.250
C(fips_code)[T.56025]                        2.1756      1.218      1.786      0.074      -0.212       4.563
C(fips_code)[T.56029]                        2.3315      1.819      1.282      0.200      -1.235       5.898
C(fips_code)[T.56033]                       -3.1156      1.223     -2.548      0.011      -5.512      -0.719
C(fips_code)[T.56037]                        2.5844      1.217      2.124      0.034       0.199       4.970
C(week)[T.11]:C(state)[T.Alaska]            -4.5198      3.804     -1.188      0.235     -11.976       2.936
C(week)[T.12]:C(state)[T.Alaska]             0.3229      3.534      0.091      0.927      -6.605       7.250
C(week)[T.13]:C(state)[T.Alaska]            -5.5507      3.563     -1.558      0.119     -12.535       1.434
C(week)[T.14]:C(state)[T.Alaska]            -5.6194      3.533     -1.591      0.112     -12.544       1.305
C(week)[T.15]:C(state)[T.Alaska]             1.2712      3.540      0.359      0.720      -5.667       8.210
C(week)[T.16]:C(state)[T.Alaska]            -0.0634      3.540     -0.018      0.986      -7.002       6.875
C(week)[T.17]:C(state)[T.Alaska]             3.2597      3.536      0.922      0.357      -3.672      10.191
C(week)[T.18]:C(state)[T.Alaska]             4.7531      3.566      1.333      0.183      -2.237      11.743
C(week)[T.19]:C(state)[T.Alaska]             9.9454      3.532      2.815      0.005       3.021      16.869
C(week)[T.20]:C(state)[T.Alaska]            11.5752      3.532      3.277      0.001       4.651      18.499
C(week)[T.21]:C(state)[T.Alaska]            13.2487      3.532      3.751      0.000       6.325      20.173
C(week)[T.22]:C(state)[T.Alaska]            10.2404      3.805      2.692      0.007       2.783      17.698
C(week)[T.23]:C(state)[T.Alaska]             8.3076      3.805      2.183      0.029       0.850      15.765
C(week)[T.11]:C(state)[T.Arizona]           -7.6601      2.606     -2.939      0.003     -12.769      -2.552
C(week)[T.12]:C(state)[T.Arizona]            1.3071      2.191      0.597      0.551      -2.987       5.601
C(week)[T.13]:C(state)[T.Arizona]           -4.0484      2.191     -1.848      0.065      -8.342       0.245
C(week)[T.14]:C(state)[T.Arizona]           -4.7801      2.193     -2.179      0.029      -9.079      -0.481
C(week)[T.15]:C(state)[T.Arizona]           -4.1483      2.207     -1.880      0.060      -8.474       0.177
C(week)[T.16]:C(state)[T.Arizona]          -10.1209      2.207     -4.586      0.000     -14.446      -5.795
C(week)[T.17]:C(state)[T.Arizona]          -10.2129      2.201     -4.640      0.000     -14.527      -5.899
C(week)[T.18]:C(state)[T.Arizona]          -13.5040      2.196     -6.151      0.000     -17.807      -9.200
C(week)[T.19]:C(state)[T.Arizona]          -12.4115      2.247     -5.523      0.000     -16.816      -8.007
C(week)[T.20]:C(state)[T.Arizona]          -12.2347      2.247     -5.444      0.000     -16.640      -7.830
C(week)[T.21]:C(state)[T.Arizona]           -9.7821      2.195     -4.456      0.000     -14.085      -5.479
C(week)[T.22]:C(state)[T.Arizona]           -8.8519      2.196     -4.032      0.000     -13.155      -4.548
C(week)[T.23]:C(state)[T.Arizona]          -12.1389      2.193     -5.535      0.000     -16.438      -7.840
C(week)[T.11]:C(state)[T.California]        -2.8296      1.533     -1.846      0.065      -5.835       0.176
C(week)[T.12]:C(state)[T.California]        -0.0120      1.610     -0.007      0.994      -3.168       3.144
C(week)[T.13]:C(state)[T.California]        -1.7528      1.614     -1.086      0.277      -4.916       1.411
C(week)[T.14]:C(state)[T.California]        -0.2039      1.555     -0.131      0.896      -3.251       2.843
C(week)[T.15]:C(state)[T.California]         1.5904      1.571      1.012      0.311      -1.489       4.670
C(week)[T.16]:C(state)[T.California]       -12.5288      1.571     -7.975      0.000     -15.608      -9.449
C(week)[T.17]:C(state)[T.California]       -18.6654      1.563    -11.943      0.000     -21.729     -15.602
C(week)[T.18]:C(state)[T.California]       -20.7436      1.554    -13.346      0.000     -23.790     -17.697
C(week)[T.19]:C(state)[T.California]       -18.3458      1.616    -11.352      0.000     -21.514     -15.178
C(week)[T.20]:C(state)[T.California]       -23.4466      1.591    -14.739      0.000     -26.565     -20.328
C(week)[T.21]:C(state)[T.California]       -23.9442      1.539    -15.557      0.000     -26.961     -20.927
C(week)[T.22]:C(state)[T.California]       -19.4158      1.539    -12.614      0.000     -22.433     -16.399
C(week)[T.23]:C(state)[T.California]       -24.5263      1.540    -15.930      0.000     -27.544     -21.508
C(week)[T.11]:C(state)[T.Colorado]          -4.5074      1.962     -2.297      0.022      -8.354      -0.661
C(week)[T.12]:C(state)[T.Colorado]          -9.6397      1.962     -4.912      0.000     -13.486      -5.793
C(week)[T.13]:C(state)[T.Colorado]          -7.5136      2.017     -3.725      0.000     -11.467      -3.560
C(week)[T.14]:C(state)[T.Colorado]          -8.8399      1.987     -4.449      0.000     -12.734      -4.945
C(week)[T.15]:C(state)[T.Colorado]          -5.0259      2.001     -2.511      0.012      -8.949      -1.103
C(week)[T.16]:C(state)[T.Colorado]         -15.9967      2.023     -7.907      0.000     -19.962     -12.031
C(week)[T.17]:C(state)[T.Colorado]         -11.5551      1.995     -5.793      0.000     -15.465      -7.645
C(week)[T.18]:C(state)[T.Colorado]         -15.4445      2.025     -7.627      0.000     -19.414     -11.475
C(week)[T.19]:C(state)[T.Colorado]         -12.3631      1.963     -6.298      0.000     -16.211      -8.515
C(week)[T.20]:C(state)[T.Colorado]         -16.3836      1.964     -8.344      0.000     -20.232     -12.535
C(week)[T.21]:C(state)[T.Colorado]         -14.0231      1.963     -7.142      0.000     -17.872     -10.175
C(week)[T.22]:C(state)[T.Colorado]          -9.1563      1.963     -4.664      0.000     -13.004      -5.308
C(week)[T.23]:C(state)[T.Colorado]         -11.4395      1.962     -5.829      0.000     -15.286      -7.593
C(week)[T.11]:C(state)[T.Connecticut]      -10.0252      2.883     -3.478      0.001     -15.675      -4.375
C(week)[T.12]:C(state)[T.Connecticut]       -5.4330      2.513     -2.162      0.031     -10.359      -0.507
C(week)[T.13]:C(state)[T.Connecticut]       -6.3569      2.559     -2.484      0.013     -11.372      -1.342
C(week)[T.14]:C(state)[T.Connecticut]       -6.2444      2.526     -2.473      0.013     -11.195      -1.294
C(week)[T.15]:C(state)[T.Connecticut]       -3.3298      2.540     -1.311      0.190      -8.309       1.649
C(week)[T.16]:C(state)[T.Connecticut]      -11.9396      2.543     -4.695      0.000     -16.924      -6.955
C(week)[T.17]:C(state)[T.Connecticut]      -13.8136      2.544     -5.431      0.000     -18.799      -8.828
C(week)[T.18]:C(state)[T.Connecticut]      -14.2192      2.528     -5.625      0.000     -19.174      -9.264
C(week)[T.19]:C(state)[T.Connecticut]      -10.6869      2.565     -4.166      0.000     -15.715      -5.659
C(week)[T.20]:C(state)[T.Connecticut]      -12.8551      2.564     -5.014      0.000     -17.881      -7.830
C(week)[T.21]:C(state)[T.Connecticut]       -9.4869      2.562     -3.702      0.000     -14.509      -4.464
C(week)[T.22]:C(state)[T.Connecticut]       -9.8211      2.514     -3.906      0.000     -14.750      -4.892
C(week)[T.23]:C(state)[T.Connecticut]      -11.4954      2.514     -4.573      0.000     -16.422      -6.568
C(week)[T.11]:C(state)[T.Delaware]          -0.0862      4.022     -0.021      0.983      -7.969       7.797
C(week)[T.12]:C(state)[T.Delaware]          -1.1455      4.022     -0.285      0.776      -9.028       6.737
C(week)[T.13]:C(state)[T.Delaware]          -5.1425      4.049     -1.270      0.204     -13.080       2.795
C(week)[T.14]:C(state)[T.Delaware]          -2.3415      4.026     -0.582      0.561     -10.233       5.550
C(week)[T.15]:C(state)[T.Delaware]           1.7583      4.032      0.436      0.663      -6.145       9.662
C(week)[T.16]:C(state)[T.Delaware]          -7.0098      4.032     -1.738      0.082     -14.914       0.894
C(week)[T.17]:C(state)[T.Delaware]          -8.1516      4.031     -2.022      0.043     -16.052      -0.251
C(week)[T.18]:C(state)[T.Delaware]         -10.7042      4.028     -2.658      0.008     -18.599      -2.809
C(week)[T.19]:C(state)[T.Delaware]          -5.7872      4.055     -1.427      0.154     -13.736       2.161
C(week)[T.20]:C(state)[T.Delaware]          -7.5350      4.056     -1.858      0.063     -15.486       0.416
C(week)[T.21]:C(state)[T.Delaware]         -12.8201      4.025     -3.185      0.001     -20.709      -4.931
C(week)[T.22]:C(state)[T.Delaware]          -8.0625      4.025     -2.003      0.045     -15.951      -0.174
C(week)[T.23]:C(state)[T.Delaware]          -6.4071      4.023     -1.593      0.111     -14.292       1.478
C(week)[T.11]:C(state)[T.Florida]           -6.4270      2.070     -3.105      0.002     -10.485      -2.369
C(week)[T.12]:C(state)[T.Florida]            3.2829      1.514      2.169      0.030       0.316       6.250
C(week)[T.13]:C(state)[T.Florida]           -2.8280      1.516     -1.866      0.062      -5.799       0.143
C(week)[T.14]:C(state)[T.Florida]           -4.2150      1.518     -2.777      0.005      -7.190      -1.240
C(week)[T.15]:C(state)[T.Florida]           -4.3525      1.538     -2.829      0.005      -7.368      -1.337
C(week)[T.16]:C(state)[T.Florida]          -11.1869      1.538     -7.272      0.000     -14.202      -8.172
C(week)[T.17]:C(state)[T.Florida]          -12.6243      1.527     -8.269      0.000     -15.617      -9.632
C(week)[T.18]:C(state)[T.Florida]          -13.5518      1.517     -8.933      0.000     -16.525     -10.578
C(week)[T.19]:C(state)[T.Florida]           -7.3276      1.589     -4.610      0.000     -10.443      -4.212
C(week)[T.20]:C(state)[T.Florida]           -9.2379      1.589     -5.813      0.000     -12.353      -6.123
C(week)[T.21]:C(state)[T.Florida]           -8.1716      1.589     -5.142      0.000     -11.287      -5.057
C(week)[T.22]:C(state)[T.Florida]           -5.2042      1.589     -3.274      0.001      -8.320      -2.089
C(week)[T.23]:C(state)[T.Florida]          -11.6386      1.588     -7.327      0.000     -14.752      -8.525
C(week)[T.11]:C(state)[T.Georgia]           -8.0528      2.057     -3.914      0.000     -12.086      -4.020
C(week)[T.12]:C(state)[T.Georgia]            2.1521      1.496      1.438      0.150      -0.781       5.085
C(week)[T.13]:C(state)[T.Georgia]           -1.2173      1.496     -0.813      0.416      -4.151       1.716
C(week)[T.14]:C(state)[T.Georgia]           -0.3916      1.498     -0.261      0.794      -3.329       2.545
C(week)[T.15]:C(state)[T.Georgia]            0.2533      1.522      0.166      0.868      -2.731       3.238
C(week)[T.16]:C(state)[T.Georgia]           -2.5991      1.522     -1.707      0.088      -5.583       0.385
C(week)[T.17]:C(state)[T.Georgia]           -2.8255      1.511     -1.870      0.061      -5.787       0.136
C(week)[T.18]:C(state)[T.Georgia]           -0.3631      1.502     -0.242      0.809      -3.308       2.581
C(week)[T.19]:C(state)[T.Georgia]           -0.0094      1.500     -0.006      0.995      -2.950       2.932
C(week)[T.20]:C(state)[T.Georgia]           -3.6539      1.497     -2.440      0.015      -6.589      -0.719
C(week)[T.21]:C(state)[T.Georgia]           -5.1103      1.497     -3.413      0.001      -8.045      -2.175
C(week)[T.22]:C(state)[T.Georgia]           -3.3865      1.497     -2.261      0.024      -6.322      -0.451
C(week)[T.23]:C(state)[T.Georgia]           -4.5580      1.500     -3.038      0.002      -7.499      -1.617
C(week)[T.11]:C(state)[T.Idaho]             -8.8844      2.660     -3.340      0.001     -14.099      -3.670
C(week)[T.12]:C(state)[T.Idaho]             -1.3343      2.660     -0.502      0.616      -6.549       3.880
C(week)[T.13]:C(state)[T.Idaho]             -1.0058      2.303     -0.437      0.662      -5.520       3.509
C(week)[T.14]:C(state)[T.Idaho]             -4.8975      2.343     -2.091      0.037      -9.489      -0.306
C(week)[T.15]:C(state)[T.Idaho]              2.9259      2.307      1.268      0.205      -1.597       7.449
C(week)[T.16]:C(state)[T.Idaho]             -1.3444      2.307     -0.583      0.560      -5.867       3.178
C(week)[T.17]:C(state)[T.Idaho]             -1.4423      2.302     -0.627      0.531      -5.954       3.069
C(week)[T.18]:C(state)[T.Idaho]             -2.8713      2.255     -1.273      0.203      -7.292       1.549
C(week)[T.19]:C(state)[T.Idaho]             -0.8496      2.255     -0.377      0.706      -5.270       3.571
C(week)[T.20]:C(state)[T.Idaho]             -5.6553      2.255     -2.508      0.012     -10.075      -1.235
C(week)[T.21]:C(state)[T.Idaho]              0.1611      2.255      0.071      0.943      -4.259       4.581
C(week)[T.22]:C(state)[T.Idaho]              5.5791      2.255      2.474      0.013       1.159       9.999
C(week)[T.23]:C(state)[T.Idaho]              2.5413      2.256      1.126      0.260      -1.881       6.963
C(week)[T.11]:C(state)[T.Illinois]         -12.8768      2.070     -6.220      0.000     -16.934      -8.819
C(week)[T.12]:C(state)[T.Illinois]         -11.8228      1.588     -7.443      0.000     -14.936      -8.709
C(week)[T.13]:C(state)[T.Illinois]         -13.4920      1.635     -8.254      0.000     -16.696     -10.288
C(week)[T.14]:C(state)[T.Illinois]          -6.3193      1.561     -4.050      0.000      -9.378      -3.261
C(week)[T.15]:C(state)[T.Illinois]          -2.3436      1.588     -1.476      0.140      -5.457       0.769
C(week)[T.16]:C(state)[T.Illinois]          -8.8930      1.594     -5.580      0.000     -12.017      -5.769
C(week)[T.17]:C(state)[T.Illinois]          -9.0173      1.575     -5.726      0.000     -12.104      -5.931
C(week)[T.18]:C(state)[T.Illinois]         -10.5853      1.569     -6.747      0.000     -13.661      -7.510
C(week)[T.19]:C(state)[T.Illinois]         -11.4598      1.636     -7.004      0.000     -14.667      -8.253
C(week)[T.20]:C(state)[T.Illinois]         -17.3643      1.632    -10.642      0.000     -20.563     -14.166
C(week)[T.21]:C(state)[T.Illinois]         -15.7179      1.632     -9.634      0.000     -18.916     -12.520
C(week)[T.22]:C(state)[T.Illinois]          -9.0939      1.631     -5.574      0.000     -12.292      -5.896
C(week)[T.23]:C(state)[T.Illinois]         -14.4131      1.552     -9.286      0.000     -17.456     -11.371
C(week)[T.11]:C(state)[T.Indiana]           -5.9170      1.514     -3.909      0.000      -8.884      -2.950
C(week)[T.12]:C(state)[T.Indiana]           -9.7637      1.514     -6.450      0.000     -12.731      -6.796
C(week)[T.13]:C(state)[T.Indiana]          -10.1455      1.584     -6.404      0.000     -13.251      -7.040
C(week)[T.14]:C(state)[T.Indiana]           -4.6174      1.536     -3.005      0.003      -7.629      -1.606
C(week)[T.15]:C(state)[T.Indiana]            1.0686      1.555      0.687      0.492      -1.979       4.116
C(week)[T.16]:C(state)[T.Indiana]           -5.1845      1.555     -3.335      0.001      -8.232      -2.137
C(week)[T.17]:C(state)[T.Indiana]           -2.6741      1.539     -1.737      0.082      -5.691       0.343
C(week)[T.18]:C(state)[T.Indiana]           -4.2473      1.531     -2.774      0.006      -7.248      -1.246
C(week)[T.19]:C(state)[T.Indiana]           -6.2616      1.528     -4.099      0.000      -9.256      -3.267
C(week)[T.20]:C(state)[T.Indiana]           -5.3112      1.517     -3.502      0.000      -8.284      -2.338
C(week)[T.21]:C(state)[T.Indiana]           -2.1745      1.517     -1.434      0.152      -5.147       0.798
C(week)[T.22]:C(state)[T.Indiana]            4.0354      1.517      2.661      0.008       1.063       7.008
C(week)[T.23]:C(state)[T.Indiana]            1.0001      1.524      0.656      0.512      -1.987       3.987
C(week)[T.11]:C(state)[T.Kansas]           -12.3427      2.323     -5.313      0.000     -16.896      -7.789
C(week)[T.12]:C(state)[T.Kansas]            -4.7461      1.855     -2.559      0.011      -8.381      -1.111
C(week)[T.13]:C(state)[T.Kansas]            -4.5432      1.848     -2.458      0.014      -8.166      -0.920
C(week)[T.14]:C(state)[T.Kansas]            -9.9220      1.849     -5.367      0.000     -13.546      -6.298
C(week)[T.15]:C(state)[T.Kansas]            -2.8102      1.877     -1.497      0.134      -6.489       0.869
C(week)[T.16]:C(state)[T.Kansas]            -8.8172      1.863     -4.733      0.000     -12.469      -5.166
C(week)[T.17]:C(state)[T.Kansas]            -7.5116      1.870     -4.017      0.000     -11.177      -3.846
C(week)[T.18]:C(state)[T.Kansas]            -7.3024      1.850     -3.946      0.000     -10.929      -3.675
C(week)[T.19]:C(state)[T.Kansas]            -5.0875      1.909     -2.666      0.008      -8.828      -1.346
C(week)[T.20]:C(state)[T.Kansas]           -10.3507      1.907     -5.427      0.000     -14.089      -6.613
C(week)[T.21]:C(state)[T.Kansas]           -13.8772      2.323     -5.973      0.000     -18.431      -9.323
C(week)[T.22]:C(state)[T.Kansas]           -12.3620      2.323     -5.321      0.000     -16.916      -7.808
C(week)[T.23]:C(state)[T.Kansas]           -14.8903      2.323     -6.410      0.000     -19.444     -10.337
C(week)[T.11]:C(state)[T.Kentucky]         -14.3655      2.228     -6.447      0.000     -18.733      -9.998
C(week)[T.12]:C(state)[T.Kentucky]          -9.0925      1.724     -5.276      0.000     -12.471      -5.714
C(week)[T.13]:C(state)[T.Kentucky]          -3.9926      1.795     -2.224      0.026      -7.511      -0.474
C(week)[T.14]:C(state)[T.Kentucky]          -3.2979      1.756     -1.878      0.060      -6.740       0.144
C(week)[T.15]:C(state)[T.Kentucky]          -0.6734      1.770     -0.380      0.704      -4.143       2.796
C(week)[T.16]:C(state)[T.Kentucky]          -6.0705      1.770     -3.429      0.001      -9.540      -2.601
C(week)[T.17]:C(state)[T.Kentucky]          -7.4833      1.763     -4.245      0.000     -10.939      -4.028
C(week)[T.18]:C(state)[T.Kentucky]          -8.7009      1.745     -4.986      0.000     -12.121      -5.281
C(week)[T.19]:C(state)[T.Kentucky]          -9.3592      1.799     -5.202      0.000     -12.886      -5.833
C(week)[T.20]:C(state)[T.Kentucky]         -14.1697      1.799     -7.876      0.000     -17.696     -10.643
C(week)[T.21]:C(state)[T.Kentucky]         -12.4256      1.790     -6.943      0.000     -15.933      -8.918
C(week)[T.22]:C(state)[T.Kentucky]          -2.2027      1.790     -1.231      0.218      -5.711       1.305
C(week)[T.23]:C(state)[T.Kentucky]          -9.6049      1.730     -5.552      0.000     -12.996      -6.214
C(week)[T.11]:C(state)[T.Louisiana]         -9.1711      2.241     -4.092      0.000     -13.564      -4.778
C(week)[T.12]:C(state)[T.Louisiana]         -4.1713      1.740     -2.397      0.017      -7.583      -0.760
C(week)[T.13]:C(state)[T.Louisiana]         -5.9310      1.814     -3.269      0.001      -9.487      -2.375
C(week)[T.14]:C(state)[T.Louisiana]         -3.9052      1.757     -2.223      0.026      -7.348      -0.462
C(week)[T.15]:C(state)[T.Louisiana]         -4.8452      1.770     -2.737      0.006      -8.315      -1.375
C(week)[T.16]:C(state)[T.Louisiana]         -7.2677      1.770     -4.106      0.000     -10.737      -3.798
C(week)[T.17]:C(state)[T.Louisiana]         -6.1119      1.762     -3.469      0.001      -9.566      -2.658
C(week)[T.18]:C(state)[T.Louisiana]         -8.0659      1.753     -4.602      0.000     -11.501      -4.631
C(week)[T.19]:C(state)[T.Louisiana]         -5.8823      1.809     -3.252      0.001      -9.427      -2.337
C(week)[T.20]:C(state)[T.Louisiana]         -9.9611      1.809     -5.508      0.000     -13.506      -6.416
C(week)[T.21]:C(state)[T.Louisiana]         -7.1453      1.741     -4.104      0.000     -10.558      -3.732
C(week)[T.22]:C(state)[T.Louisiana]         -5.7239      1.741     -3.287      0.001      -9.137      -2.311
C(week)[T.23]:C(state)[T.Louisiana]         -7.9582      1.741     -4.571      0.000     -11.371      -4.545
C(week)[T.11]:C(state)[T.Maine]             -9.7752      2.606     -3.751      0.000     -14.884      -4.667
C(week)[T.12]:C(state)[T.Maine]             -7.9200      2.191     -3.616      0.000     -12.214      -3.626
C(week)[T.13]:C(state)[T.Maine]             -9.5128      2.327     -4.089      0.000     -14.073      -4.952
C(week)[T.14]:C(state)[T.Maine]            -10.5900      2.391     -4.428      0.000     -15.278      -5.903
C(week)[T.15]:C(state)[T.Maine]             -7.7478      2.403     -3.224      0.001     -12.458      -3.037
C(week)[T.16]:C(state)[T.Maine]             -7.7511      2.403     -3.226      0.001     -12.461      -3.041
C(week)[T.17]:C(state)[T.Maine]             -9.4411      2.398     -3.937      0.000     -14.141      -4.741
C(week)[T.18]:C(state)[T.Maine]            -12.8778      2.391     -5.385      0.000     -17.565      -8.191
C(week)[T.19]:C(state)[T.Maine]             -8.9283      2.434     -3.668      0.000     -13.700      -4.157
C(week)[T.20]:C(state)[T.Maine]             -9.1106      2.377     -3.833      0.000     -13.769      -4.452
C(week)[T.21]:C(state)[T.Maine]             -2.7140      2.327     -1.166      0.243      -7.275       1.847
C(week)[T.22]:C(state)[T.Maine]             -0.1590      2.283     -0.070      0.944      -4.635       4.317
C(week)[T.23]:C(state)[T.Maine]             -4.3666      2.245     -1.945      0.052      -8.766       0.033
C(week)[T.11]:C(state)[T.Maryland]           0.2023      1.898      0.107      0.915      -3.519       3.924
C(week)[T.12]:C(state)[T.Maryland]          -0.7911      1.898     -0.417      0.677      -4.512       2.930
C(week)[T.13]:C(state)[T.Maryland]          -6.0128      1.899     -3.167      0.002      -9.734      -2.291
C(week)[T.14]:C(state)[T.Maryland]          -4.5330      1.907     -2.378      0.017      -8.270      -0.796
C(week)[T.15]:C(state)[T.Maryland]          -0.6813      1.938     -0.352      0.725      -4.480       3.118
C(week)[T.16]:C(state)[T.Maryland]          -9.4899      1.938     -4.897      0.000     -13.289      -5.691
C(week)[T.17]:C(state)[T.Maryland]         -11.7678      1.950     -6.035      0.000     -15.590      -7.946
C(week)[T.18]:C(state)[T.Maryland]         -13.9611      1.924     -7.256      0.000     -17.733     -10.190
C(week)[T.19]:C(state)[T.Maryland]         -10.6725      1.976     -5.400      0.000     -14.546      -6.799
C(week)[T.20]:C(state)[T.Maryland]         -11.9040      1.960     -6.073      0.000     -15.746      -8.062
C(week)[T.21]:C(state)[T.Maryland]         -10.5811      1.960     -5.398      0.000     -14.423      -6.739
C(week)[T.22]:C(state)[T.Maryland]          -3.3769      1.960     -1.723      0.085      -7.219       0.465
C(week)[T.23]:C(state)[T.Maryland]         -10.8849      1.899     -5.733      0.000     -14.607      -7.163
C(week)[T.11]:C(state)[T.Massachusetts]    -10.3325      2.660     -3.884      0.000     -15.547      -5.118
C(week)[T.12]:C(state)[T.Massachusetts]     -9.0565      2.255     -4.017      0.000     -13.476      -4.637
C(week)[T.13]:C(state)[T.Massachusetts]     -8.3484      2.309     -3.615      0.000     -12.875      -3.822
C(week)[T.14]:C(state)[T.Massachusetts]     -5.6215      2.272     -2.474      0.013     -10.076      -1.167
C(week)[T.15]:C(state)[T.Massachusetts]      0.0333      2.286      0.015      0.988      -4.448       4.514
C(week)[T.16]:C(state)[T.Massachusetts]    -10.1493      2.288     -4.437      0.000     -14.633      -5.665
C(week)[T.17]:C(state)[T.Massachusetts]    -11.0064      2.285     -4.817      0.000     -15.485      -6.527
C(week)[T.18]:C(state)[T.Massachusetts]    -12.8840      2.285     -5.639      0.000     -17.363      -8.405
C(week)[T.19]:C(state)[T.Massachusetts]    -10.2174      2.329     -4.387      0.000     -14.782      -5.653
C(week)[T.20]:C(state)[T.Massachusetts]    -12.6347      2.328     -5.428      0.000     -17.197      -8.072
C(week)[T.21]:C(state)[T.Massachusetts]    -15.3900      2.269     -6.783      0.000     -19.837     -10.943
C(week)[T.22]:C(state)[T.Massachusetts]    -11.2163      2.268     -4.945      0.000     -15.662      -6.771
C(week)[T.23]:C(state)[T.Massachusetts]    -15.7894      2.277     -6.934      0.000     -20.253     -11.326
C(week)[T.11]:C(state)[T.Michigan]         -13.8725      2.106     -6.586      0.000     -18.001      -9.744
C(week)[T.12]:C(state)[T.Michigan]         -15.4047      1.563     -9.857      0.000     -18.468     -12.341
C(week)[T.13]:C(state)[T.Michigan]         -19.9430      1.631    -12.228      0.000     -23.140     -16.746
C(week)[T.14]:C(state)[T.Michigan]         -11.4522      1.597     -7.169      0.000     -14.583      -8.321
C(week)[T.15]:C(state)[T.Michigan]          -7.1019      1.633     -4.349      0.000     -10.303      -3.901
C(week)[T.16]:C(state)[T.Michigan]         -14.0385      1.633     -8.596      0.000     -17.240     -10.837
C(week)[T.17]:C(state)[T.Michigan]         -11.7658      1.625     -7.241      0.000     -14.951      -8.581
C(week)[T.18]:C(state)[T.Michigan]         -11.5511      1.680     -6.874      0.000     -14.845      -8.257
C(week)[T.19]:C(state)[T.Michigan]         -11.1808      1.600     -6.990      0.000     -14.316      -8.045
C(week)[T.20]:C(state)[T.Michigan]         -15.6067      1.595     -9.786      0.000     -18.733     -12.481
C(week)[T.21]:C(state)[T.Michigan]         -11.0689      1.595     -6.940      0.000     -14.195      -7.943
C(week)[T.22]:C(state)[T.Michigan]          -6.3744      1.595     -3.997      0.000      -9.501      -3.248
C(week)[T.23]:C(state)[T.Michigan]         -11.3557      1.576     -7.206      0.000     -14.445      -8.267
C(week)[T.11]:C(state)[T.Minnesota]         -8.6717      2.156     -4.022      0.000     -12.897      -4.446
C(week)[T.12]:C(state)[T.Minnesota]         -9.8854      1.629     -6.068      0.000     -13.078      -6.692
C(week)[T.13]:C(state)[T.Minnesota]         -7.4706      1.695     -4.408      0.000     -10.792      -4.149
C(week)[T.14]:C(state)[T.Minnesota]        -10.2314      1.652     -6.195      0.000     -13.469      -6.994
C(week)[T.15]:C(state)[T.Minnesota]         -6.5269      1.694     -3.854      0.000      -9.847      -3.207
C(week)[T.16]:C(state)[T.Minnesota]        -10.6612      1.679     -6.350      0.000     -13.952      -7.370
C(week)[T.17]:C(state)[T.Minnesota]         -8.3731      1.671     -5.010      0.000     -11.649      -5.097
C(week)[T.18]:C(state)[T.Minnesota]         -9.1703      1.658     -5.531      0.000     -12.420      -5.920
C(week)[T.19]:C(state)[T.Minnesota]         -8.3346      1.712     -4.867      0.000     -11.691      -4.978
C(week)[T.20]:C(state)[T.Minnesota]        -12.7194      1.706     -7.456      0.000     -16.063      -9.376
C(week)[T.21]:C(state)[T.Minnesota]        -11.1647      1.636     -6.823      0.000     -14.372      -7.957
C(week)[T.22]:C(state)[T.Minnesota]         -8.5009      1.636     -5.195      0.000     -11.708      -5.294
C(week)[T.23]:C(state)[T.Minnesota]         -5.6327      1.636     -3.443      0.001      -8.839      -2.426
C(week)[T.11]:C(state)[T.Mississippi]       -8.3808      2.447     -3.424      0.001     -13.178      -3.584
C(week)[T.12]:C(state)[T.Mississippi]       -8.4230      2.401     -3.508      0.000     -13.129      -3.717
C(week)[T.13]:C(state)[T.Mississippi]       -4.0241      2.026     -1.986      0.047      -7.996      -0.053
C(week)[T.14]:C(state)[T.Mississippi]       -4.2285      2.076     -2.037      0.042      -8.297      -0.160
C(week)[T.15]:C(state)[T.Mississippi]       -4.2273      2.039     -2.073      0.038      -8.224      -0.231
C(week)[T.16]:C(state)[T.Mississippi]       -5.1979      2.039     -2.549      0.011      -9.194      -1.201
C(week)[T.17]:C(state)[T.Mississippi]       -3.4380      2.033     -1.692      0.091      -7.422       0.546
C(week)[T.18]:C(state)[T.Mississippi]       -6.4765      2.083     -3.110      0.002     -10.558      -2.395
C(week)[T.19]:C(state)[T.Mississippi]       -3.6628      2.026     -1.808      0.071      -7.635       0.309
C(week)[T.20]:C(state)[T.Mississippi]       -5.5354      2.026     -2.732      0.006      -9.507      -1.564
C(week)[T.21]:C(state)[T.Mississippi]       -4.1352      2.026     -2.041      0.041      -8.107      -0.163
C(week)[T.22]:C(state)[T.Mississippi]       -3.1923      2.027     -1.575      0.115      -7.165       0.781
C(week)[T.23]:C(state)[T.Mississippi]       -9.0480      2.454     -3.687      0.000     -13.859      -4.237
C(week)[T.11]:C(state)[T.Missouri]         -11.5992      2.156     -5.380      0.000     -15.825      -7.374
C(week)[T.12]:C(state)[T.Missouri]          -5.2597      1.629     -3.229      0.001      -8.453      -2.067
C(week)[T.13]:C(state)[T.Missouri]          -9.2938      1.633     -5.693      0.000     -12.494      -6.094
C(week)[T.14]:C(state)[T.Missouri]         -10.4607      1.674     -6.251      0.000     -13.741      -7.180
C(week)[T.15]:C(state)[T.Missouri]          -9.5358      1.650     -5.779      0.000     -12.770      -6.301
C(week)[T.16]:C(state)[T.Missouri]         -10.1581      1.650     -6.155      0.000     -13.393      -6.923
C(week)[T.17]:C(state)[T.Missouri]         -10.2168      1.643     -6.220      0.000     -13.436      -6.997
C(week)[T.18]:C(state)[T.Missouri]          -8.0564      1.631     -4.938      0.000     -11.254      -4.859
C(week)[T.19]:C(state)[T.Missouri]          -1.4537      1.629     -0.892      0.372      -4.648       1.740
C(week)[T.20]:C(state)[T.Missouri]          -7.2923      1.629     -4.476      0.000     -10.486      -4.099
C(week)[T.21]:C(state)[T.Missouri]          -2.0919      1.629     -1.284      0.199      -5.286       1.102
C(week)[T.22]:C(state)[T.Missouri]          -0.3948      1.630     -0.242      0.809      -3.589       2.799
C(week)[T.23]:C(state)[T.Missouri]          -3.6924      1.629     -2.266      0.023      -6.886      -0.499
C(week)[T.11]:C(state)[T.Montana]          -13.2571      2.987     -4.438      0.000     -19.113      -7.401
C(week)[T.12]:C(state)[T.Montana]           -6.4547      2.633     -2.452      0.014     -11.615      -1.294
C(week)[T.13]:C(state)[T.Montana]           -5.8675      2.674     -2.194      0.028     -11.108      -0.627
C(week)[T.14]:C(state)[T.Montana]          -10.9130      2.634     -4.143      0.000     -16.076      -5.750
C(week)[T.15]:C(state)[T.Montana]           -3.0347      2.644     -1.148      0.251      -8.217       2.148
C(week)[T.16]:C(state)[T.Montana]           -9.3077      2.644     -3.520      0.000     -14.490      -4.125
C(week)[T.17]:C(state)[T.Montana]           -8.3131      2.639     -3.150      0.002     -13.486      -3.140
C(week)[T.18]:C(state)[T.Montana]           -6.4285      2.715     -2.368      0.018     -11.750      -1.107
C(week)[T.19]:C(state)[T.Montana]            2.0835      2.756      0.756      0.450      -3.318       7.485
C(week)[T.20]:C(state)[T.Montana]           -3.3130      2.633     -1.258      0.208      -8.475       1.849
C(week)[T.21]:C(state)[T.Montana]           -0.8702      2.633     -0.330      0.741      -6.032       4.291
C(week)[T.22]:C(state)[T.Montana]            4.4909      2.633      1.705      0.088      -0.671       9.653
C(week)[T.23]:C(state)[T.Montana]            4.3068      2.633      1.636      0.102      -0.855       9.468
C(week)[T.11]:C(state)[T.Nevada]            -7.7109      3.117     -2.474      0.013     -13.821      -1.601
C(week)[T.12]:C(state)[T.Nevada]            -0.3739      2.779     -0.135      0.893      -5.821       5.074
C(week)[T.13]:C(state)[T.Nevada]            -1.1530      2.779     -0.415      0.678      -6.601       4.294
C(week)[T.14]:C(state)[T.Nevada]             4.7260      2.781      1.699      0.089      -0.726      10.178
C(week)[T.15]:C(state)[T.Nevada]             7.8825      2.792      2.824      0.005       2.411      13.354
C(week)[T.16]:C(state)[T.Nevada]             4.0937      2.792      1.466      0.143      -1.378       9.566
C(week)[T.17]:C(state)[T.Nevada]             3.7556      2.787      1.348      0.178      -1.707       9.218
C(week)[T.18]:C(state)[T.Nevada]             0.9396      2.783      0.338      0.736      -4.514       6.394
C(week)[T.19]:C(state)[T.Nevada]             1.9920      2.823      0.706      0.480      -3.542       7.526
C(week)[T.20]:C(state)[T.Nevada]            -0.8575      2.823     -0.304      0.761      -6.392       4.677
C(week)[T.21]:C(state)[T.Nevada]             0.9856      2.823      0.349      0.727      -4.549       6.520
C(week)[T.22]:C(state)[T.Nevada]             5.8762      2.823      2.081      0.037       0.342      11.410
C(week)[T.23]:C(state)[T.Nevada]            -0.3920      2.821     -0.139      0.889      -5.922       5.138
C(week)[T.11]:C(state)[T.New Hampshire]     -8.2367      2.796     -2.946      0.003     -13.717      -2.757
C(week)[T.12]:C(state)[T.New Hampshire]     -3.1706      2.413     -1.314      0.189      -7.900       1.559
C(week)[T.13]:C(state)[T.New Hampshire]     -2.5001      2.516     -0.994      0.320      -7.432       2.432
C(week)[T.14]:C(state)[T.New Hampshire]     -0.7945      2.479     -0.321      0.749      -5.654       4.064
C(week)[T.15]:C(state)[T.New Hampshire]      5.3557      2.491      2.150      0.032       0.473      10.238
C(week)[T.16]:C(state)[T.New Hampshire]     -0.3699      2.491     -0.148      0.882      -5.253       4.513
C(week)[T.17]:C(state)[T.New Hampshire]     -0.7400      2.486     -0.298      0.766      -5.612       4.133
C(week)[T.18]:C(state)[T.New Hampshire]     -2.8545      2.483     -1.150      0.250      -7.721       2.012
C(week)[T.19]:C(state)[T.New Hampshire]     -0.3744      2.528     -0.148      0.882      -5.330       4.582
C(week)[T.20]:C(state)[T.New Hampshire]      0.1181      2.528      0.047      0.963      -4.838       5.074
C(week)[T.21]:C(state)[T.New Hampshire]     10.9487      2.471      4.431      0.000       6.105      15.792
C(week)[T.22]:C(state)[T.New Hampshire]     13.5795      2.471      5.495      0.000       8.736      18.423
C(week)[T.23]:C(state)[T.New Hampshire]      3.9223      2.466      1.591      0.112      -0.911       8.755
C(week)[T.11]:C(state)[T.New Jersey]        -8.6979      2.344     -3.711      0.000     -13.292      -4.104
C(week)[T.12]:C(state)[T.New Jersey]        -4.6608      1.933     -2.411      0.016      -8.450      -0.871
C(week)[T.13]:C(state)[T.New Jersey]       -11.8844      1.930     -6.157      0.000     -15.668      -8.101
C(week)[T.14]:C(state)[T.New Jersey]       -11.1362      1.885     -5.909      0.000     -14.830      -7.442
C(week)[T.15]:C(state)[T.New Jersey]       -10.9329      1.904     -5.741      0.000     -14.666      -7.200
C(week)[T.16]:C(state)[T.New Jersey]       -21.6526      1.910    -11.335      0.000     -25.397     -17.908
C(week)[T.17]:C(state)[T.New Jersey]       -23.8006      1.899    -12.533      0.000     -27.523     -20.078
C(week)[T.18]:C(state)[T.New Jersey]       -24.6918      1.901    -12.991      0.000     -28.418     -20.966
C(week)[T.19]:C(state)[T.New Jersey]       -21.8074      1.949    -11.187      0.000     -25.628     -17.987
C(week)[T.20]:C(state)[T.New Jersey]       -22.5935      1.947    -11.606      0.000     -26.409     -18.778
C(week)[T.21]:C(state)[T.New Jersey]       -22.6310      1.944    -11.639      0.000     -26.442     -18.820
C(week)[T.22]:C(state)[T.New Jersey]       -17.4949      1.942     -9.007      0.000     -21.302     -13.687
C(week)[T.23]:C(state)[T.New Jersey]       -23.9765      1.950    -12.296      0.000     -27.799     -20.154
C(week)[T.11]:C(state)[T.New Mexico]        -9.1539      2.606     -3.512      0.000     -14.262      -4.045
C(week)[T.12]:C(state)[T.New Mexico]         0.1961      2.191      0.090      0.929      -4.098       4.490
C(week)[T.13]:C(state)[T.New Mexico]        -3.4532      2.240     -1.542      0.123      -7.844       0.938
C(week)[T.14]:C(state)[T.New Mexico]        -1.9581      2.234     -0.876      0.381      -6.338       2.421
C(week)[T.15]:C(state)[T.New Mexico]        -1.8205      2.249     -0.810      0.418      -6.229       2.588
C(week)[T.16]:C(state)[T.New Mexico]        -8.2420      2.249     -3.665      0.000     -12.650      -3.834
C(week)[T.17]:C(state)[T.New Mexico]        -6.9404      2.243     -3.094      0.002     -11.337      -2.544
C(week)[T.18]:C(state)[T.New Mexico]       -10.1579      2.201     -4.616      0.000     -14.471      -5.844
C(week)[T.19]:C(state)[T.New Mexico]       -12.9405      2.281     -5.674      0.000     -17.411      -8.470
C(week)[T.20]:C(state)[T.New Mexico]       -17.6982      2.194     -8.067      0.000     -21.999     -13.398
C(week)[T.21]:C(state)[T.New Mexico]       -15.9227      2.194     -7.258      0.000     -20.223     -11.622
C(week)[T.22]:C(state)[T.New Mexico]       -14.4059      2.194     -6.566      0.000     -18.706     -10.105
C(week)[T.23]:C(state)[T.New Mexico]        -9.6411      2.191     -4.399      0.000     -13.937      -5.346
C(week)[T.11]:C(state)[T.New York]          -2.8283      1.496     -1.890      0.059      -5.762       0.105
C(week)[T.12]:C(state)[T.New York]          -4.0363      1.574     -2.564      0.010      -7.122      -0.951
C(week)[T.13]:C(state)[T.New York]         -11.1552      1.577     -7.076      0.000     -14.245      -8.065
C(week)[T.14]:C(state)[T.New York]          -6.4933      1.515     -4.287      0.000      -9.462      -3.524
C(week)[T.15]:C(state)[T.New York]          -9.0838      1.550     -5.861      0.000     -12.122      -6.046
C(week)[T.16]:C(state)[T.New York]         -18.6077      1.545    -12.045      0.000     -21.636     -15.580
C(week)[T.17]:C(state)[T.New York]         -20.0847      1.537    -13.068      0.000     -23.097     -17.072
C(week)[T.18]:C(state)[T.New York]         -20.3547      1.528    -13.320      0.000     -23.350     -17.359
C(week)[T.19]:C(state)[T.New York]         -21.0591      1.594    -13.208      0.000     -24.184     -17.934
C(week)[T.20]:C(state)[T.New York]         -21.5963      1.578    -13.683      0.000     -24.690     -18.503
C(week)[T.21]:C(state)[T.New York]         -16.9316      1.578    -10.732      0.000     -20.024     -13.839
C(week)[T.22]:C(state)[T.New York]         -15.9375      1.574    -10.122      0.000     -19.024     -12.851
C(week)[T.23]:C(state)[T.New York]         -20.8106      1.582    -13.157      0.000     -23.911     -17.710
C(week)[T.11]:C(state)[T.North Carolina]     0.8796      1.424      0.617      0.537      -1.913       3.672
C(week)[T.12]:C(state)[T.North Carolina]     1.3623      1.424      0.956      0.339      -1.430       4.154
C(week)[T.13]:C(state)[T.North Carolina]     1.9128      1.425      1.343      0.179      -0.880       4.705
C(week)[T.14]:C(state)[T.North Carolina]     0.6508      1.433      0.454      0.650      -2.157       3.459
C(week)[T.15]:C(state)[T.North Carolina]     4.1846      1.457      2.873      0.004       1.329       7.040
C(week)[T.16]:C(state)[T.North Carolina]     0.8977      1.457      0.616      0.538      -1.958       3.753
C(week)[T.17]:C(state)[T.North Carolina]    -2.3218      1.448     -1.604      0.109      -5.160       0.516
C(week)[T.18]:C(state)[T.North Carolina]    -0.8826      1.434     -0.616      0.538      -3.693       1.928
C(week)[T.19]:C(state)[T.North Carolina]     1.3751      1.511      0.910      0.363      -1.586       4.337
C(week)[T.20]:C(state)[T.North Carolina]    -1.8686      1.505     -1.241      0.214      -4.819       1.082
C(week)[T.21]:C(state)[T.North Carolina]    -5.7532      1.507     -3.817      0.000      -8.707      -2.799
C(week)[T.22]:C(state)[T.North Carolina]     0.0883      1.426      0.062      0.951      -2.707       2.883
C(week)[T.23]:C(state)[T.North Carolina]    -2.7217      1.429     -1.905      0.057      -5.523       0.079
C(week)[T.11]:C(state)[T.Ohio]              -4.0020      1.415     -2.828      0.005      -6.776      -1.228
C(week)[T.12]:C(state)[T.Ohio]             -10.0355      1.415     -7.092      0.000     -12.809      -7.262
C(week)[T.13]:C(state)[T.Ohio]              -9.9658      1.495     -6.664      0.000     -12.897      -7.035
C(week)[T.14]:C(state)[T.Ohio]              -4.0223      1.424     -2.824      0.005      -6.814      -1.231
C(week)[T.15]:C(state)[T.Ohio]               0.0145      1.444      0.010      0.992      -2.816       2.845
C(week)[T.16]:C(state)[T.Ohio]              -5.0155      1.445     -3.472      0.001      -7.847      -2.184
C(week)[T.17]:C(state)[T.Ohio]              -3.7995      1.437     -2.643      0.008      -6.617      -0.982
C(week)[T.18]:C(state)[T.Ohio]              -3.9033      1.429     -2.732      0.006      -6.704      -1.103
C(week)[T.19]:C(state)[T.Ohio]              -6.0844      1.507     -4.038      0.000      -9.038      -3.131
C(week)[T.20]:C(state)[T.Ohio]              -6.2755      1.500     -4.185      0.000      -9.215      -3.336
C(week)[T.21]:C(state)[T.Ohio]              -2.1163      1.500     -1.411      0.158      -5.056       0.824
C(week)[T.22]:C(state)[T.Ohio]               6.1703      1.498      4.119      0.000       3.234       9.107
C(week)[T.23]:C(state)[T.Ohio]               1.8412      1.499      1.229      0.219      -1.096       4.778
C(week)[T.11]:C(state)[T.Oregon]           -10.7522      2.366     -4.544      0.000     -15.390      -6.114
C(week)[T.12]:C(state)[T.Oregon]            -0.7938      1.898     -0.418      0.676      -4.515       2.927
C(week)[T.13]:C(state)[T.Oregon]            -7.8745      1.972     -3.994      0.000     -11.740      -4.009
C(week)[T.14]:C(state)[T.Oregon]            -2.8888      1.921     -1.504      0.133      -6.653       0.876
C(week)[T.15]:C(state)[T.Oregon]             4.5226      1.934      2.339      0.019       0.732       8.313
C(week)[T.16]:C(state)[T.Oregon]            -0.5775      1.934     -0.299      0.765      -4.368       3.213
C(week)[T.17]:C(state)[T.Oregon]            -4.3609      1.927     -2.263      0.024      -8.139      -0.583
C(week)[T.18]:C(state)[T.Oregon]            -6.3285      1.905     -3.323      0.001     -10.062      -2.595
C(week)[T.19]:C(state)[T.Oregon]            -3.0123      1.964     -1.534      0.125      -6.861       0.837
C(week)[T.20]:C(state)[T.Oregon]           -13.3560      1.903     -7.019      0.000     -17.086      -9.626
C(week)[T.21]:C(state)[T.Oregon]            -6.8703      1.903     -3.611      0.000     -10.600      -3.141
C(week)[T.22]:C(state)[T.Oregon]            -0.9806      1.903     -0.515      0.606      -4.711       2.750
C(week)[T.23]:C(state)[T.Oregon]            -2.1458      1.900     -1.129      0.259      -5.870       1.578
C(week)[T.11]:C(state)[T.Pennsylvania]      -9.2748      2.046     -4.533      0.000     -13.286      -5.264
C(week)[T.12]:C(state)[T.Pennsylvania]     -10.0204      1.481     -6.767      0.000     -12.923      -7.118
C(week)[T.13]:C(state)[T.Pennsylvania]     -12.5688      1.494     -8.410      0.000     -15.498      -9.639
C(week)[T.14]:C(state)[T.Pennsylvania]      -8.9442      1.489     -6.007      0.000     -11.863      -6.026
C(week)[T.15]:C(state)[T.Pennsylvania]      -6.0280      1.509     -3.996      0.000      -8.985      -3.071
C(week)[T.16]:C(state)[T.Pennsylvania]     -12.6883      1.509     -8.410      0.000     -15.646      -9.731
C(week)[T.17]:C(state)[T.Pennsylvania]     -14.3125      1.500     -9.541      0.000     -17.253     -11.372
C(week)[T.18]:C(state)[T.Pennsylvania]     -13.6265      1.489     -9.150      0.000     -16.546     -10.707
C(week)[T.19]:C(state)[T.Pennsylvania]     -13.4367      1.563     -8.597      0.000     -16.500     -10.373
C(week)[T.20]:C(state)[T.Pennsylvania]     -14.6203      1.520     -9.620      0.000     -17.599     -11.641
C(week)[T.21]:C(state)[T.Pennsylvania]     -11.7296      1.501     -7.812      0.000     -14.673      -8.787
C(week)[T.22]:C(state)[T.Pennsylvania]      -8.5149      1.490     -5.715      0.000     -11.435      -5.595
C(week)[T.23]:C(state)[T.Pennsylvania]     -11.5128      1.515     -7.597      0.000     -14.483      -8.542
C(week)[T.11]:C(state)[T.Rhode Island]      -7.6677      3.282     -2.336      0.019     -14.101      -1.234
C(week)[T.12]:C(state)[T.Rhode Island]      -8.4959      2.963     -2.867      0.004     -14.304      -2.688
C(week)[T.13]:C(state)[T.Rhode Island]      -5.9500      3.002     -1.982      0.047     -11.834      -0.066
C(week)[T.14]:C(state)[T.Rhode Island]      -7.1318      3.106     -2.296      0.022     -13.220      -1.043
C(week)[T.15]:C(state)[T.Rhode Island]      -0.9039      3.114     -0.290      0.772      -7.009       5.201
C(week)[T.16]:C(state)[T.Rhode Island]      -9.5408      3.115     -3.063      0.002     -15.646      -3.435
C(week)[T.17]:C(state)[T.Rhode Island]     -11.0583      3.111     -3.554      0.000     -17.157      -4.960
C(week)[T.18]:C(state)[T.Rhode Island]     -11.9496      2.973     -4.020      0.000     -17.776      -6.123
C(week)[T.19]:C(state)[T.Rhode Island]      -8.9038      3.141     -2.834      0.005     -15.061      -2.746
C(week)[T.20]:C(state)[T.Rhode Island]      -6.5752      3.008     -2.186      0.029     -12.472      -0.679
C(week)[T.21]:C(state)[T.Rhode Island]      -2.1158      3.008     -0.704      0.482      -8.011       3.779
C(week)[T.22]:C(state)[T.Rhode Island]       5.2909      3.007      1.759      0.079      -0.604      11.186
C(week)[T.23]:C(state)[T.Rhode Island]      -5.2747      2.964     -1.780      0.075     -11.084       0.534
[truncated: 1,035,299 more chars]
